# Genomic insights into the physiology of *Quinella*, an iconic uncultured rumen bacterium

**DOI:** 10.1038/s41467-022-34013-1

**Published:** 2022-10-20

**Authors:** Sandeep Kumar, Eric Altermann, Sinead C. Leahy, Ruy Jauregui, Arjan Jonker, Gemma Henderson, Sandra Kittelmann, Graeme T. Attwood, Janine Kamke, Sinéad M. Waters, Mark L. Patchett, Peter H. Janssen

**Affiliations:** 1 AgResearch Limited, Grasslands Research Centre, Palmerston North, New Zealand; 2 School of Natural Sciences, Massey University, Palmerston North, New Zealand; 3 Riddet Institute, Massey University, Palmerston North, New Zealand; 4 Animal and Bioscience Research Department, Animal & Grassland Research and Innovation Centre, Teagasc, Grange, Dunsany, Co, Meath, Ireland; 5 Present Address: Bacthera AG, Basel, Switzerland; 6 Present Address: Wilmar International Limited, WIL@NUS Corporate Laboratory, Centre for Translational Medicine, National University of Singapore, Singapore, Singapore; 7 Present Address: Auckland Council, Auckland, New Zealand

**Keywords:** Bacterial genomics, Bacteriology, Cellular microbiology

## Abstract

*Quinella* is a genus of iconic rumen bacteria first reported in 1913. There are no cultures of these bacteria, and information on their physiology is scarce and contradictory. Increased abundance of *Quinella* was previously found in the rumens of some sheep that emit low amounts of methane (CH_4_) relative to their feed intake, but whether *Quinella* contributes to low CH_4_ emissions is not known. Here, we concentrate *Quinella* cells from sheep rumen contents, extract and sequence DNA, and reconstruct *Quinella* genomes that are >90% complete with as little as 0.20% contamination. Bioinformatic analyses of the encoded proteins indicate that lactate and propionate formation are major fermentation pathways. The presence of a gene encoding a potential uptake hydrogenase suggests that *Quinella* might be able to use free hydrogen (H_2_). None of the inferred metabolic pathways is predicted to produce H_2_, a major precursor of CH_4_, which is consistent with the lower CH_4_ emissions from those sheep with high abundances of this bacterium.

## Abstract (web summary)

Uncultured bacteria of the genus *Quinella* are found in the rumen of ruminant animals, especially in sheep that emit low amounts of methane. Here, Kumar et al. reconstruct genomic sequences from *Quinella* cells to provide insights into their metabolic capabilities, including lactate and propionate formation as major fermentation pathways and an apparent lack of production of H_2_, a major precursor of methane.

## Introduction

*Quinella* is a genus of rumen bacteria that form large oval-shaped cells up to 4 μm in diameter and up to 8 μm long^1^. Their cell volumes are about 60 times greater than *Escherichia coli*, which is often thought of as a standard bacterium (Supplementary Note 1). Cells that are now thought to be members of the genus *Quinella* were first reported in 1913 by Woodcock and Lapage^2^, who observed them during microscopy of goat rumen contents, but mistook them for protistan parasites because of their large cell size. Thirty years later, Quin^3^ observed *Quinella* in the rumen of sheep and thought that these large cells were a pseudo-yeast which she named *Schizosaccharomyces ovis*. Later, it was found that *Quinella* was not a yeast, as it could not be grown in standard yeast media, was motile and stained gram-negative^4,5^. Based on comparative analyses of 16S rRNA gene sequences, *Quinella* was found to be closely related to members of the *Selenomonas*-*Megasphaera*-*Sporomusa* group of bacteria^1^. They are now classified in the order *Selenomonadales* in the class *Negativicutes*^6^. There have been some isolation attempts by Purdom^7^ and Orpin^8^, but no one has succeeded in isolating and maintaining a pure culture of *Quinella*. The taxon named *Quinella ovalis* was described under rule 18a of chapter 3 of the International Code of Nomenclature of Bacteria, which allows the naming of a bacterial taxon without a culture being isolated and deposited in a culture collection^9^. The description of *Quinella ovalis* is based on features observed in various studies, and a partial 16S rRNA gene sequence that was obtained from enriched cells matching that description^1^.

*Quinella* spp. have been detected in the rumen and similar forestomachs using molecular ecology methods. They made up an average of 2.3 to 4.5% of bacteria, based on 16S rRNA gene abundance, in sheep, goats, deer, antelopes, bison, South American camelids and giraffes in the Global Rumen Census^10^ (Supplementary Table 1), and about 4% of 16S rRNA genes detected in rumen samples from sheep grazing pasture in New Zealand^11^. *Quinella* spp. appeared to be much rarer in cattle and water buffalo (Supplementary Table 1). An analysis of the rumen microbiomes of sheep with contrasting methane emissions identified two different community types associated with individual sheep that had low methane yields^12^. One of these low methane yield community types, designated the Q-type, was characterised by large populations of *Quinella* spp., with an average of 32.5% of 16S rRNA genes originating from members of this genus^12^. How this bacterium contributes to low methane emissions in these sheep has not yet been investigated. Two fermentation studies, one in vitro^13^ and the other in vivo^14^, have been conducted to understand *Quinella* physiology. However, the results from these studies are contradictory. Brough et al.^13^ suggested that the preferred substrate, glucose, was fermented mainly to lactate by enriched *Quinella* cells, with traces of acetate, propionate and CO_2_ also detected. In contrast, Vicini et al.^14^ reported that when *Quinella* was present as the most abundant bacteria in sheep fed molasses, acetate and propionate were the major fermentation end-products in the rumen, with no lactate formed. A higher relative ruminal propionate concentration was also found by Kittelmann et al.^12^ in sheep with elevated populations of *Quinella* spp., and this was postulated to be a reason for lower methane yields from those sheep compared to high methane yield sheep that had small *Quinella* populations.

There has been a continual development of methods for the assembly of draft and even complete genomes from metagenomic datasets^15–17^, resulting in genome assemblies for uncultured bacteria. Similarly, genome reconstruction from rumen metagenomic samples^18–20^ has also improved, resulting, for example, in nearly 5000 rumen metagenome-assembled genomes (genome completeness ≥80% and contamination ≤10%) in one study alone^20^.

In the study reported here, we assembled genomes of *Quinella* spp. from DNA extracted from cells that were physically enriched from sheep rumen samples that contained large populations of members of this genus. We then analysed these metagenomically assembled genomes to gain insights into the physiology of *Quinella*.

## Results and Discussion

### Taxonomic definition of *Quinella*

**Fig. 1:**
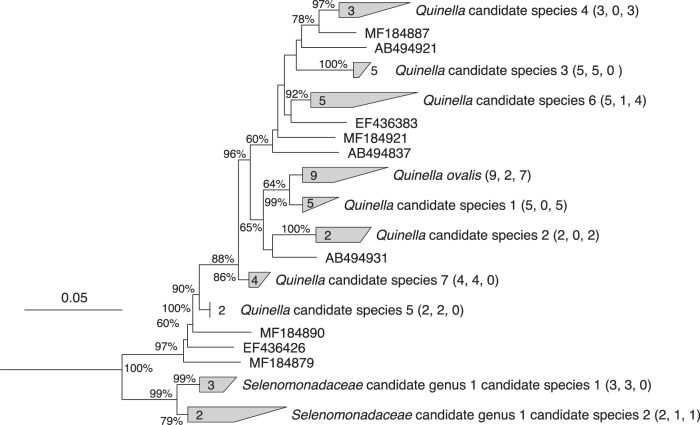
Phylogenetic tree of the genus *Quinella* and relatives. Numbers in brackets represent, in order, the total number of sequences in a particular cluster, the number of sequences from GenBank, and the number of new sequences generated in this study (Supplementary Table 2). The sequences in each cluster are given in Supplementary Fig. 1, while singletons are labelled with their GenBank accessions (sequences from this study are prefixed MF). The tree was generated using the Jukes–Cantor genetic distance model^44^ with the Neighbor-Joining method^45^, implemented in ARB^42^. The numbers at the nodes are the percentage of trees that conserved that node in 1000 bootstrap resamplings. The 16S rRNA gene sequence of *Fibrobacter succinogenes* (GenBank accession CP002158) was used as an out-group sequence. The scale bar represents 0.05 changes per nucleotide position.

To obtain good quality and almost full-length 16S rRNA gene sequences from *Quinella* spp., seven rumen samples from the study of Kittelmann et al.^12^ were selected for clone library construction. Almost full length bacterial 16S rRNA genes were amplified from these samples, and 124 clones were sequenced and apparent chimeras removed. Twenty-six non-chimeric sequences were possibly affiliated to the genus *Quinella* (Supplementary Table 2) based on an initial BLAST analysis using a database of 16S rRNA gene sequences with improved resolution of rumen bacteia^21^. These new sequences plus an additional 23 sequences from public databases resulted in a set of 49 potential *Quinella* sequences >1443 nt long that were used to generate a phylogenetic tree of *Quinella* and some of its relatives (Fig. 1). Different authors use different sequences similarity cut-offs to define species- and genus-level taxa, varying from 93% to 98%^22,23^. Conservative sequence similarity cut-offs of 97% similarity for species and 93% similarity for genera were used in this study. Six of the newly cloned *Quinella* sequences clustered with the reference sequence of *Quinella ovalis* (GenBank accession M62701), with sequence similarities ≥97.3%, and a seventh with a sequence similarity of 96.9% to that reference sequence (Fig. 1, Supplementary Fig. 1). Based on a criterion that sequences with similarities of ~97% or more may originate from the same species, these seven new sequences plus two other sequences from GenBank could be designated as *Q. ovalis*. The remaining sequences likely represent seven other candidate species of *Quinella* if only clusters with two or more sequences are considered. Three of the seven candidate species in the refined *Quinella* tree contained sequences only from the newly formed clone libraries, and the sequences in those originated from two different individual sheep rumen samples each. Sequences in *Quinella* candidate species 3 and *Quinella* candidate species 5 were from one single study^24^. In addition, there were multiple sequences that did not group with these eight clusters (Fig. 1) and may represent other species if they are not amplification artefacts. Overall, this analysis suggests that there are multiple species of the genus *Quinella*, and more intensive investigation and generation of high-quality sequences would help confirm this. Additionally, one potentially new genus-level cluster (*Selenomonadaceae* candidate genus 1) was found that was closely related (>92.4% similarity) to *Quinella*. There appeared to be two species-level clusters within this potentially new genus (Fig. 1, Supplementary Fig. 1).

The refined phylogenetic tree of *Quinella* spp. (Fig. 1) was used as a reference to compare the distribution and abundance of these species in 236 sheep rumen samples based on short reads of 16S rRNA genes (~400 bp) that had previously been generated from 118 sheep by Kittelmann et al.^12^. These findings suggest that many more potential *Quinella* species may exist for which no long-length 16S rRNA gene sequences were obtained in that study (Supplementary Note 2, Supplementary Fig. 2).

### Physical enrichment of putative *Quinella* cells from rumen samples

**Fig. 2:**
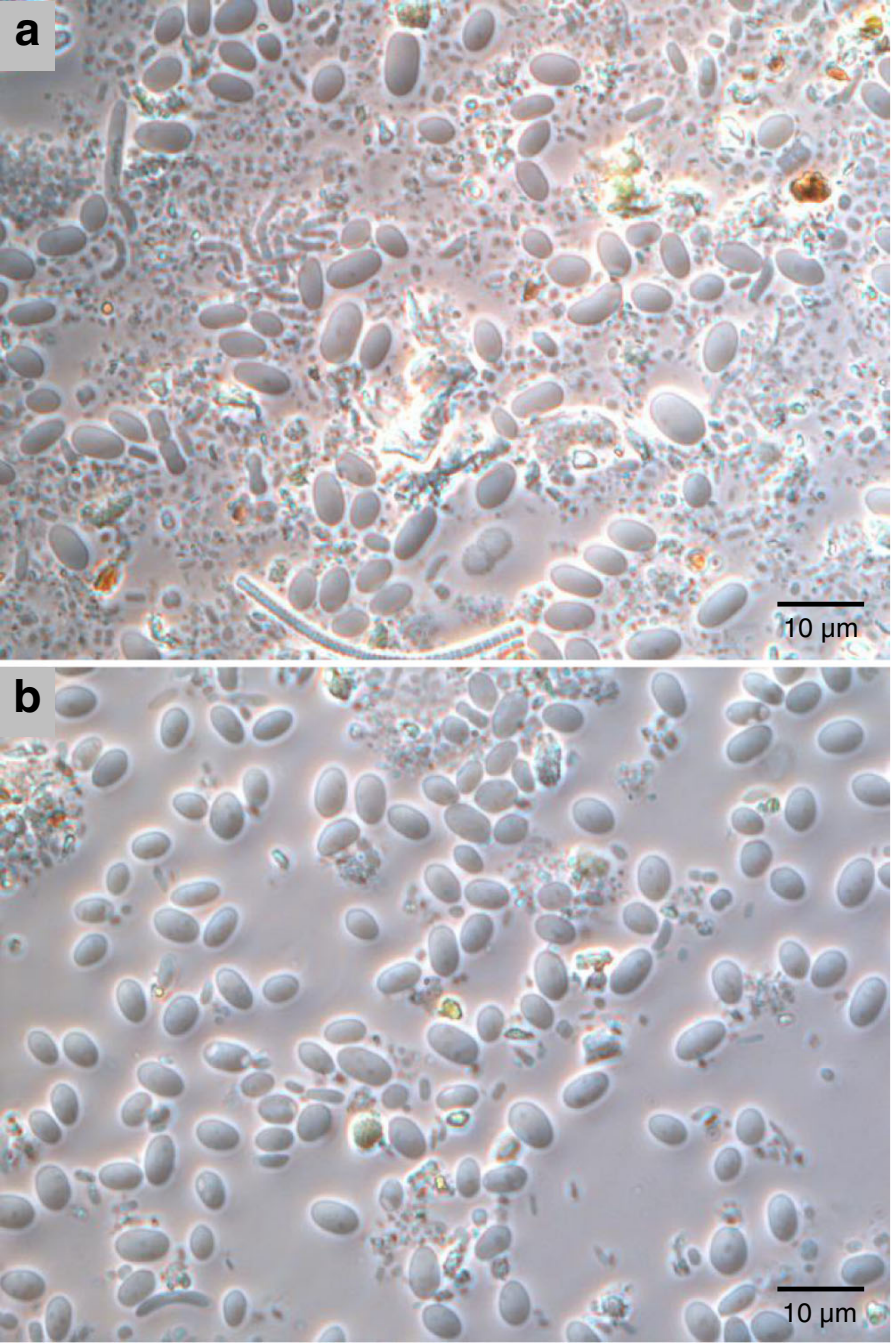
Phase contrast images of enriched *Quinella*-like cells. **a** Supernatant after the first filtration through 23-µm pore size nylon mesh and subsequent centrifugation (100 × *g*). **b** *Quinella*-like cells after the enrichment protocol was completed. The scale bar indicates a distance of 10 μm. Panel **b** was from sample 3, which was prepared from rumen contents (**a**), and similar results were observed with samples 1 and 2. This photo was selected to illustrate the enrichment outcome. Untreated samples from 12 sheep looked similar to panel (**a**).

Three preparations (designated samples 1, 2 and 3; Table 1) of enriched *Quinella* cells were made from freshly collected rumen contents samples using a modification of Wicken and Howard’s physical enrichment method^4^. Freshly collected rumen contents were filtered through polyester mesh and large cells collected using low-speed (800 × *g*) centrifugation steps, as described in the Methods. This cell concentration method resulted in a visual increase in the relative abundance of oval cells about 3–5 μm long, which we postulated to be *Quinella* (Fig. 2). Between 43 and 57% of 16S rRNA gene sequences amplified from these samples originated from *Quinella* spp. (Table 1). This was determined by phylogenetic analysis of these sequences (see next section).

**Table 1:** 16S rRNA gene clone libraries prepared from *Quinella*-enriched samples

Sample	Description	No. of clones sequenced	No. of *Quinella* clones identified^*a*^	Inferred *Quinella* abundance (%)
1	Pooled samples from 12 sheep	111	51	45.9
2	Pooled samples (two samplings 2 weeks apart) from one sheep	95	41	43.2
3	Single rumen sample from one sheep	92	52	56.5

^a^GenBank accession numbers OM320214 to OM320357.

### Diversity of *Quinella* in the enriched samples

The diversity of *Quinella* in the enriched samples was investigated by importing the 155 *Quinella*-affiliated cloned sequences derived from the three *Quinella*-enriched samples (Table 1) into the refined long-length *Quinella* phylogenetic tree (Fig. 1), using the parsimony insertion tool in ARB. Imported cloned sequences were distributed throughout the tree (Supplementary Fig. 3), suggesting that the samples contained more than one *Quinella* species. These samples might therefore be suitable to gain a general insight into the metabolic potential of *Quinella*, by reconstructing genomes from DNA extracted from these samples. However, the diversity suggested that generation of genomes from single strains should not be expected.

### Fluorescence in situ hybridisation to confirm the presence of *Quinella* in concentrated rumen samples

**Fig. 3:**
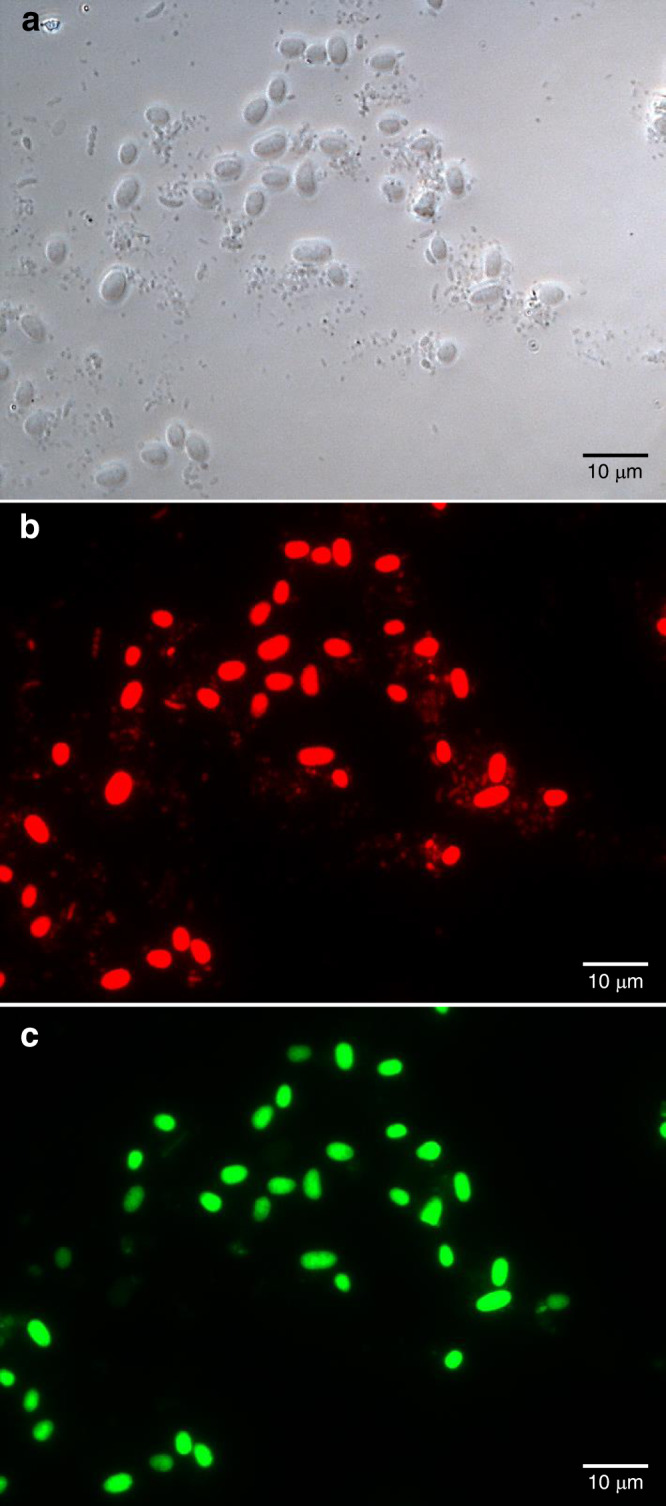
Micrographs of concentrated *Quinella* cells labelled with probes targeting rRNA. The preparation was enriched for large cells using a sample from the rumen of a sheep with a large population of *Quinella*. **a** Phase contrast image of the enriched preparation. **b** The same field showing cells that hybridised with the Cy3-labelled universal bacterial probe EUB338. **c** The same field showing cells that hybridised with the Alexa 488-labelled *Quinella*-specific probe Quin1231. The scale bar indicates a distance of 10 μm. This image was selected to illustrate the binding of probe Quin1231 to the large oval cells, and similar results were obtained using rumen samples from 6 other sheep.

Fluorescence in situ hybridisation (FISH) microscopy was used to confirm that the large cells in the enriched samples were *Quinella* spp., using DNA probes that bind to 16S rRNA in the cells (Fig. 3). Probes Quin1231, which was designed to match to 16S rRNA of *Quinella* (Supplementary Note 3, Supplementary Table 3), and EUB338, which binds to 16S rRNA from most bacteria^25^, were distinguished by labelling them with different fluorophores. They were then used together to label cells concentrated from rumen contents. Probe Quin1231 hybridised with the large oval cells in the enriched sample 3 (Fig. 3c), whereas other bacterial cells can be clearly seen in the phase contrast image of the same field (Fig. 3a) and clearly hybridised with the universal bacterial probe (Fig. 3b). Sizes of *Quinella* cells in rumen samples from 7 different sheep were measured. The mean length of 114 cells that bound the Quin1231 probe was 4.2 μm (SD = 0.75, min = 2.9, max = 6.5) and the mean diameter was 2.8 µm (SD = 0.44, min = 1.6, max = 3.9). The mean calculated cell volume was 18.4 μm^3^ (SD = 8.4, min = 5.8, max = 46.7). These cells occurred singly and not in noticeable clusters, pairs or chains. No cells bound the nonsense probe, nonEUB338.

### Ultrastructure of *Quinella*

Sample 3 was used to prepare material for scanning electron microscopy (SEM) and transmission electron microscopy (TEM) to study cell size and structure more closely. The SEM and TEM images also suggested that the enrichment method worked well because a large proportion of the cells observed were the large oval type postulated to be *Quinella*. These had a range of sizes and outer cell surface textures, which may reflect cells at different stages in the growth cycle or perhaps different species.

**Fig. 4:**
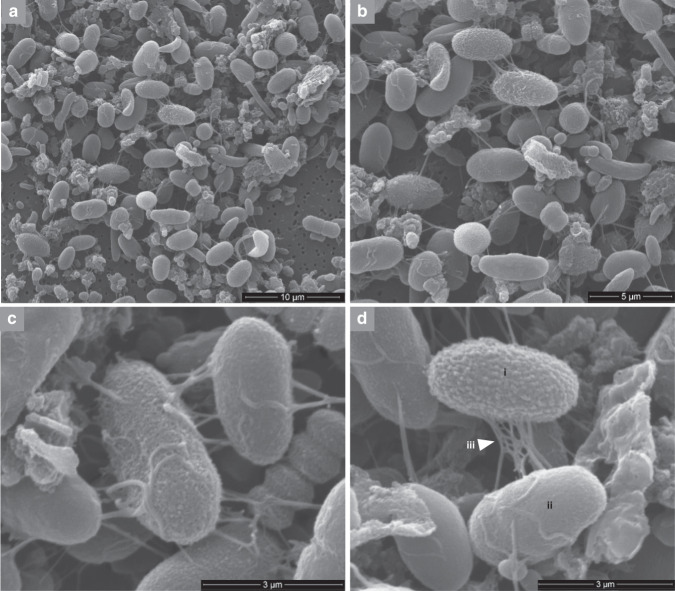
Scanning electron microscopic images of putative *Quinella* cells and other cells in sample 3. Panels **b**–**d** are magnified views of panel **a**. Features: (i) granular cell type; (ii) smooth cell type; (iii) unidentified material peeling off from cell surface, possibly a dehydrated glycocalyx or denatured surface associated proteins. The scale bars indicate different distances for each panel. SEM was performed only on sample 3, and these images were selected to illustrate the different features observed.

SEM images revealed that the cells were 3–5 µm long and 1–2 µm in diameter (Fig. 4), similar to the sizes observed using light microscopy. These are smaller than the cells reported by Orpin^8^, which were 5.8–8.0 µm long and 2.5–6.0 µm in diameter. It was interesting to find that, unlike *Selenomonas ruminantium* (the cultured genus closest to *Quinella* in phylogenetic analyses), *Quinella* in these preparations did not have thick and complex flagella (curled-up and 20 nm in diameter), and it is unclear if these *Quinella* cells possessed flagella. The flagella may have been sheared off during the cell concentration process, which included filtering and centrifugation steps. *Quinella* has previously been observed to be motile but the presence of flagella was not reported^8^. Some *Quinella* cells were fully covered with a granular material while others were either partially covered or had a smooth cell surface (Fig. 4d), but both types were similar in shape and size. Surface structures were visible, and these may be artefacts of preparing the cells for SEM, a polysaccharide layer, or some other structure.

**Fig. 5:**
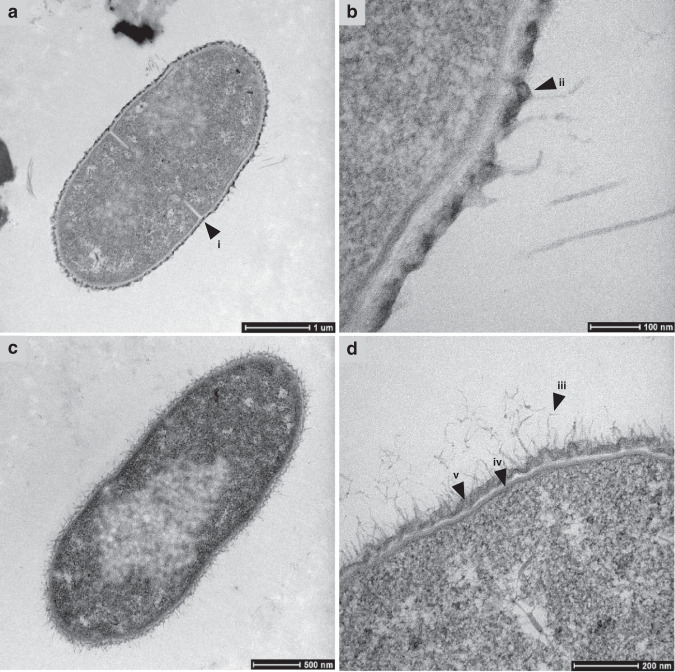
Transmission electron microscopic images of putative *Quinella* cells from sample 3. Panel **b** is a magnified view of part of panel **a**, while panels **c** and **d** are from different cells. Features: (i) division site resembling those found in Gram-positive bacteria^72^; (ii) electron-dense material on outer membrane; (iii) possible fimbriae or outer surface material; (iv) cytoplasmic membrane; (v) outer membrane. The scale bars indicate different distances for each panel. TEM was performed only on sample 3, and these images were selected to illustrate the different features observed.

In TEM images, two different types of cell surfaces were also observed. Some *Quinella* cell surfaces were covered with an uneven electron-dense layer (Fig. 5a, b), while in others this was absent (Fig. 5c, d). Cells of both types had short tuft-like surface structures, but these were more prevalent on the cells without the electron-dense layer. The surface features observed by SEM and TEM may be strands of a surface capsule or similar material.

The cells had a thin cell envelope probably composed of peptidoglycan, sandwiched between inner and outer cell membranes (a characteristic of Gram-negative bacteria), confirming that *Quinella* spp. possess a Gram-negative cell wall structure (Fig. 5), like its cultured relatives in the family *Selenomonadaceae*. *Quinella* belongs to the phylum *Firmicutes*, which contains mainly Gram-positive bacteria, but it is placed in the class *Negativicutes*, which contains bacteria with Gram-negative cell wall structures^6^. Unlike most other members of this class, it is oval in shape, rather than spherical like *Megasphaera* and *Veillonella* or curved or crescent shaped rods like *Selenomonas* and *Sporomusa*^6^. Notably, *Quinella*-like cells showed Gram-positive type cell division even though they have a Gram-negative cell wall structure (Fig. 5a). Condensed material observed inside the cells in the TEM images might be genomic DNA dividing between future daughter cells (Fig. 5a, c).

### Attempts to culture *Quinella*

The sheep that were the source of sample 1 (Table 1), and had large populations of *Quinella* based on microscopy, were sampled to provide inocula for the cultivation attempts. This was unsuccessful and the cultures of large cells that were isolated (not as large as *Quinella*) were identified as *Staphylococcus warneri*, with >97.6% sequence identity to 16S rRNA genes from *S. warneri* over the first 500 bp of the gene.

### Construction of *Quinella* genome bins from concentrated *Quinella* samples

The three samples of concentrated *Quinella* cells (Table 1) were used to generate metagenome-assembled genomes. DNA was extracted from these samples and sequenced using Illumina MiSeq technology to produce >4.5 million pairs of 300 nt reads per sample (Supplementary Table 4). The raw reads were quality checked, trimmed, assembled and assigned to 85 bins, and 34 bins with size >1 Mbp were selected for further analysis (Supplementary Tables 5 and 6). Genomic phylotyping of the bins showed that reads from members of the family *Selenomonadaceae* were the most abundant on the basis of homology to marker genes (Supplementary Fig. 4). As expected, some other common rumen bacterial families^10^ were also detected in genomic bins, for example, *Spirochaetaceae*, *Eubacteriaceae*, *Lachnospiraceae*, and *Clostridiaceae*. Thirteen bins were identified as containing possible genomic DNA from *Quinella* (five from sample 1, and four each from samples 2 and 3; Supplementary Table 7). These bins contained partial 16S rRNA gene sequences that matched those of *Quinella* spp. Following the proposed genome quality classification scheme of Parks et al.^15^, four bins were considered nearly (≥90%) complete with medium (5% to 10%) to low (≤5%) contamination (Table 2; Supplementary Tables 6 and 7, Supplementary Note 4). These were selected for further study: bin 5 from sample 1 (designated bin SR1Q5), bin 7 from sample 1 (SR1Q7), bin 5 from sample 2 (SR2Q5) and bin 1 from sample 3 (SR3Q1). Among other *Quinella* bins, bin 2 from sample 1, bin 18 from sample 2 and bin 5 from sample 3 were 100% complete but at the same time they were highly contaminated (≥48%). Post-binning strategies^16^ were applied to reduce contamination from these bins, but these proved to be unsuccessful and so these bins were not used.

**Table 2:** *Quinella* genome bins

Genome bin character	*Quinella* genome bins			
	SR1Q5	SR1Q7	SR2Q5	SR3Q1
Genome bin size (bp)	2,125,473	2,584,672	1,821,931	2,614,227
G + C content (mol%)	49.0	52.9	56.0	49.1
Number of contigs	132	169	42	68
Length of largest contigs (bp)	62,332	68,294	179,392	219,728
Genome completeness (%)	90.9	94.2	92.6	91.4
Genome contamination (%)	5.7	8.6	0.2	10.3
Strain heterogeneity (%)	48.5	77.8	0	78.4

The four selected genome bin varied in size from 1.8 to 2.6 Mbp (Supplementary Note 5, Supplementary Fig. 5). However, the larger bins also showed greater heterogeneity, suggesting that the bin sizes could have been inflated by sequences from other genomes. The G + C contents of these bins also varied (Supplementary Note 6, Supplementary Fig. 5).

### Confirmation of identities of *Quinella* in genome bins

Partial 16S rRNA sequences extracted from the sequence data in the four selected *Quinella* genome bins were compared against a curated bacterial 16S rRNA gene database^21^. All four 16S rRNA sequences showed best matches with 16S rRNA genes from *Quinella* spp., with e-values of 0 (SR1Q7 and SR2Q5), 6.00E−61 (SR1Q5) and 4.00E−46 (SR3Q1). To obtain full-length 16S rRNA gene sequences, the genome bin sequence information was used to design primers (Supplementary Table 8, Supplementary Fig. 6a), which were then used to generate clone libraries containing almost full-length 16S rRNA gene sequences plus diagnostic flanking regions from the DNA extracted from samples 1–3. The DNA fragments, containing 16S rRNA genes plus flanking regions, were of the expected sizes (1851–2390 bp long) (Supplementary Fig. 6b). Two 16S rRNA gene libraries were prepared from sample 1, using two sets of primers targeting the unique 16S rRNA gene sequences found in each of bins SR1Q5 and SR1Q7. Two further different primer sets were used to target 16S rRNA genes in bin SR2Q5 and SR3Q1 from samples 2 and 3, respectively. This produced one library targeting the 16S rRNA gene and flanking regions for each of the four genome bins. Cloned amplicons were sequenced using multiple primers. Five clones from each of the four clone libraries were selected based on the electropherogram quality, and on matches to the flanking regions and partial 16S rRNA genes in the bin that was being targeted, and these sequences were used for the phylogenetic analysis (Supplementary Note 7, Supplementary Table 9).

The 20 cloned sequences representing the four *Quinella* genome bins grouped in different parts of the tree (Supplementary Fig. 7), as expected if the *Quinella* genome bins were from separate *Quinella* species. All five 16S rRNA clone sequences assigned to SR1Q5 grouped together and branched with *Quinella ovalis* with >97% similarity with other sequences in that cluster (excluding sequences Unl25493; >96%). Genome bin SR1Q5 may therefore contain genomic sequences that represent strains of the species *Quinella ovalis* or a very close relative of it. Based on the placement of the other 16S rRNA genes, it appears that the other three genomic bins represent three different species of the genus *Quinella* (Supplementary Figs. 7 and 8). These 16S rRNA gene sequences from the genome bins were also related to the genes of *Quinella* spp. detected in an earlier study on sheep (Supplementary Note 8, Supplementary Fig. 9). The genome bins therefore represent common *Quinella* species in sheep rumen. Genome Taxonomy Database (GTDB, release 207)^26^ classifies the three genome bins with <10% contamination as *Quinella* within the family *Selenomonadaceae*, in agreement with the relationships deduced by 16S rRNA gene-based analyses. All four grouped within *Selenomonadaceae* using genome taxonomy implemented in GTDB-Tk^27^.

### *Quinella* genome bin annotation

A summary of the genome bin annotation is shown in Supplementary Table 10, and a list of all genes is given in Supplementary Data 1. Of the 8761 complete genes identified in *Quinella* genome bins, 28.2 % were predicted to be of unknown function and annotated as hypothetical. It was interesting to find that, even though the genome bins varied in size, they all shared similar percentages of genes for particular COG (Clusters of Orthologous Groups) categories (Supplementary Table 11). Furthermore, of 1058 gene families from all four *Quinella* genome bins, 330 gene families were present in all four genome bins (Supplementary Fig. 10). Genome bins SR3Q1 and SR1Q7 were found to be different from SR1Q5 and SR2Q5 in terms of the numbers of unique gene families. This finding was also supported by a Functional Genome Distribution (FGD) analysis (Supplementary Fig. 11), where genome bins SR1Q5 and SR2Q5 were more similar to each other, and SR1Q7 and SR3Q1 were more similar to each other. FGD calculates the similarity between pairs of genome bins using amino-acid sequences predicted from the ORFeome^28^. This is a BLAST-based ORF-position-independent approach based on amino-acid sequence similarities of ORFs, compared between genomes, and is considered to be a function-based analysis. The groupings in the FGD tree were slightly different from the 16S rRNA gene-based analysis, in which SR1Q7and SR3Q1 were close to each other while SR1Q5 and SR2Q5 branched separately. Thus, these *Quinella* species in the bins are potentially not functionally identical.

Nearly all genes required for synthesis of a complete flagellum was found in the four genome bins (Supplementary Fig. 12), and analysis using EffectiveT3 and EffectiveS346^29^ found no genes coding for diagnostic components of a type III secretion system. While this does not prove that functional flagella were observed by electron microscopy (Fig. 4), the completeness suggests they can be produced. The presence of MotA and MotB suggest that the flagellum is H^+^ driven^30^, supported by the absence of the Na^+^-specific MotX and MotY^31^. CAZyme analysis (Supplementary Table 12) of the genome bins indicated the presence of enzymes involved in the formation of lipopolysaccharide and surface sugar polymers (Supplementary Table 13), like lipid A disaccharide synthase (CAZyme family GT19), lipopolysaccharide heptosyltransferases I, II, and III (GT9), tetraacyldisaccharide 4′-kinase (GT30), and other glycosyl transferases (GT4, GT8, GT26, GT41, GT83). These may better explain the surface structures observed by electron microscopy.

### Genome bin analysis to deduce the fermentation pathway of *Quinella* spp

**Fig. 6:**
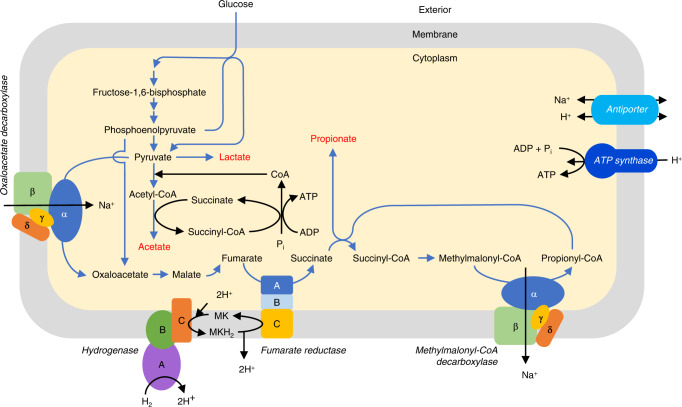
Schematic showing the glucose fermentation pathway of *Quinella*. The construction is based on the four *Quinella* genomic bins and analyses presented in this paper. The proposed end products are shown in red font, carbon flow is indicated by blue arrows, other transfers by black arrows, and some enzymes are labelled in italic font. The subunits and functions of the oxaloacetate decarboxylase and methylmalonyl-CoA decarboxylase (Supplementary Note 14), fumarate reductase (Supplementary Note 15), and hydrogenase (Supplementary Note 19) are described in more detail in the Supplementary Information. The colours of enzyme subunits are solely to differentiate them more readily. P_i_ inorganic phosphate, MK is a postulated menaquinone.

The analysis of the four genome bins allowed a metabolic scheme for *Quinella* to be constructed (Fig. 6; Supplementary Fig. 13, Supplementary Table 14). CAZymes analysis of all four *Quinella* genomes suggested that *Quinella* spp. may not degrade polysaccharides and so are dependent on other rumen microbes to break down the polysaccharide components of feed, and then use the breakdown products for growth (Supplementary Note 9, Supplementary Tables 12 and 13). However, *Quinella* may be able to use cellobiose and cellodextrins released by other rumen microbes using a β-glucosidase (Supplementary Table 15). Genes coding for enzymes in the phosphotransferase system (PTS) for glucose, sorbitol, fructose, maltose, mannose, galactitol, and ascorbate transport were present in *Quinella* genome bins, further indicating that members of this genus use smaller carbohydrates released by polysaccharide-degrading microbes (Supplementary Table 16). These carbohydrates seem to be converted to pyruvate by glycolysis (Supplementary Note 10). All *Quinella* genome bins also contained a gene for L-lactate dehydrogenase (Supplementary Note 11, Supplementary Fig. 14), suggesting that they can produce lactate as an end product, use lactate as a substrate, or even both (Supplementary Fig. 15). Previous work reported that *Quinella* may produce lactate^13^, and this genomic analysis supports that. The potential use of lactate will require further studies, preferably with cultures of these bacteria.

All four *Quinella* genomic bins contained genes that code for pyruvate:ferredoxin oxidoreductase, which converts pyruvate to acetyl-CoA (Supplementary Note 12, Supplementary Fig. 16). We could not find evidence for the much more common formation of acetate (and perhaps propionate) via the standard phosphate acetyl transferase and kinase steps. Instead, acetyl-CoA appears to be converted to acetate using succinate CoA-transferase (Supplementary Fig. 17), with concurrent conversion of succinate to succinyl-CoA. Succinyl-CoA synthetase then generates ATP from ADP with the release of succinate and CoA (Supplementary Fig. 18). This reaction is found in helminths, protists and fungi^32^. *Selenomonas ruminantium*, a close relative of *Quinella*, may also have this succinyl-CoA cycle for generating ATP^32^.

*Quinella* does not appear to produce formate, ethanol, or butyrate (Supplementary Note 13, Supplementary Fig. 19). It seems to form propionate to balance the electrons released in conversion of glucose to pyruvate and pyruvate to acetyl-CoA (Supplementary Fig. 20). This balance would result in the formation of acetate and propionate in a ratio of 1 to 2 (Supplementary Fig. 15b). All the enzymes needed to form propionate were present in all four *Quinella* genome bins. Formation of oxaloacetate is a key step in this pathway, either from phosphoenolpyruvate via pyruvate or directly from phosphoenolpyruvate. Both mechanisms seem possible in *Quinella* (Supplementary Fig. 13). If there is a functional oxaloacetate decarboxylase (OACD) in *Quinella*, it appears to have an unusual structure (Supplementary Note 14, Supplementary Figs. 21–25). The OACD enzyme in *Quinella* may be a hybrid of the α subunit of OACD, and the β, γ and δ subunits of methylmalonyl-CoA decarboxylase (MMCD). All other key enzymes in the propionate formation pathway, including the Na^+^-translocating MMCD (Supplementary Note 14), fumarate reductase (Supplementary Note 15), and a quinol-fumarate oxidoreductase (Supplementary Note 16, Supplementary Table 17, Supplementary Figs. 26 and 27) were present. *Quinella* does not appear to be able to directly use the Na^+^ gradient that might be generated by the MMCD because the ATP synthase appears to be H^+^-coupled (Supplementary Note 17, Supplementary Fig. 28). Instead, the gradient may be used to drive oxaloacetate formation from pyruvate by OADC (Fig. 6) or converted into a H^+^ gradient by a Na^+^/H^+^ antiporter (Supplementary Note 18). Overall, these analyses indicate that *Quinella* uses the randomizing pathway of propionate formation. An earlier study of sheep rumens dominated by *Quinella* indicated that this bacterium may be able to form propionate^14^, and this study supports that.

Formation of lactate (Supplementary Fig. 15a) or of acetate and propionate (Supplementary Fig. 15b) will not result in production of hydrogen, one of the major precursors of CH_4_ formation, explaining how *Quinella* might be functioning inside the low CH_4_ emitting sheep rumen. However, the *Quinella* genomes contain the genes that code for a hydrogenase system. Interestingly, it seems that the *Quinella* genomes encode enzymes that could allow uptake of exogenous hydrogen using a putative NiFe membrane-bound uptake hydrogenase, which would transfer electrons to cytochrome *b* (Supplementary Note 19, Supplementary Figs. 29–32), and then further to a fumarate reductase (Fig. 6). This would allow the production of more propionate by using exogenous hydrogen, at the expense of acetate (Supplementary Fig. 15c). Greening et al.^33^ found that the most highly expressed H_2_ uptake system in a group of sheep studied by Kamke et al.^34^ were Group 1d hydrogenases from the order *Selenomonadales*. We analysed the transcriptome data from that study by adding our new *Quinella* hydrogenases to the HydDB database of Greening et al.^33^ and found that 26% of metatranscriptome reads assigned to these Group 1d bacterial uptake hydrogenases were likely from *Quinella* spp. This is a remarkably large proportion considering that the sheep in that study had mainly the S-type low CH_4_ microbiome type rather than the *Quinella*-rich Q-type^34^. The presence of an active uptake hydrogenase needs to be demonstrated in a pure *Quinella* culture, which will also confirm the directionality of the hydrogenase (hydrogen uptake, hydrogen producing, or bidirectional). Formate is energetically equivalent to hydrogen and is also formed in the rumen^35^. Genome bin SR3Q1 contained genes that might indicate the ability to use formate (Supplementary Note 9), but this may also be a result of a contamination and so cannot be considered a likely property of the genus.

The overall pathway for hexose fermentation to propionate is very similar to those of *Selenomonas* and *Prevotella*^32^. However, we did not find evidence for genes encoding the Rnf complex (Supplementary Note 20). How reduced ferredoxin or NADH are recycled during propionate formation is still unclear, but direct use of NADH for fumarate reduction has been proposed for *Fibrobacter succinogenes*^32^. *Quinella* would thus forego additional ATP generation through Na^+^ or H^+^ translocation at this step^32^. This lower ATP yield could result in a rate-yield trade-off^36^, allowing *Quinella* to be less efficient per hexose fermented but increase its overall rate of ATP formation. These speculations remain to be investigated.

Based on the inferred functions of the genes found in the genome bins, *Quinella* may carry out three different possible pathways of end-product formation. These are fermentation to form lactate (Supplementary Fig. 15a), fermentation to form acetate plus propionate (Supplementary Fig. 15b), or metabolism of glucose plus hydrogen to form propionate (Supplementary Fig. 15c). The first two of these are standard and well-known metabolic schemes, but the third, the use of hydrogen plus glucose, is not. These different pathways of lactate, acetate and propionate production result in the conservation of different amounts of ATP. Lactate formation from glucose yields 2 ATP while formation of propionate and acetate, or just propionate produce ~2.7–4.4 ATP (Supplementary Table 18). If lactate is used as a substrate then electrons from the lactate to pyruvate and pyruvate to acetyl-CoA conversions may be used in the propionate pathway (Supplementary Fig. 15d), yielding 0.33 to 0.66 ATP (Supplementary Table 18). Lactate could, however, theoretically be used together with hydrogen to form only propionate (Supplementary Fig. 15e) with formation of up to 1.2 ATP (Supplementary Table 18). Some of these pathways might operate at the same time, producing a mix of products. For example, simultaneous formation of lactate, acetate and propionate from sugars seems feasible, as does the use of hydrogen plus sugars with variable amounts of hydrogen to yield different ratios of acetate to propionate.

Attempts to culture *Quinella*, performed by Purdom^7^, Orpin^8^, and in our work, have been unsuccessful, so the information gathered using *Quinella* genome bins analyses may play a useful role in future isolation attempts. The activity of *Quinella* in the rumen still needs to be confirmed by metatranscriptomic analyses of low CH_4_ emitting sheep containing the *Quinella*-dominated (Q-type) community (see Kittelmann et al.^12^), much like Kamke et al.^34^ analysed gene expression in sheep with *Sharpea*- and *Kandleria*-enriched (S-type) rumen communities found in the sheep studied by Kittelmann et al.^12^. The genome bins generated here will help with mapping transcripts because they provide genomic data for *Quinella*. In addition, since *Quinella* spp. seem to be associated with increased relative propionate formation and low methane yields in sheep^12^, these genome bins may be useful for rumen microbiome-based prediction and selection of farmed sheep with smaller environmental impacts^37^.

## Methods

Sample size was not predetermined using statistical methods and the experiments were not randomised. The investigators were not blinded to sample identity and assessment of outcomes.

### Ethics approvals and sampling

All procedures involving animals were approved by the AgResearch Grasslands Animal Ethics Committee, Palmerston North, New Zealand, and adhered to the guidelines of the 1999 New Zealand Animal Welfare Act and AgResearch Code of Ethical Conduct. The collection of rumen contents from fistulated cows for culture media preparation was approved under animal ethics approval AE13398. The collection of rumen samples from sheep for metagenomic analyses, cultivation attempts, and microscopy was under animal ethics approvals AE11975 and AE13282. Samples of rumen contents were collected by oral stomach intubation from sheep fed lucerne pellets in a study described by Jonker et al.^38^ and kept on ice immediately after collection and transported to the laboratory within 30 min.

### Physical enrichment of *Quinella* cells

A method for the physical enrichment of *Quinella* cells based on differential centrifugation^4^ was adapted for this study. Rumen content samples collected from sheep for *Quinella* enrichment were transported to the laboratory on ice and squeezed through 300-µm PETEX polyester mesh (Sefar, Edling, Germany). The filtrate was diluted 1:1 with PBS (137 mM NaCl, 2.7 mM KCl, 8 mM Na_2_HPO_4_, and 2 mM KH_2_PO_4_) and left at room temperature for 10 min. The diluted filtrate was then filtered through 23-µm pore size polyester mesh into clean sterile 50-mL tubes (40 mL sample/tube) and centrifuged at 100 × *g* for 5 min at room temperature to pellet protozoa and particulate materials. The supernatant was decanted, filtered again through 23-µm mesh and centrifuged at 800 × *g* for 5 min. The supernatant was discarded and the pellet (containing *Quinella*-like cells) was washed three times by resuspending in 40 mL PBS buffer and then filtered through 23-µm mesh and centrifuged at 800 × *g* for 5 min to collect the pellet. *Quinella* abundance was monitored at each step by visualising samples using light microscopy (with a 100 × oil immersion lens) for large oval *Quinella*-like cells. Finally, cells were re-suspended in PBS and used for light or electron microscopy or stored at −20 °C until used.

### *Quinella*-enriched samples

For metagenomic and *Quinella* cultivation experiments, rumen samples were collected from 24 13-month-old female sheep. Twelve of these contained large numbers of oval *Quinella*-like cells (assessed using phase contrast microscopy; see below) and were used for the cultivation attempt (see below) and also pooled and processed through the cell enrichment method, explained above. This enriched sample was designated sample 1. Two rumen samples from one sheep that appeared to contain a high proportion of *Quinella* cells (determined by phase contrast microscopy) were collected two weeks apart. These were pooled and enriched to produce sample 2. A single rumen sample from a different sheep that contained *Quinella* cells (based on phase contrast microscopy) was enriched in the same way to produce sample 3.

The relative abundance of *Quinella* in the cell suspensions (samples 1, 2 and 3) produced by the enrichment method was estimated by preparing 16S rRNA gene clone libraries. Briefly, DNA was extracted from these samples using Genomic-tips 100/G (Qiagen, Hilden, Germany). Concentrated *Quinella* cells were first pelleted by centrifugation at 5000 × *g* for 7 min then snap-frozen in liquid nitrogen before the cell pellet was ground for 1 min using a mortar and pestle. The pellet was refrozen using a small amount of liquid nitrogen and ground again a further 7 times. The final ground material was collected from the mortar using 11 mL of buffer B1 (Qiagen; containing 22 µl of 1 mg/mL RNase A). Subsequent DNA extraction was carried out using the Qiagen Genomic-tip 100/G kit (Qiagen) according to the manufacturer’s instructions for bacteria. Extracted DNA concentration and purity were checked using an ND-1000 spectrophotometer (NanoDrop Technologies Inc., Wilmington, DE, USA). These DNA samples were used to generate clone libraries (see below) using the universal bacterial primers 27F and 1492R and PCR conditions listed in Supplementary Table 19.

### Further 16S rRNA gene sequences from *Quinella* spp

Seven sheep rumen samples (Supplementary Table 20) from the study reported by Kittelmann et al.^12^ were selected for clone library construction, to obtain almost full-length good quality 16S rRNA gene sequences of *Quinella* spp. from DNA that had been extracted by Kittelmann et al.^12^ and stored at −80 °C. 16S rRNA genes were amplified using the universal bacterial primers 27F and 1492R and PCR conditions listed in Supplementary Table 19, and cloned and then sequenced using primer 514R as described below. Clones of interest were then fully sequenced.

Short reads of bacterial 16S rRNA genes (~400 bp) that had previously been generated from 236 rumen samples from 118 sheep by Kittelmann et al.^12^ were used to further assess the potential diversity of *Quinella* spp. (GenBank BioProject PRJEB4486).

### Clone library construction and sequencing

PCR products were checked by agarose gel electrophoresis for quantity, size and the presence of single bands, and then purified using the Wizard SV Gel and PCR Clean-Up System (Promega, Madison, WI, USA) following the manufacturer’s instructions. Gel images were captured using UV transillumination a Gel Logic 200 imaging system (Eastman Kodak, New York, NY, USA) with Camera Control Pro 2 V2.7 (Nikon Corporation, Tokyo, Japan) and -Lightroom 2.15.0 (Adobe, San Jose, CA, USA) software. Purified PCR products were ligated into the pCR2.1-TOPO cloning vector (TOPO-TA cloning kit; Life Technologies, Carlsbad, CA, USA), following the manufacturer’s instructions. Ligated plasmids were then transformed into One Shot TOP10 chemically competent *Escherichia coli* cells (Life Technologies) following the manufacturer’s instructions. Transformed cells were plated onto LB agar plates (10 g Bacto-tryptone, 5 g yeast extract, 10 g NaCl and 15 g bacteriological agar dissolved in distilled water made up to 1 litre, pH 7.0) containing ampicillin (50 µg/mL), 5-bromo-4-chloro-3-indolyl-β-D-galactopyranoside (50 µg/mL) and isopropyl β-D-1-thiogalactopyranoside (1 mM) for blue-white colony selection. White colonies were picked as positive and streaked onto LB agar plates containing ampicillin (50 µg/mL). Colony PCR was performed to test whether the transformed cells contained a plasmid with the expected insert size. For colony PCR, a colony from a streaked LB-ampicillin plate was used as DNA template and transferred to a PCR master mix ((*Taq* PCR Master Mix, Qiagen) containing all essential PCR components except primers and DNA template) using a sterile toothpick. GEM2987F and TOP168R primers were used to amplify the cloned fragment (Supplementary Table 19). PCR products were then checked by agarose gel electrophoresis for quantity and size. Clones with the expected insert size were grown in LB medium containing 50 µg/mL of ampicillin. These clones were then stored at −80 °C in LB medium supplemented with sterile glycerol at final concentration of 50% (v/v) for future use.

Colony PCR products of the expected size were sequenced using the universal bacterial primer 514R (Supplementary Table 19) to determine their origins. Full-length sequences of some cloned inserts were obtained using region-specific universal bacterial primers (514R, 518F, 800R, 968F and 1100R; see Supplementary Table 19) targeting different locations of 16S rRNA gene in separate reactions.

All clone sequencing was carried out either at the Massey Genome Sequencing Service (Massey University, Palmerston North, New Zealand) or at Macrogen Inc. (Seoul, Republic of Korea), using BigDye™ Terminator v3.1 cycle sequencing chemistry (Applied Biosystems, Foster City, CA, USA). DNA sequences were processed by trimming vector sequences and poor-quality sequences (with ambiguous electropherogram base calls from the 5′ and 3′ ends) using Geneious 8.1 software (Biomatters Ltd., Auckland, New Zealand). Reads from each clone were assembled to generate almost full-length 16S rRNA gene sequences.

Resulting sequences were used to query the SILVA version 123 bacterial 16S rRNA gene database, which contains a refined taxonomy of rumen bacteria^21^, using the BLAST sequence similarity algorithm in QIIME^39^ to find their closest relative.

### Phylogenetic analysis

Full-length 16S rRNA gene sequences were checked for chimeras using Bellerophon^40^ and UCHIME^41^, and the fractional treeing method^42^. In the fractional treeing method, 450 bp of sequence from each end (5´ and 3´) of the 16S rRNA gene was used to generate two trees, one using sequences from the 5´ end and another using 3´ end sequences. The sequences with conserved positions in both trees were taken as non-chimeric sequences. These full-length clone library sequences together with pre-existing non-chimeric sequences of *Quinella* were used for taxonomic refinement.

Newly generated long-length sequences were aligned to entries in the SILVA 123 bacterial 16S rRNA gene database^21^ using the SINA aligner^43^ and then imported into ARB^42^. A phylogenetic tree was generated in ARB using the Jukes-Cantor genetic distance model^44^ with the Neighbor-Joining method^45^. Additionally, a maximum likelihood phylogeny was implemented in RAxML version 8^46^ using the GTRGAMMA nucleotide substitution model with rapid bootstrap analysis, to confirm the position of each sequence in the tree. Trees were rooted using the 16S rRNA gene sequence of *Fibrobacter succinogenes* (FibSuc43, GenBank accession CP002158 FSU_1948 (rrsB)). The resulting RAxML tree was imported into ARB^42^. Clusters of sequences that generally had bootstrap support >70% were identified and defined at species and genus levels based on average sequence identities within each cluster (>97% for species; >93% for genera). The new long-length 16S rRNA gene sequences used in the tree were deposited in the National Centre for Biotechnology Information (NCBI) database with GenBank accession numbers MF184869 to MF184922 (PopSet 1199303303).

Short sequences of interest were aligned to the whole database using the SINA aligner and imported into ARB^42^ using the ARB parsimony (quick add mark tool) insertion function, using the phylogenetic tree with long-length *Quinella* 16S rRNA gene sequences as the reference.

### Cultivation

We used the methods and medium described by Kenters et al.^22^ to try to culture *Quinella* spp. RM02 medium containing 2GenRFV plus (final concentrations) 20 mM mannitol, 10 mM salicin and 0.08% (w/v) pectin was prepared in Hungate tubes (Bellco, Vineland, NJ, USA). Rumen content samples from 12 sheep shown to have high concentration of *Quinella*-like cells were diluted in growth medium so that each tube received an estimated 10 or 40 bacterial cells, based on an approximation of 10^9^ bacterial cells per mL of rumen contents^22^. Tubes that showed any turbidity were examined microscopically and sub-cultured into fresh media. Tubes that were identified as having large cells were used for spread-plating onto the same medium solidified with 15 g bacteriological agar per L. These procedures were carried out in an anaerobic chamber (Coy Laboratory Products Inc., Grass Lake, MI, USA), containing an atmosphere of 92% carbon dioxide and 8% hydrogen (industrial grade quality; BOC Gas, Auckland, New Zealand). The plates were incubated in 2.5 L AnaeroJars with rack plate canisters (Oxoid, Hampshire, UK) containing resazurin indicator strips (Oxoid). Agar plates were checked every 24 h for colonies. Colonies were examined microscopically, and those with larger cells were used for fluorescence in situ hybridisation (see below) and 16S rRNA gene sequence-based identification (see above) to identify *Quinella*.

### Fluorescence in situ hybridisation (FISH)

The *Quinella*-specific FISH probe Quin1231 (5′-TTCAGCCCATTGTAGTAC) was designed based on long length 16S rRNA gene sequences to target the 16S rRNA of *Quinella* at *Escherichia coli* positions 1231-1248. Its theoretical specificity was assessed using the ARB probe match tool^42^ and it was labelled with Alexa 488. The bacteria-specific domain-level probe EUB338 (5´-GCTGCCTCCCGTAGGAGT; target site *E. coli* position 338–355, labelled with Cy3), and a nonsense probe nonEUB338 (the reverse complement of EUB338, labelled with Cy5) were used as positive and negative controls, respectively. Probes were synthesised by IDT (Custom Science, Auckland, New Zealand). Before use, probes were re-suspended in sterile nuclease-free water to make a working concentration of 50 ng/μL.

These probes were applied to paraformaldehyde (PFA)-fixed rumen fluid, enriched cells, or culture samples. The samples to be probed were fixed in 4% PFA (w/v) solution:sample (1:3) and kept at 4 °C for 2 h. Samples were then centrifuged at 8000 × *g* for 5 min. Supernatants were discarded and the pellets were washed twice in PBS buffer by repeating the centrifugation step. Finally, cell pellets were diluted in 750 µl of PBS and pure ethanol (1:1) and stored at −20 °C. Probe stringencies (conditions at which probes specifically hybridised only to *Quinella*-like cells) were optimised by varying formamide concentration, NaCl concentration and hybridisation temperature. The rest of the procedure was as described by Hugenholtz et al.^47^ with slight modifications. Briefly, 3–5 μL (depending upon cell concentration) samples of fixed cells were applied on 10-well FISH slides and air dried for at least 3 h or overnight. Slides were then dehydrated using a series of ethanol washes starting at 50%, 80% and finally 100% ethanol for 3 min each and finally air dried. A hybridisation oven was pre-warmed to the desired hybridisation temperature, e.g., 46 °C, 50 °C or 52 °C. Hybridisation buffers (8 μL; Supplementary Table 20) with various formamide and NaCl concentrations were applied to different slides in duplicate. Probes (1 μL/well of 50 ng/μL) were applied either singly or in combination to different wells and mixed gently with a micropipette tip without touching the well surface. Each slide was then transferred carefully into a 50-mL Falcon tube containing moistened paper towels, capped firmly and placed horizontally in the hybridisation oven for 2 h. After hybridisation, slides were rinsed well with the same pre-warmed wash buffer (Supplementary Table 19) to remove unhybridised probes. Slides were then transferred into 50-mL tubes containing wash buffer (Supplementary Table 19) and placed in a waterbath (at a temperature 2 °C higher than the hybridisation temperature) for 15 min. Slides were then rinsed briefly with ice-cold distilled water and dried immediately either with compressed air or in a 39 °C oven. Antifade mounting solution (VECTASHIELD; Vector Laboratories Ltd., Burlingame, CA, USA) was applied carefully on the slides and covered with a coverslip without trapping air bubbles in the wells. Slides were then visualised by phase contrast and epifluorescence microscopy (DM2500, Leica Microsystems, Wetzlar, Germany) using filters appropriate for each probe’s fluorophore, and images captured using Leica Application Suite V2.2 (Leica Microsystems Cambridge, Cambridge, UK). Cell sizes were estimated from captured images using Paint version 21H2 (Microsoft Corporation, WA, USA), with scaling calibrated using parallel images of a stage micrometer (Olympus Corporation, Tokyo, Japan) made using the same magnification.

### Electron microscopy

Scanning electron microscopy (SEM) and transmission electron microscopy (TEM) was performed at the Manawatu Microscopy and Imaging Centre, Massey University, Palmerston North, New Zealand. SEM was performed on an aliquot of sample 3 (see section on *Quinella*-enriched samples, above) fixed to a Formvar grid (Sigma-Aldrich, St. Louis, MO, USA), stained with 2% (w/v) uranyl acetate and examined with a FEI Quanta 200 scanning electron microscope (Philips Electron Optics, Eindhoven, The Netherlands). Part of sample 3 was prepared for TEM by washing the cell pellet three times in sterile water, resuspending in modified Karnovsky’s fixative (2% [w/v] paraformaldehyde and 3% [w/v] glutaraldehyde in 0.1 M sodium phosphate buffer, pH 7.2) for embedding in resin (Procure 812; ProSciTech, Qld, Australia), and thin sections made using an EM UC7 ultra-microtome (Leica Microsystems, Wetzlar, Germany). TEM used a Tecnai G2 Biotwin transmission electron microscope (FEI, Hillsboro, OR, USA). Electron microscopy used XT Microscope Control and TUI version 4.5 software (FEI).

### *Quinella* diversity, abundance and metagenome sequencing

DNA extracted from the three samples containing enriched *Quinella* cells was used to construct bar-coded genomic shotgun libraries and sequenced using a single lane of Illumina MiSeq version 3 generating 2 × 300 bp paired-end reads. Library preparation and sequencing was carried out by Eurofins Genomics (Ebersberg, Germany). 16S rRNA genes and flanking regions were also amplified from this DNA using primers described in Supplementary Table 19, and cloned and sequenced as described above.

### Quality control, filtering, assembly and binning

The sequences were first checked for quality using FastQC^48^, then poor quality reads were trimmed using Trimmomatic^49^, both using default parameters. The resulting sequencing reads were assembled using SPAdes Genome Assembler^50^. The Quality Assessment Tool for Genome Assemblies (QUAST) was used to evaluate the genome assemblies (% G + C content, number of contigs, contig length, N50 and N75). The assembled reads in contigs were binned into potential genome bins using MetaBAT^16^, a tool that uses integrated empirical probabilistic distances of genome abundance and tetranucleotide frequency to bin contigs. To run MetaBAT, an index file was first generated for each assembly which was later used for contig binning.

To understand the initial taxonomic composition of genome bins, AMPHORA2 software^51^, which uses a set of 31 bacterial and 104 archaeal protein-coding marker genes, was used. The genome bins generated by MetaBAT were assessed for quality (completeness and contamination) using CheckM^15^.

### *Quinella* genome bin annotation and analysis

Genome annotation of the four selected *Quinella* genome bins was performed using the GAMOLA2 annotation tool^52^ in combination with the Artemis software suite^53^. Genes were predicted using Prodigal^54^. To understand the similarity between genome bins, functional genome distribution (FGD) analysis was conducted. This analysis does not represent the phylogenetic distance. Instead, overall genome bin similarities are calculated using ORFeome amino-acid sequences based on the assumption that highly similar proteins are indicative of functional similarities. The similarities and absence of predicted proteins are combined into a pairwise FGD dissimilarity matrix^28^. This was then used to generate a tree by an unweighted pair group method with arithmetic mean (UPGMA) method in MEGA 7^55^. Additionally, genomes were classified in the Genome Taxonomy Database (GTDB Release 207) using GTDB-Tk based on average nucleotide identity values across multiple gene loci^26,27^. OrthoMCL analysis^56^ was conducted to identify the orthologous gene families present across all the genome bins.

The Kyoto Encyclopedia of Genes and Genomes (KEGG)^57^ and MetaCyc^58^ were used as reference databases to construct metabolic pathways and flagellar assembly. Prediction of type III secretion systems used EffectiveT3 and EffectiveS346 in EffectiveDB^29^. Key genes involved in metabolic pathways were compared with their experimentally validated homologues using the BLOSUM62 (BLOcks SUbstitution Matrix) sequence alignment option within Geneious^59^. CAZymes (carbohydrate active enzymes in CAZyDB^60^) and transporters were searched for in *Quinella* genome bins using dbCAN 3.0^61^ and TransportDB 2.0^62^, respectively.

Genes that were identified as playing major roles in metabolism were translated and the deduced amino-acid sequences were manually checked and compared to reference proteins using different databases: NCBI’s non-redundant protein database^63^, clusters of orthologous groups (COG) database^64^, Pfam^65^ and TIGRFAM^66^ databases, and reviewed sequences from the UniProtKB/Swiss-Prot database^67^. Deduced amino-acid sequences were aligned using MUSCLE and Clustal W with default parameters in Geneious. Membrane protein topologies were predicted using Phobius and SPOCTOPUS algorithms implemented in TOPCONS^68^. Trees of sequences based on amino-acid sequence similarity were generated using Geneious’s Tree Builder based on Jukes-Cantor distances and Neighbor-Joining consensus tree protein alignment. Hydrogenase sequences were aligned to the database described by Greening et al.^69^.

### Classification of hydrogenases in transcriptome data

Metatranscriptome data^33,34^ (GenBank PRJNA202380) were downloaded from the NCBI repository and written into paired forward and reverse read files. The reads in these paired files were joined by overlapping them using Flash2^70^, and the resulting extended read files were subsampled to keep only 10 million reads per sample using seqtk (https://github.com/lh3/seqtk) with parameters sample -s100 query.fastq 10000000. These subsampled files were compared to the HydDB hydrogenase database^69^, to which were added five new *Quinella* hydrogenase sequences, using the program Diamond^71^, keeping only one match per query and only those with identity >65% and an alignment length >40 aa. These results were used to count the number of hits matching each of the hydrogenase class and those matching hydrogenases Group 1d from the order *Selenomonadales*.

### Reporting summary

Further information on research design is available in the Nature Research Reporting Summary linked to this article.

## Supplementary information

Supplementary Information

Description of Additional Supplementary Files

Supplementary Data 1

Supplementary Data 2

Reporting Summary

## Data Availability

The reconstructed *Quinella* genomes and the raw sequence data are deposited in GenBank under BioProject PRJNA373898. New long length 16S rRNA gene sequences are deposited in GenBank under accessions MF184869 to MF184922 in PopSet 1199303303. Shorter 16S rRNA gene sequences amplified from concentrated *Quinella* cell suspensions are deposited in GenBank under accessions OM320214 to OM320357 (see Supplementary Data 2 for hyperlinks). 16S rRNA genes with flanking regions amplified from the DNA preparations used to generate the reconstructed genomes are deposited in GenBank under accessions OM303038 to OM303057 (see Supplementary Data 2 for hyperlinks). Previously published metatranscriptome data were from GenBank BioProject PRJNA202380. Previously published 16S rRNA gene sequence data from sheep were from GenBank BioProject PRJEB4486.

## Code availability

The code for Functional Genome Distribution^28^ (FGD) is available from Eric Altermann (e.altermann@massey.ac.nz).
