# Supplementary material for: Genomic insights into the physiology of Quinella, an iconic uncultured rumen bacterium
Source: Nat Commun. 2022 Oct 20;13:6240. doi: 10.1038/s41467-022-34013-1 (PMC9585023; doi:10.1038/s41467-022-34013-1)
Supplement: Supplementary file 1 — Supplementary Information [file 41467_2022_34013_MOESM1_ESM.pdf]

Supplementary Information

Genomic insights into the physiology of *Quinella*, an iconic uncultured rumen bacterium

Sandeep Kumar, Eric Altermann, Sinead C Leahy, Ruy Jauregui, Arjan Jonker, Gemma Henderson, Sandra Kittelmann, Graeme T. Attwood, Janine Kamke, Sinéad M. Waters, Mark L. Patchett, and Peter H. Janssen

Contents

Supplementary Note 1. Calculating cell sizes 6

Supplementary Note 2. *Quinella* diversity in sheep rumens 7

Supplementary Note 3. FISH probe 8

Supplementary Note 4. Quality assessment of the *Quinella* genome bins 9

Supplementary Note 5. Potential genome sizes 10

Supplementary Note 6. G+C contents 11

Supplementary Note 7. Analysis of 16S rRNA genes amplified from genome bins 12

Supplementary Note 8. Comparison of 16S rRNA genes from genome bins to sequences in sheep rumens 14

Supplementary Note 9. Degradation of polysaccharides 15

Supplementary Note 10. Degradation of sugars to pyruvate 17

Supplementary Note 11. Lactate dehydrogenase 19

Supplementary Note 12. Acetate formation 20

Supplementary Note 13. Formate, butyrate or ethanol formation 23

Supplementary Note 14. MMCD and OACD 24

Supplementary Note 15. Fumarate reductase 30

Supplementary Note 16. Detailed analysis of *Quinella* QFR 31

Supplementary Note 17. ATP synthase 33

Supplementary Note 18. Na^+/H^+ antiporter 34

Supplementary Note 19. Hydrogenase (Ni-Fe) 35

Supplementary Note 20. Rnf complex 39

Supplementary Figure 1. Sequence identities between 16S rRNA gene sequences from *Quinella* .. 40

Supplementary Figure 2. Phylogenetic distribution of repset sequences within the radiation of *Quinella* 41

Supplementary Figure 3. Phylogenetic tree of 16S rRNA gene sequences from concentrated samples enriched for *Quinella* cells and affiliated with *Quinella* 43

Supplementary Figure 4. Phylotyping of genomic bins........44

Supplementary Figure 5. Comparison of *Quinella* genome bins........45

Supplementary Figure 6. Primer targets used to amplify 16S rRNA genes from DNA of *Quinella*-enriched samples........46

Supplementary Figure 7. Phylogenetic tree of *Quinella* 16S rRNA gene sequences........47

Supplementary Figure 8. Sequence similarity matrix of clone library sequences amplified from the DNA samples used to generate the genome bins........49

Supplementary Figure 9. Phylogenetic distribution of repset sequences representing abundant OTUs of *Quinella*........51

Supplementary Figure 10. Venn diagram of orthologous protein families among all four *Quinella* genome bins........52

Supplementary Figure 11. Functional genome distribution (FGD) tree of *Quinella* genome bins ...53

Supplementary Figure 12. Flagellar assemblies inferred by genes detected in *Quinella* genome bins........54

Supplementary Figure 13. Deduced fermentation pathways of *Quinella*........55

Supplementary Figure 14. Phylogenetic analysis of L-lactate dehydrogenases (LDH) from the *Quinella* genome bins........56

Supplementary Figure 15. Possible end product formation by *Quinella* from glucose and lactate utilisation........57

Supplementary Figure 16. Identification of PFOR in the *Quinella* genome bins........58

Supplementary Figure 17. Identification of succinate CoA-transferase in the *Quinella* genome bins........59

Supplementary Figure 18. Possible acetate formation pathways from acetyl-CoA........60

Supplementary Figure 19. Enzymes involved in formate, butyrate and ethanol pathways........61

Supplementary Figure 20. Pathway showing possible electron flow from the glycolytic pathway and conversion of pyruvate to acetyl-CoA........62

Supplementary Figure 21. Phylogenetic tree of α subunits of MMCD (MmdA) and OACD (OadA), based on amino acid sequences........63

Supplementary Figure 22. Alignment of alpha subunits of MMCD (MmdA) and OACD (OadA) of *Quinella* with reference sequences 64

Supplementary Figure 23. Beta subunits of MMCD and OACD 66

Supplementary Figure 24. Gamma and delta subunits of MMCD and OACD 67

Supplementary Figure 25. MMDC and OADC structures 68

Supplementary Figure 26. Alignments of amino acid sequences of hydrophilic subunits of fumarate reductase from *Quinella* 69

Supplementary Figure 27. Subunit C of fumarate reductase 70

Supplementary Figure 28. Alignment of amino acid sequences from subunit c of ATP synthase 71

Supplementary Figure 29. Large subunit of the hydrogenase of *Quinella* 72

Supplementary Figure 30. Phylogenetic classification of *Quinella* hydrogenases 73

Supplementary Figure 31. Small subunit of the hydrogenase of *Quinella* 74

Supplementary Figure 32. Cytochrome-b subunit of the *Quinella* hydrogenase 75

Supplementary Table 1. Relative abundance of *Quinella* spp. in rumen and forestomach samples from the Global Rumen Census 76

Supplementary Table 2. Long-length 16S rRNA gene sequences of *Quinella* spp. obtained from rumen samples 77

Supplementary Table 3. Alignment of probe Quin1231 (*Escherichia coli* positions 1231-1248) to matching region of *Quinella* 16S rRNA gene sequences 78

Supplementary Table 4. Read number and sequence quality from DNA sequencing data 80

Supplementary Table 5. Assemblies and bins generated from metagenomic DNA sequence data ... 81

Supplementary Table 6. Steps to generate *Quinella* genome bins from metagenomic DNA sequence data 82

Supplementary Table 7. Lineage-specific quality control assessment of *Quinella* genome bins 83

Supplementary Table 8. Primers used to amplify 16S rRNA genes from DNA of *Quinella*-enriched samples 84

Supplementary Table 9. Similarity matrix of cloned sequences 85

Supplementary Table 10. *Quinella* genome bin statistics 86

Supplementary Table 11. Functional classification of the predicted genes in the four *Quinella* genome bins 87

Supplementary Table 12. CAZyme counts in the *Quinella* genome bins 89

Supplementary Table 13. CAZymes found in *Quinella* genome bins 90

Supplementary Table 14. Key enzymes in sugar fermentation and associated energetics found in *Quinella* genome bins 99

Supplementary Table 15. GH enzyme family and related enzymes found in all *Quinella* genome bins 105

Supplementary Table 16. Phosphotransferase system (PTS) transporter components found in *Quinella* genome bins 106

Supplementary Table 17. Amino acid sequence similarities of fumarate reductase subunit C of *Quinella* genome bins 109

Supplementary Table 18. Steps and enzymes involved in ATP formation and consumption 110

Supplementary Table 19. Primers used for amplification and sequencing of DNA and plasmid fragments 112

Supplementary Table 20. Hybridisation and washing buffer preparation for FISH 113

Supplementary References 114

Supplementary Note 1. Calculating cell sizes

Quinella cells were assumed to be prolate spheroids, 8 μm long and 4 μm in diameter[1], with a volume of 67.02 μm^3. *Escherichia coli* cells were assumed to be cylinders with hemispherical caps, and 3 μm long and 0.7 μm in diameter for glucose-grown cells[2], with a volume of 1.06 μm^3.

The volume (V) of a prolate spheroid[3] was calculated from

$$V = {}^{4}/_{3}\pi(L/2)r^2$$

and the volume (V) of a cylinder with hemispherical caps[2] from

$$V = \pi r^2(L - {}^{2}/_{3}r)$$

where r is the cell radius at the widest point perpendicular to the longest axis (i.e., half the diameter), and L is the length along the longest axis.

Supplementary Note 2. *Quinella* diversity in sheep rumens

The refined phylogenetic tree of *Quinella* spp. (Figure 1) was used as a reference to compare the distribution and abundance of these species in 236 sheep rumen samples based on short reads of 16S rRNA genes (approx. 400 bp) that had previously generated from 118 sheep by Kittelmann et al.[4]. These short-read sequences were clustered at 97% sequence similarity using QIIME[5] and the resultant repset sequences were assigned to genera using the SILVA 123 16S rRNA gene database updated with rumen bacteria described by Henderson et al.[6] as a BLAST database. The repset sequences affiliated to *Quinella* and with >10 sequences were extracted and then placed in the refined phylogenetic tree of long-length sequences using the parsimony (quick add marked tool) insertion function in ARB[7] (Supplementary Figure 2). Sequences from this set of analysed rumen samples grouped into most of the possible *Quinella* species shown in Figure 1, except *Quinella* candidate species 5 and *Quinella* candidate species 7. The clusters with the largest number of reads assigned to them were *Q. ovalis* and *Quinella* candidate species 6. Some of the repset sequences also grouped separately from the earlier-defined species but formed coherent clusters with existing singleton long (>1443 nt) sequences (Supplementary Figure 2). Six clusters of short reads in the genus *Quinella*, two in *Selenomonadaceae* candidate genus 1 and one cluster adjacent to *Selenomonadaceae* candidate genus 1 did not contain any long-length *Quinella* sequences but all contained ≥3 repset sequences that represented OTUs with 71 to 3176 pyrosequencing reads (Supplementary Figure 2). These findings suggest that more *Quinella* species may exist for which no long-length 16S rRNA gene sequences were obtained in this study. However, this should also be interpreted with caution, as errors in pyrosequencing can lead to over estimation of diversity[8, 9], and placing these short sequences into the tree of previously-defined species is not robust because of their limited lengths.

Supplementary Note 3. FISH probe

A *Quinella*-specific FISH probe has not been reported before, based on searches in NCBI, ProbeBase[10] and published literature. The *Quinella*-targeted FISH probe Quin1231 was designed based on long length 16S rRNA gene sequences from the newly refined *Quinella* phylogenetic tree (Figure 1). Probe Quin1231 targeted the 16S rRNA of *Quinella* at *Escherichia coli* positions 1231-1248 and matched exactly to 43 of the available 44 full-length *Quinella* sequences (Supplementary Table 4). The ARB probe match tool[7] predicted Quin1231 to be specific to *Quinella* 16S rRNAs, assuming no probe-binding mismatches.

Supplementary Note 4. Quality assessment of the *Quinella* genome bins

Three (SR1Q5, SR1Q7 and SR2Q5) of the final four *Quinella* genome bins were assigned to the phylum *Firmicutes* based on 295 lineage-specific marker genes (present in >97% of particular lineage) arranged in 158 marker sets. The fourth genome bin (SR3Q1) was only assigned to the domain Bacteria (it contained 104 lineage-specific marker genes to separate it from other domains). Markers genes present at more than one copy per bin are indicative of contamination while the absence of these genes contributes towards incompleteness[11]. Contamination in the *Quinella* genome bins ranged from estimates of 0.21 to 10.3% (Table 2, Supplementary Table 7). Of 295 *Firmicutes* lineage-specific marker genes, 18 and 33 were found in duplicate copies in bins SR1Q5 and SR1Q7 respectively. Bin SR3Q1 contained 10 genes in duplicate, six in three and four copies each, while one gene was present in five or more copies. As a result, this bin was estimated to be 10.25% contaminated. Because only one duplicate marker gene was found in bin SR2Q5, this bin was considered the least contaminated (0.21%) *Quinella* genome bin. Furthermore, of 295 lineage-specific marker genes, 30, 18 and 17 were absent from genome bins SR1Q5, SR1Q7 and SR2Q5 respectively, indicative of genome incompleteness. Notably, in genome bin SR3Q1, six out of 104 bacteria domain level marker genes were absent, suggesting that this bin should be treated with care when predicting the overall *Quinella* physiology. The contamination level could have been further reduced by excluding contigs with duplicated marker genes, but it was unclear which of the duplicates originated from *Quinella*, as they appear to have similar coverage, G+C contents (Supplementary Figure 5) and gene arrangements.

The contigs with very different G+C content and coverage were investigated in more detail, but none of these contigs appeared to have multiple copy marker genes and hence had no role in bin contamination and completeness values. It was interesting to find no strain heterogeneity in bin SR2Q5, suggesting that it represented a single strain. In contrast, the other three bins (SR1Q5, SR1Q7 and SR3Q1) were mixed (≥48.5% contamination) and likely contained multi-strain genomic information.

Supplementary Note 5. Potential genome sizes

The differences in genome bins size may also reflect differences in the level of contamination. For example, genome bin SR3Q1 (2.6 Mbp) was the largest bin, but was estimated to contain 10.2 % contamination, while bin SR2Q5 which contained almost no contamination was the smallest (1.8 Mbp). Genome sizes of other members of the family *Selenomonadaceae* (to which *Quinella* belongs) vary from 1.26 Mbp (*Dialister micraerophilus*) to 5.28 Mbp (*Pelosinus fermentans*)[12]. Significant variations in genome sizes have been observed previously within a genus. For example, in the genus *Selenomonas*, which is the closest genus to *Quinella*, has 10 species currently identified[13] with genome sizes varying from 2.3 Mbp (*Selenomonas* sp. F0592; GenBank accession NZ_CP012071) to 3.0 Mbp (*Selenomonas ruminantium* subsp. *lactilytica*[14]).

Supplementary Note 6. G+C contents

The lowest G+C content was found in SR3Q1 (49.2 mol%) and the highest was in SR2Q5 (56.0 mol%). This pattern was similar to that found in the genus *Selenomonas*, where G+C content varies from 50.7 mol% (*Selenomonas ruminantium* subsp. *lactilytica*[14]) to 57.1 mol% (*Selenomonas* sp. F0592; GenBank accession NZ_CP012071). Therefore, the variation in the G+C content of genomic DNA is not unusual. However, genome completeness, contamination and strain heterogeneity may also play a major role in the observed G+C content. Missing regions or contaminated regions as well as mixed genomes at strain level might also affect the overall G+C content of genome bins.

Supplementary Note 7. Analysis of 16S rRNA genes amplified from genome bins

The cloned sequences amplified using the same forward primer were highly similar but not identical over the full length of the cloned fragments that included the 16SrRNA gene plus flanking regions (Supplementary Table 9, upper triangle of matrix). The greatest similarity was found in between clones targeting bin SR1Q5 (≥98.9%) followed by clones of bin 2Q5 (≥98.2%) and then clones from bin SR3Q1 (≥98.4%) whereas the lowest level of similarity occurred for clones targeting bin SR1Q7 (≥91.0%). The sequence similarities between only the 16S rRNA genes in clones amplified using the same forward primer was >99% (Supplementary Table 9, lower triangle of matrix). The similarities between cloned sequences amplified using different forward primers was lower (≤82.6% over the full cloned sequences, ≤95.7% for the 16S rRNA gene regions of the clones). The clones generated with a single primer set contained the same gene upstream of the 16S rRNA gene, but that gene was different between clones generated using different primer sets (schematic in Supplementary Figure 6). This indicates that the 16S rRNA genes amplified using the different primer sets were from potentially different *Quinella* species, although they could have been some different ribosomal RNA operons. The variations within any one clone library could have been due to amplification of DNA from closely related strains within the same DNA sample, operons within any one strain, or amplification errors.

Next, the clones were compared with the contigs in the four genome bins that contained partial 16S rRNA genes. When the clone regions overlapping with the reference contigs sequences from the four bins were aligned, all four clone libraries contained at least one clone that showed ≥99.6% similarity over the partial 16S rRNA gene and the adjacent gene region targeted by the primer set. These best matches were for all five clones targeting bin SR1Q5 (100%), clone SR1Q7_49 (99.1%) targeting bin SR1Q7, clone SR2Q5_87 (99.8%) targeting bin SR2Q5 and clone SR3Q1_120 (99.9%) targeting bin SR3Q1. When this analysis was restricted to just the 16S rRNA regions in the 20 clones, all clones had ≥99.0% similarity (SR1Q5, 100%; SR1Q7, ≥99.0%; SR2Q5, ≥99.2%; and SR3Q1, ≥99.2%) and at least one clone in each library had 99.8% to 100% similarity to the partial 16S rRNA genes in the bins that were targeted by that primer set (Supplementary Table 9). This suggests that some of the nearly full length 16S rRNA genes in the clone libraries were from the same strains or very close relatives of the strains that contributed the contigs containing the 16S rRNA genes in each of the four bins.

The analysis was repeated limiting it to only the parts of the cloned sequences that matched the bin contig sequences outside the 16S rRNA gene. Clones assigned to bin SR1Q5 all had 100%

similarity with the references contig. In the other clones, the similarities were ≥85.2% with the respective contigs from the bins they were assigned to. This showed that the differences between the clones and the contigs from the bins they were targeting were mainly in these regions adjacent to the 16S rRNA genes. Translating these regions showed that this also reflected amino acid level differences, suggesting that the clones might represent different strains of same *Quinella* species. Overall, it seems that the cloned 16S rRNA genes are representative of the bins they were assigned to. This variation in the flanking regions of the cloned sequences showed that each of samples 1 to 3 contained different but closely related strains that shared very similar 16S rRNA genes. However, this doesn't allow conclusions to be made about the sequence data in the bins. If the starting samples contained very closely related strains, it is likely that the bins represent aggregates of genomes from closely related strains.

Supplementary Note 8. Comparison of 16S rRNA genes from genome bins to sequences in sheep rumens

Repset sequences from the study of Kittelmann et al.[4] representing the operational taxonomic units (OTUs) that contained ≥100 sequencing reads were added to the tree of high quality *Quinella* 16S rRNA gene sequences plus the 16S rRNA gene sequences from the genome bins (Supplementary Figure 7), using the ARB parsimony (quick add mark tooled) insertion function[7] to generate a tree containing all of these sequences (Supplementary Figure 9). Some of the highly abundant OTUs from the study of Kittelmann et al.[4] clustered with the 16S rRNA gene sequences representing genomic bins SR1Q5 and SR1Q7, whereas the 16S rRNA gene sequences representing genome bins SR2Q5 and SR3Q1 did not cluster with any of the repset sequences. This is not surprising, because these genome bins came from samples from a different group of sheep than the one used to generate the repset sequences.

Supplementary Note 9. Degradation of polysaccharides

The genes in the four *Quinella* genome bins genes were compared with different databases (NCBI non-redundant protein sequences database, Pfam database, TIGRFAMs database) using BLAST in GAMOLA2[15] as the search tool to identify their ability to attack plant polysaccharides. A separate carbohydrate-active enzymes (CAZyme) search was conducted using the CAZyDB database[16], which contains a curated reference set of carbohydrate degrading enzymes. A summary of the CAZyme analysis of the four *Quinella* genome bins is presented in Supplementary Table 12 and details can be found in Supplementary Table 13.

Enzymes in GH families 1, 3, 13, 23, 77 and 84 were present in all genome bins. Details of these enzymes are listed in Supplementary Table 15. Signal peptide and transmembrane topology analysis using Phobius[17] and SPOCTOPUS[18], implemented in TOPCONS[19], suggested that all of these enzymes were non-cytoplasmic. Among these enzymes, β-glucosidase, α-phosphotrehalase, transglycosylase and *O*-GlcNAcase ((protein)-3-*O*-(*N*-acetyl-D-glucosaminyl)-L-serine/threonine *N*-acetyl-glucosaminyl hydrolase) contained potential signal peptides, which suggested they are secreted and act extracellularly.

Glycosyl hydrolases (GH), a CAZyme class of enzymes that hydrolyse the glyosidic bonds between carbohydrates or between carbohydrate and non-carbohydrate components of polysaccharides, contains most of the enzymes that are involved in cellulose and hemicellulose degradation. Of those enzymes, endo-1,4-β-D-glucan hydrolase (EC 3.2.1.4), exo-1,4-β-D-glucan cellobiohydrolase (EC 3.2.1.91) and β-D-glucosidase (EC 3.2.1.21) are the key enzymes needed for cellulose hydrolysis[20]. None of the GH enzymes found in the *Quinella* genome bins appear to be endo-1,4-β-D-glucan hydrolases or exo-1,4-β-D-glucan cellobiohydrolases, but β-glucosidase was present in all genome bins. This enzyme cannot degrade cellulose alone, as it is involved in the third and last step of cellulose hydrolysis (conversion of cellobiose and cellodextrins to glucose). Thus, it appears that none of the *Quinella* genomes encode the primary enzymes for cellulose-degradation. However, the β-glucosidase may act on cellobiose and cellodextrins, released by other rumen microbes. Similarly, 1,4-β-D-xylan xylanohydrolase (EC 3.2.1.8) and 1,4-β-D-xylan xylohydrolase (EC3.2.1.37) are key enzymes involved in xylan degradation[21]. These enzymes fall in GH families 10 and 11. The amino acid sequences for these enzymes were also searched for in the four *Quinella* genome bins but none of these CAZyme families were found.

Acetyl-xylan esterase (EC 3.1.1.72) and feruloyl esterases (EC 3.1.1.73), which belong to carbohydrate esterases (CE) families 1 to 7 and hydrolyse ester-linked side groups of xylan[22], were absent from the *Quinella* genome bins. However, 8 to 19 CE enzymes were detected in *Quinella*

genomic bins. They belonged to CE family 10 (esterase, putative carboxylesterase and Tat pathway signal sequence), which are not involved in primary attacks on complex plant polysaccharides.

Gene sequences coding for pectin-degrading enzymes (pectin lyase, polygalacturonase and pectin methylesterases) which fall in CAZyme families PL8, PL16, GH28 and CE8 were also searched for in the *Quinella* genome bins but were not found.

Common starch-degrading enzymes are α-amylase, β-amylase, dextrin 6-α-glucanohydrolase, pullulanase and iso-amylase[23]. None of the *Quinella* genome bins contained genes sequences coding for any of these starch-degrading enzymes.

Quinella cells have been reported to contain storage polysaccharides[24]. Three of the enzymes found in all the genome bins may have a role in glycogen metabolism (1,4-α-glucan branching enzyme, α-phosphotrehalase and 4-α-glucanotransferase). The predicted extracellular location of these enzymes and the presence of a signal peptide in the α-phosphotrehalase makes the inference of their roles in intracellular glycogen metabolism speculative.

Almost one-third of the of the glycosyl transferases (GT) found in the *Quinella* genome bins were identified as poly-β-1,6 *N*-acetyl-D-glucosamine synthases, potentially involved in polymerisation of UDP-*N*-acetylglucosamine in bacterial cell wall synthesis. It was interesting to find lytic transglycosylase, a lysozyme type enzyme, which in some cases is reported to contribute to pathogenesis[25]. However, it is predicted that, together with transglycosylase, lytic transglycosylase may take part in the conversion of peptidoglycan to 1,6-anhydro sugars[26] and so these may play a role in cell wall metabolism. *O*-GlcNAcase was found in all *Quinella* genome bins but its function in bacteria is still unknown[27]. It too acts on *N*-acetylglucosamine, one of the two amino sugars in peptidoglycan. The *O*-GlcNAcase and transglycosylase had predicted extracellular locations (see above), which is expected for enzymes that act on the cell wall.

Overall, these analyses suggested that *Quinella* does not contain genes that encode enzymes responsible for the primary degradation of plant polysaccharides. Instead, it seems that *Quinella* relies on other microbes to hydrolyse the polysaccharide components of feed, and it appears able to compete effectively for the breakdown products of that primary attack for its own growth, as well as presumably using soluble sugars in the plant material.

Supplementary Note 10. Degradation of sugars to pyruvate

Each of the four *Quinella* genome bins contained all the genes coding for the enzymes for glucose fermentation to pyruvate (Supplementary Figure 13), with the exception of pyruvate kinase in bin SR2Q5 (Supplementary Table 14). Transporters were searched for using BLAST with the TransportDB 2.0 database[28]. Phosphotransferase systems (PTS) for glucose, sorbitol, fructose, maltose, mannose, galactitol, glucitol and ascorbate were detected (Supplementary Table 16). The phosphoenolpyruvate-dependent PTS system consists of the shared enzyme I (phosphoenolpyruvate-protein phosphotransferase, PstI) and phosphocarrier protein (Hpr), and the substrate-specific enzyme II[29]. These enzymes were present in all *Quinella* genome bins. Enzymes I and Hpr are listed in Supplementary Table 16. The substrate-specific enzymes II were present either as multi subunits proteins in operons or in fused forms (Supplementary Table 16). By using PTS, glucose, for example, would be imported and phosphorylated, and then converted to two pyruvate molecules via the standard glycolytic pathway, characterised by an ATP-dependent phosphofructokinase and a fructose bisphosphate aldolase. The gene coding for triosephosphate isomerase, a key enzyme in glycolysis, was present as a fused gene with phosphoglycerate kinase in all four *Quinella* genome bins. This type of fusion has been reported before in *Thermotoga maritima*[30]. *Quinella* genome bin SR2Q5 lacked the gene for pyruvate kinase that catalyses the conversion of phosphoenolpyruvate (PEP) to pyruvate[31], but pyruvate kinase was present in the other three *Quinella* genome bins (Supplementary Table 14). This *Quinella* strain may use alternative enzymes to make pyruvate. For example, in the fermentation of glucose to two pyruvate, it could use the glucose PTS to convert one molecule of PEP to one molecule of pyruvate and the other pyruvate may be formed by carboxylation of PEP to oxaloacetate followed by decarboxylation of oxaloacetate to pyruvate. However, it seems most likely that this gene was just missed from bin SR2Q5 at the assembly step. In the formation of two molecules of pyruvate from one molecule of glucose, one molecule of ATP will be used (fructose-6-phosphate to fructose-1,6-bisphosphate) and three ATP will be released (two at the step of 1,3-bisphospho glycerate to 3-phosophoglycerate and one at the step of phosphoenolpyruvate to pyruvate), resulting in the net gain of two ATP.

The *Quinella* genome bins were also searched for genes that code for enzymes involved in the pentose phosphate and Entner-Doudoroff pathways. These may act as parallel or alternative pathways to glycolysis[32, 33]. The *Quinella* genome bins contained three (ribose-5-phosphate isomerase, ribulose-5-phosphate 3-epimerase, transketolase) of four genes (transaldolase is only present in SR3Q1) that code for the enzymes in the non-oxidative phase of the pentose phosphate

pathway. As the genes for the key enzymes of the reversible oxidative phase (glucose-6-phosphate dehydrogenase, 6-phosphogluconolactonase and 6-phosphogluconate dehydrogenase) were missing, it doesn't seem possible for it to result in sugar transformation to pyruvate. However, the incomplete pathway can operate to make intermediate products required for a number of biosynthetic pathways (D-ribose 5-phosphate, D-ribulose 5-phosphate, D-xylulose 5-phosphate and D-erythrose 4-phosphate).

Genes coding for key enzymes (6-phosphogluconate dehydratase and 2-keto-3-deoxygluconate 6-phosphate aldolase) of the Entner-Doudoroff pathway[34] were missing in all *Quinella* genome bins, suggesting that this pathway was not present in *Quinella*.

Supplementary Note 11. Lactate dehydrogenase

BLAST analysis of the *Quinella* genome bins suggested that all of them contained genes that code for L-lactate dehydrogenase (L-LDH), which indicates that *Quinella* may either produce lactate or use it for growth or possibly both. There appears to be no way to differentiate between lactate producing and lactate using L-LDHs based on amino acid sequence motifs. However, a metagenomic and metatranscriptomic study[35] on *Sharpea*-enriched low-methane-yield sheep rumen microbiomes suggested that there may be 11 distinct *ldh* clusters from bacteria present in the rumen. The authors of that study predicted that the L-LDH from *Sharpea* spp. was associated with lactate formation, while L-LDH from *Megasphaera* spp. was associated with lactate utilisation. Furthermore, substrate utilisation experiments and measurement of fermentation end products conducted with *Sharpea* and *Kandleria* spp. showed that these organisms are lactate producers[36]. Sequences of L-LDH from *Megasphaera* spp. together with some other known lactate users (*Propionibacterium freudenreichii*, *Clostridium propionicum*, *Veillonella parvula* and *Desulfovibrio vulgaris*) and those from *Sharpea* and *Kandleria* spp. and other known lactate producers (*Lactobacillus acidophilus*, *Streptococcus equinus*, *Butyrivibrio fibrisolvens*, *Olsenella umbonata*, and *Ruminococcus gnavus*) were used for a phylogenetic analysis of L-LDH from the *Quinella* genomes bins. L-LDH from *Selenomonas ruminantium*, which is both a producer and user of lactate[37], was also included. All sequences were >300 amino acids long, and were considered to be nearly full length. A phylogenetic tree was constructed using the malate dehydrogenase sequence from *M. elsdenii* as an outgroup sequence (Supplementary Figure 14). The analysis showed that L-LDHs from lactate users and lactate producers did not group separately. LDH from the lactate producers *Sharpea*, *Kandleria* and *Olsenella* clustered together with 99% bootstrap support, whereas others formed separate groups (*B. fibrisolvens* and *R. gnavus* in one, and LDH1 and LDH2 of *L. acidophilus* with *S. equinus* in another). LDH from the lactate-using *V. parvula, C. propionicum* and *M. elsdenii* formed one cluster with 95.8% bootstrap support, but were separated from LDH from *D. vulgaris* and *P. freudenreichii*, which are also lactate users that formed separate branches in the tree. All *Quinella* LDH sequences were >93 % similar to each other and formed a stable group with LDH from *S. ruminantium*, with bootstrap support of 91.2%, perhaps suggesting that LDH in *Quinella* might also be involved in both lactate use and formation. However, experimental proof will be needed to confirm this. Orpin[38] observed the requirement for lactate as a sustrate while growing *Quinella* in mixed suspensions, but it was not clear whether the lactate was used by *Quinella* or other contaminating bacteria.

Supplementary Note 12. Acetate formation

The deduced activities of the proteins coded by genes in the genome bins suggested that *Quinella* may produce propionate as a major end product. However, to produce propionate from pyruvate requires four electrons. Two of those electrons can be gained from the conversion of 0.5 glucose to 1.0 pyruvate but to be able to get the other two electrons, *Quinella* would have to oxidize pyruvate to acetate or use external electrons, for example, from hydrogen produced by other microbes using a hydrogenase. The electrons are required in the malate dehydrogenase step (in the form of NADH) and the fumarate reductase step (in the form of reduced quinones). The possibility that *Quinella* can produce acetate will be discussed here. The most common way for anaerobes to produce acetate from pyruvate is to first convert pyruvate to acetyl-CoA using pyruvate:ferredoxin (or flavodoxin) oxidoreductase (PFOR) or a pyruvate:formate lyase.

All four *Quinella* genome bins contained genes that code for PFOR (Supplementary Table 14), that transfers electron to either ferredoxin or flavodoxin. The analysis was conducted to first confirm the presence of PFOR in all four *Quinella* genome bins and then to identify whether they are ferredoxin associated or flavodoxin associated. For the analysis, PFOR amino acid sequences of flavodoxin-type (nifJ) was represented by *Escherichia coli*, *Anabaena variabilis* and *Klebsiella pneumoniae* while *Desulfovibrio africanus* was used as a ferredoxin-type PFOR (Supplementary Figure 16). Amino acid sequences of *Selenomonas ruminantium* and *Megasphaera elsdenii* PFOR were also included in the analysis as they were identified as closest relatives in GAMOLA2 annotation of PFOR in the *Quinella* genome bins. On the basis of sequence similarity and phylogenetic analysis (Supplementary Figure 16), *Quinella* PFOR were most similar to NifJ type PFOR with greatest sequence similarity with *K. pneumoniae* (≥60.4%), and they also had ≤55.5% amino acid sequence similarity with *D. africanus*. Metal-binding cysteine residues[39] were also found conserved in all analysed sequences suggesting that both type of PFOR are very similar to each other. However, the presence of genes for NAD(P)H:flavin oxidoreductase in all four *Quinella* genomic bins (Supplementary Table 14), which would transfer electrons from the reduced flavodoxin to NADH, and the absence of any genes for enzymes that can transfer electrons from ferredoxin through to quinone, suggests that the *Quinella* PFOR is a flavodoxin-linked oxidoreductase. Nevertheless, it was surprising not to find any enzymes that transfer electrons from NADH to quinone or ubiquinone (Supplementary Figure 20). Usually NADH:quinone oxidoreductase catalyses this reaction, but genes for this were absent in all four *Quinella* genome bins.

From acetyl-CoA, there are four different routes for acetate formation (Supplementary Figure 18), three of which generate ATP[40, 41, 42]. All four *Quinella* genome bins were searched for the genes

involved in these pathways. None of the genome bins contained genes that code for phosphate acetyl transferase or acetate kinase, suggesting that the acetate-forming pathway characterised by these two enzymes (Supplementary Figure 18a) is not present in *Quinella*. Similarly, acetyl-CoA synthetase (Supplementary Figure 18b) was also absent in all four *Quinella* genome bins, which left two other options (Supplementary Figure 18c, d) for acetate formation by *Quinella*. All four *Quinella* genome bins contained DNA sequences that coded for putative acetyl-CoA hydrolases and succinyl-CoA synthetases, suggesting that *Quinella* could use pathways (c) or (d) shown in Supplementary Figure 18. The greatest identities of these two enzymes were with proteins coded by the *Selenomonas ruminantium* NBRC 103574 genome (>78% identity), annotated as an acetyl-CoA hydrolase and a succinyl-CoA synthetase. Acetyl-CoA hydrolase can convert acetyl-CoA to acetate by hydrolysis without ATP formation (Supplementary Figure 18c). Succinyl-CoA synthetase cannot be used for acetate formation unless succinate CoA-transferase is also present (Supplementary Figure 18d). Further analysis of the putative acetyl-CoA hydrolase from *Quinella*, using different databases used in the GAMOLA2 annotation pipeline, suggested that the protein annotated as an acetyl-CoA hydrolase in *S. ruminantium*, and by extension the homologue in *Quinella*, may actually be a succinate CoA-transferase. If that is the case, then *Quinella* would code for enzymes to convert acetyl-CoA to acetate and generate ATP through a succinate/succinyl-CoA cycle (Supplementary Figure 18d).

To better understand this possible acetyl-CoA hydrolase/succinate CoA-transferase, the deduced protein sequences were analysed in more detail. Mack and Buckel[42] conducted a study on the glutaconate CoA-transferase from *Acidaminococcus fermentans*. Glutaconate CoA-transferase catalyses the transfer of CoA from acetyl CoA to glutaconate. Mack and Buckel[42] showed that the change of glutamate (E54) in the active site to aspartate (D54) converted the glutaconate CoA-transferase to a glutaconate-CoA hydrolase. Additionally, this glutamate residue in the active site is essential to transfer the CoA moiety to the acceptor acid[43]. Using this clue, putative acetyl-CoA hydrolase sequences from the four *Quinella* genome bins were aligned with amino acid sequences of glutaconate CoA-transferase of *A. fermentans*, the putative acetyl-CoA hydrolase of *S. ruminantium*, the reviewed acetyl-CoA hydrolase of *Saccharomyces cerevisiae* and the reviewed succinate CoA-transferase of *Clostridium kluyveri*. From the alignments it was deduced that the *Quinella* genome bins contained a succinate CoA-transferase instead of an acetyl-CoA hydrolase. This was because the diagnostic glutamate (E54 in the *A. fermentans* glutaconate CoA-transferase, E432 in *Quinella*) was found to be conserved in all four *Quinella* genome bins, as found in known CoA-transferases[42] (Supplementary Figure 17). In contrast, an aspartate (D447 in *Saccharomyces cerevisiae*) was found in the homologous position in known acetyl-CoA hydrolases (Supplementary

Figure 17). Furthermore, two conserved motifs identified byTielens et al.[44] in CoA-transferases family 1 (the only family out of three that is involved in acetate formation[45]) was also found in all analysed CoA-transferases, suggesting that not only do all the *Quinella* genome bins contain CoA-transferase, but that the putative acetyl-CoA hydrolase of *S. ruminantium* may also be a succinate CoA-transferase. Tielens et al.[44] also divided CoA-transferases family 1 into three sub-families based on amino acid sequence homology. The CoA-transferases in the *Quinella* genome bins, and of *S. ruminantium*, *S. cerevisiae* and *Clostridium kluyveri*, belong to subfamily 1C on the basis of overall sequence homology and the sequence similarity in the conserved motif GxGGxD (Supplementary Figure 17).

Once the likely presence of succinate CoA-transferase in the *Quinella* genome bins was confirmed, it was logical to predict that *Quinella* may convert acetyl-CoA to acetate using succinate CoA-transferase to generate succinyl-CoA from succinate, then use succinyl-CoA synthetase to regenerate succinate with the formation of ATP from ADP (Supplementary Figure 18d). Succinyl-CoA synthetase is composed of two subunits (α and β), and works bidirectionally[46]. DNA sequences predicted to encode both of these subunits were found in all four *Quinella* genome bins except that the β subunit was absent from genome bin SR3Q1. In conclusion, on the basis of genes identified in the genome bins, it appears that *Quinella* catalyses a succinate-dependent conversion of acetyl-CoA to acetate using succinate CoA-transferase and succinyl-CoA synthetase. This pathway is present in parasitic helminths and protists[47, 48] and in the rumen fungus *Neocallimastix* sp. L2[49]. A biochemical study conducted on *S. ruminantium* to identify the key enzymes involved in fermentation of hexoses to different end products suggested that acetyl-CoA synthetase is involved in the conversion of acetyl-CoA to acetate[50]. This suggested that *S. ruminantium* uses pathways (a) or (b) as illustrated in Supplementary Figure 18. It will be interesting to investigate whether succinate CoA-transferase and succinyl-CoA synthetase are actually involved in acetate formation (pathway (d) in Supplementary Figure 18) in *S. ruminantium*.

Supplementary Note 13. Formate, butyrate or ethanol formation

All four *Quinella* genome bins were searched for genes that code for enzymes involved in formate, butyrate and ethanol formation (Supplementary Figure 19). Pyruvate dehydrogenase catalyses the conversion of pyruvate and CoA to acetyl-CoA and formate, but in the absence of oxygen this reaction is catalysed by pyruvate formate lyase[51, 52]. These enzymes were searched for in all four *Quinella* genome bins, but none were found. A gene (SR3Q1_647) coding for pyruvate formate lyase-activating enzyme (PFL-AE) was present in *Quinella* genome bin SR3Q1, but its absence in other bins suggested that it might be a part of contaminated sequence. Even if that is not the case, on its own it does not consitute a functional pyruvate formate lyase system. It was interesting to find the genes for all three subunits of formate dehydrogenase (an enzyme that catalyses the oxidation of formate to CO_2) in *Quinella* genome bin SR3Q1. Furthermore, a gene possibly coding for a formate dehydrogenase accessory protein that is required to assemble formate dehydrogenase was found in genome bins SR1Q7 and SR2Q5 but none of the subunits of formate dehydrogenase were found in these genome bins. So, the species represented by *Quinella* genome bin SR3Q1 might be able to produce or use formate in some way, or the contig containing the formate dehydrogense and related genes was a contaminated contig in this bin.

Acetaldehyde dehydrogenase and NAD-dependent alcohol dehydrogenase are needed to produce ethanol[53]. All four *Quinella* genome bins were searched for these enzymes, first using the automated GAMOLA2 search tool and then by BLAST-based searching of the reviewed ethanol dehydrogenases amino acid sequence of *Streptococcus mutans* against *Quinella* genome bins. Even though GAMOLA2 assigned gene 413 of genome bin SR1Q7 as an ethanol dehydrogenase, the BLAST search using the ethanol dehydrogenase of *Streptococcus mutans* suggested this gene is absent from all four *Quinella* genome bins. Gene 413 from *Quinella* genome bin SR1Q7 was most similar (27.8 to 37.7 % identity) to a lactaldehyde reductase from *Selenomonas bovis*, based on BLAST, and not to known ethanol dehydrogenases. This suggests that *Quinella* probably does not produce ethanol.

A series of enzymes is needed for butyrate formation. Acetyl-CoA is first converted to butyryl-CoA in a four-step process catalysed by acetyl-CoA acetyltransferase, hydroxybutyryl-CoA dehydrogenase, 3-hydroxybutyryl-CoA dehydratase and butyryl-CoA dehydrogenase. Butyryl-CoA is then converted to butyryl-phosphate by phosphotransbutyrylase and then finally to butyrate by butyrate kinase[54, 55]. None of these enzymes were found in any of the *Quinella* genome bins, indicating that *Quinella* does not produce butyrate.

Supplementary Note 14. MMCD and OACD

Methylmalonyl-CoA decarboxylase (MMCD) and oxaloacetate decarboxylase (OACD) are membrane-bound class II decarboxylases that catalyse Na^+ transport from the cytoplasm to the periplasm or cell exterior[56]. The Na^+ gradient that is generated can be used to form ATP[57]. MMCD is composed of four[58, 59] or five subunits[60]: alpha (α or MmdA), beta (β or MmdB), gamma (γ or MmdC), delta (δ or MmdD) and epsilon (ε or MmdE). In contrast, OACD only contains three subunits: alpha (α or OadA), beta (β or OadB) and gamma (γ or OadC)[58]. Initial BLAST analysis of inferred amino acid sequences from all four *Quinella* genome bins suggested that *Quinella* has all the genes that code for the α, β, γ and δ subunits of MMCD but only the α subunit of OACD. Members of the genus *Veillonella* have been extensively studied to characterise MMCD and its subunits[58, 60, 61, 62, 63], whereas *Klebsiella pneumoniae, Salmonella typhimurium* and *Vibrio cholerae* have been used for OACD characterisation[64, 65, 66, 67, 68, 69]. Only one crystal structure of MMCD is available. This is from *Escherichia coli*[70], but this reference is of limited use for making generalisations because of the low sequence similarity with the majority of reviewed MMCD. There is no complete crystal structure available for the OACD complex, but an X-ray crystal structure of the carboxyltransferase domain (part of the α subunit) from *Vibrio cholerae* is available and has been studied in detail[69].

All four *Quinella* genome bins appeared to contain genes coding for MMCD of the four subunit type rather than the five subunit type. These subunits were identified by BLAST analysis of the *Quinella* genomes against several databases (nr database, pfam and Tigerfam) using GAMOLA2 as a search tool, and then confirmed by BLAST searching of the *Veillonella parvula* MMCD subunits[71] against the complete inferred amino acid sequences of all four *Quinella* genome bins. When the same method was applied using *Klebsiella pneumoniae* OACD subunits sequences as references, only α subunits of OACD were found. BLAST searches for the β subunit were unsuccessful. The best identity sequences found were the MMCD β subunit genes. Similarly, no OACD γ subunit sequences were found in any of the *Quinella* genome bins.

Each subunit of MMCD and OACD from the *Quinella* genome bins was analysed separately to identify conserved amino acid residues and to understand key features. For MMCD, homologues from *Veillonella parvula, Propionigenium modestum, Anaerolinea thermophila, Thermococcus gammatolerans,* and *Pyrococcus abyssi* were used as reference amino acid sequences. For OACD, homologues from *Klebsiella pneumoniae, Vibrio cholerae, Salmonella typhimurium,* and *Klebsiella variicola* were used. All subunit sequences were aligned and phylogenetic trees were constructed.

The inferred amino acid sequence lengths of the α subunit of MMCD and OACD are different and the proteins possess different functions. MmdA sequences of *Quinella* were 58.2 to 79.0% similar to reference MmdA sequences and less than 11% identical to OadA references. This strongly supports their identification as α subunits of MMCD and not of OACD. In contrast, amino acid sequences of OadA from the *Quinella* genome bins were 40.2 to 42.4% identical to the reference OadA sequences and less than 12% identical to MmdA reference sequences, again confirming their likely classification. Furthermore, in a phylogenetic analysis, the *Quinella* decarboxylase α subunits (MmdA and OadA types) sequences grouped with their respective reference sequences, with greater than 99% bootstrap support (Supplementary Figure 21). This indicated that all four *Quinella* genome bins contained genes coding for both OadA and MmdA proteins.

The α subunit comprises of three domains (Supplementary Figure 22): a *N*-terminus domain of 450 amino acid residues and possessing the carboxyltransferase catalytic site, a *C*-terminus domain of 70 amino acid residues which contains a conserved biotin-binding lysine (35 residues upstream of the *C*-terminus), and an association domain made up of a total of 40 amino acids of both the *N*- and *C*-termini and that is necessary for binding the α subunit to the *C*-terminus of the γ subunit[66, 68, 69]. It was interesting to find that OadA from the *Quinella* genome bins only contained the *N*-terminus domain and that the biotin-binding and association domains were missing when compared with reference sequences (Supplementary Figure 22). So, even though OadA was present in *Quinella* genomes, it may not function as a normal OadA since it only appeared to be able to catalyse the carboxyltransferase reaction.

In contrast, the *Quinella* genomes contained genes coding for full-length (around 509 amino acid residues) sequences of the α subunit of MMCD. However, unlike normal OACD, α subunits of MMCD are only involved in catalysing the carboxyltransferase activity[72] and do not contain association and biotin-binding domains. In MMCD, the biotin-binding domain necessary for function is part of the γ subunit (MmdC). Because of the unavailability of structures, it was not possible to identify key amino acid residues of the α subunit of MMCD. Furthermore, sequence similarity between carboxyltransferase domain of OadA and MmdA is poor[60], which restricts using OadA as references to predict key amino acid residues in MmdA.

The β subunits of MMCD and OACD are highly hydrophobic, integral membrane proteins[66], and contain binding sites for Na^+ translocation across the cell membrane[72, 73]. Beta subunit amino acid sequences of MMCD and OACD are >50% similar to each other and are argued to serve the same function[60]. Genes encoding for β subunits of membrane-bound decarboxylases were present in all

four *Quinella* genome bins, but *Quinella* bin SR1Q5 contained two copies. When the β subunit genes of bin SR1Q5 were inspected in detail, it was found that each of these were present at the end of a different contig. The overlapping regions of these sequences were 100% identical to each other, so these genes were assembled and the consensus sequence was used as one MmdB sequence (SR1Q5_307/1610). When the putative β subunit amino acid sequences from the *Quinella* genome bins were aligned with reference β subunit sequences of MMCD (MmdB) and OACD (OadB), it was found that all sequences were >43% similar to each other. However, the *Quinella* β subunit amino acid sequences were more similar to MmdB (>76% identity with *V. parvula* MmdB) than to OadB (<51% similar to *K. pneumonia* and *S. typhimurium* OadB). Furthermore, a region of approximately 57 amino acid residues is found in all OadB sequences downstream from the *N* terminus[62], but this region was absent from the *Quinella* β subunit amino acid sequences (Supplementary Figure 23a). So, based on the high percentage similarities and the absence of these extra amino acids, it was concluded that the β subunits present in *Quinella* genomes are MmdB.

The β subunit membrane topology model of *K. pneumoniae* predicted by Jockel et al.[67] was used as a reference to understand MmdB from the *Quinella* genomes. Topology analysis suggested that the β subunit contained 9 transmembrane regions (Supplementary Figure 23b). The extended 57 amino acid residues of OadB are in the first cytoplasmic loop and may provide stability to the enzyme complex. In the *Quinella* β subunits this loop is smaller, like in other MmdB[60]. Site-specific mutagenesis studies[67, 74, 75, 76] on OadB of *Klebsiella pneumoniae* have uncovered some key amino acid residues and predicted their roles. These residues are also found in the closely-related MmdB[67]. Mutations in D203 (helix III) and S382 (helix VIII) lead to complete loss in decarboxylase and Na^+ transport activity of OACD, supporting their identification as Na^+ binding sites[74, 75]. However, S382 can be replaced by T382 or D382 without affecting the decarboxylase and Na^+ transport ability. Similarly, G377 (helix VIII) may be involved in a Na^+ conducting channel or serve as a contact site for other helices, and so cannot be replaced by any other residue. A change of N373 (helix VIII) to L373 or D373 slightly affects the decarboxylase activity. Furthermore, Y227 (helix IV) is an important residue for high catalytic activity of enzyme complex but changes to C227 or F227 can increase the catalytic activity. Y229 (helix IV) takes part in a proton conduction network which leads to decarboxylation of carboxybiotin. All of these important residues (D203, S382, G377, N373, Y227 and Y229) were found to be conserved in the putative MmdB of all four *Quinella* genome bins when aligned with OadB of *K. pneumoniae* (Supplementary Figure 23a).

Genes coding for γ subunits of OACD were not found in any of the *Quinella* genome bins. In contrast, all four *Quinella* bins contained genes coding for full length γ subunits (MmdC) of

MMCD, which serve almost the same function as the biotin-binding *C*-terminus domain of the α subunit of OACD. The γ subunits also play a major role in MMCD assembly, by binding to α and δ subunit of MMCD[77]. These γ subunit sequences were aligned with MmdC sequences of *V. parvula* and *P. modestum* and OadA *C*-terminus sequences of *K. pneumoniae* and *S. typhimurium.* The *Quinella* MmdC sequences were 54.7% to 59.7% similar to MmdC of *V. parvula* and 47.4% to 49.8% similar to that of *P. modestum*. They were only 31.7 % to 38.7 similar to the OadA *C*-terminus sequences. Like other proteins containing a biotin prosthetic group, MmdC of *Quinella* also contained a putative biotin-binding lysine residue (35 residues upstream of the *C*-terminus) within a highly-conserved amino acid motif, LEAMKM[59] (Supplementary Figure 24a). Furthermore, the alanine-proline linker (around residues 31 to 60) was also found in the *N*-terminus of all of *Quinella* MmdC. This linker provides flexible movement for the biotin group to move between the catalytic centres of different biotin-containing enzymes[60].

Genes coding for the δ subunit of MMCD were found in all four *Quinella* genome bins. The δ subunit of *Quinella* was found to be more similar to that of *V. parvula* (32.8% to 37.4% amino acid similarity) than to that of *P. modestum* (21.2% to 24.3%), but were only 14.8 to 18.7% identical to the γ subunit of OADC of *K. pneumoniae* and *S. typhimurium*. Even though very low sequence similarity was found between the δ subunits of MMCD and the γ subunits of OACD, they share similarities in their hydrophobicity patterns and the presence of identical alanine-proline linkers[62] (Supplementary Figure 24b), and appear to have similar functions[78]. Importantly, the *N*-termini of both are predicted to be anchored in the membrane, indicating that the hydrophobic *N*-terminus could bind to the hydrophobic β subunit while the hydrophilic *C*-terminus could bind to the α subunit[78]. MmcD sequences lack the triple histidine that serves as a Zn^+ binding site at the *C*-terminus, suggesting that divalent metal ions are not present in biotin-containing carboxyltransferase enzymes that react with thioester (CoA-bound) substrates, in contrast to those that react with keto acid substrates[79]. The *Quinella* MmcD sequences also lack these triple histidine residues. In *V. parvula*, there was an extra subunit reported (epsilon), but in other bacteria with MMCD, like *P. modestum*, this subunit is absent, as it appears to be from all four *Quinella* genome bins. The function of this subunit is still unknown but its strong sequence similarity with the *C*-terminus of δ subunit indicated that its origin may be from a duplication of the δ subunit[60].

In summary, all four *Quinella* genome bins contain genes for the essential subunits needed to assemble Na^+-translocating methylmalonyl-CoA decarboxylases (MMCD). These have the α subunit (MmcA) containing the carboxyltransferase catalytic site, the integral membrane β subunit (MmcB) with important conserved amino acid residues involved in Na^+ translocation, the γ subunit

(MmcC) containing a biotin-binding lysine residue, and the δ subunit (MmcD) with the alanine-proline linker. On the basis of available literature, a probable schematic of MMCD (Supplementary Figure 25) may be drawn, where the hydrophobic integral membrane β subunit is connected to the hydrophobic *N*-terminus δ subunit, and the highly hydrophilic *C*-terminus of the δ subunit is connected to the γ subunit.

Quinella does not appear to have all the genes needed for oxaloacetate decarboxylase (OADC), only containing the gene for the α (carboxyltransferase, OadA) subunit. While it can be speculated that the OadB and OadC subunits were missed in the sequencing, this seems unlikely as the four *Quinella* bins all have genes for OadA and all do not have genes for OadB or OadC for, unless the latter two were rejected at the assembly steps. It is also possible that OACD activity is conferred in a different way in *Quinella*. One possibility is that functional OACD in *Quinella* uses the OACD α (carboxyltransferase) subunit in a hybrid with other subunits from the MMCD complex: the β subunit (which is similar to the β subunit of OACD except for 52 extra amino acid residues in a cytoplasmic loop), the γ subunit and the δ subunit (which has a similar hydrophobicity to that of γ subunit of OACD but no sequence homology). This speculation is made appealing by the unusual structure of the α subunit of the OACD of *Quinella*. In contrast to other OadA, it lacks the biotin-containing domain. In MMCD, that function is found in the γ subunit. A normal OadA in conjunction with MmdC would contain two biotin-binding domains, so that loss of this domain from the *Quinella* OadA would render the interaction with the MMCD components possible (Supplementary Figure 25). Here it is also speculated that the OACD would catalyse the conversion of pyruvate to oxaloacetate (Supplementary Figure 13) instead of its well-known direction (oxaloacetate to pyruvate). However, even in the absence of a functional OACD (because of the lack of OACD specific β and γ subunits), *Quinella* could still form oxaloacetate from phosphoenolpyruvate by using phosphoenolpyruvate carboxykinase, and genes for this were present in all four genome bins (Supplementary Figure 13, Supplementary Table 14). This was confirmed by BLAST against the Swiss-Prot database[80]. The amino acid sequences of phosphoenolpyruvate carboxykinases in the *Quinella* genome bins were ≥70.0% similar to amino acid sequences of reviewed phosphoenolpyruvate carboxykinase (ATP-forming) of *Agathobacter rectalis, Eubacterium eligens, Parabacteroides distasonis and Bacteroides fragilis*. Using phosphoenolpyruvate carboxykinase to form oxaloacetate will result in formation of one ATP molecule. In contrast, the two-step conversion process will result in generation of one ATP molecule by pyruvate kinase during conversion of phosphoenolpyruvate to pyruvate, followed by the generation of oxaloacetate from pyruvate via the reversed OADC, which would be driven by

one Na^+ from outside the cell to the inside. This is equivalent to using approximately 3/10 ATP to carry out the carboxylation. Oxaloacetate decarboxylation (the reverse reaction) in *K. pneumoniae* extrudes one Na^+[81], which then can be used to generate ATP via an ATP synthase that generates approximately 3 ATP per 10 Na^+[82]. The two-step process therefore yields approximately 7/10 ATP for the same conversion. It is unclear which option is used by *Quinella*.

Supplementary Note 15. Fumarate reductase

Fumarate reductase (quinol:fumarate reductase; QFR) catalyses the reduction of fumarate to succinate coupled to the oxidation of quinol to quinone[83]. This reaction can also be reversed, coupling quinone reduction to quinol with succinate oxidation to fumarate by succinate dehydrogenase (succinate:quinone reductase; SQR)[84]. These enzymes are structurally and mechanistically similar with very high sequence similarity and cofactor composition, and belong to the succinate:quinone oxidoreductase (SQOR) superfamily[85, 86]. QFR is involved in anaerobic respiration[87, 88] whereas SQR participates in aerobic respiration[84]. Under certain conditions they can functionally replace each other[89]. The SQORs are mainly composed of four subunits: two hydrophilic subunits, A and B, and two hydrophobic integral membrane subunits, C and D. In some instances, instead of two, only one comparatively large hydrophobic[83] integral membrane subunit is found, which is predicted to have evolved from fusion of the C and D subunits[83, 85]. SQORs can be classified into five different types based on their hydrophobic domains and heme *b* content[83, 85]. Type A contain two hydrophobic subunits and two heme, type B one hydrophobic subunit and two heme, type C two hydrophobic subunits and one heme, type D two hydrophobic subunits and no heme, while type E contain two hydrophobic subunits which are very different from those of the other types and no heme[90]. All four *Quinella* genome bins appeared to have the genes coding for three subunits of QFR (Supplementary Table 14). These were the two hydrophilic subunits A and B, and one hydrophobic subunit C. These subunits were identified by BLAST analysis of the *Quinella* genomes against several databases (nr database, pfam and Tigerfam) using GAMOLA2 as a search tool, and then confirmed by BLAST of the sequences of the SQOR subunits from *Escherichia coli* and *Wolinella succinogenes* against the complete inferred amino acid sequences of all four *Quinella* genomes. Subunit D was not found by either of these methods, suggesting that *Quinella* SQORs were type B (the only type with one hydrophobic subunit)[90].Type B contains SQR from *Bacillus subtilis* and *Paenibacillus macerans* and QFR from *Campylobacter jejuni* and *Helicobacter pylori* and *Wolinella succinogenes*.

Supplementary Note 16. Detailed analysis of *Quinella* QFR

Two crystal structures of QFR are available, one from *Escherichia coli* [86] and another from *Wolinella succinogenes*[85]. These QFR amino acid sequences were used as references to understand the possible QFR from the *Quinella* genomes. All subunit sequences from *Quinella* were aligned with the reference amino acid sequences to identify conserved key amino acid residues.

The hydrophilic flavoprotein A subunits from all four *Quinella* genomes were 31.4% to 31.1% similar to the SQOR subunit of *E. coli* and around 27.9% to 28.5% similar to that from *W. succinogenes*. Fumarate binding sites are present between the FAD-binding domain and capping domain (among four domains) of subunit A[90]. FAD-binding residue H45[85, 86] of *Quinella* and *E. coli* and H43 of *W. succinogenes* were conserved in all sequences. Similarly, other important amino acid residues with key functions were also found to be conserved in the predicted *Quinella* enzymes, such as G52 (which accepts a hydrogen bond from FAD), a 'HPT triad' (H233, P234 and T235), and R404 and A409 (in *W. succinogenes*) involved in proton movement. Furthermore, a dicarboxylate-binding site containing two arginine (R301 and R404) and one histidine (H369) found in *W. succinogenes* was also found in the enzymes predicted from the *Quinella* genomes (Supplementary Figure 26). However, it was interesting to find that S45 was conserved in all four *Quinella* enzymes and apparently homologous to S44 in *Wolinella succinogenes* (one of the 11 residue that bind to FAD). This was replaced by threonine (T45 in *E. coli*) in most of the SQORs[85]. Based on these similarities, it appears that the *Quinella* genomes code for subunit A of a QFR.

The second hydrophilic subunit, subunit B, consists of two domains (a *N*-terminal 'plant ferredoxin domain' and a *C*-terminal 'bacterial ferredoxin domain') and three Fe-S clusters[86]. Three of the four *Quinella* genome bins contained full length amino acid sequences of subunit B. The fourth, *Quinella* genome bin SR1Q7, contained a sequence coding for only the first 35 residues of subunit B but it was 100% identical to the same region of the subunit B sequences in the other *Quinella* genome bins amino acid sequences, suggested that that strain also contained a QFR subunit B. This short fragment was at the end of a contig, and so is probably not a gene remnant. The amino acid sequences of the *Quinella* subunit B were around 25% similar to the homologous sequences from *W. succinogenes* and *E. coli*. Fumarate reductase contains three Fe-S clusters (2Fe-2S, 3Fe-4S and 4Fe-4S) which are coordinated with cysteine residues[85, 86]. The *Quinella* subunit B sequences contained four conserved cysteine residues that coordinate with 2Fe-2S clusters that are in contact with plant ferredoxin domain and subunit A[85]. The 3Fe-4S cluster coordinates with three cysteine residues whereas the 4Fe-4S is coordinated with four cysteine residues[85, 86], and all of these were

conserved in *Quinella* (Supplementary Figure 26). However, like *W. succinogenes*, the enzyme in *Quinella* contains an extra cysteine residue (C158 in *Quinella*) which is replaced by leucine in *E. coli* (Supplementary Figure 26b). The 3Fe-4S and 4Fe-4S are bound by the bacterial ferredoxin domain, which is in contact with the hydrophobic subunit(s)[85, 86].

Genes coding for only one hydrophobic subunit of QFR were found in the *Quinella* genome bins. Sequence alignment of this subunit C with the subunit C from the *E. coli* SQOR (which also contains only one hydrophobic subunit) showed <15.6% identity, suggesting that it is quite different to subunit from the one in *E. coli*. It was also <17.8% similar to the subunit C from *W. succinogenes*. Therefore, subunit C sequences from *Bacillus subtilis*, *Paenibacillus macerans*, *Campylobacter jejuni* and *Helicobacter pylori*, which are B type SQOR[83, 90, 91], were included in the analysis. The *Quinella* sequences were 33.8% to 39.3% similar to those of *B. subtilis* and *P. macerans* and only around 15% similar to those of *C. jejuni* and *H. pylori* (Supplementary Table 17), suggesting that *Quinella* QFR does belongs to type B and is more similar to those from other Gram positive bacteria (*B. subtilis* and *P. macerans*) (Supplementary Table 17). Similarly, transmembrane topology analysis using SPOCTOPUS[18] predicted that subunit C of *Quinella* is composed of five membrane spanning helices (Supplementary Figure 27), a characteristic feature of QFR with only one hydrophobic subunit[85]. In contrast, subunits C and D each contain three membrane spanning helices[86] in those fumarate reductases with two hydrophobic subunits.

Histidine residues (H27, H71, H114 and H158 in *Quinella*) that are the axial ligands for proximal and distal hemes[85] were also conserved in all four predicted *Quinella* proteins (Supplementary Figure 27), suggesting that *Quinella* QFR subunit C binds two heme. This is a further indicator of a type B SQOR[90]. An essential glutamic acid residue (E66 in *W. succinogenes*) is involved in menaquinol oxidation[92] and was also conserved in all four *Quinella* genomes. In summary, all QFR subunits found in the *Quinella* genome bins contained conserved key amino acid residues that match with QFR of type B SQOR (*W. succinogenes*), suggesting that *Quinella* has a fully functional QFR. This suggests that the enzyme functions to reduce fumarate to succinate, rather than to oxidise succinate to fumarate.

Supplementary Note 17. ATP synthase

All four *Quinella* genome bins contained genes that code for all eight subunits of the bacterial type ATP synthase (F_1F_o). The deduced amino acids of these had the greatest similarity to ATP synthase subunits of *Selenomonas* spp. The F_o part of the bacterial ATP synthase is the integral membrane ion-translocating complex containing α, β, γ, δ and ε subunits whereas the F_1 part is a peripheral membrane unit on the cytoplasmic face and is composed of a, b and c subunits[93]. Grüber et al.[94] identified conserved amino acid residues that allow classification of ATP synthases as either Na^+- or H^+-binding types in a wide range of bacteria and archaea. They analysed amino acid sequences of subunit c (the ion carrier) and suggested that motif Q/E…E T/S (at positions 32, 65 and 66 in the *Ilyobacter tartaricus* protein) are conserved in Na^+-binding ATP synthases, while N/I…E/D A were found conserved in the corresponding positions in H^+-binding bacterial ATP synthases. Amino acid sequences from the ATP synthase c subunits from the *Quinella* genome bins were aligned with the reviewed and experimentally verified bacterial sequences[94] from the Na^+-binding c subunits from *Acetobacterium woodii*, *Ilyobacter tartaricus*, and *Propionigenium modestum*, and the H^+-binding c subunits from *Bacillus subtilis* and *Escherichia coli*, to identify the positions of these diagnostic residues. The sequences from the four *Quinella* bins did not contain Q/E at position 32 or T/S at position 66 as would be expected for Na^+-binding c subunits. Instead, at those positions the amino acids were D at position 32 and A at position 66, indicative of a H^+-coupled enzyme. The LxEALxxI motif in the sequences from the *Quinella* genome bins which includes positions 65 and 66, was similar to that from *Bacillus subtilis* (LxEALxxI) (Supplementary Figure 28), which is a H^+-binding ATP synthase[95]. Furthermore, the overall amino acid sequence was also most similar to that of *Bacillus subtilis* (42.9%) than to the other reviewed reference sequences compared (34.1 to 38.6%). Therefore, on the basis of sequences similarity and conserved amino acid residues, *Quinella* ATP synthases appear to be H^+-binding enzymes.

Supplementary Note 18. Na^+/H^+ antiporter

All four *Quinella* genome bins contained genes that code for a Na^+/H^+ antiporter. So far, seven structurally different antiporter genes have been found in bacteria[96]. BLAST was used to compare the amino acid sequences from the putative Na^+/H^+ antiporter genes in the *Quinella* genome bins to reviewed sequences of all seven different types of antiporters. This comparison showed that the *Quinella* genome bins contained sequences most similar to a putative *nhaC* from *Selenomonas ruminantium* (72% to 74% identity). There were no matches to validated antiporters. The Na^+/H^+ antiporter assists in maintaining pH homeostasis and may also help lowering cytoplasmic Na^+ concentration[96]. However, in *Quinella*, it could be speculated that the Na^+/H^+ antiporter plays a role in converting the Na^+ gradient generated by the methylmalonyl-CoA decarboxylase into a H^+ gradient that can be used by the H^+-translocating ATP synthase to generate ATP. This would be especially true if oxaloacetate is formed from phosphoenolpyruvate by a phosphoenolpyruvate carboxykinase rather than by a reversed oxaloacetate decarboxylase. In the case of the former, the Na^+ gradient generated by the methylmalonyl-CoA decarboxylase would not be needed to drive the synthesis of oxaloacetate and could be converted to a H^+ gradient by the antiporter.

Supplementary Note 19. Hydrogenase (Ni-Fe)

BLAST-based analyses of all four *Quinella* genome bins against different databases revealed that all of them contained complete sets of genes that code for the large subunit, the small subunit and *b*-type cytochrome of hydrogenases (Supplementary Table 14). BLAST analyses of these *Quinella* hydrogenases against the hydrogenase database of Greening et al.[97] suggested that the hydrogenases in all four genome bins were closely related to the hydrogenase of *Selenomonas ruminantium* (NCBI accession no. WP_029541750), which was classified as a NiFe-type oxygen-tolerant membrane-bound H_2-uptake hydrogenase[97].

The large subunits of the three major classes of hydrogenases contain different H_2-binding metal centres: L1 and L2 motifs for [NiFe]-hydrogenases[98]; P1, P2 and P3 motifs for [FeFe]-hydrogenases[98]; and Cys176 in [Fe]-hydrogenases[99]. L1 and L2 motifs (Supplementary Figure 29) surrounding the metal-ligating cysteine residues were found in the amino acid sequences of all four *Quinella* hydrogenases, which suggested that *Quinella* has [NiFe]-hydrogenases. However, the variation in consensus motifs meant that further subgrouping was not clear. The L1 motif (xxRICGVCTxxH) was identical to that of subgroup 1d (oxygen-tolerant) type uptake hydrogenases, but the L2 motif (SFDPCxACxxH) was identical to that of subgroup 1e (possible bidirectional hydrogenases) based on the classification of Greening et al.[97]. A phylogenetic analysis was performed using the amino acid sequences of the large subunit of all 1d and 1e type hydrogenase sequences, using five sequences each from types 1a, 1b, 1c, 1f and 1h/5 (to cover all NiFe-hydrogenase type), and all four *Quinella* hydrogenase sequences. The *Quinella* hydrogenase sequences formed a stable cluster (100% bootstrap value) within a radiation that contained only hydrogenase sequences from group 1d, and in the same radiation as all other group 1d sequences (Supplementary Figure 30). Most of the sequences that clustered with them originated from members of the class *Negativicutes. Quinella* is also a member of this class. Again, the large subunit hydrogenase sequence from *Selenomonas ruminantium* (Wp_029541750.1) was found to be the closest relative of the *Quinella* hydrogenases. Therefore, even though the L2 motif matched with group 1e hydrogenases, the *Quinella* hydrogenase sequences may still be classified as belonging to group 1d, as a NiFe-type membrane-bound H_2-uptake hydrogenase.

The initial analyses suggested that *Quinella* has a NiFe-type membrane-bound H_2-uptake hydrogenase that may be further categorised as an oxygen-tolerant type based on the classification proposed by Greening et al.[97]. However, the L2 motif was different to that expected for an oxygen-tolerant (1d) type. Flanagan and Parkin [100] identified and compared the variation in amino acid

sequences involved in binding the metal-cofactors and iron-sulfur (FeS) clusters of the small and large subunits of O_2-tolerant and O_2-sensitive NiFe type membrane-bound hydrogenases. They concluded that conserved cysteine residues play an important role by participating in stabilisation of the unusual proximal FeS cluster in O_2-tolerant and O_2-sensitive sensitive NiFe-type membrane-bound hydrogenases.

Three O_2-tolerant (*E. coli* Hyd-1, *Ralstonia eutropha* and *Aquifex aeolicus*) and three O_2-sensitive (*E. coli* Hyd-2, *Desulfovibrio gigas* and *Desulfovibrio vulgaris*) bacterial hydrogenases were used as references to compare and understand the *Quinella* hydrogenases. When the amino acid sequences of the *Quinella* hydrogenases were aligned and compared with those of the six reference hydrogenases, it was found that amino acid similarity varied from 36.1% to 58.3% but that the key amino acid residues aligned and matched well (Supplementary Figures 29 and 31).

The large and small subunits of membrane-bound hydrogenases contain four co-factors: one NiFe catalytic centre in the large subunit and three FeS clusters in the small subunit. The large, or catalytic, subunit is responsible for H_2 oxidation and contains the NiFe active site. When compared with the reference hydrogenases, four cysteine residues (Cys62, Cys65, Cys608 and Cys610) surrounding the NiFe active site were found to be conserved in the proposed large catalytic subunit of the *Quinella* hydrogenases (Supplementary Figure 29). Furthermore, a histidine residue (His236), a metal-binding amino acid that coordinates with the proximal 4Fe4S or 4Fe3S cluster of the small subunit[101], was also found in the correct location when aligned with the amino acid sequences from the reference hydrogenases (Supplementary Figure 29). The large subunit sequences from the *Quinella* genomes are therefore very similar to those of other O_2-tolerant and O_2-sensitive NiFe type membrane-bound hydrogenases. This was expected, since the large catalytic subunits of O_2-tolerant and O_2-sensitive type hydrogenases are very similar in arrangement and this subunit does not confer the O_2 sensitivity[102].

The small subunit contains three FeS clusters, termed the proximal, medial and distal clusters. The proximal FeS cluster accepts electrons from the large subunit, where the hydrogen is oxidised at the NiFe centre, and the electrons are conducted via the medial FeS to the distal FeS cluster[103]. On the basis of crystallography studies conducted by Shomura et al.[104] and Fritsch et al.[103], the O_2-sensitive NiFe type membrane-bound hydrogenases, termed 1d group type hydrogenases by Greening et al.[97], contain standard 4Fe-4S clusters. In contrast, the O_2-tolerant type, termed group 1e by Greening et al.[97], has a novel proximal 4Fe3S cluster. When the amino acid sequences of the small subunits from *Quinella* were aligned to reference hydrogenase sequences (Supplementary Figure 31), it was

found that the *Quinella* small subunits have four cysteine residues (Cys62, Cys65, Cys160 and Cys194) that should bind a proximal 4Fe-3S cluster instead of the six cysteine residues that bind the 4Fe-4S cluster found in the O_2-tolerant type. In the O_2-tolerant type, there are two extra cysteines (Cys55 and Cys165), and targeted mutation of these two cysteines to glycine can convert an O_2-tolerant hydrogenase to an O_2-sensitive type[105]. This analysis therefore suggests that the small subunit of the *Quinella* hydrogenase is similar to those of O_2-sensitive NiFe type membrane-bound uptake hydrogenases, and therefore probably not bidirectional.

Membrane-bound hydrogenases are proposed to be dimers of heterotrimers, i.e., two copies of each of the large and small subunits and a *b*-type cytochrome (cyt*b*)[104, 106]. The small subunits of the hydrogenases from the *Quinella* genomes also contained three conserved residues (Arg238, Lys263 and Arg311; see Supplementary Figure 31a) that are proposed to be exposed towards the carboxylate group of the heme of the *b*-type cytochrome associated with NiFe type membrane-bound hydrogenases[107]. All four *Quinella* genomes contained genes coding for cytochrome *b* (cyt*b*), the redox partner of membrane-bound hydrogenases. The amino acid sequence of the *Aquifex aeolicus* protein was the most similar to the *Quinella* amino acid sequences, with an average of 38% amino acid similarity. Overall, cyt*b* amino acid sequences of *Quinella* were found to be 26% identical to that of *E. coli* Hyd-1 and 31% identical to that of *Ralstonia eutropha*. All *Quinella* cyt*b* sequences were >81% similar to each other and the *S. ruminantium* cyt*b* was found to be the closest relative by BLAST analysis against the NCBI non-redundant database. Out of 21 conserved surface amino acid sequences from cyt*b* that have contact with the small subunit of the hydrogenases[107], 13 were found in cyt*b* of *Quinella* (Supplementary Figure 32a). Transmembrane topology analysis using SPOCTOPUS[18], suggested that the all four *Quinella* genome bins cyt*b* contain four transmembrane helices (Supplementary Figure 32b), which is reported for other cyt*b* associated with membrane-bound NiFe-hydrogenases[106].

Cytochrome *b* is unstable when removed from the cytoplasmic membrane[108], and to date there is only one crystal structure of a hydrogenase (*E.* coli Hyd-1) with its cytochrome *b* complex available[107]. The amino acid sequence of this enzyme, *E. coli* Hyd-1, plus sequences from *E. coli* Hyd-2, *R. eutropha* and *A. aeolicus* (well annotated and reviewed cyt*b* sequences) were used as references to understand the *Quinella* cyt*b*. The amino acid sequence of the cyt*b* from the *E. coli* Hyd-2 was very different to the other amino acid sequences on the basis of the alignment. Histidine residues (His25, His67, His181 and His195), metal-binding amino acid residues, were conserved in all sequences (Supplementary Figure 32a) except for *E. coli* Hyd-2. At least three (His25, His67, and His195) of the four conserved histidine residues served as ligands for two heme, the carrier for

electron movement from the periplasmic to the cytoplasmic side of the membrane[109], and mutations in any of these conserved histidines leads to total loss of cytochrome *b* reduction by H_2[109, 110, 111]. Furthermore, these four conserved histidines are also conserved in quinone-interacting *b*-type cytochromes[109, 111] and His25, His67, and His195 are required for electron transport from H_2 to fumarate and for quinone reactivity in *Wolinella succinogenes*[111].

To understand the orientation of *Quinella* hydrogenase, membrane protein topology and signal peptides prediction was done using Phobius[17] and SPOCTOPUS[18]. The large subunits from all four *Quinella* genome bins were predicted to be non-cytoplasmic, suggesting they are present outside the cell, although no leader sequences were detected. The small subunits appeared to have one *N*-terminal signal peptide region and contained two transmembrane-helices (Supplementary Figure 31). The position of the proximal FeS cluster of the small subunit that faces towards the large subunit appeared to be adjacent to the signal peptide sequence and so oriented towards the outer surface of the cell, based on the location of the residues that coordinate it as reported by Volbeda et al.[101]. This suggests that the large subunit of the hydrogenase is on the outside of the cell where it is connected to the small subunit, which has three FeS clusters that transfer electrons, ultimately to the fumarate reductase.

In summary, evidence was found for the presence of hydrogenases in all four *Quinella* genome bins. These can be classified as NiFe type membrane-bound H_2-uptake hydrogenases. Based on the inferred presence of a proximal 4Fe3S cluster in the small subunit, it appears that the *Quinella* hydrogenase is an O_2 sensitive type, even though other analyses (overall amino acid similarity and phylogeny) suggested that it is an O_2 tolerant type. Confirmation of this will require experimental evidence. Evidence was found to suggest that the hydrogenase is associated with a *b*-type cytochrome that contains ligands for two heme groups, and that the reaction with hydrogen occurs on the outer face of the cell membrane.

Supplementary Note 20. Rnf complex

The *Quinella* genome bins were searched for evidence of the *rnf* gene cluster *rnfCDGEAB* that codes for the ferredoxin:NAD$^+$-oxidoreductase complex and is composed of at least six subunits. This enzyme complex might allow H_2 generation from NADH and ferredoxin via an electron bifurcating mechanism[112]. Reviewed rnf complex amino acid sequences from the bacteria *Acetobacterium woodii* and *Rhodobacter capsulatus* were used as queries in a BLAST search against the *Quinella* genome bins. None of the subunits were found in any of the *Quinella* genome bins using this approach. However, the GAMOLA2 search tool suggested that a gene sequence potentially coding for subunit D (SR3Q1_1981; 29% identity with NADH:ubiquinone oxidoreductase of *Anaerococcus* sp. PH9) was present in *Quinella* genome bin SR3Q1, and that genes for subunit E (SR1Q7_1676; 78% identity with putative sugar-specific permease SgaT of *Candidatus* Arthromitus sp. SFB-3) and subunit G (SR1Q7_1737; 49% identity with FMN-binding protein *Selenomonas* sp. CM52) were found in bin SR1Q7. All six subunits are required for a fully functional rnf complex[113], and so it can be assumed that *Quinella* does not contain a fully functional rnf complex. Consequently, the subunits detected may come from assembly or binning errors, or play a different role in these bacteria. This conclusion is supported by the generally complete nature of other multi-subunit enzyme complexes found in these four genome bins.

Taxa	*Quinella* Candidate species 4			*Quinella* Candidate species 3					*Quinella* Candidate species 6					*Quinella ovalis*									*Quinella* candidate species 1					*Quinella* candidate species 2		*Quinella* candidate species 7				*Quinella* candidate species 5		*Selenomonadaceae* candidate genus 1 candidate species 1			*Selenomonadaceae* candidate genus 1 candidate species 2	
	MF184892	MF184893	MF184888	EF436437	EF436435	EF436434	EF436436	EF436332	MF184915	MF184920	MF184919	MF184917	DQ673489	MF184871	MF184873	MF184872	MF184874	MF184899	MF184876	M62701	MF184889	AB494823	MF184897	MF184898	MF184895	MF184896	MF184894	MF184914	MF184916	DQ673510	DQ673569	DQ673570	EF436320	EF436424	EF436425	DQ673559	DQ673560	DQ673499	MF184882	DQ673561
***Quinella* Candidate species 4**	100.0	97.9	97.2	94.0	93.9	93.8	93.8	94.0	94.6	93.9	94.5	94.6	94.6	93.9	94.0	93.8	92.9	94.2	93.7	93.9	94.4	93.3	92.6	94.0	94.4	94.2	94.2	95.4	94.2	93.8	93.9	93.7	93.3	91.8	91.8	89.4	89.4	89.6	87.2	89.4
	97.9	100.0	96.4	93.8	93.9	93.7	93.9	93.9	93.9	94.2	93.9	94.4	94.2	94.2	94.2	94.2	93.2	94.4	93.9	93.7	93.9	93.1	92.4	93.7	94.0	93.9	93.9	94.6	93.6	93.4	93.5	93.3	93.2	91.6	91.6	89.3	89.3	89.3	87.3	88.8
	97.2	96.4	100.0	95.3	95.3	95.1	95.2	95.2	94.4	94.4	94.6	95.0	94.2	94.2	94.3	94.1	93.3	94.3	94.2	93.7	94.8	93.8	93.3	94.7	95.1	94.9	95.0	94.7	93.8	93.8	93.9	94.1	93.6	92.5	92.5	89.8	89.8	89.8	87.5	89.7
***Quinella* Candidate species 3**	94.0	93.8	95.3	100.0	99.8	99.7	99.7	99.2	93.2	94.0	93.1	94.1	94.4	94.2	94.3	94.1	93.4	94.4	94.2	94.8	94.3	93.7	93.1	94.4	94.9	94.7	94.9	94.0	92.6	94.2	94.3	94.1	94.3	93.4	93.4	90.8	90.8	91.0	88.0	91.0
	93.9	93.9	95.3	99.8	100.0	99.7	99.8	99.3	93.1	94.0	93.0	94.2	94.5	94.2	94.2	94.2	93.3	94.4	94.1	94.9	94.2	93.8	93.1	94.3	94.8	94.7	94.9	94.0	92.5	94.3	94.3	94.0	94.4	93.4	93.4	90.7	90.7	90.9	87.9	90.9
	93.8	93.7	95.1	99.7	99.7	100.0	99.7	99.2	93.0	93.8	92.9	94.1	94.4	94.0	94.1	94.0	93.1	94.2	94.0	94.7	94.1	93.6	93.0	94.2	94.7	94.5	94.7	93.8	92.4	94.1	94.2	93.9	94.3	93.3	93.3	90.6	90.6	90.8	87.8	90.7
	93.8	93.9	95.2	99.7	99.8	99.7	100.0	99.2	93.2	94.0	93.1	94.3	94.6	94.1	94.2	94.1	93.2	94.3	94.0	94.8	94.2	93.7	93.1	94.2	94.7	94.6	94.8	93.9	92.6	94.2	94.3	94.0	94.3	93.4	93.4	90.7	90.7	90.9	87.9	90.9
	94.0	93.9	95.2	99.2	99.3	99.2	99.2	100.0	93.1	94.3	93.0	94.2	94.5	94.4	94.5	94.4	93.6	94.7	94.4	94.9	94.5	94.1	93.4	94.6	95.1	94.9	95.1	94.2	92.8	94.5	94.6	94.3	94.7	93.7	93.7	90.6	90.6	90.8	87.9	90.9
**Quinella Candidate species 6**	94.6	93.9	94.4	93.2	93.1	93.0	93.2	93.1	100.0	96.8	96.5	95.8	95.6	93.6	93.7	93.6	91.8	94.4	93.4	93.7	93.7	91.9	92.2	93.6	94.0	93.9	93.8	92.7	94.7	93.3	93.3	93.8	93.5	93.3	93.3	89.3	89.3	89.4	88.5	88.7
	93.9	94.2	94.4	94.0	94.0	93.8	94.0	94.3	96.8	100.0	97.2	97.4	95.9	93.7	93.8	93.7	93.0	94.7	93.6	93.8	93.5	93.9	92.7	94.1	94.5	94.4	94.5	94.0	95.6	94.1	94.2	95.2	94.7	94.5	94.5	89.5	89.5	89.6	89.7	90.2
	94.5	93.9	94.6	93.1	93.0	92.9	93.1	93.0	96.5	97.2	100.0	96.2	96.8	93.5	93.5	93.5	91.2	94.5	93.5	93.6	93.6	92.0	92.8	94.2	94.6	94.4	94.4	92.4	95.1	93.4	93.5	94.1	93.8	93.5	93.5	89.0	89.0	89.1	88.7	88.8
	94.6	94.4	95.0	94.1	94.2	94.1	94.3	94.2	95.8	97.4	96.2	100.0	96.6	92.8	92.9	93.0	91.6	93.8	92.8	93.3	92.9	92.0	92.4	93.7	94.1	93.9	93.9	92.0	95.1	93.7	93.8	94.3	93.9	93.7	93.7	89.5	89.5	89.6	89.0	89.1
	94.6	94.2	94.2	94.4	94.5	94.4	94.6	94.5	95.6	95.9	96.8	96.6	100.0	94.3	94.4	94.4	93.4	95.0	93.9	95.1	94.8	94.0	93.0	94.2	94.6	94.5	94.5	94.0	94.3	94.2	94.3	94.5	94.2	92.9	92.9	90.3	90.3	90.4	87.9	89.2
Quinella ovalis	93.9	94.2	94.2	94.2	94.2	94.0	94.1	94.4	93.6	93.7	93.5	92.8	94.3	100.0	100.0	99.7	98.8	98.2	99.4	98.2	98.0	96.9	94.9	96.4	96.8	96.6	96.7	95.3	94.0	94.6	94.7	94.3	94.5	92.8	92.8	90.1	90.1	90.3	88.6	90.7
	94.0	94.2	94.3	94.3	94.2	94.1	94.2	94.5	93.7	93.8	93.5	92.9	94.4	100.0	100.0	99.8	98.9	98.2	99.5	98.3	98.1	97.0	95.0	96.4	96.9	96.7	96.8	95.3	94.0	94.7	94.7	94.3	94.5	92.9	92.9	90.1	90.1	90.3	88.6	90.8
	93.8	94.2	94.1	94.1	94.2	94.0	94.1	94.4	93.6	93.7	93.5	93.0	94.4	99.7	99.8	100.0	98.7	98.0	99.2	98.2	97.8	96.9	94.9	96.2	96.6	96.5	96.6	95.1	94.0	94.6	94.7	94.1	94.5	92.8	92.8	90.1	90.1	90.3	88.6	90.7
	92.9	93.2	93.3	93.4	93.3	93.1	93.2	93.6	91.8	93.0	91.2	91.6	93.4	98.8	98.9	98.7	100.0	97.1	98.4	97.3	97.3	95.1	94.0	95.4	95.8	95.7	95.8	93.3	92.7	93.7	93.8	93.4	93.5	91.9	91.9	89.2	89.2	89.5	87.9	90.0
	94.2	94.4	94.3	94.4	94.4	94.2	94.3	94.7	94.4	94.7	94.5	93.8	95.0	98.2	98.2	98.0	97.1	100.0	97.7	97.3	96.9	95.8	94.6	96.0	96.4	96.3	96.4	95.3	94.1	95.6	95.7	95.3	95.4	93.7	93.7	89.6	89.6	89.8	88.5	90.3
	93.7	93.9	94.2	94.2	94.1	94.0	94.0	94.4	93.4	93.6	93.5	92.8	93.9	99.4	99.5	99.2	98.4	97.7	100.0	97.9	97.8	96.9	95.1	96.6	97.0	96.9	96.9	95.2	93.9	94.7	94.7	94.3	94.8	93.2	93.2	90.2	90.2	90.4	88.6	91.0
	93.9	93.7	93.7	94.8	94.9	94.7	94.8	94.9	93.7	93.8	93.6	93.3	95.1	98.2	98.3	98.2	97.3	97.3	97.9	100.0	97.4	96.3	94.8	95.7	96.5	96.4	96.4	95.8	94.2	94.9	94.9	94.9	95.2	93.4	93.4	90.8	90.8	91.1	88.8	91.0
	94.4	93.9	94.8	94.3	94.2	94.1	94.2	94.5	93.7	93.5	93.6	92.9	94.8	98.0	98.1	97.8	97.3	96.9	97.8	97.4	100.0	96.7	95.1	96.5	96.9	96.8	96.9	95.5	94.1	94.9	94.9	95.1	94.4	92.9	92.9	90.6	90.6	90.7	88.2	90.8
	93.3	93.1	93.8	93.7	93.8	93.6	93.7	94.1	91.9	93.9	92.0	92.0	94.0	96.9	97.0	96.9	95.1	95.8	96.9	96.3	96.7	100.0	94.9	96.1	96.6	96.4	96.5	93.9	93.5	94.1	94.1	94.1	94.0	92.7	92.7	90.8	90.8	90.9	88.9	91.2
***Quinella* candidate species 1**	92.6	92.4	93.3	93.1	93.1	93.0	93.1	93.4	92.2	92.7	92.8	92.4	93.0	94.9	95.0	94.9	94.0	94.6	95.1	94.8	95.1	94.9	100.0	97.8	98.2	98.0	97.9	93.8	92.7	93.4	93.4	93.6	93.9	92.7	92.7	88.9	88.9	88.9	88.5	89.6
	94.0	93.7	94.7	94.4	94.3	94.2	94.2	94.6	93.6	94.1	94.2	93.7	94.2	96.4	96.4	96.2	95.4	96.0	96.6	95.7	96.5	96.1	97.8	100.0	99.6	99.5	99.4	95.2	94.1	94.6	94.7	94.9	95.1	93.9	93.9	90.3	90.3	90.3	88.9	91.0
	94.4	94.0	95.1	94.9	94.8	94.7	94.7	95.1	94.0	94.5	94.6	94.1	94.6	96.8	96.9	96.6	95.8	96.4	97.0	96.5	96.9	96.6	98.2	99.6	100.0	99.9	99.8	95.6	94.5	95.1	95.1	95.4	95.6	94.4	94.4	90.7	90.7	90.7	89.3	91.4
	94.2	93.9	94.9	94.7	94.7	94.5	94.6	94.9	93.9	94.4	94.4	93.9	94.5	96.6	96.7	96.5	95.7	96.3	96.9	96.4	96.8	96.4	98.0	99.5	99.9	100.0	99.7	95.5	94.4	94.9	95.0	95.3	95.5	94.3	94.3	90.6	90.6	90.6	89.2	91.3
	94.2	93.9	95.0	94.9	94.9	94.7	94.8	95.1	93.8	94.5	94.4	93.9	94.5	96.7	96.8	96.6	95.8	96.4	96.9	96.4	96.9	96.5	97.9	99.4	99.8	99.7	100.0	95.7	94.4	95.0	95.1	95.3	95.5	94.3	94.3	90.6	90.6	90.6	89.2	91.2
***Quinella* candidate species 2**	95.4	94.6	94.7	94.0	94.0	93.8	93.9	94.2	92.7	94.0	92.4	92.0	94.0	95.3	95.3	95.1	93.3	95.3	95.2	95.8	95.5	93.9	93.8	95.2	95.6	95.5	95.7	100.0	96.6	94.5	94.6	94.6	94.9	93.4	93.4	89.7	89.7	89.6	88.6	89.8
	94.2	93.6	93.8	92.6	92.5	92.4	92.6	92.8	94.7	95.6	95.1	95.1	94.3	94.0	94.0	94.0	92.7	94.1	93.9	94.2	94.1	93.5	92.7	94.1	94.5	94.4	94.4	96.6	100.0	93.2	93.3	93.6	93.7	93.5	93.5	88.7	88.7	88.8	88.8	89.0
***Quinella* candidate species 7**	93.8	93.4	93.8	94.2	94.3	94.1	94.2	94.5	93.3	94.1	93.4	93.7	94.2	94.6	94.7	94.6	93.7	95.6	94.7	94.9	94.9	94.1	93.4	94.6	95.1	94.9	95.0	94.5	93.2	100.0	100.0	99.0	98.8	96.4	96.4	89.9	89.9	90.1	88.4	90.3
	93.9	93.5	93.9	94.3	94.3	94.2	94.3	94.6	93.3	94.2	93.5	93.8	94.3	94.7	94.7	94.7	93.8	95.7	94.7	94.9	94.9	94.1	93.4	94.7	95.1	95.0	95.1	94.6	93.3	100.0	100.0	99.0	98.8	96.4	96.4	90.0	90.0	90.1	88.4	90.4
	93.7	93.3	94.1	94.1	94.0	93.9	94.0	94.3	93.8	95.2	94.1	94.3	94.5	94.3	94.3	94.1	93.4	95.3	94.3	94.9	95.1	94.1	93.6	94.9	95.4	95.3	95.3	94.6	93.6	99.0	99.0	100.0	99.0	96.7	96.7	90.3	90.3	90.3	88.7	90.2
	93.3	93.2	93.6	94.3	94.4	94.3	94.3	94.7	93.5	94.7	93.8	93.9	94.2	94.5	94.5	94.5	93.5	95.4	94.8	95.2	94.4	94.0	93.9	95.1	95.6	95.5	95.5	94.9	93.7	98.8	98.8	99.0	100.0	97.1	97.1	89.9	89.9	89.9	88.6	90.3
***Quinella* candidate species 5**	91.8	91.6	92.5	93.4	93.4	93.3	93.4	93.7	93.3	94.5	93.5	93.7	92.9	92.8	92.9	92.8	91.9	93.7	93.2	93.4	92.9	92.7	92.7	93.9	94.4	94.3	94.3	93.4	93.5	96.4	96.4	96.7	97.1	100.0	100.0	89.9	89.9	90.1	89.2	90.6
	91.8	91.6	92.5	93.4	93.4	93.3	93.4	93.7	93.3	94.5	93.5	93.7	92.9	92.8	92.9	92.8	91.9	93.7	93.2	93.4	92.9	92.7	92.7	93.9	94.4	94.3	94.3	93.4	93.5	96.4	96.4	96.7	97.1	100.0	100.0	89.9	89.9	90.1	89.2	90.6
***Selenomonadaceae* candidate genus 1 candidate species 1**	89.4	89.3	89.8	90.8	90.7	90.6	90.7	90.6	89.3	89.5	89.0	89.5	90.3	90.1	90.1	90.1	89.2	89.6	90.2	90.8	90.6	90.8	88.9	90.3	90.7	90.6	90.6	89.7	88.7	89.9	90.0	90.3	89.9	89.9	89.9	100.0	100.0	98.4	92.6	95.0
	89.4	89.3	89.8	90.8	90.7	90.6	90.7	90.6	89.3	89.5	89.0	89.5	90.3	90.1	90.1	90.1	89.2	89.6	90.2	90.8	90.6	90.8	88.9	90.3	90.7	90.6	90.6	89.7	88.7	89.9	90.0	90.3	89.9	89.9	89.9	100.0	100.0	98.4	92.6	95.0
	89.6	89.3	89.8	91.0	90.9	90.8	90.9	90.8	89.4	89.6	89.1	89.6	90.4	90.3	90.3	90.3	89.5	89.8	90.4	91.1	90.7	90.9	88.9	90.3	90.7	90.6	90.6	89.6	88.8	90.1	90.1	90.3	89.9	90.1	90.1	98.4	98.4	100.0	92.4	96.0
***Selenomonadaceae* candidate genus 1 candidate species 2**	87.2	87.3	87.5	88.0	87.9	87.8	87.9	87.9	88.5	89.7	88.7	89.0	87.9	88.6	88.6	88.6	87.9	88.5	88.6	88.8	88.2	88.9	88.5	88.9	89.3	89.2	89.2	88.6	88.8	88.4	88.4	88.7	88.6	89.2	89.2	92.6	92.6	92.4	100.0	93.9
	89.4	88.8	89.7	91.0	90.9	90.7	90.9	90.9	88.7	90.2	88.8	89.1	89.2	90.7	90.8	90.7	90.0	90.3	91.0	91.0	90.8	91.2	89.6	91.0	91.4	91.3	91.2	89.8	89.0	90.3	90.4	90.2	90.3	90.6	90.6	95.0	95.0	96.0	93.9	100.0

Supplementary Figure 1. Sequence identities between 16S rRNA gene sequences from *Quinella* and *Selenomonadaceae* candidate genus 1. The sequences are those in the clusters shown in the refined phylogenetic tree of *Quinella* spp., excluding singletons (Figure 1). Each row (and column) represents one sequence, and the sequence accessions are given in the second row. The sequence accession numbers are shown in the second row and are also in the same order down the table. The 22 new sequences generated in this study have accession numbers starting with MF. Blue shaded cells show sequence identities of 93.0 to 96.9% (potential genus level clusters) whereas red shaded cells represented sequence identities of ≥ 97.0%.

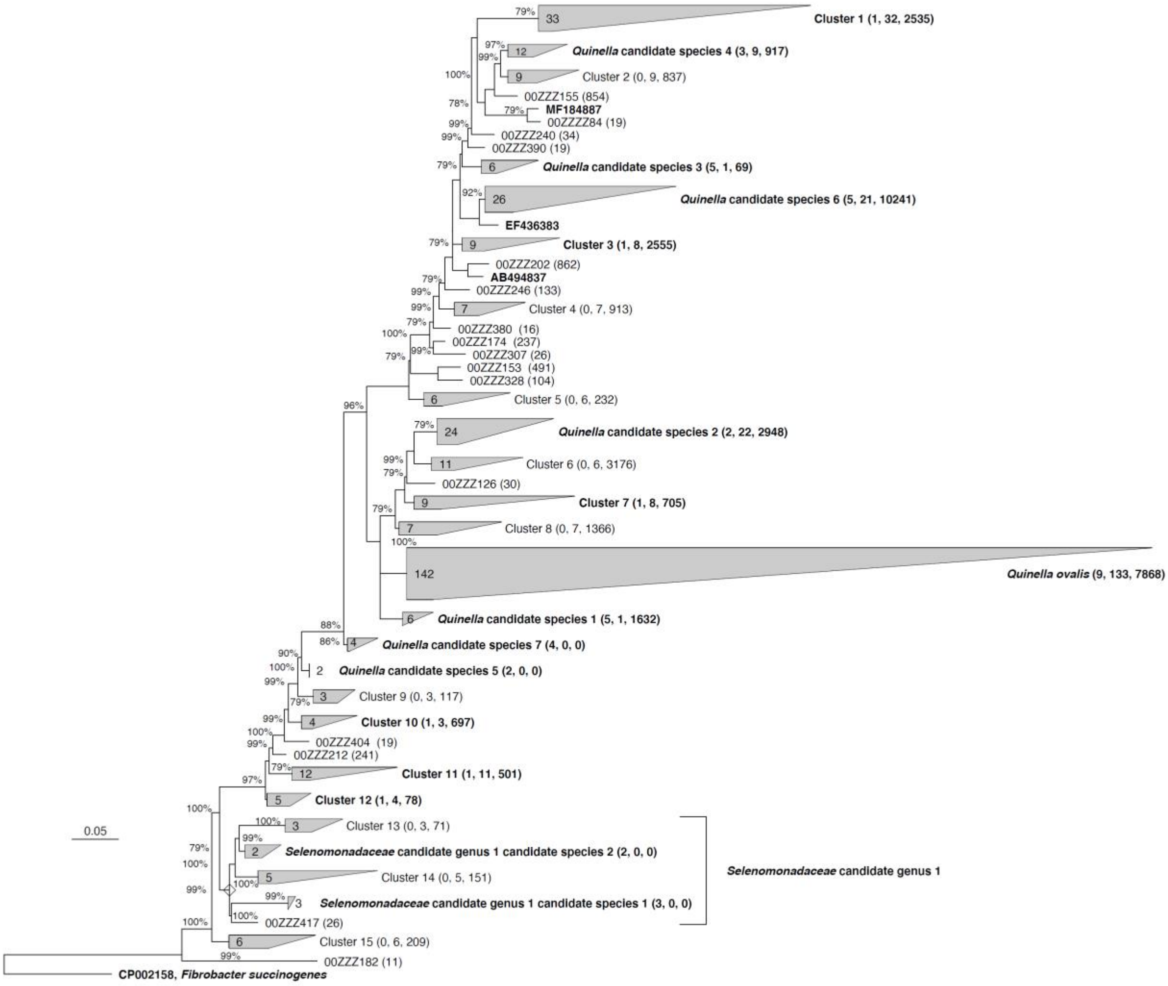

Supplementary Figure 2. Phylogenetic distribution of repset sequences within the radiation of *Quinella*. Repset OTUs with >10 sequences from the study of Kittelmann et al.[4] were added to the refined tree shown in Figure 1 using the ARB parsimony (quick add marked tool) insertion function[7]. Sequences or clusters labelled in non-bold font contain only repset sequences. Numbers in parentheses next to the cluster name show, in this order, the total number of long-length sequences from the refined tree (Figure 1), the total number of repset sequences (i.e., OTUs), and the total number of pyrosequencing reads in those OTUs. The 16S rRNA gene sequence of *Fibrobacter succinogenes* (FibSuc43, GenBank accession CP002158 FSU_1948 (rrsB)) was used as an outgroup sequence. The scale bar represents 0.05 changes per nucleotide position. The numbers at the nodes are the percentage of trees that conserved that node in 1000 bootstrap resamplings.

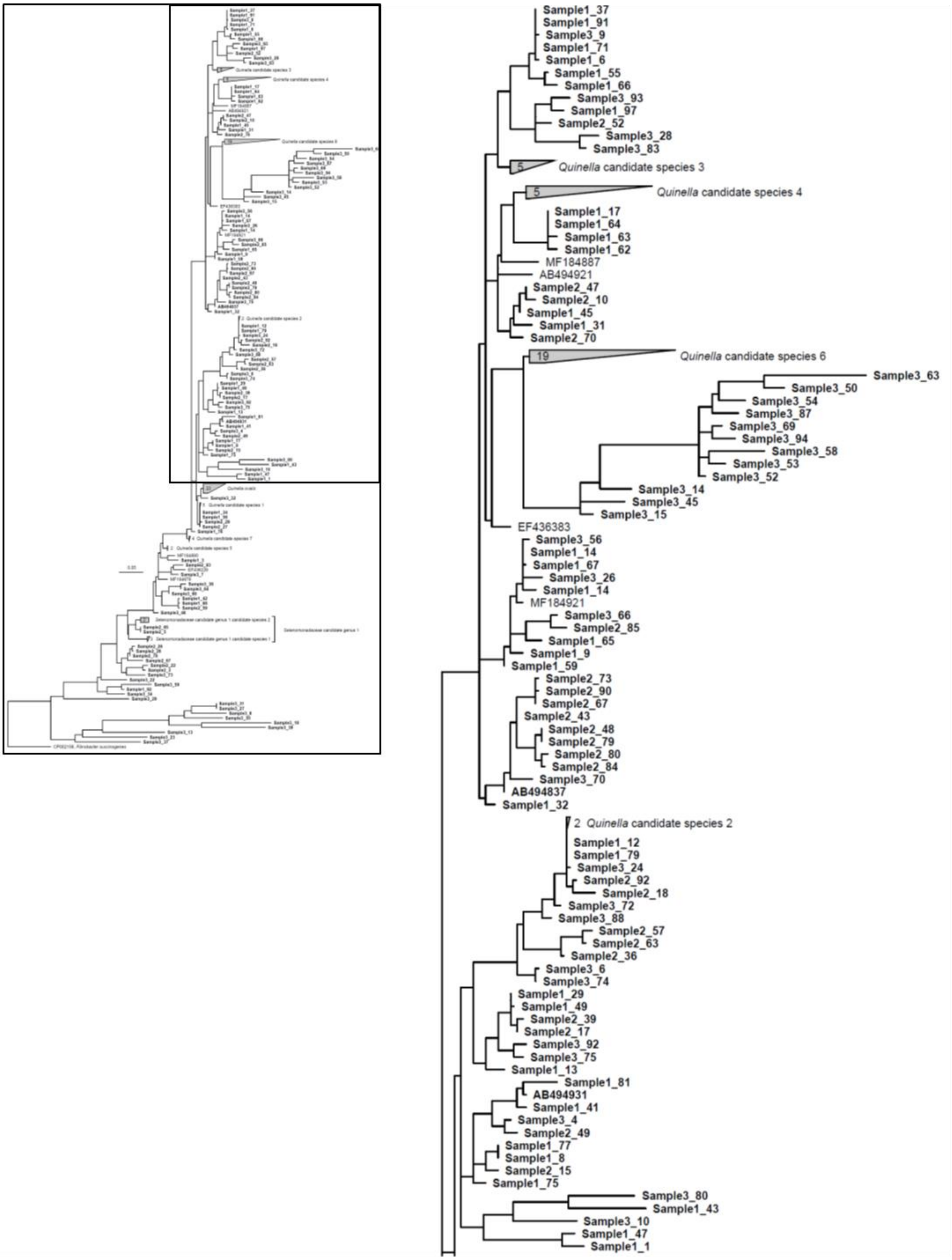
Sample1_37
Sample1_91
Sample3_9
Sample1_71
Sample1_6
Sample1_55
Sample1_66
Sample3_93
Sample1_97
Sample2_52
Sample3_28
Sample3_83
5 Quinella candidate species 3
5 Quinella candidate species 4
Sample1_17
Sample1_64
Sample1_63
Sample1_62
MF184887
AB494921
Sample2_47
Sample2_10
Sample1_45
Sample1_31
Sample2_70
19 Quinella candidate species 6
Sample3_63
Sample3_50
Sample3_54
Sample3_87
Sample3_69
Sample3_94
Sample3_58
Sample3_53
Sample3_52
Sample3_14
Sample3_45
Sample3_15
EF436383
Sample3_56
Sample1_14
Sample1_67
Sample3_26
Sample1_14
MF184921
Sample3_66
Sample2_85
Sample1_65
Sample1_9
Sample1_59
Sample2_73
Sample2_90
Sample2_67
Sample2_43
Sample2_48
Sample2_79
Sample2_80
Sample2_84
Sample3_70
AB494837
Sample1_32
2 Quinella candidate species 2
Sample1_12
Sample1_79
Sample3_24
Sample2_92
Sample2_18
Sample3_72
Sample3_88
Sample2_57
Sample2_63
Sample2_36
Sample3_6
Sample3_74
Sample1_29
Sample1_49
Sample2_39
Sample2_17
Sample3_92
Sample3_75
Sample1_13
Sample1_81
AB494931
Sample1_41
Sample3_4
Sample2_49
Sample1_77
Sample1_8
Sample2_15
Sample1_75
Sample3_80
Sample1_43
Sample3_10
Sample1_47
Sample1_1

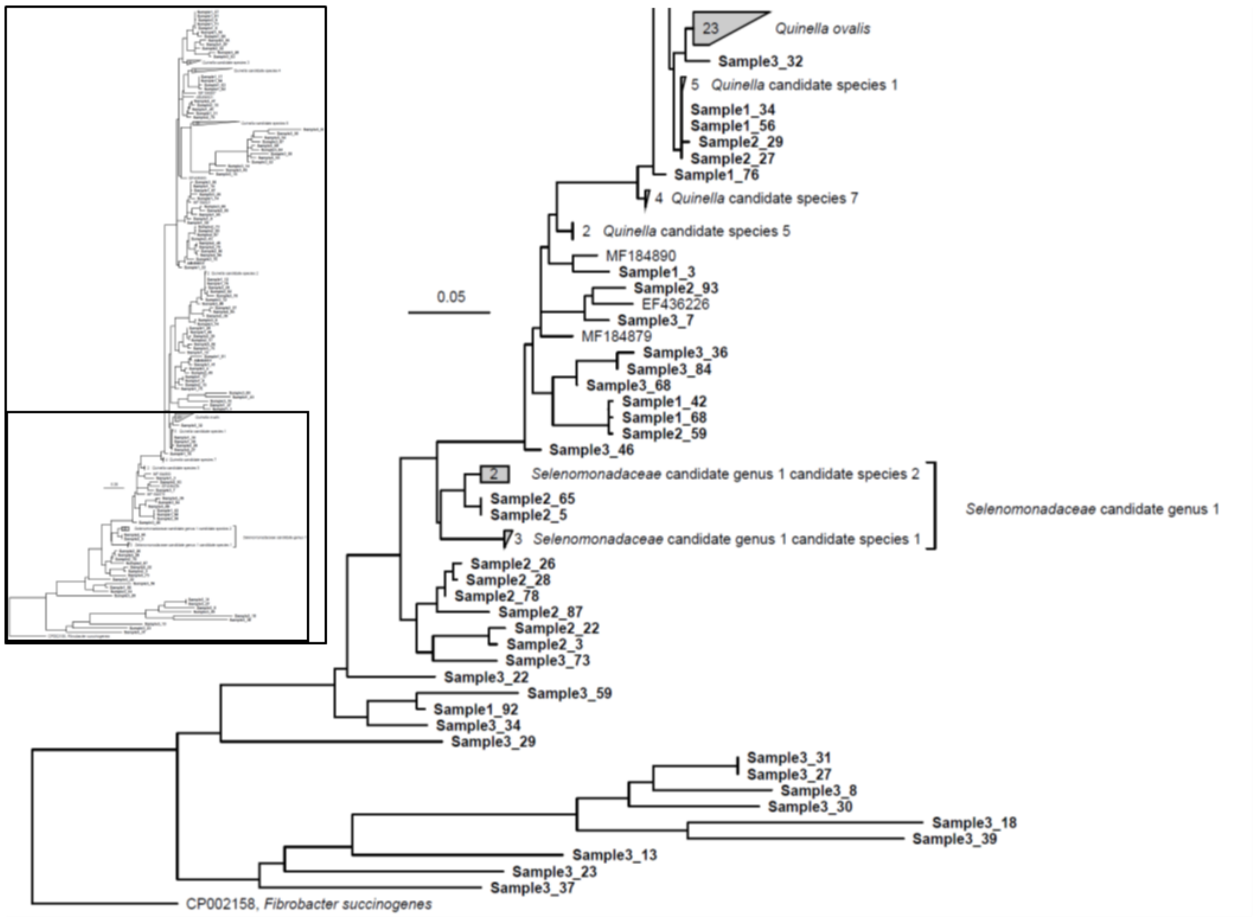

Supplementary Figure 3. Phylogenetic tree of 16S rRNA gene sequences from concentrated samples enriched for *Quinella* cells and affiliated with *Quinella*. The tree is split into two parts (see an overview of the full tree on the top left of each panel). Sequence identifiers in bold letters represent 16S rRNA gene sequences from the different samples of concentrated *Quinella* cells (Table 1). Numbers inside the collapsed clusters represent the total number of sequences in a particular cluster. The 16S rRNA gene sequence of *Fibrobacter succinogenes* (FibSuc43, GenBank accession CP002158 FSU_1948 (rrsB)) was used as an outgroup sequence. The scale bar represents 0.05 changes per nucleotide position.

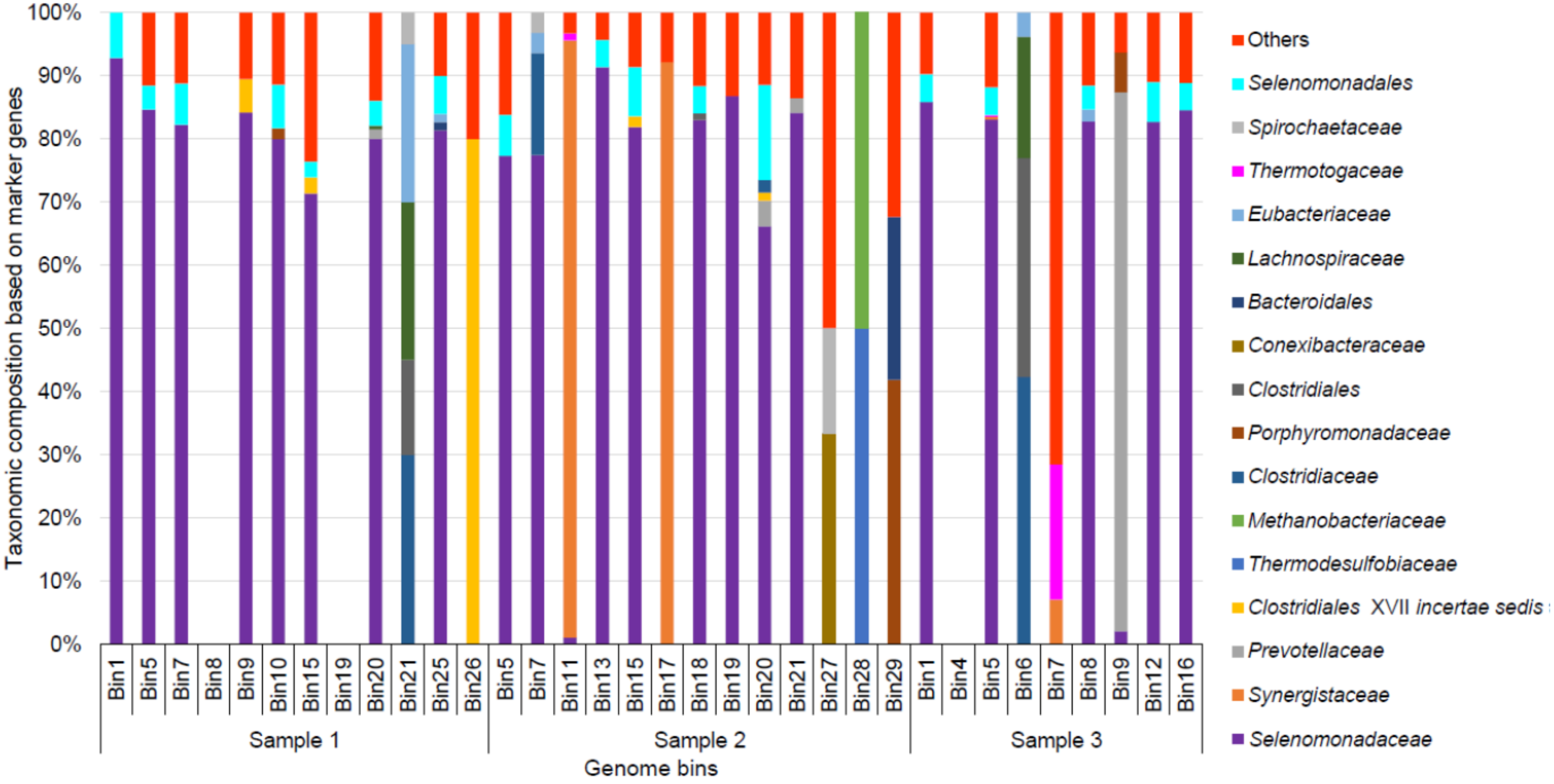

Supplementary Figure 4. Phylotyping of genomic bins. Taxonomic composition of genomic bins was assessed using the AmphoraNet web-based server which used 31 bacterial and 104 archaeal protein coding marker genes for genomic phylotyping[114]. Bin8 and Bin19 from sample 1 and Bin4 from sample 3 were not able to be assigned any taxonomic identities.

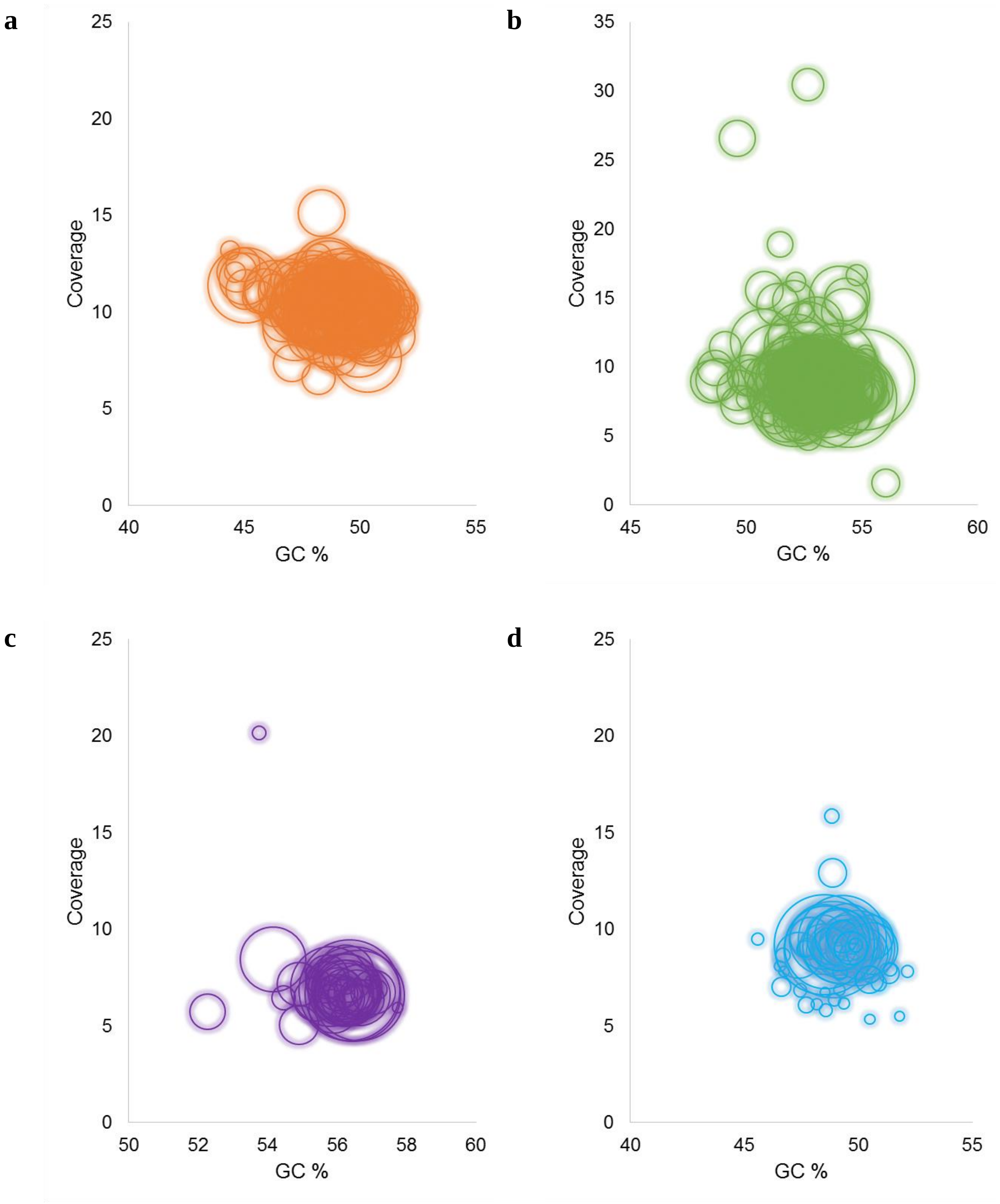

Supplementary Figure 5. Comparison of *Quinella* genome bins. (a) SR1Q5, (b) SR1Q7, (c) SR2Q5, (d) SR3Q1. G+C content (mol%; x-axis), coverage (y-axis), and relative contig lengths (size of the circles) are shown. The coverage is the k-mer coverage (read k-mers per contig k-mer) for the largest k value calculated by SPAdes[115], which was 127 in this case.

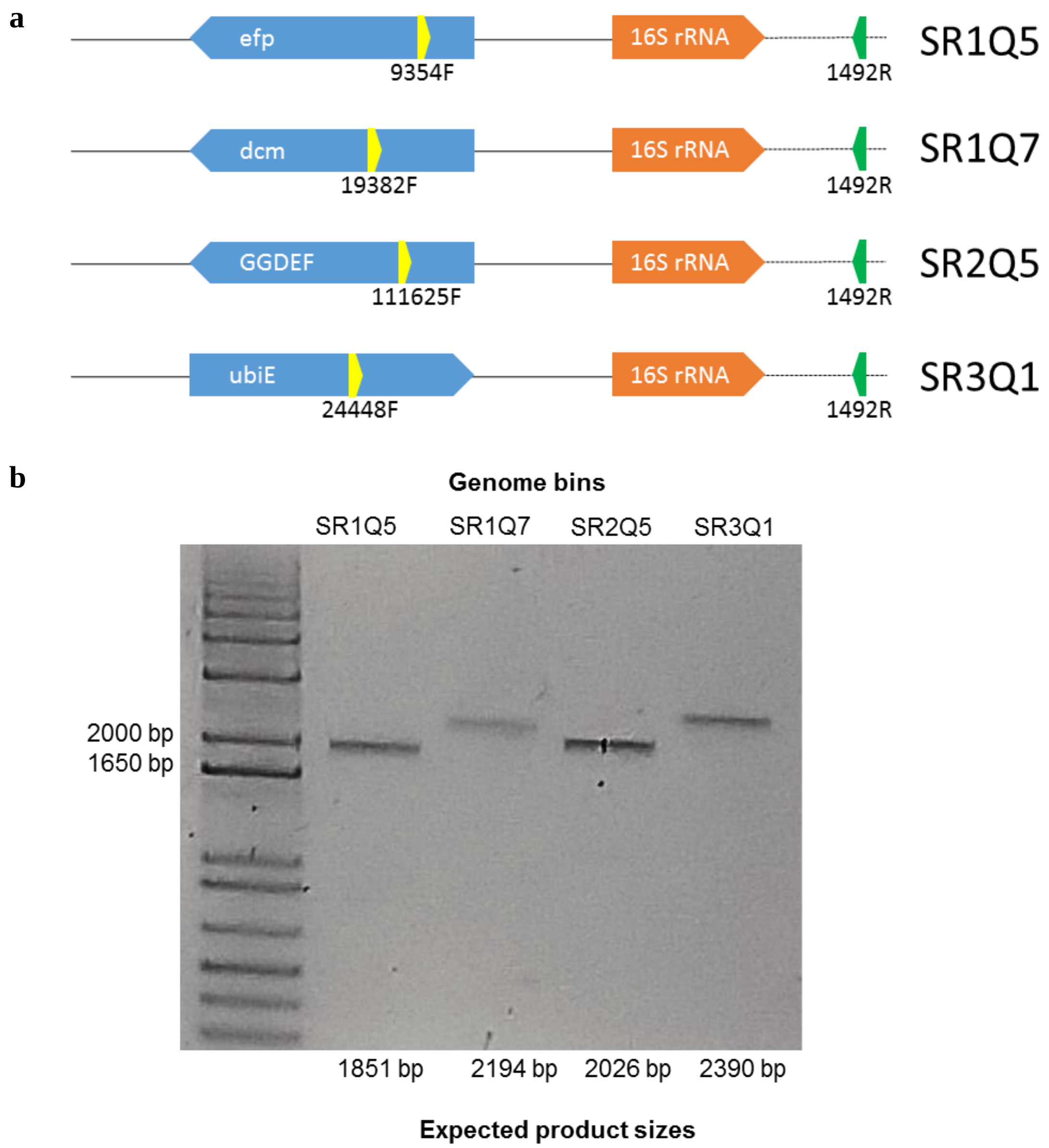

Supplementary Figure 6. Primer targets used to amplify 16S rRNA genes from DNA of *Quinella*-enriched samples. (a) Arrangement of contigs containing 16S rRNA genes and the adjacent genes, showing the regions targeted by the combination of newly designed forward primers (yellow; see Supplementary Table 19) and the universal bacterial primer 1492R (green). Primers 9354F and 19382F were used to amplify products from DNA extracted from sample 1, each in combination with the universal bacterial primer 1492R. Primers 111625F and 24448F were used with DNA from samples 2 and 3 respectively, again each in combination with 1492R. (b) Agarose gel of selected PCR amplified products showing that the products generated using the different primers were of the sizes expected (Supplementary Table 8). The left-hand lane contains a DNA ladder (1 Kb Plus, Invitrogen, Waltham, MA, United States). This experiment was performed only once, but multiple amplicons were sequenced (Supplementary Figure 7).

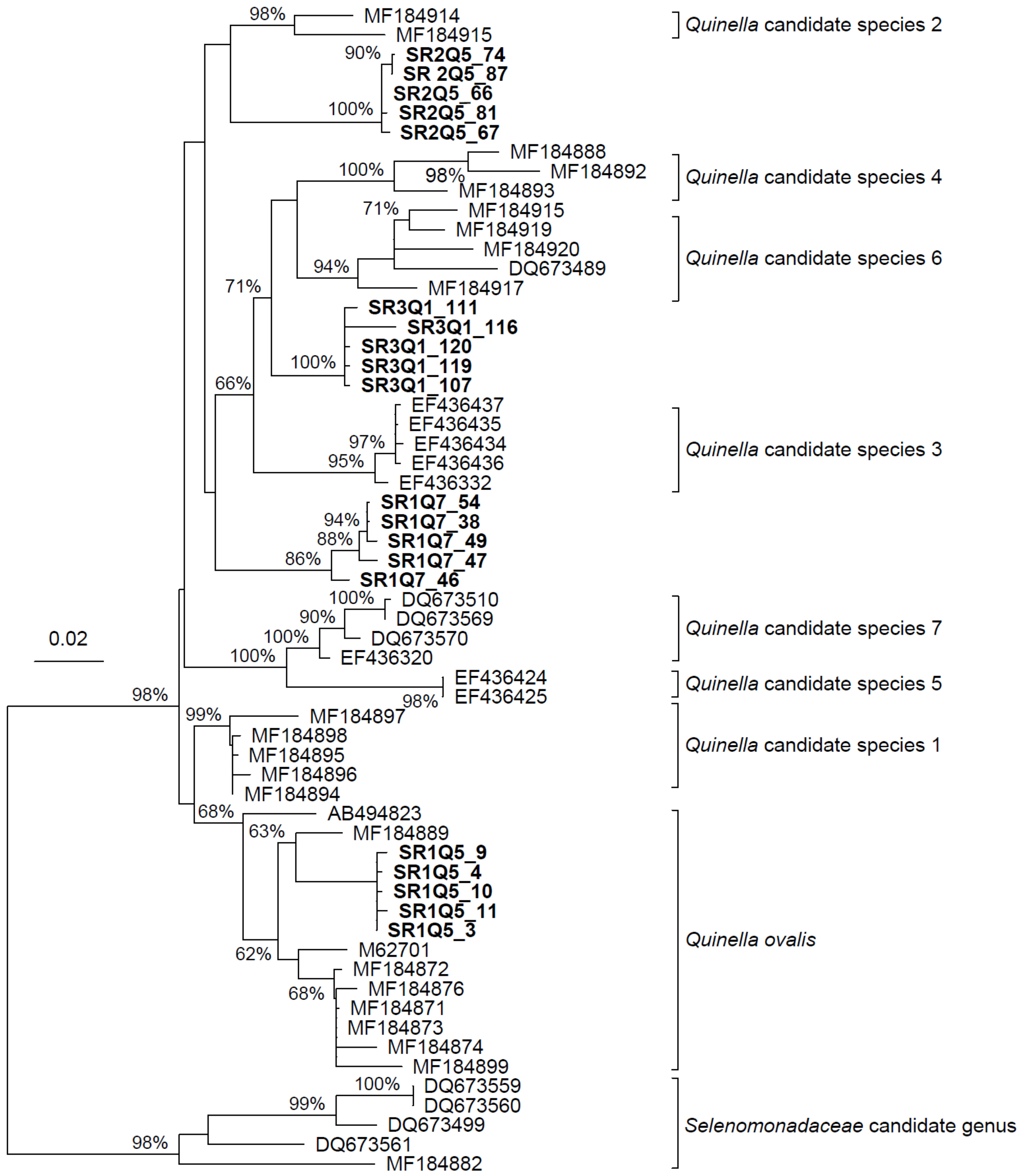

Supplementary Figure 7. Phylogenetic tree of *Quinella* 16S rRNA gene sequences, including those amplified from the DNA samples used to generate the *Quinella* genome bins. The sequences amplified from the samples used to generate the genome bins are shown in bold font. The scale bar represents 0.02 changes per nucleotide position. Bootstrap values of <60% and some of those within defined clusters are not shown. The sequenced 16S rRNA gene plus flanking regions are deposited in GenBank under accessions OM303038 to OM303057.

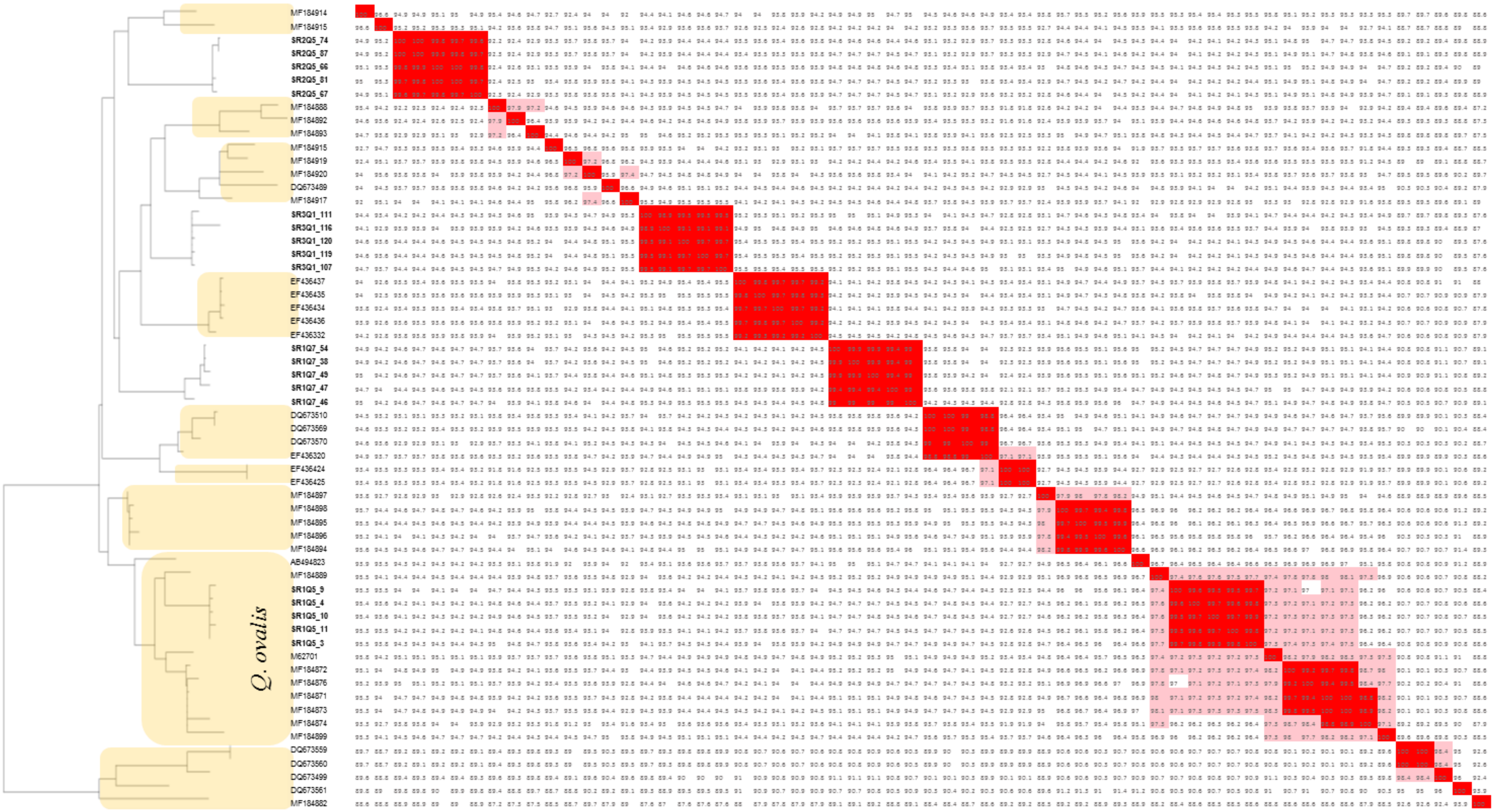
MF184914
MF184915
SR2Q5_74
SR2Q5_87
SR2Q5_66
SR2Q5_81
SR2Q5_67
MF184888
MF184892
MF184893
MF184915
MF184919
MF184920
DQ673489
MF184917
SR3Q1_111
SR3Q1_116
SR3Q1_120
SR3Q1_119
SR3Q1_107
EF436437
EF436435
EF436434
EF436436
EF436332
SR1Q7_54
SR1Q7_38
SR1Q7_49
SR1Q7_47
SR1Q7_46
DQ673510
DQ673569
DQ673570
EF436320
EF436424
EF436425
MF184897
MF184898
MF184895
MF184896
MF184894
AB494823
MF184889
SR1Q5_9
SR1Q5_4
SR1Q5_10
SR1Q5_11
SR1Q5_3
M62701
MF184872
MF184876
MF184871
MF184873
MF184874
MF184899
DQ673559
DQ673560
DQ673499
DQ673561
MF184882
Q. ovalis

Supplementary Figure 8. Sequence similarity matrix of clone library sequences amplified from the DNA samples used to generate the genome bins, and other sequences assigned to *Quinella*. Clone library sequence identifiers are represented in bold letters while similarity values are coloured using two different cut-offs dark red (≥98.7 %) and light red (97% to 98.7%), following the recommendations for delineating species made by Stackebrandt and Goebel [116] and Kim et al.[117] respectively. Sequences defining potential species of *Quinella* are delineated by yellow shapes in the phylogenetic tree on the left, based on the tree shown in Supplementary Figure 7.

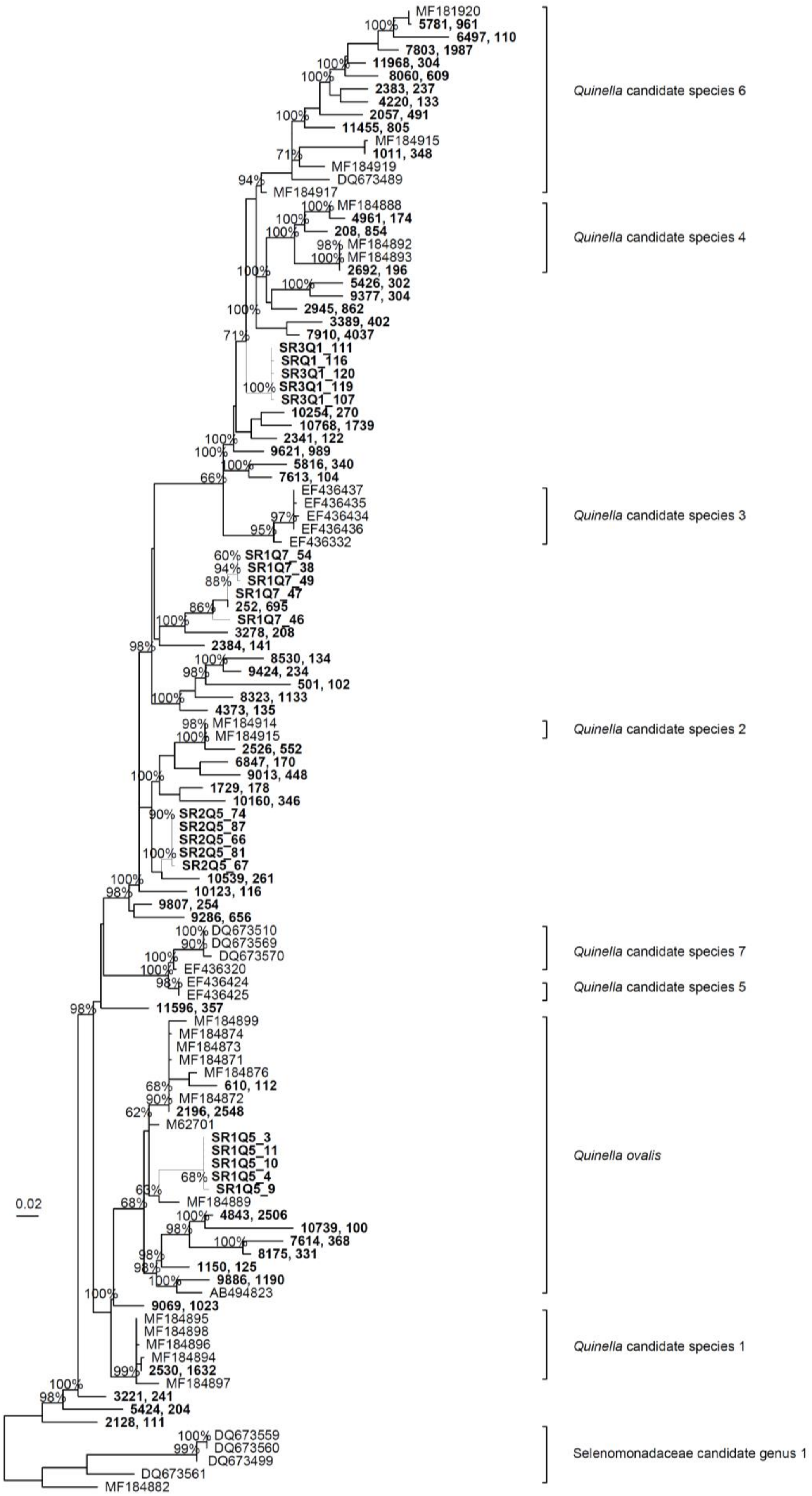
MF181920
5781, 961
6497, 110
7803, 1987
11968, 304
8060, 609
2383, 237
4220, 133
2057, 491
11455, 805
MF184915
1011, 348
MF184919
DQ673489
MF184917
Quinella candidate species 6
MF184888
4961, 174
208, 854
MF184892
MF184893
2692, 196
Quinella candidate species 4
5426, 302
9377, 304
2945, 862
3389, 402
7910, 4037
SR3Q1_111
SRQ1_116
SR3Q1_120
SR3Q1_119
SR3Q1_107
10254, 270
10768, 1739
2341, 122
9621, 989
5816, 340
7613, 104
EF436437
EF436435
EF436434
EF436436
EF436332
Quinella candidate species 3
SR1Q7_54
SR1Q7_38
SR1Q7_49
SR1Q7_47
252, 695
SR1Q7_46
3278, 208
2384, 141
8530, 134
9424, 234
501, 102
8323, 1133
4373, 135
MF184914
MF184915
Quinella candidate species 2
2526, 552
6847, 170
9013, 448
1729, 178
10160, 346
SR2Q5_74
SR2Q5_87
SR2Q5_66
SR2Q5_81
SR2Q5_67
10539, 261
10123, 116
9807, 254
9286, 656
DQ673510
DQ673569
DQ673570
EF436320
EF436424
EF436425
Quinella candidate species 7
Quinella candidate species 5
11596, 357
MF184899
MF184874
MF184873
MF184871
MF184876
610, 112
MF184872
2196, 2548
M62701
SR1Q5_3
SR1Q5_11
SR1Q5_10
SR1Q5_4
SR1Q5_9
MF184889
4843, 2506
10739, 100
7614, 368
8175, 331
1150, 125
9886, 1190
AB494823
9069, 1023
Quinella ovalis
0.02
MF184895
MF184898
MF184896
MF184894
2530, 1632
MF184897
Quinella candidate species 1
3221, 241
5424, 204
2128, 111
DQ673559
DQ673560
DQ673499
DQ673561
MF184882
Selenomonadaceae candidate genus 1

Supplementary Figure 9. Phylogenetic distribution of repset sequences representing abundant OTUs of *Quinella* and 16S rRNA gene sequences amplified from the DNA samples used to generate the *Quinella* genome bins. Both types of sequences are in bold font. Repset sequences are designated by numbers, while those amplified from the DNA used to generate the genome bins are prefixed with SR. Repset sequences representing OTUs with ≥100 sequences were added to the tree (Supplementary Figure 7) using the ARB parsimony (quick add mark tool) insertion function[7]. Numbers after the comma give the total number of pyrosequencing reads in those OTUs. The candidate species are those defined in Figure 1. The scale bar represents 0.02 changes per nucleotide position. Bootstrap values of <60% and some of those within defined clusters are not shown.

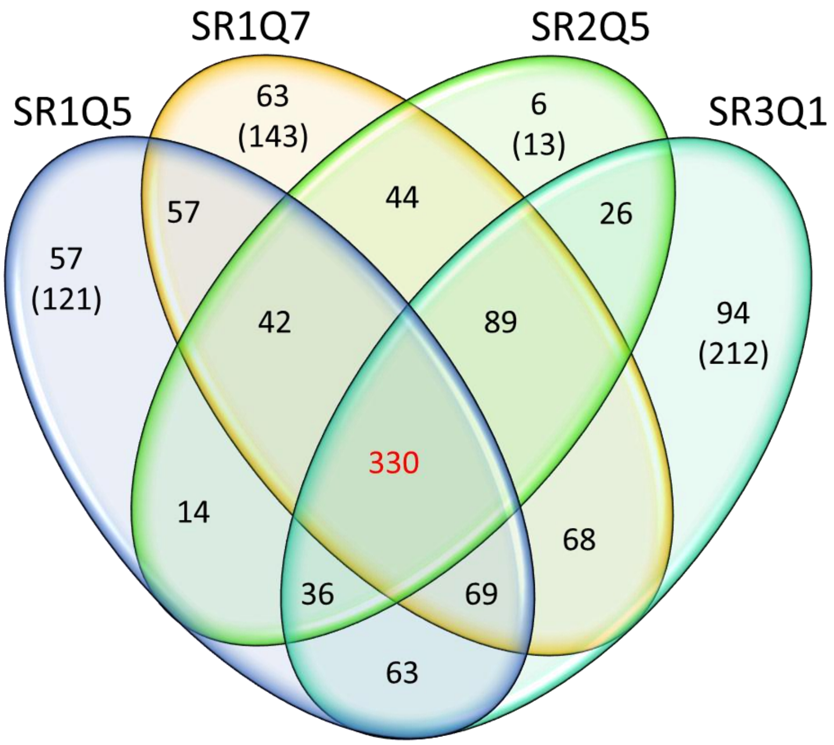

Supplementary Figure 10. Venn diagram of orthologous protein families among all four *Quinella* genome bins. Unoverlapped regions represent unique gene families, with the numbers in the parentheses representing total number of genes present in those families.

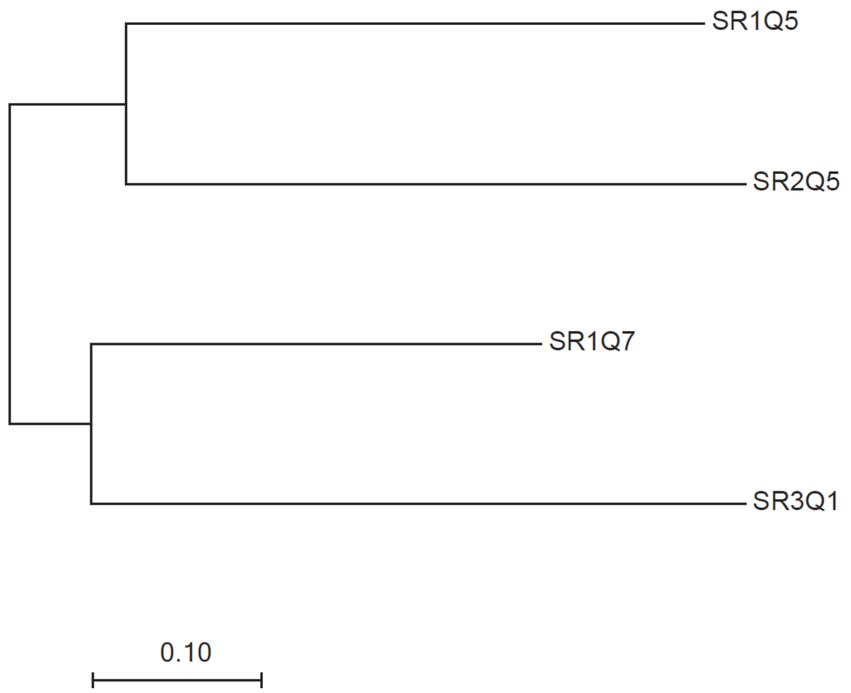

Supplementary Figure 11. Functional genome distribution (FGD) tree of *Quinella* genome bins. The individual bins in FASTA format were concatenated using a universal spacer-stop-spacer sequence and automatically annotated using GAMOLA2[15]. Predicted ORFeomes of all bins were subjected to an FGD analysis[118] and the resulting distance matrix was imported into MEGA7[119].The functional distribution was visualized using the UPGMA method[120]. The tree is drawn to scale, with branch lengths in the same units as those of the functional distances used to infer the distribution tree. The scale bar is a dissimilarity measure between genome bins based on the similarity and absence of ORFs in the genome dataset used to generate the tree[118].

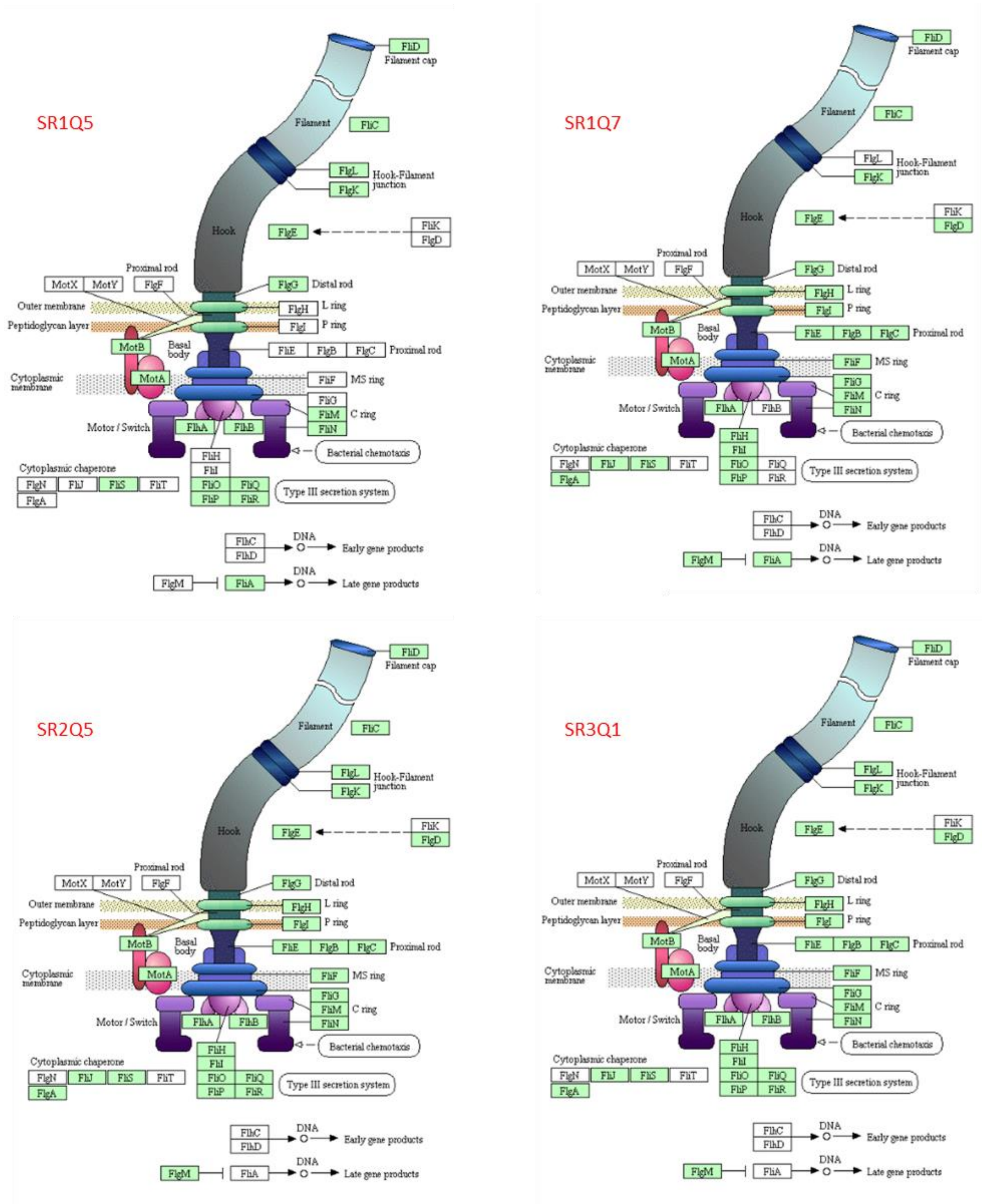

Supplementary Figure 12. Flagellar assemblies inferred by genes detected in *Quinella* genome bins. The proteins likely encoded in the genome bins are highlighted in green boxes with the gene symbol, while those not found are in white boxes. The solid arrows indicate positive regulatory processes, while the line with a short cap on the end indicates a negative regulatory process. The dashed arrow indicates a role in assembly. The model is from the Kyoto Encyclopedia of Genes and Genomes (KEGG)[121]. Figure reproduced with permission from the copyright holder, Kanehisa Laboratories.

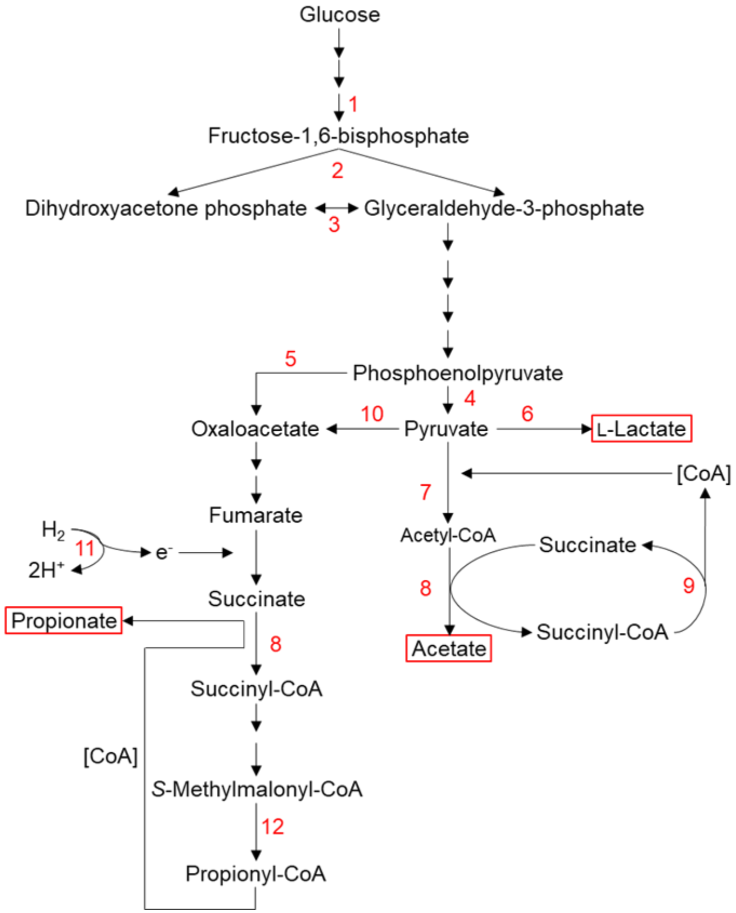

Steps	Enzyme names	E.C. numbers
1	Phosphofructokinase	2.7.1.11
2	Fructose bisphosphate aldolase	4.1.2.13
3	Triose phosphate isomerase	5.3.1.1
4	Pyruvate kinase	2.7.2.3
5	Phosphoenolpyruvate carboxykinase	4.1.1.49
6	L-Lactate dehydrogenase	1.1.1.37
7	Pyruvate:flavodoxin oxidoreductase	1.2.7.1
8	Succinate CoA-transferase	2.8.3.-
9	Succinyl-CoA synthetase	6.2.1.4
10	Oxaloacetate decarboxylase	4.1.1.3
11	NiFe-hydrogenase	1.12.2.1
12	Methylmalonyl-CoA decarboxylase	4.1.1.41

Supplementary Figure 13. Deduced fermentation pathways of *Quinella*. Numbered arrows in the flow chart represent enzymes that are listed in the table together with their Enzyme Commission (E.C.) numbers[122]. The initial steps of glycolysis before phosphofructokinase are not shown.

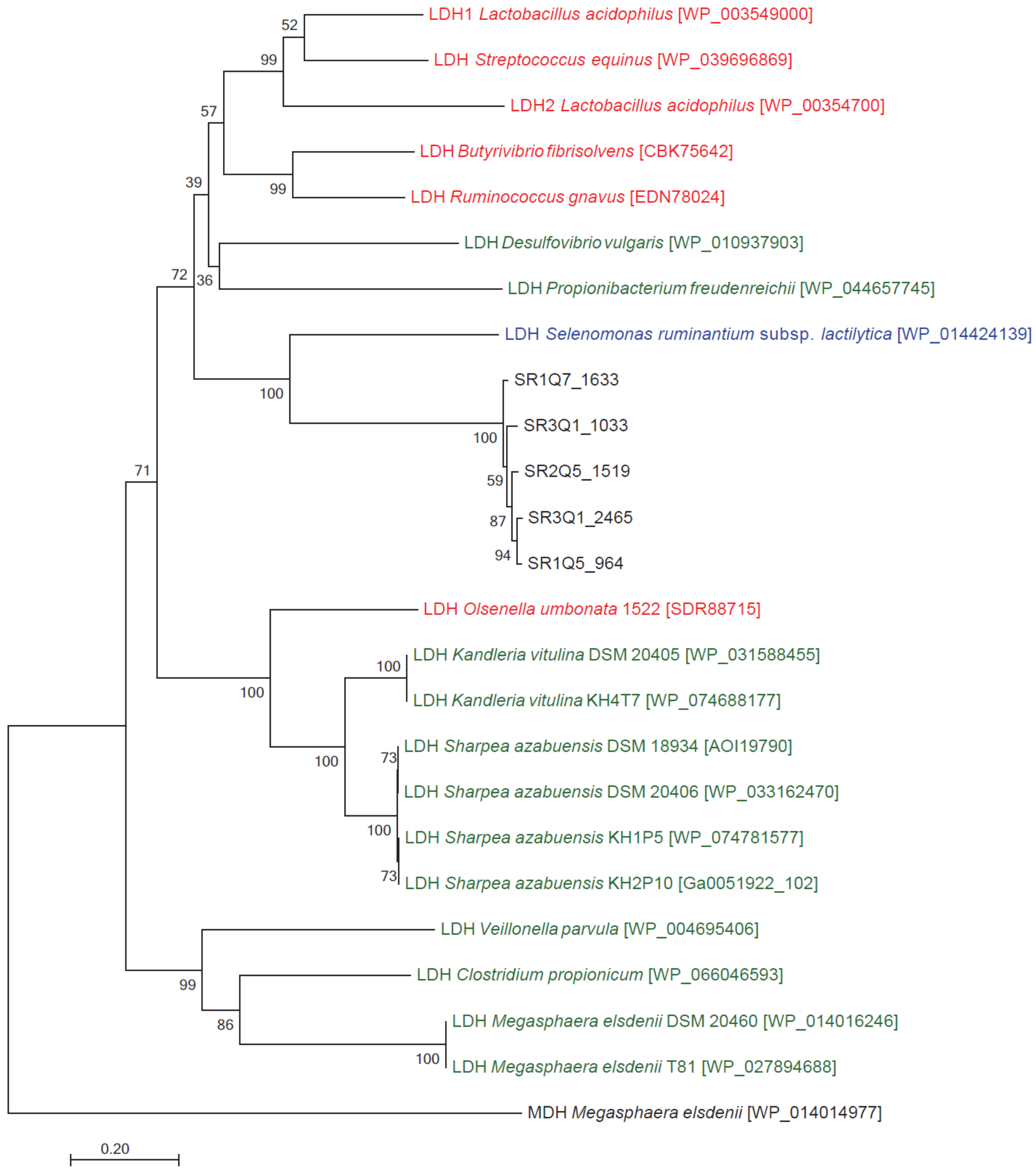

Supplementary Figure 14. Phylogenetic analysis of L-lactate dehydrogenases (LDH) from the *Quinella* genome bins. Malate dehydrogenase (MDH) of *Megasphaera elsdenii* was used as an outgroup sequence. The tree was constructed using the Jukes-Cantor genetic distance model[123] with the Neighbor-Joining method[124] and 500 bootstrap resamplings. LDH, lactate dehydrogenase; MDH, malate dehydrogenase. Lactate producing species are coloured red while lactate utilising species are coloured green. *Selenomonas ruminantium*, coloured blue, can both use and produce lactate. The scale bar represents 0.2 changes per amino acid position. The numbers at the nodes are the percentage of trees that conserved that node in 500 bootstrap resamplings. GenBank accession numbers are given after each sequence name.

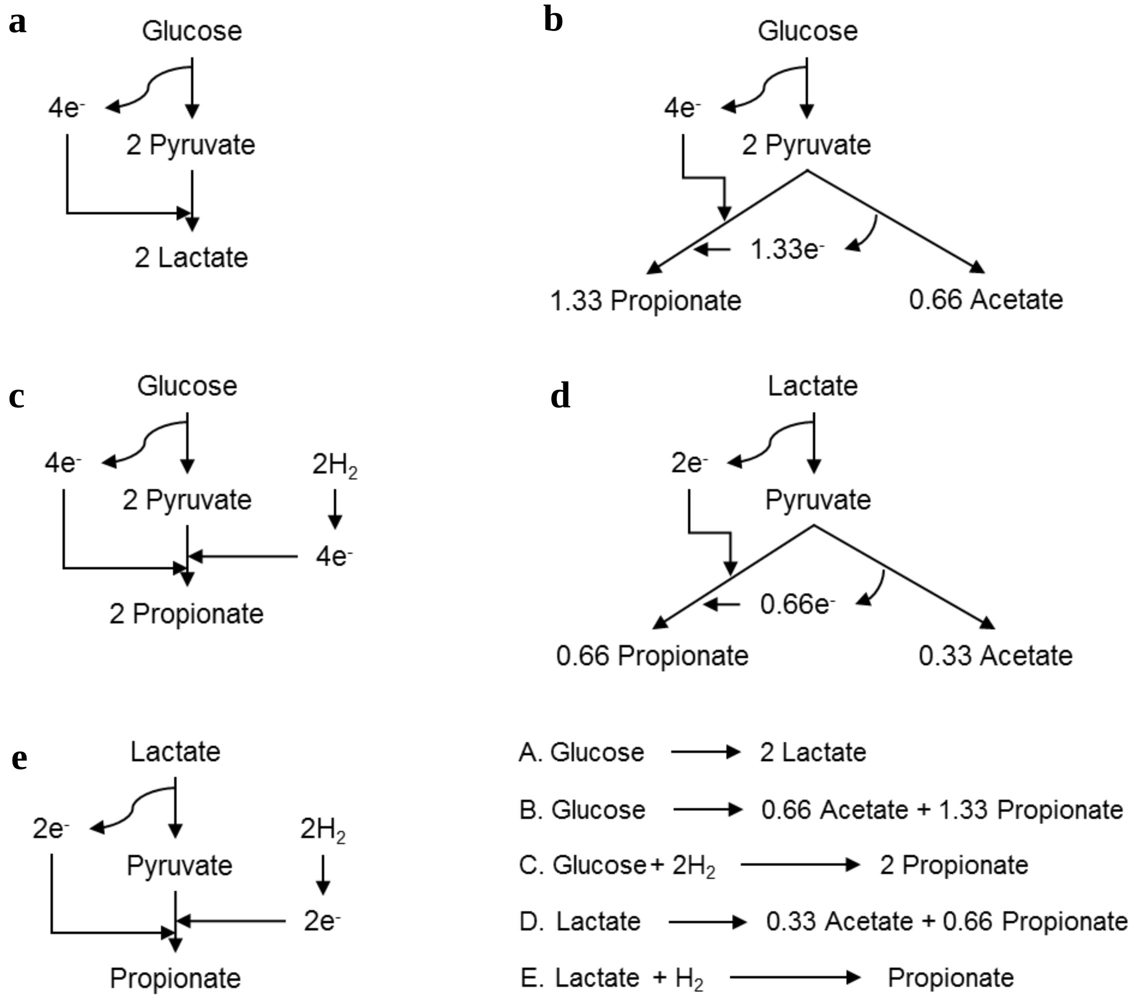

Supplementary Figure 15. Possible end product formation by *Quinella* from glucose and lactate utilisation. There are three possible schemes for using glucose as a substrate. (a) Two lactate molecules can be produced from one glucose. (b) When glucose is fermented without hydrogen uptake, then it will produce 0.66 acetate together with 1.33 propionate. (c) If hydrogen is used via an uptake hydrogenase together with glucose, then two propionate molecules can be produced. There are two pathways for using lactate as a substrate. (d) Lactate can be fermented to 0.33 acetate and 0.66 propionate without hydrogenase activity, while (e) one propionate will be produced if hydrogen is used by the uptake hydrogenase when using lactate. e^- represents electrons transferred by NAD, ferredoxin, cytochrome *b*, or other electron carriers.

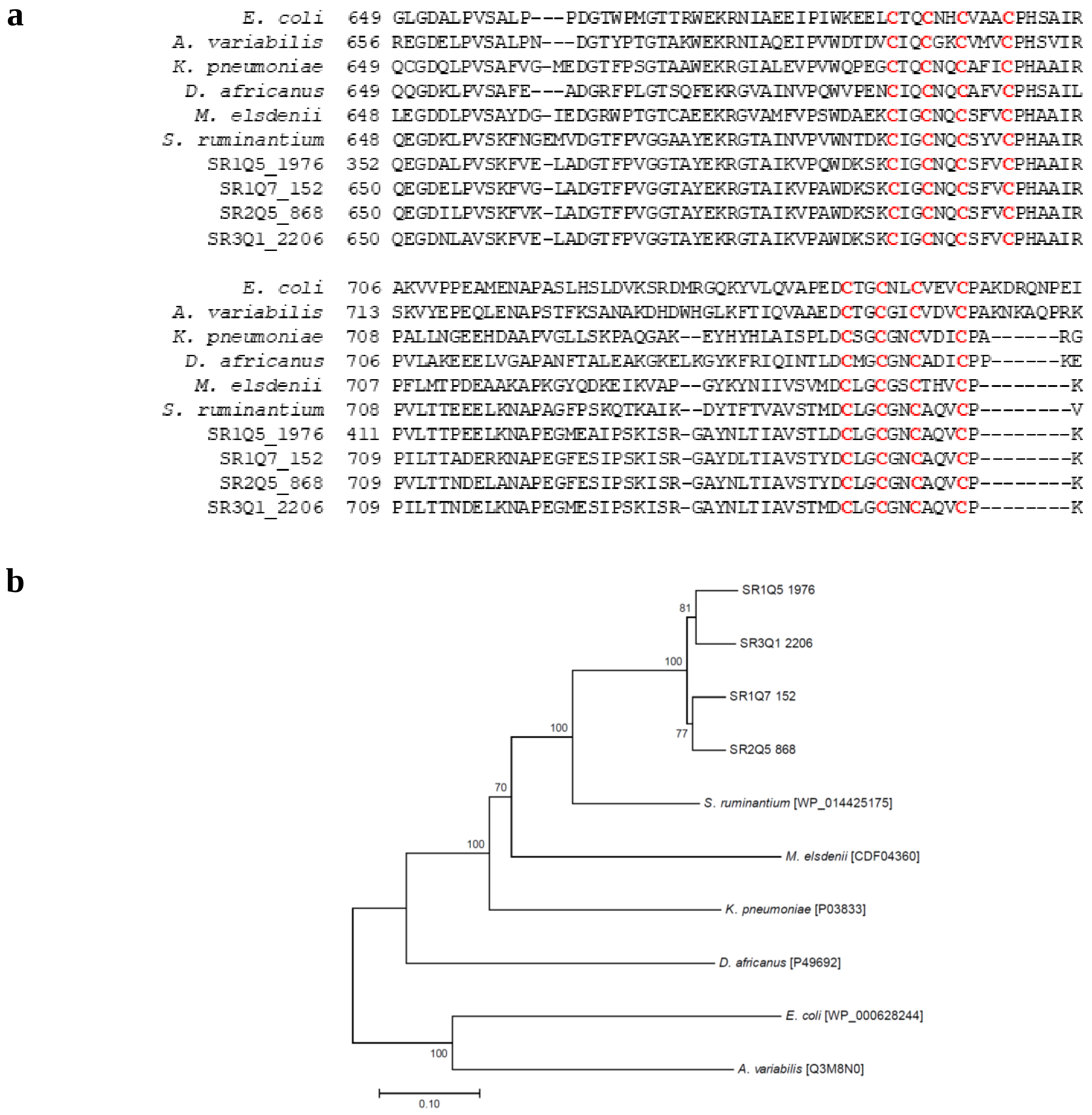

Supplementary Figure 16. Identification of PFOR in the *Quinella* genome bins. (a) Amino acid alignment of *Quinella* PFOR with *Escherichia coli*, *Anabaena variabilis*, *Klebsiella pneumoniae*, *Desulfovibrio africanus*, *Selenomonas ruminantium*, and *Megasphaera elsdenii.* All metal binding cysteine (**C**) residues were found conserved in all analysed sequences. (b) Phylogenetic analysis of PFOR from the *Quinella* genome bins. The tree was constructed using the Jukes-Cantor genetic distance model[123] with the Neighbor-Joining method[124] and 500 bootstrap resamplings. The scale bar represents 0.1 changes per amino acid position. The numbers at the nodes are the percentage of trees that conserved that node in 500 bootstrap resamplings. GenBank accession numbers are given after each sequence name.

```
                                             GxGGxxD
Saccharomyces cerevisiae ACH1   384 NVNGSRMLNGLGGSADFLRNAKLSIMHAPSARPTKVDPTGISTIVPMASHVDQTEHDLDI
      Clostridium kluyveri Cat1   412 HVMGSKMMNGIGGSGDFARNAYLTIFTTESI----AKKGDISSIVPMVSHVDHTEHDVMV
    Selenomonas ruminantium   372 HITGTKMMNGIGGSGDFARNAYLTIFYTPSI----AKGGKISAVVPMCSHIDHTEHDVDI
                  SR3Q1_778   373 HVTGTKMMNGIGGSGDFARNAYLTIFYTPST----AKGGKISSIVPFCSHIDHTEHDVDI
                 SR2Q5_1058   373 HVTGTKMMNGIGGSGDFARNAYLTIFYTPST----AKGGKISSVVPMCSHIDHTEHDVDV
                  SR1Q7_972   373 HVTGTKMMNGIGGSGDFARNAYLTIFYTPST----AKGGKISSVVPFCSHIDHTEHDVDV
                  SR1Q5_482   373 HVTGTKMMNGIGGSGDFARNAYLTIFYTPST----AKGGKISSVVPFCSHIDHTEHDVDI

Saccharomyces cerevisiae ACH1   444 LVTDQGLADLRGLSPKERAREIINKCAHPDYQALLTDYLDRAEHYAKKHNCLHEPHMLKN
      Clostridium kluyveri Cat1   468 IVTEQGVADLRGLSPREKAVAIIENCVHPDYKDMLMEYFEEACKSSGGN-T---PHNLEK
    Selenomonas ruminantium   428 IITEQGIADLRGKAPRERALEIINNCAHPDYRPILLDYFERATEATHHANT---PHILEE
                  SR3Q1_778   429 IISEYGIADLRGLEPRTRALEVINKCAHPDYRPMLLDYYERALAATKKAAT---PHLLHE
                 SR2Q5_1058   429 IISEYGIADLRGLEPRRRALEVINKCAHPDYRPLLLDYYERALAATKKAAT---PHLLNE
                  SR1Q7_972   429 IISEYGIADLRGLEPRTRALEVINKCAHPDYRPMLLDYYERALAATKKAAT---PHLLHE
                  SR1Q5_482   429 IISEYGIADLRGLEPRTRALEVINKCAHPDYRPMLLDYYERALAATKKAAT---PHLLHE
                                       ExG
```

Supplementary Figure 17. Identification of succinate CoA-transferase in the *Quinella* genome bins. Two conserved motifs (ExG and GxGGxxD) of family 1 CoA-transferases[44] were also found in the likely succinate CoA-transferases of *Quinella* and *Selenomonas ruminantium*. Mutation of glutamate (**E**471 in the *Clostridium kluyveri* protein) to aspartate (**D**447 in the *Saccharomyces cerevisiae* protein) results in the conversion of CoA-transferase to CoA-hydrolase[42], and this served as an additional key feature to differentiate between these two enzymes.

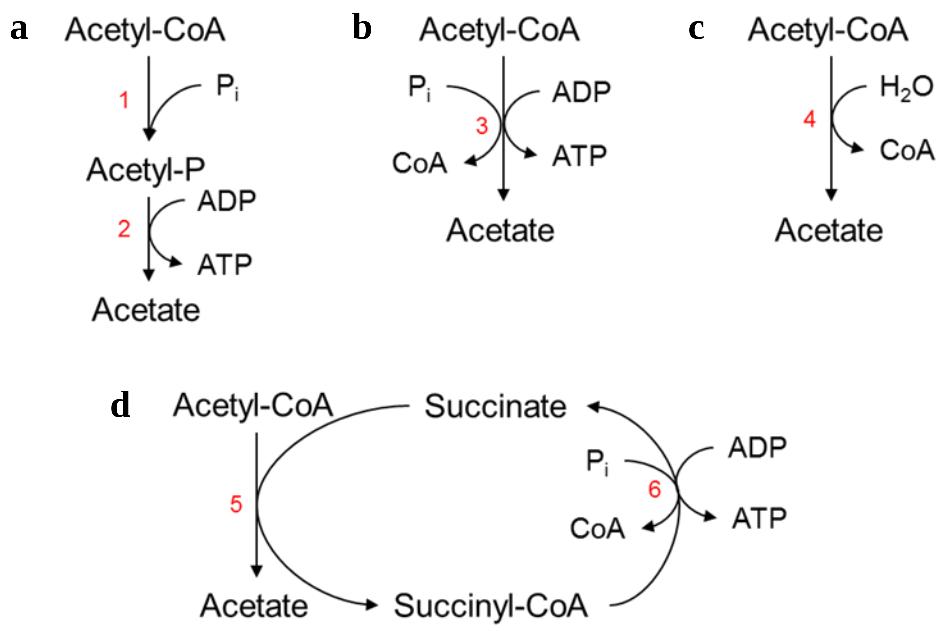

Supplementary Figure 18. Possible acetate formation pathways from acetyl-CoA. Numbered arrows represent enzymes. (a) Two-step conversion of acetyl-CoA to acetate involving (1) phosphate acetyl transferase and (2) acetate kinase. (b) Acetyl-CoA conversion to acetate using (3) acetyl-CoA synthetase. (c) Acetyl-CoA hydrolysis to acetate using (4) acetyl-CoA hydrolase. (d) Acetyl-CoA conversion to acetate using (5) succinate CoA-transferase and (6) succinyl-CoA synthetase.

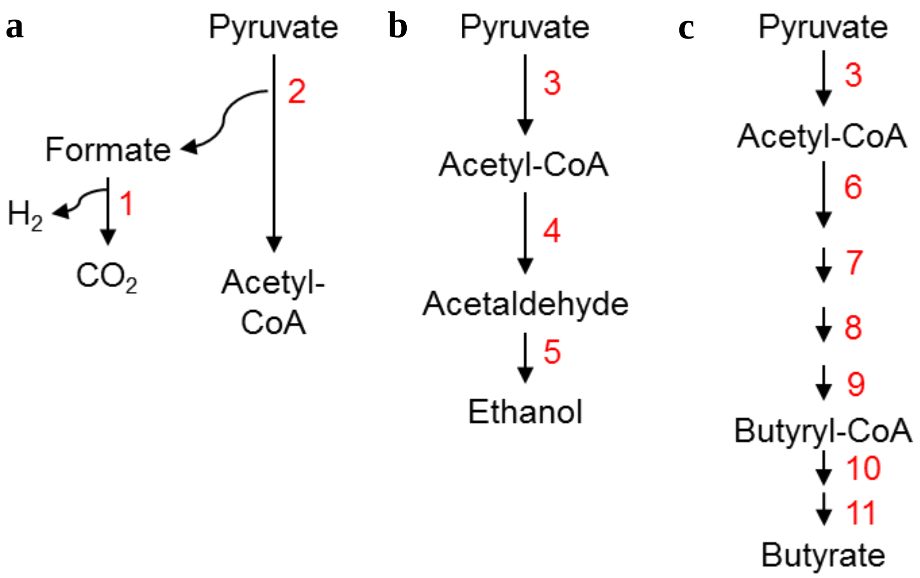

Supplementary Figure 19. Enzymes involved in formate, butyrate and ethanol pathways. Numbered arrows in the flow charts represent enzymes. (a) Formate formation and metabolism: (1) formate dehydrogenase, (2) pyruvate formate lyase. (b) Ethanol formation: (3) pyruvate:ferredoxin (or pyruvate:flavodoxin) oxidoreductase, (4) acetaldehyde dehydrogenase, (5) alcohol dehydrogenase. (c) Butyrate formation: (3) pyruvate:ferredoxin (or pyruvate:flavodoxin) oxidoreductase, (6) acetyl-CoA acetyltransferase, (7) hydroxybutyryl-CoA dehydrogenase, (8) 3-hydroxybutyryl-CoA dehydrogenase, (9) butyryl-CoA dehydrogenase, (10) phosphotransbutyrylase and (11) butyrate kinase.

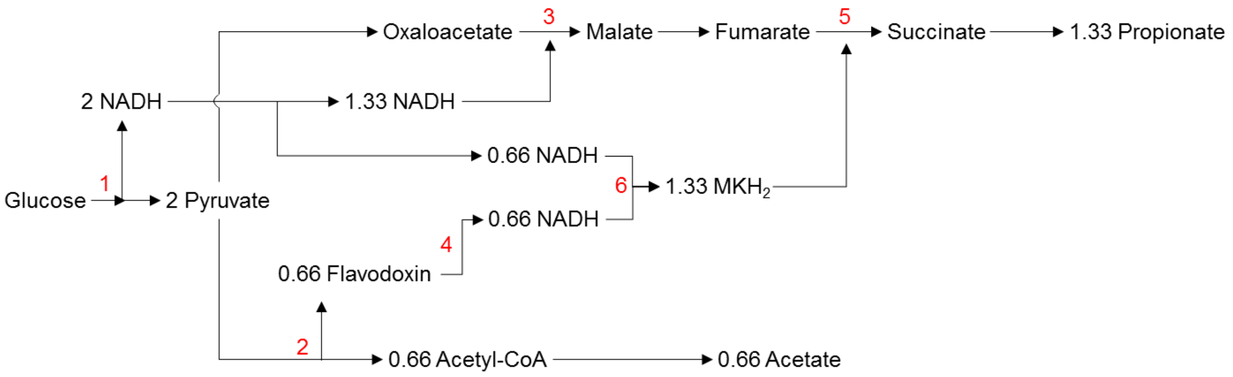

Supplementary Figure 20. Pathway showing possible electron flow from the glycolytic pathway and conversion of pyruvate to acetyl-CoA. Numbered arrows in the flow chart represent enzymes. (1) enzymes involve in glycolytic pathway, (2) PFOR, (3) malate dehydrogenase, (4) NAD(P)H:flavin oxidoreductase, (5) fumarate reductase, (6) NADH:quinone oxidoreductase. An enzyme catalysing step 6 was not found in any of the *Quinella* genome bins.

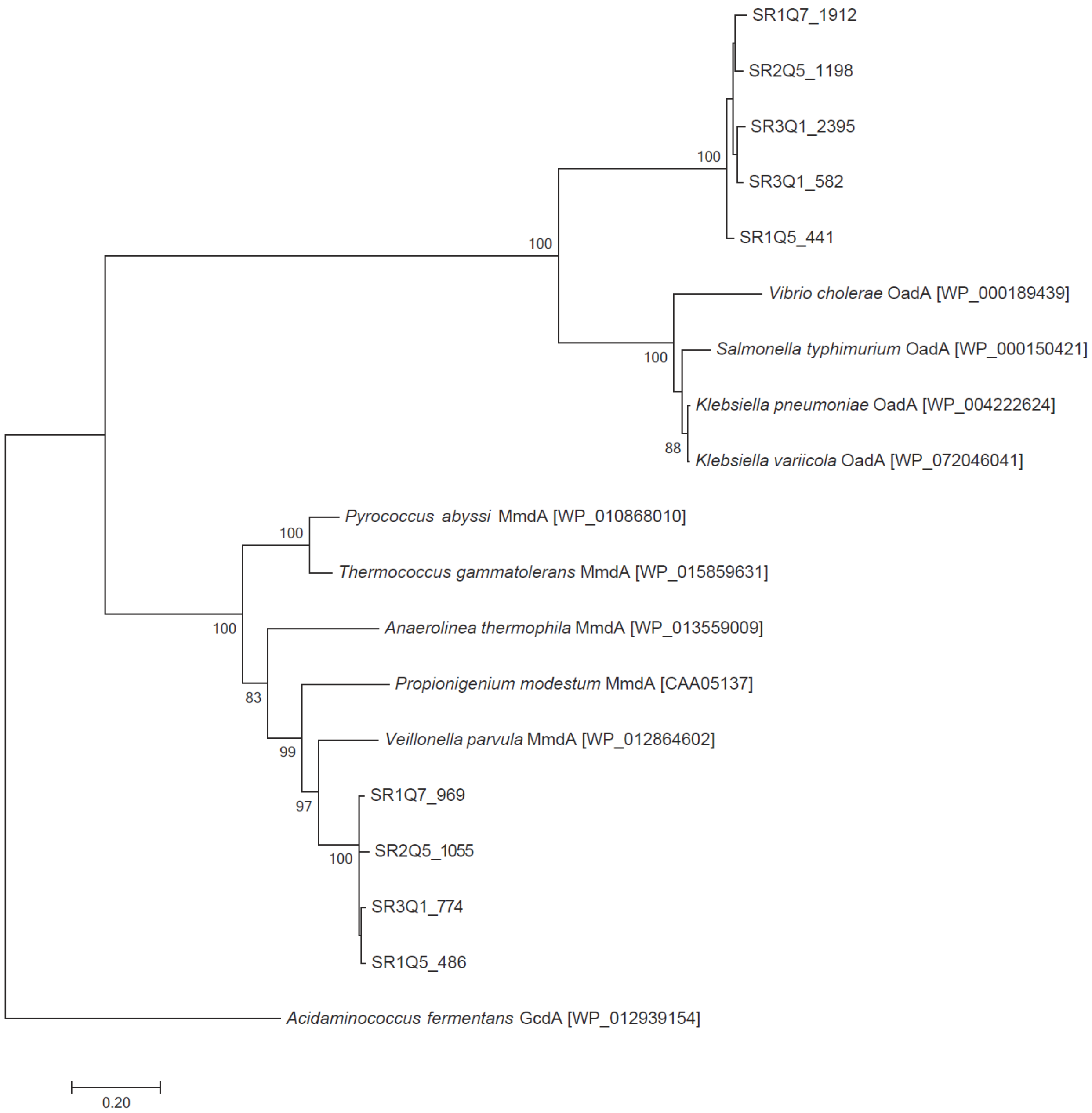

Supplementary Figure 21. Phylogenetic tree of α subunits of MMCD (MmdA) and OACD (OadA), based on amino acid sequences. The *Acidaminococcus fermentans* glutaconyl-CoA decarboxylase α subunit sequence (GcdA) was used as an outgroup sequence. The tree was generated using the Jukes-Cantor genetic distance model[123] with the Neighbor-Joining method[124] and 500 bootstrap resamplings. The scale bar represents 0.2 changes per amino acid position. The numbers at the nodes are the percentage of trees that conserved that node in 500 bootstrap resamplings. GenBank accession numbers are given after each sequence name.

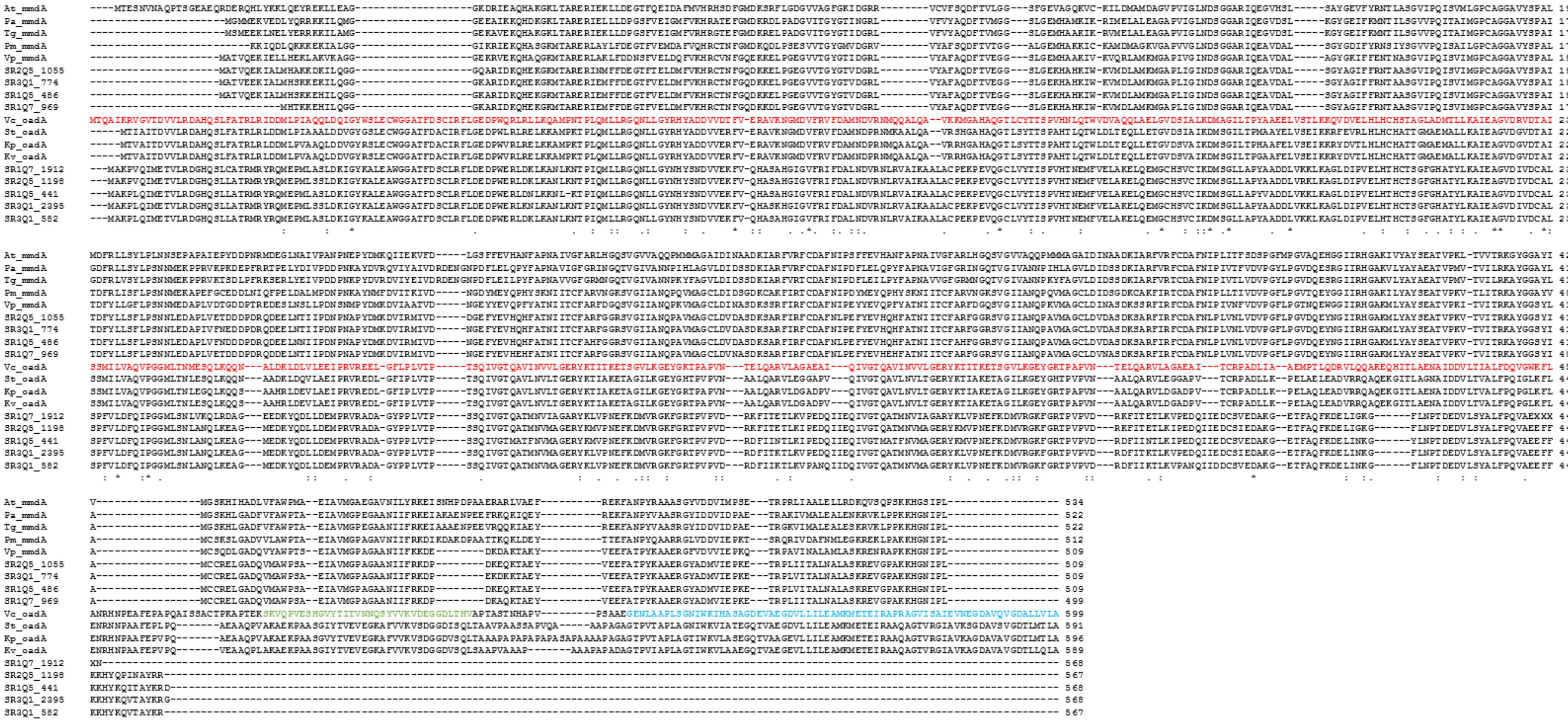

Supplementary Figure 22. Alignment of alpha subunits of MMCD (MmdA) and OACD (OadA) of *Quinella* with reference sequences. The MmdA references are from *Anaerolinea thermophila* (At), *Pyrococcus abyssi* (Pa), *Thermococcus gammatolerans* (Tg), *Propionigenium modestum* (Pm), and *Veillonella parvula* (Vp), and the OadA reference sequences are from *Klebsiella pneumoniae* (Kp), *Vibrio cholerae* (Vc) *Salmonella typhimurium* (St) and *Klebsiella variicola* (Kv). Three domains; *N*-terminus domain (red), association domain (green) and a *C*-terminus domain (light blue) containing a biotin-binding lysine residue (purple) were marked in the *Vibrio cholerae* sequence.

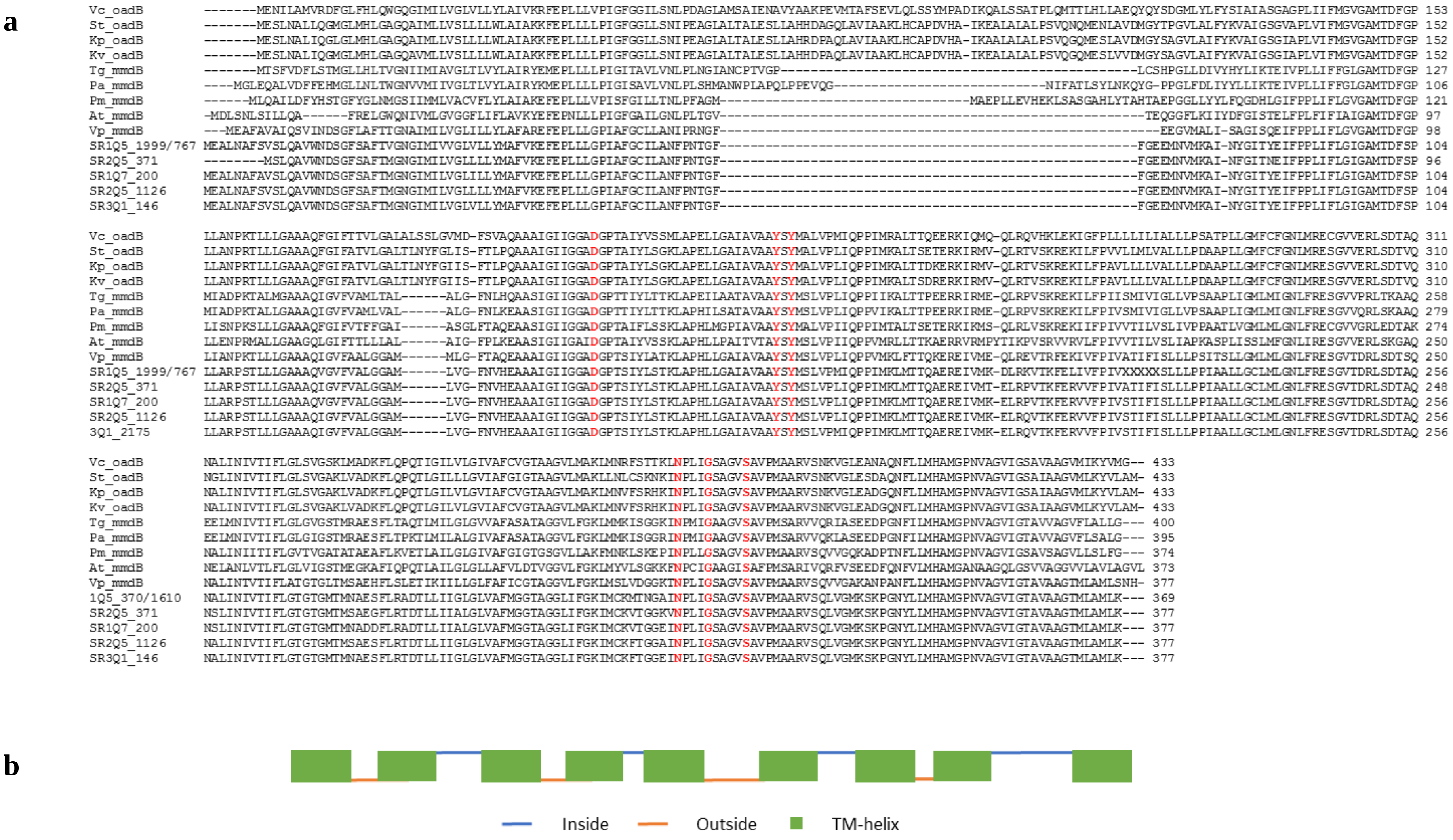
a
b
Inside
Outside
TM-helix

Supplementary Figure 23. Beta subunits of MMCD and OACD. (a) Alignment of beta subunits of MMCD (mmdB) and OACD (oadB) of *Quinella* with reference sequences. Beta subunit amino acid sequences of MMCD and OACD are reported to have >50% identity[60], similar to what was observed here (all aligned sequences are >43% similar to each other). Key amino acids (red) in the reviewed *Klebsiella pneumoniae*[67, 75, 76] OadB were also found to be conserved in *Quinella* MmdB. The reference sequences use the same species name codes as in Supplementary Figure 22).
(b) Schematic representation of the *Quinella* MmdB. Nine transmembrane regions were predicted using SPOCTOPUS[18]. TM, transmembrane.

a

```
St_OadA C terminus EAAQPVAKAEKPAASGIYTVEVEGKAFVVKVSDGGDI--SQLTAAVP-------------AASSAPVQAAAP-----AGAGT-PVTAPLAGNIWKVIATEGQTVAEGDVLLILEAMKMETEIRAAQAGTVRGIAVKSGDAVSVGDTLMTLA 130
Kp_OadA C terminus EAAQPVAKAEKPAASGVYTVEVEGKAFVVKVSDGGDV--SQLTAAAP-------------APAPAPAPASAPAAAAPAGAGT-PVTAPLAGTIWKVLASEGQTVAAGEVLLILEAMKMETEIRAAQAGTVRGIAVKAGDAVAVGDTLMTLA 135
Pm_MmdC            M--KN------------FKVTVNGTEYDVAVEEMGGAAVASAPAARP---AAAPAPAAPKPAAAPAPAPAPKTTA-AGAGAGANTVTAPMPGTILNVGCHAGDKVSKGDTLVVLEAMKMENEIMAPHDGVVSEVRVQQGASVNAGDILVVLS 134
Vp_MmdC            M--KK------------FNVTVNGTAYDVEVNEVKAAAPAAAPKAAP---AAAPAPK---AAPAPAPAAAAAPVP-----AGAETVKAPMPGKILSVAVSAGQAVKKGETLLILEAMKMQNEIAAPHDAVVSEVRVSANQTVSTGDDMVVLG 127
SR1Q7_968          MATKK------------FNVTVNGTTYEVEVEEVKAA--GGAPKAAPAPKAAAPAPKAAAPA-APAPAPKAAAPAKVAAGAGEHSIDSPMPGKIVKLVAAEGAAVKAGDVLLILEAMKMQNEITADADGTVKKFNVAAGQSVKAHESLVILG 137
SR3Q1_773          MATKK------------FNVTVNGTTYEVEVEEVKAA--GGAPKAAP-----APAPKAAAPAPAPKAAPAPAAAPKVAAGAGEHSIDAPMPGKVIKLVAAEGAAVKAGDVLLILEAMKMQNEITADADGTVKKFNVAAGQSVKAHESLVILG 133
SR1Q5_488          MATKK------------FNVTVNGTTYEVEVEEVKAA--GGAPKAAP-----APKVA---PAPAPAPKAAPATAPKVAAGAGEHSIDAPMPGKVIKLVAAEGQAVKAGDVLLILEAMKMQNEITADADGTVKKFNVAAGQSVKAHESLVILG 130
SR2Q5_1055         MATKK------------FNVTVNGTTYEVEVEEVKAA--GSAPKAAP-----APAPK---AAPAPAPKAAPAAAPKVAAGAGEHSIDAPMPGKVVKLVAAEGQAVKAGDVLLILEAMKMQNEITADADGTVKKFNVAAGQSVKAHESMVILG 130
```

b

```
Kp_oadC      M-------------TDNAVLLGEGFT----LMCLGMGFVLVFLLLLIFAIRGMSLA-------VNRLFP---EP------PAAPKPAPAAVAPA-DD------FARLKPAIVAA------------IHH------------------HRRLHP 83
St_oadC      M-------------TNAALLLGEGFT----LMFLGMGFVLAFLFLLIFAIRGMSAA-------VNRFFP---EP------APAPKAAPAAAAPVVDD------FTRLKPVIAAA------------IHH------------------HHRLNA 84
Pm_mmdD      MNITELMELFSNPETIKTLETGDLMTGIGVTVVLGMGITVVALIFLMYIIGGMAAIMAEKPKEVKETAAAAPKPEAAA--------APA---PAANN--DEELVAVIAAAVAAQLGTSASNLIIRNVTRSLDTTPAWGRAGIVDQMATRL--- 137
Vp_mmdD      ME--------GQAVTTNPW----------LIMAINMTVVFAVLIALGILMEIVHLIDPTKKKKE---APAATAPVATPTA------TPVAPANASAQN-EDEVVAAIVGAIVA-MGYSSEQIASIRPTA--TSAK-WRLEGRL---SGRG--- 115
SR1Q7_201    MS--------HSPVTTNPL----------IIMMINMTVVFIVLVVLMYLIKLIHIIDPTKPREVPK-SDDEDLPLVAVKSEPSTATPPPEPEPVT-QGVSPEIIAAISAAIAA-YGFAGQVKAVRIINH--EGTP-WRASARI-NTFSRK--- 125
SR2Q5_370    MG--------HSPVTTNPL----------IIMMINMTVVFIVLVVLMYLIKLIHIVDPTKPKEEPAAAPVDDEPLVAVKSA-----APE---PVLDAGPTPEIIAAISAAITA-YGFVGQVKAVRIINH--EGTP-WRSSARF-NTLKRK--- 119
SR3Q1_145    ME--------HSPVTTNPL----------IIMMINMTVVFIVLVSLMFLIKLIHIVDPTKEPEVKE-APADDEPLVAVKS------EPAAIAPVVEEGIKPEVIAAISAAIAA-YGFAGQVKAVHIINH--EGTP-WSNAMRNAQGVMRNGRR 124
SR1Q5_2000   ME--------HSPVTTNPL----------IIMMINMTVVFIVLIVLMYLIKLIHFLDPTKEPEAKE-APEEEEPLVAVKSATASAPEPV---PVVEEGIKPEVIAAITAAIAA-YGFAGQVKAVHIINH--EGTP-WRAAAKFNNNLQRK--- 124
SR2Q5_1127   ME--------HSPVTTNPL----------IIMLINMTVVFLVLVSLIYLIKLIHFVDPTKEKPKPE--VEEDEPLVAVKSAPAAS-APA---PVVEEGINSEVIAAISAAIAA-YGFAGQVKAVHILNH--EGTP-WRAAAKF-NNFPRK--- 121
```

Supplementary Figure 24. Gamma and delta subunits of MMCD and OACD. (a) Alignment of MmdC of MMCD and the *C*-termini of OadA of OACD. The conserved lysine residue in the LEAMKM motif (red) is implicated in biotin binding[59, 60], and was conserved in the *Quinella* sequences. (b) Alignment of MmdD of MMCD and OadC of OACD. The alanine-proline linker region (purple) in *Klebsiella pneumoniae* (Kp) and *Salmonella typhimurium* (St) was present in MmdC with different degrees of similarity.

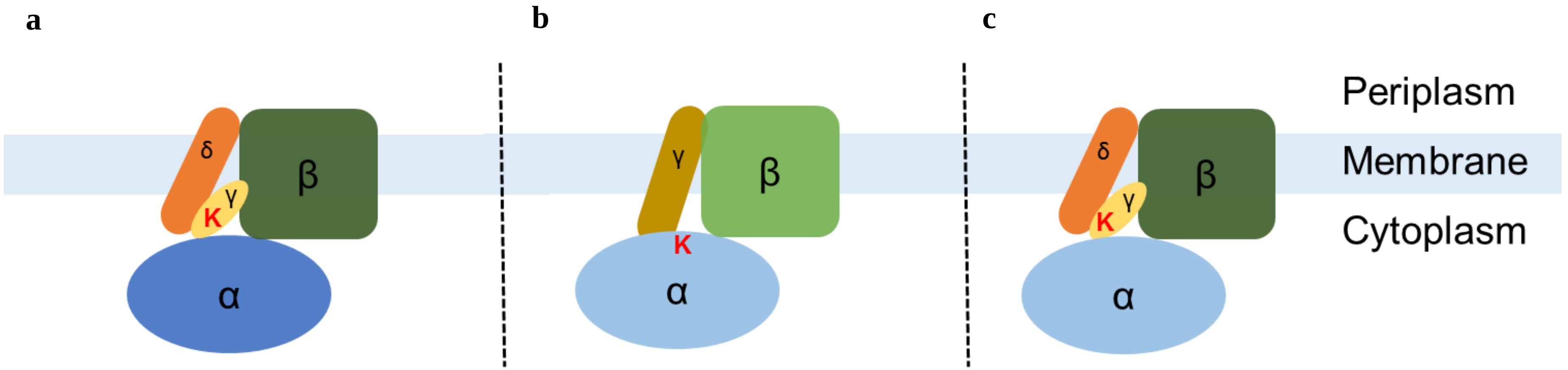

Supplementary Figure 25. MMDC and OADC structures. Diagrams are adapted from Klimchuk et al.[125]. (a) MMCD structure in *Quinella* (this study) and other bacteria[78]. (b) Normal OADC structure[78]. (c) Proposed functional OADC structure in *Quinella* (this study) using the α subunit of the OACD in a hybrid with the β, γ and δ subunits of MMDC. The K represents the putative biotin-binding lysine residue.

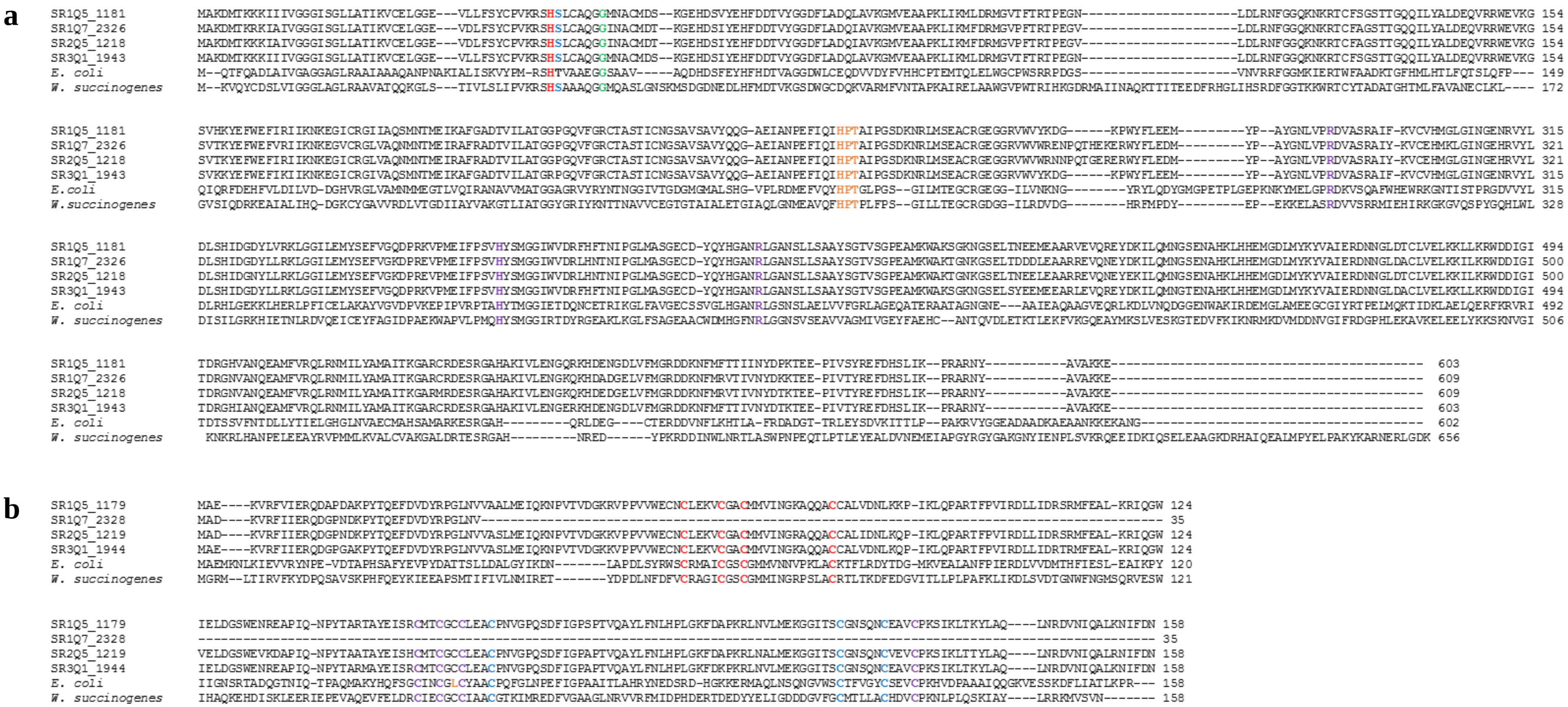

Supplementary Figure 26. Alignments of amino acid sequences of hydrophilic subunits of fumarate reductase from *Quinella* with those from *Escherichia coli* and *Wolinella succinogenes*. (a) Subunit A, showing conserved FAD-binding histidine residues (red), glycines that accept an H-bond from FAD (green), the 'HPT triad' (orange), the dicarboxylate-binding site (purple) and serines that are one of the 11 residue that bind to FAD (blue). (b) Subunit B, showing conserved cysteine residues that coordinate with 2Fe-2S clusters (red), with 3Fe-4S clusters (blue) or with 4Fe-4S clusters (purple). Like in *W. succinogenes*, and unlike in *E. coli*, cysteine (C154) is replaced by leucine.

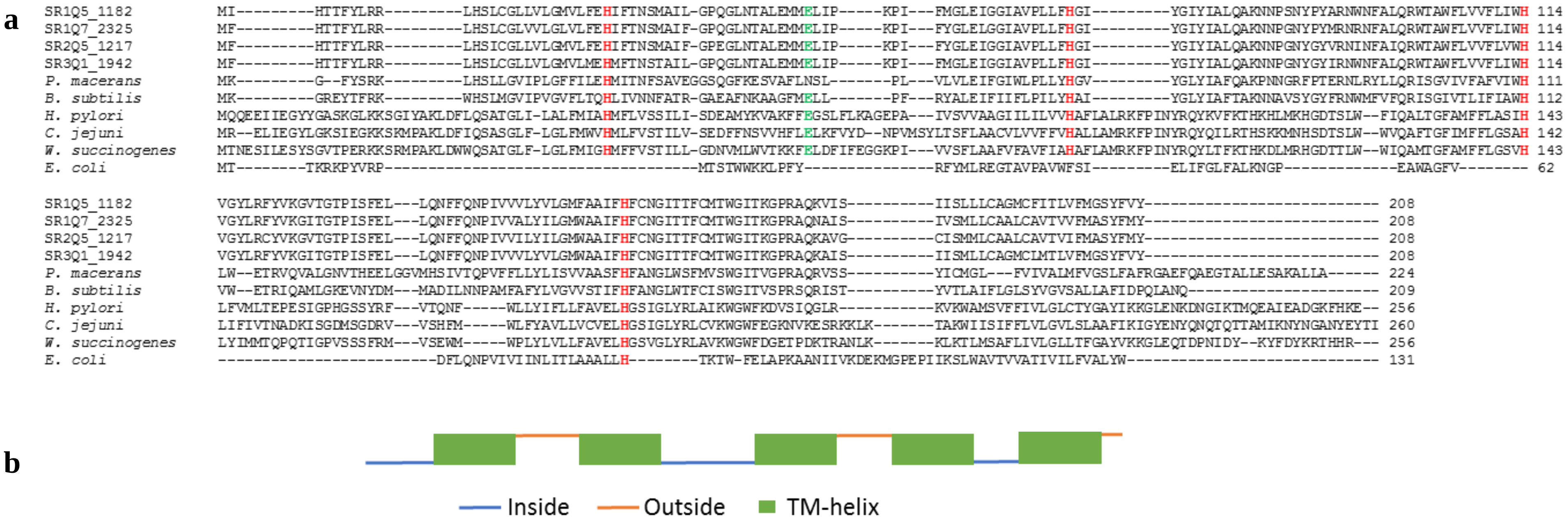

Supplementary Figure 27. Subunit C of fumarate reductase. (a) Alignment of amino acid sequences of *Quinella* subunit C with type B SQOR from *P. macerans*, *B. subtilis*, *H. pylori*, *C. jejuni*, *W. succinogenes*, and *E. coli*. Conserved histidine residues (red) provide axial ligands for proximal and distal heme groups. The conserved glutamate (green) is an essential residue, involved in menaquinol oxidation. (b) Schematic representation of the fumarate reductase predicted using SPOCTOPUS[18], suggesting that *Quinella* subunit C is composed of five membrane-spanning domains. TM, transmembrane.

```
SR1Q5_1786              1 ----MEHAIMVAAALLGAGITMGLAAIGAGVGDGLVTSKFIDGITRQPEAKNTLFTNTL
SR1Q7_892               1 ----MEHAIMVAAALLGAGITMGLAAIGAGVGDGLVTSKFIDGITRQPEAKNTLFTNTL
SR2Q5_1189              1 ----MEHAIMVAAALLGAGITMGLAAIGAGVGDGLVTSKFIDGITRQPEAKNTLFTNTL
SR3Q1_1280              1 ----MEHAIMVAAALLGAGITMGLAAIGAGVGDGLVTSKFIDGITRQPEAKNTLFTNTL
Bacillus subtilis       1 ------------MNLIAAAIAIGLGALGAGIGNGLIVSRTVEGIARQPEAGKELRTLMF
Escherichia coli        1 -----MENLNMDLLYMAAAVMMGLAAIGAAIGIGILGGKFLEGAARQPDLIPLLRTQFF
Propionigenium modestum 1 MDMVLAKTVVLAASAVGAGAAM-IAGIGPGVGQGYAAGKAVESVARQPEAKGDIISTMV
Ilyobacter tartaricus   1 MDMLFAKTVVLAASAVGAGTAM-IAGIGPGVGQGYAAGKAVESVARQPEAKGDIISTMV
Acetobacterium woodii   1 ---MEGLDFIKACSAIGAGIAM-IAGVGPGIGQGFAAGKGAEAVGRQPEAQSDIIRTML

SR1Q5_1786              56 ISVGLIEAMAIIATVVALIMLYANPLL---
SR1Q7_892               56 ISVGLIEAMAIIATVVALIMLYANPLL---
SR2Q5_1189              56 ISVGLIEAMAIIATVVALIMLYANPLL---
SR3Q1_1280              56 ISVGLIEAMAIIATVVALIMLYANPLLG--
Bacillus subtilis       48 MGIALVEALPIIAVVIAFLAFFG-------
Escherichia coli        55 IVMGLVDAIPMIAVGLGLYVMFAVA-----
Propionigenium modestum 59 LGQAIAESTGIYSLVIALILLYANPFVGLL
Ilyobacter tartaricus   59 LGQAVAESTGIYSLVIALILLYANPFVGLL
Acetobacterium woodii   56 LGAAVAETTGIYGLIVALILLFANPFF---
```

Supplementary Figure 28. Alignment of amino acid sequences from subunit c of ATP synthase. The diagnostic residue at position 32 of the *Quinella* sequences was not the **Q**/E expected for Na^+-specific ATP synthases[94]. The motif coloured red contains the conserved amino acid residue **E** at position 65 found in nearly all sequences except *E. coli*, and the residue at position 66 which is **S/T** in bacterial Na^+-specific ATP synthases and **A** in bacterial H^+-specific ATP synthases[94]. Sequence numbering is based on the *Ilyobacter tartaricus* protein.

```
A. aeolicus      --------------------MKRVVVDPVTRIEGHLRIEIMVDEETGQVKDALSAGTMWRGIELIVRNRDPRDVWAFTQRICGVCTSIHALASLRAVEDALEITIPKNANYIRNIMYGSLQVHDHVVHFYHLHALDWVSPVEALKADPVATAALANKILEKYGVLNEFM 149
EcHyd-1          ------MSTQYETQGYTINNAGRRLVVDPITRIEGHMRCEVNINDQN-VITNAVSCGTMFRGLEIILQGRDPRDAWAFVERICGVCTGVHALASVYAIEDAIGIKVPDNANIIRNIMLATLWCHDHLVHFYQLAGMDWIDVLDALKADPRKTSELAQSL----------- 152
R. eutropha      -------MSAYATQGFNLDDRGRRIVVDPVTRIEGHMRCEVNVD-ANNVIRNAVSTGTMWRGLEVILKGRDPRDAWAFVERICGVCTGCHALASVRAVENALDIRIPKNAHLIREIMAKTLQVHDHAVHFYHLHALDWVDVMSALKADPKRTSELQQLV----------- 138
SR1Q5_381        --------------------MQHVVVDPITRIEGHLRVEVTVDETKGTVTDAISSGTAWRGLELIMRDRDPRDAWAYIQRICGVCTTAHALASVRAVEDALGIGIPLNANYIRNIMAATLTVQDHLVHFYHLHALDWVSPVEALAADPAKTAELQVAVLNAYR-VDLKV 149
SR1Q7_532        --------------------MQHVVVDPITRIEGHLRVEVTVDETNGTVTDAISSGTAWRGLELILRDRDPRDAWAYIQRICGVCTTAHALASVRAVEDALGISIPNNANYIRNIMAATLTVQDHIIHFYHLHALDWVSPVEALAADPAKTAELQVAVLNAYR-LDLKV 149
SR2Q5_1553       --------------------MQHVVVDPITRIEGHLRVEITVDETTGTVTDALSSGTAWRGLELILRDRDPRDAWAYIQRICGVCTTAHALASVRAVEDALGISIPNNANYIRNIMAATLTVQDHLIHFYHLHALDWVSPVEALNADPAKTAELQTAVLNAYR-LDIKV 149
SR3Q1_2344       --------------------MQHVVVDPITRIEGHLRVEVTVDETNGTVTDAISSGTAWRGLELVLRDRDPRDAWAYIQRICGVCTTAHALASVRAVEDALGIAIPLNANYIRNIMAATLTTQDHLIHFYHLHALDWVSPVEALNADPAKTAELQTAVLNAYR-VDLKV 149
SR3Q1_2244       --------------------MQHVVVDPITRIEGHLRVEVTVDEANGTVTDAISSGTAWRGLELVMKGRDPRDAWAYIQRICGVCTTAHALASVRSVEDALGIAIPLNANYIRNIMAATLTVQDHLVHFYHLHALDWVSPVEALSADPAKTAELQTAVLNAYR-LDLKV 149
EcHyd-2          -------------------MSQRITIDPVTRIEGHLRIDCEIE--NGVVSKAWASGTMWRGMEEIVKNRDPRDAWMIVQRICGVCTTTHALSSVRAAESALNIDVPVNAQYIRNIILAAHTTHDHIVHFYQLSALDWVDITSALQADPTKASEMLKGV----------- 153
D. vulgaris      MSGCRAQNAPGGIPVTPKSSYSGPIVVDPVTRIEGHLRIEVEVE--NGKVKNAYSSSTLFRGLEIILKGRDPRDAQHFTQRTCGVCTYTHALASTRCVDNAVGVHIPKNATYIRNLVLGAQYLHDHIVHFYHLHALDFVDVTAALKADPAKAAKVASSI----------- 158
D. gigas         ---------------MSEMQGNKIVVDPITRIEGHLRIEVEVE--GGKIKNAWSMSTLFRGLEMILKGRDPRDAQHFTQRACGVCTYVHALASVRAVDNCVGVKIPENATLMRNLTMGAQYMHDHLVHFYHLHALDWVNVANALNADPAKAARLANDL----------- 142

A. aeolicus      PDFLGHRAYPKKFPKATPGYFREFQKKIKKLVESGQLGIFAAHWW--DHPDYQMLPPEVHLIGIAHYLNMLDVQRELFIPQVVFGGKNPHPHY-IVGGVNCSISMDD---MNAPVNAERLAVVEDAIYTQVESTDFFYIPDILAIADIYLNQHNWFY-GGGLSKKRVIGY 312
EcHyd-1          ----------SSWPKSSPGYFFDVQNRLKKFVEGGQLGIFRNGYW--GHPQYKL-PPEANLMGFAHYLEALDFQREIVKIHAVFGGKNPHPNW-IVGGMPCAINIDESG-AVGAVNMERLNLVQSIITRTADFINNVMIPDALAIGQFN---KPWSEIGTGLSDKCVLSY 306
R. eutropha      --------SPAH-PLSSAGYFRDIQNRLKRFVESGQLGPFMNGYW--GSKAYVL-PPEANLMAVTHYLEALDLQKEWVKIHTIFGGKNPHPNY-LVGGVPCAINLDGIGAASAPVNMERLSFVKARIDEIIEFNKNVYVPDVLAIGTLY-KQAGWLY-GGGLAATNVLDY 285
SR1Q5_381        PEEVNTEAYPHDFPAATPQYFAGIKSRVQAIVQSGQLGIFSAHWW--DHPDYKLLPPEVHLMAVAHYLEMLDKQREIITPHVVFGGKNPHPHY-VVGGMPCSISLTD---GNAPINTARLAIVDRAINMARDLVNNYYLPDLVAIGVIY-AKASRVD-GGGLARERVLAF 310
SR1Q7_532        PNEVATEAFPHDFPAATPQYFAGIKARVQAIVESGQLGIFAAQWW--DHPDYKLLPPEVHLMAVAHYLEMLDKQREIITPHVIFGGKNPHPHY-AVGGMPCSISMTD---GNAPINTARLAIVDRAINMARDLVNNYYLPDLVAIGVIY-AKAGRVD-GGGLSKTRVLGF 310
SR2Q5_1553       PEEVNTNAFPHDFPAATPQYFAGIKAKVQAIVASGQLGIFAAQWW--DHPDYKLLPPEVHLMAVAHYLEMLDKQREIITPHVIFGGKNPHPHY-AVGGMPCSISMTD---GNAPVNTARLAIVDRAINMARDLVNNYYLPDLVAIGVIY-AKAGRVD-GGGLSKTRVMAF 310
SR3Q1_2344       PNEVNTEAFPHDFPAATPQYFAGIKAKVQAIVQSGQLGIFSAHWW--DHPDYKLLPPEVHLMAVAHYLEMLDKQREIITPHVVFGGKNPHPHY-VVGGVPCSISLTD---GNAPINTARLAIVDRAINMARDLVNNYYLPDLVAIGAIY-AKAGRID-GGGLSKTRVLAF 310
SR3Q1_2244       PEEVMTEAYPHDFPAATPQYFASIKARVQAIVQSGQLGIFSAHWW--DHPDYKLLPPEVHLMAVAHYLEMLDKQREIITPHVVFGGKNPHPHY-VVGGVPCAISLTD---GNAPINTARLAIVDRAINMARDLVNNYYLPDLVAIGAIY-AKAGRID-GGGLARYRVLAF 310
EcHyd-2          ----------STWHLNSPEEFTKVQNKIKDLVASGQLGIFANGYW--GHPAMKL-PPEVNLIAVAHYLQALECQRDANRVVALLGGKTPHIQNLAVGGVANPINLD----GLGVLNLERLMYIKSFIDKLSDFVEQVYKVDTAVIAAFY---PEWLTRGKG--AVNYLSV 304
D. vulgaris      --------SPRKTTAAD---LKAVQDKLKTFVESGQLGPFTNAYFLGGHPAYYL-DPETNLIATAHYLEALRLQVKAARAMAVFGAKNPHTQFTVVGGVTCYDALTP----------QRIAEFEALWKETKAFVDEVYIPDLLVVAAAY---KDWTQYGG---TDNFITF 299
D. gigas         --------SPRKTTTES---LKAVQAKVKALVESGQLGIFTNAYFLGGHPAYVL-PAEVDLIATAHYLEALRVQVKAARAMAIFGAKNPHTQFTVVGGCTNYDSLRP----------ERIAEFRKLYKEVREFIEQVYITDLLAVAGFY---KNWA--GIGK-TSNFLTC 283

A. aeolicus      GDYPDEPYTGIKN-GDYHKKILWHSNGVVEDFYKGVEKAKFYNLEG-KDFTDPEQIQEFVTHSWYKYPDET-KGLHPWDGITEPNYT-GPKE-GTKTHWKYLDENGKYSWIKAPRWRGKACEVGPLARYIIVYTKVKQG--HI-KPTWVDELIVNQI-DTVSKILNLPPE 473
EcHyd-1          GAFPDIA-------NDFGEKSLLMPGGAVIN-------GDFNNVLP-VDLVDPQQVQEFVDHAWYRYPNDQ-VGRHPFDGITDPWYNPGDVK-GSDTNIQQLNEQERYSWIKAPRWRGNAMEVGPLARTLIAYHKGDAA--T-----------VESV-DRMMSALNLPLS 458
R. eutropha      GEYPN---------VAYNKSTDQLPGGAILN---GNWDEVF-PV----DPRDSQQVQEFVSHSWYKYADES-VGLHPWDGVTEPNYVLGANTKGTRTRIEQIDESAKYSWIKSPRWRGHAMEVGPLSRYILAYAHARSGNKYAERPKEQLEYSAQMINSAIPKALGLPET 414
SR1Q5_381        GDYPLTGYKGTST-GGYFENLLVRSNGVVENFGMGLDKVVFTPVTA-EDLKAPDIITEGVEHAWYEYPESAGKNLHPWKGVTKDKYT-GPKT-GTPTMWETLNEAGKYSWLKTPKWKGKLCEVGPLAHYIVIYTKAAKG--LLPEPTWAEQMMLKQI-EVVSKVLGVSAE 473
SR1Q7_532        GDYPLTGYKGTSNGGGYFDNLLVRSNGVVEDFGMGVDKARFTPVTA-DDLKAPDTFAEGVEHAWYEYPTGGAKDLHPWEGVTKDKYT-GPKT-GTPTMWETLNEAGKYSWLKTPKWKGKLCEVGPLAHYIIIYTKAAKG--LLPDPTWAEQMMLKQI-EAVSTVLGVSAE 474
SR2Q5_1553       GDYPLTGYKGTSNGGGYFDNLLVRSNGVVENFGQGVAKAVFHELKA-EDLKAPETVTEGVEHAWYEYPTNGPKDLHPWQGVTKDKYT-GPKT-GTPTMWETLNEAGKYSWLKTPKWKGKLCEVGPLAHYIIIYTKAAKG--LLPDPTWAEQLMLKQI-EAVSKVLGVSAE 474
SR3Q1_2344       GDYPLTGYKGTSS-GGYFENLLVRSNGVVENFGMGVEKAVFTPITA-EDLKAPDVISEAVEHAWYEYPENAGKNLHPWKGVTKDKYT-GPKT-GTPTMWETLNEAGKYSWLKTPKWKGKLCEVGPLAHYIIIYTKAQKG--QLPEPTWAEQMMLKQI-EVVSTVVGVPPH 473
SR3Q1_2244       GDYPLAGYKGTST-GGYFENLLVRSNGVVENFADGVDKAFFSPITA-EDLKAPDVISEAVEHAWYEYPENAGKNLHPWMGVTKDKYT-GPKT-GTPTMWETLNEAGKYSWLKTPKWKGRLCEVGPLAHYIIIYTKAQKG--LLPDPTWAEQMMLKQI-EVVSTVLGVPPH 473
EcHyd-2          PEFP----------TDSKNGSFLFPGGYIEN----ADLSSYRPITSHSDEYLIKGIQESAKHSWYKDEAPQ----APWEGTTIPAYD-G---------W---SDDGKYSWVKSPTFYGKTVEVGPLANMLVKLAAGRES----------TQNKLNEIVAIYQKLTGNTLE 443
D. vulgaris      GEFPK---------DEYDLNSRFFKPGVV-------FKRDFKNI----KPFDKMQIEEHVRHSWYEGAEAR----HPWKGQTQPKYT-------------DLHGDDRYSWMKAPRYMGEPMETGPLAQVLIAYSQGHPK-------------VKAVTDAVLAKLGVGPE 418
D. gigas         GEFPT---------DEYDLNSRYTPQGVI-------WGNDLSKV----DDFNPDLIEEHVKYSWYEGADAH----HPYKGVTKPK-------------WTEFHGEDRYSWMKAPRYKGEAFEVGPLASVLVAYAKKHEP------------TVKAV-DLVLKTLGVGPE 402

A. aeolicus      KW-----LPTTVGRTIARALEAQMSAHTNLYWMKKLYDNIKAGDTSVANMEKWDPSTWPKEAKGVGLTEAPRGALGHWVIIKDGKVANYQCVVPTTWNGSPKDPKGQHGAFEESMIDTKVKVPEKPLEVLRGIHSFDPCLACSTHLYNEKGEEIASVRVQGVVHV 633
EcHyd-1          G------IQSTLGRILCRAHEAQWAAGKLQYFFDKLMTNLKNGNLATASTEKWEPATWPTECRGVGFTEAPRGALGHWAAIRDGKIDLYQCVVPTTWNASPRDPKGQIGAYEAALMNTKMAIPEQPLEILRTLHSFDPCLACSTHVLGDDGSELISVQVR----- 618
R. eutropha      QYTLKQLLPSTIGRTLARALESQYCGEMMHSDWHDLVANIRAGDTATANVDKWDPATWPLQAKGVGTVAAPRGALGHWIRIKDGRIENYQCVVPTTWNGSPRDYKGQIGAFEASLMNTPMVNPEQPVEILRTLHSFDPCLACSTHVMSAEGQELTTVKVR----- 567
SR1Q5_381        VW-----MPTMLGRTACRCLDAQLAAEINKFFFDKLIANIKMGDTAVMNNEKWTPDTWATDCMGVGLYEAPRGGLSHWVCIKNGKISNYQCIVPTTWNACPRDDQAGHGAYELAMMTTHVAVPDKPLEIAKVIRSFDPCMACATHMFNAKGEEINIISTDPYGR- 632
SR1Q7_532        IW-----MPTMLGRTACRCLDAQLAAEINKFFFDKLIANIKMGDTATMNNEKWFPETWARECHGVGLYEAPRGALSHWVCIKDGKVDNYQCIVPTTWNACPRDDQAGHGAYELAMMTTHVAVPDKPLEIAKVIRSFDPCMACATHMFNANGEEINIISTDPYGR- 633
SR2Q5_1553       VW-----MPTMLGRTACRCLDAQLAAEINKFFFDKLIANIKMGDTATMNNEKWFPETWAPESMGVGLYEAPRGALSHWVCIKDGKVDNYQCLVPTTWNACPRDDSAGHGAYEQAMMTTHVAVPDKPLEIAKVIRSFDPCMACATHMFNAKGEELGIISTDPYGR- 633
SR3Q1_2344       VW-----MPTMLGRTACRCLDAQLAAEVNKFFFDKLIANLKTGDTSVMNNEKWTPDTWATECMGVGLYEAPRGGLSHWVCIKNGKIDNYQCIVPTTWNACPRDDQAGHGAYELAMMTTHVAIPDKPLEIGKVVRSFDPCMACATHMYNAKGEEINIISTDPYGR- 632
SR3Q1_2244       VW-----MPTMLGRTACRCLDAQLAAEVNKFFFDKLIANLKTGDTTTMNNEKWTPDTWATECMGVGLYEAPRGGLSHWVKIKDGKIDNYQCIVPTTWNACPRDDIAGHGAYELAMMTTHVAIPNKPLEIGKVIRSFDPCMACATHMYNAKGEEINIISTDPYGR- 632
EcHyd-2          VAQ----LHSTLGRIIGRTVHCCELQDILQNQYSALITNIGKGDHTTFVKPNI-PAT--GEFKGVGFLEAPRGMLSHWMVIKDGIISNYQAVVPSTWNSGPRNFNDDVGPYEQSLVGTPVADPNKPLEVVRTIHSFDPCMACAVHVVDADGNEVVSVKVL----- 597
D. vulgaris      A------LFSTLGRTAARGIETAVIAEYVGVMLQEYKDNIAKGDNVICA--PW---EMPKQAEGVGFVNAPRGGLSHWIRIEDGKIGNFQLVVPSTWTLGPRCDKNKLSPVEASLIGTPVADAKRPVEILRTVHSFDPCIACGVHVIDGHTNEVHKFRIL----- 567
D. gigas         A------LFSTLGRTAARGIQCLTAAQEVEVWLDKLEANVKAGKDDLYT--DW---QYPTESQGVGFVNAPRGMLSHWIVQRGGKIENFQHVVPSTWNLGPRCAERKLSAVEQALIGTPIADPKRPVEILRTVHSYDPCIACGVHVIDPESNQVHKFRIL----- 551
```

Supplementary Figure 29. Large subunit of the hydrogenase of *Quinella*. Amino acid sequence alignment of the large subunit of the hydrogenase of *Quinella*. shown together with O_2-tolerant (*Escherichia coli* Hyd-1, *Ralstonia eutropha* and *Aquifex aeolicus*) and O_2-sensitive (*E. coli* Hyd-2, *Desulfovibrio vulgaris* and *D. gigas*) large subunit sequences. Motif 1 (light blue with conserved cysteines in red) matches with 1d type hydrogenases while motif 2 (purple with conserve cysteines in red) matches with 1e type hydrogenases as defined by Greening et al.[97]. Conserved metal-binding residue His236 (green) in *Quinella* that coordinates with the proximal 4Fe4S or 4Fe3S cluster of the small subunit[101].

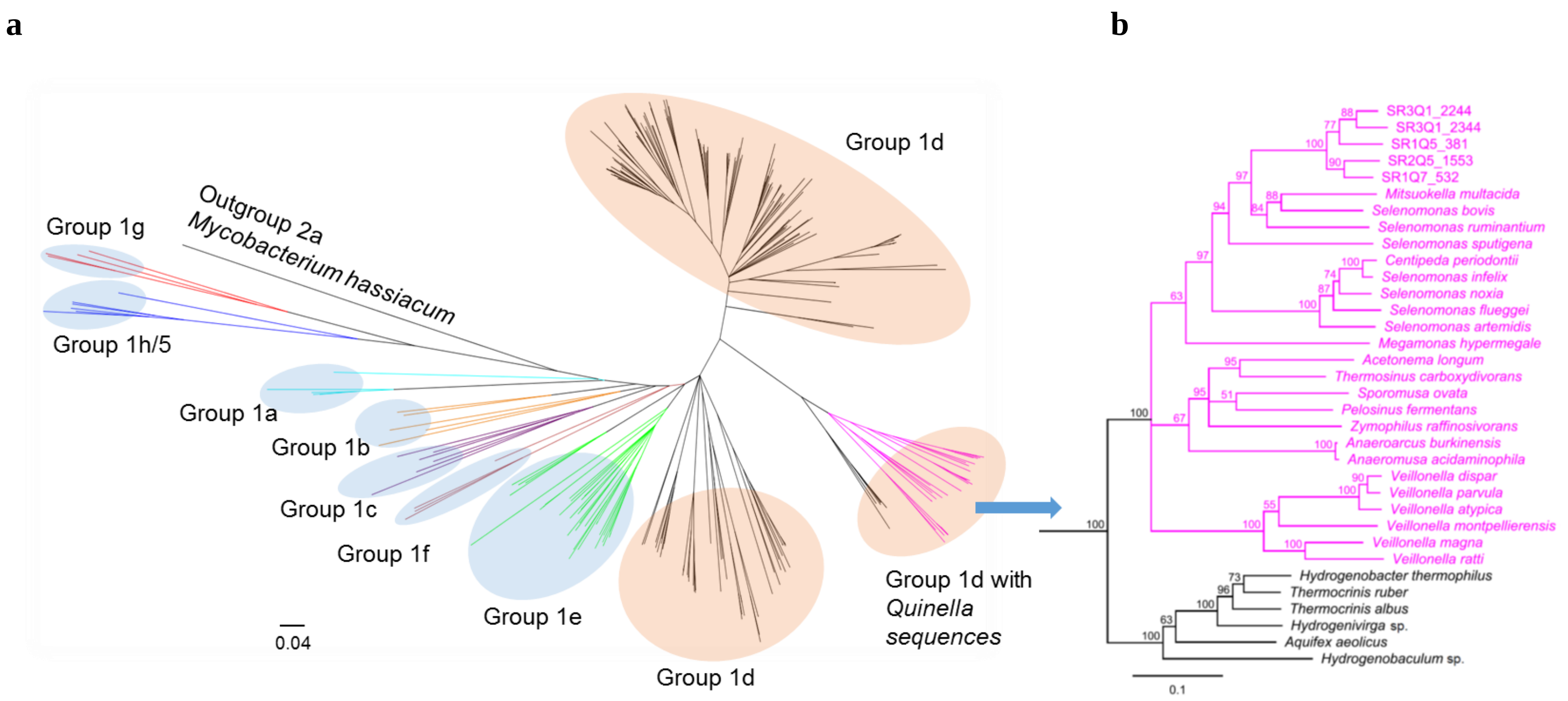

Supplementary Figure 30. Phylogenetic classification of *Quinella* hydrogenases using the amino acid sequences of the large (catalytic) subunit. (a) Phylogenetic tree built using all sequences from 1d and 1e type, and five sequences each from 1a, 1b, 1c, 1f and 1h/5 type hydrogenases from the Greening et al.[97] database and 5 sequences from the *Quinella* genome bins. Sequences were aligned using MUSCLE[126] and Clustal W[127], and then a phylogenetic tree was constructed using the Jukes-Cantor genetic distance model[123] with the Neighbor-Joining method[124] and 500 bootstrap resamplings. A 2a type hydrogenase sequence from *Mycobacterium hassiacum* was used as an outgroup sequence to root the tree. (b) Detailed view of the cluster containing the *Quinella* sequences (pink) and adjacent 1d sequences (black). The scale bar represents 0.04 and 0.1 changes per amino acid position in panels (a) and (b) respectively. The numbers at the nodes are the percentage of trees that conserved that node in 500 bootstrap resamplings.

a

```
A. aeolicus     ----------------------------------------------------------------------------------------------METFWEVFKRHGVSRRDFLKFATTITGLMGLAPSMVPEVVRAMETKPRVPVLWIHGLECTCCSESFIRSATPLASD 76
EcHyd-1         -------------------------------------------------------------------------------------------MNNEETFYQAMRRQGVTRRSFLKYCSLAATSLGLGAGMAPKIAWALENKPRIPVVWIHGLECTCCTESFIRSAHPLAKD 79
R. eutropha     ---------------------------------------------------------------------------------------------MVETFYEVMRRQGISRRSFLKYCSLTATSLGLGPSFLPQIAHAMETKPRTPVLWLHGLECTCCSESFIRSAHPLAKD 77
SR1Q5_380       -------------------------------------------------------------------------------------------MEKRESLWEAYLRRGFSRRSFLKGCVALTSLMGFSTDMFSKVVEAAESKPLPVVIWIHGHECTGCDESFIRSGAPLASD 79
SR1Q7_531       -------------------------------------------------------------------------------------------METRESLWEAYLKRGFSRRSFLKGCVALTSLMGLSTDMFSKVVEAAETKPLPVVIWIHGHECTGCDESFIRSGAPLASD 79
SR2Q5_1552      -------------------------------------------------------------------------------------------METRESLWEAYLRRGLSRRSFLKGCVALTSLMGLSADMFSKVVEAAENKPLPVVIWIHGHECTGCDESFIRSGAPLASD 79
SR3Q1_2245      -------------------------------------------------------------------------------------------MEKRESLWEAYLRRGFSRRSFLKGCVALTSLMGLSTDMFSKVVEAAESKPLPVVIWIHGHECTGCDESFIRSGAPLASD 79
SR3Q1_2502      -------------------------------------------------------------------------------------------MEKRESLWEAYLKRGFSRRSFLKGCVALTSLMGFGTDMFSKVVEAAETKPLPVVIWIHGHECTGCDESFIRSGAPLASD 79
SR3Q1_2343      MAISFRRFGNILLLKIFIVKLRYPLTSEKYRRRVDNRRAFFYKWRSTSGRIYKNFGESCCQYRIDIAQRMLKFFGTYYAYHLPIIFEEGRTLEKRESLWEAYLRRGFSRRSFLKGCVALTSLMGLSTDMFSKVVEAAEMKPLPVVIWIHGHECTGCDESFIRSGAPLASD 170
EcHyd-2         -------------------------------------------------------------------------------------------MTGDNTLIHS---HGINRRDFMKLCAALAATMGLSSKAAAEMAESVTNPQRPPVIWIGAQECTGCTESLLRATHPTVEN 76
D. vulgaris     --------------------------------------------------------------------------------------MKISIGLGKEGVEERLAERGVSRRDFLKFCTAIAVTMGMGPAFAPEVARALMGPRRPSVVYLHNAECTGCSESVLRAFEPYIDT 84
D. gigas        ----------------------------------------------------------------------------------------------------------------MKFCTAVAVAMGMGPAFAPKVAEALTAKKRPSVVYLHNAECTGCSESLLRTVDPYVDE 58

A. aeolicus     VVLSMISLEYDDTLSAAAGEAVEKHRERIIKEYWGNYILAVEGNPPLGEDGMYCIIGGRPFVEILKESAEGAKAVIAWGSCASWGCVQAAKPNPTTAVPIDKVIK--DKPIIKVPGCPPIAEVMTGVIMYMVLFDRIPPLDSQGRPKMFYGNRIHDTCYRRSFFNAGQFV 244
EcHyd-1         VILSLISLDYDDTLMAAAGTQAEEVFEDIITQYNGKYILAVEGNPPLGEQGMFCISSGRPFIEKLKRAAAGASAIIAWGTCASWGCVQAARPNPTQATPIDKVIT--DKPIIKVPGCPPIPDVMSAIITYMVTFDRLPDVDRMGRPLMFYGQRIHDKCYRRAHFDAGEFV 247
R. eutropha     VVLSMISLDYDDTLMAAAGHQAEAILEEIMTKYKGNYILAVEGNPPLNQDGMSCIIGGRPFIEQLKYVAKDAKAIISWGSCASWGCVQAAKPNPTQATPVHKVIT--DKPIIKVPGCPPIAEVMTGVITYMLTFDRIPELDRQGRPKMFYSQRIHDKCYRRPHFDAGQFV 245
SR1Q5_380       VLLSSIALEYDHLLSAACGAPFEAHLDETITKYNGQYILAVEGAIATKDSGVYCMSGGHTFTHLLEKAAKGAAAIIAYGSCSAYGGIQAAKPNPTGSAGIERFVG--GKPVVKVPGCPPIPEVMTGVVMHVALFGTLPPVDADGRPKQFYGNRIHDTCYRRPFFDAGMFA 247
SR1Q7_531       VVLSSIALEYDHLLSAACGAPFEAHLDETIAKYNGQYILAVEGAIATKDSGVYCMSGGHTFTHLLEKAAKGAAAIIAYGSCSAYGGIQAAKPNPTGSAGIERFVG--GKPVVKVPGCPPIPEVMTGVVMHVALFGTIPPVDGDGRPKQFYGNRIHDTCYRRPFFDAGMFA 247
SR2Q5_1552      VVLSSIALEYDHLLSAACGAPFEAHLDETIAKYNGQYILCVEGAIATKDSGVYCMSGGHTFTHLLEKAAKGAAAIIAYGSCSAYGGIQAAKPNPTGSAGIERFVG--GKPVVKVPGCPPIPEVMTGVVMHVALFGTIPPVDGDGRPKQFYGNRIHDTCYRRPFFDAGMFA 247
SR3Q1_2245      VVLSSIALEYDHLLSAACGAPFEAHLDETIAKYKGQYILAVEGAVATRDSGVYCMSGGHTFTHLLEKAAAGAAAIIAYGSCAAYGGIQAAKPNPTGSAGIEHFVG--GKPVVKVPGCPPIPEVMTGVVMHVALFGSLPPCDADGRPKQFYGNRIHDTCYRRPFFDAGMFA 247
SR3Q1_2502      VVLSSIALEYDHLLSAACGAPFEAHLDETIAKYNGQYILAVEGAVATKDSGVYCMSGGHTXXXX-------------------------------------------------------------------------------------------------------- 144
SR3Q1_2343      VVLSSIALEYDHLLSAACGAPFEAHLDETIAKYNGQYILAVEGAVATRDSGVYCMSGGHTFTHLLEKAAKGAAAIIAYGSCSAYGGIQAAKPNPTGSAGIERFVG--GKPVVKVPGCPPIPEVMTGVVMHVALFGSLPPCDADGRPKPFYGNRIHDTCYRRPFFDAGMFA 338
EcHyd-2         LVLETISLEYHEVLSAAFGHQVEENKHNALEKYKGQYVLVVDGSIPLKDNGIYCMVAGEPIVDHIRKAAEGAAAIIAIGSCSAWGGVAAAGVNPTGAVSLQEVLP--GKTVINIPGCPPNPHNFLATVAHIITYGKPPKLDDKNRPTFAYGRLIHEHCERRPHFDAGRFA 244
D. Vulgaris     LILDTLSLDYHETIMAAAGDAAEAALEQAVNSPHG-FIAVVEGGIPTAANGIYGKVANHTMLDICSRILPKAQAVIAYGTCATFGGVQAAKPNPTGAKGVNDALKHLGVKAINIAGCPPNPYNLVGTIVYYLKNKAAPELDSLNRPTMFFGQTVHEQCPRLPHFDAGEFA 253
D. gigas        LILDVISMDYHETLMAGAGHAVEEALHEAI---KGDFVCVIEGGIPMGDGGYWGKVGRRNMYDICAEVAPKAKAVIAIGTCATYGGVQAAKPNPTGTVGVNEALGKLGVKAINIAGCPPNPMNFVGTVVHLLTKG-MPELDKQGRPVMFFGETVHDNCPRLKHFEAGEFA 244

A. aeolicus     EQFDDEGAKKGWCLYKVGCRGPTTYNSCGNMRWYN-GLSYPIQSGHGCIGCAENNF-----------WDN-GPFYERIGGIPVPGIESKADKVGAIAAAAAAGGAIIHGIASKIRKSGEKEE---------------- 353
EcHyd-1         QSWDDDAARKGYCLYKMGCKGPTTYNACSSTRWND-GVSFPIQSGHGCLGCAENGF-----------WDR-GSFYSRVVDIPQMGTHSTADTVGLTALGVVAAAVGVHAVASAVDQRRRHNQQPTETEHQPGNEDKQA 372
R. eutropha     EEWDDESARKGFCLYKMGCKGPTTYNACSTTRWNE-GTSFPIQSGHGCIGCSEDGF-----------WDK-GSFYDRLTGISQFGVEANADKIGGTASVVVGAAVTAHAAASAIKRASKKNETSGSEH---------- 361
SR1Q5_380       ENFDDAGAKAGWCLYKLGCRGPETYNSCGNLRWWQ-GMSYPIQSGAPCIGCSNSNF-----------WDE-DPFTDRL---PKYGKLGDVDKIGVGLGVLTAAGVGAHAVASVIQRNQRENLLEEDHHE--------- 361
SR1Q7_531       ETFDDAGAKAGWCLYKLGCRGPETYNSCGNLRWWQ-GMSYPIQSGAPCIGCSNSNF-----------WDE-DPFTARL---PQYGKLGDADKIGVGLGVLTAAGVVGHAVASIVQRNQRDNLID-EHHE--------- 360
SR2Q5_1552      ETFDDAGAKAGWCLYKLGCRGPETYNSCGNLRWWQ-GMSYPIQSGAPCIGCSNSNF-----------WDE-DPFTARL---PQYGKIGDADKIGTALGVLTAAGVVGHAVASVIQRNQRDNLIDEEHHE--------- 361
SR3Q1_2245      ETFDDAGAKAGWCLYKLGCRGPETYNSCGNLRWWQ-GMSYPIQSGAPCIGCSNSNF-----------WDE-DPFTDRL---PKYGKFGDVDKIGAALGVLTAAGVAGHAVASIVQRNTRENLIDEEHHE--------- 361
SR3Q1_2502      ------------------------------------------------------------------------------------------------------------------------------------------ 144
SR3Q1_2343      ETFDDAGAKAGWCLYKLGCRGPETYNSCGNLRWWQ-GMSYPIQSGAPCIGCSNANF-----------WDE-DPFTNRL---PQYGKFGDVDKIGAALGVLTAAGVAGHAVASIVQRNQRENLIDEEHH---------- 451
EcHyd-2         KEFGDEGHREGWCLYHLGCKGPETYGNCSTLQFCDVGGVWPVAIGHPCYGCNEEGIGFHKGIHQLANVENQTPRSQKPDVNAKEGGNVSAGAIGLLGGVVGLVAGVSVMAVRELGRQQKKDNADSRGE---------- 373
D. vulgaris     PSFESEEARKGWCLYELGCKGPVTMNNCPKIKFNQ--TNWPVDAGHPCIGCSEPDF-----------WDAMTPFYQN------------------------------------------------------------- 318
D. gigas        TSFGSPEAKKGYCLYELGCKGPDTYNNCPKQLFNQ--VNWPVQAGHPCIACSEPNF-----------WDLYSPFYSA------------------------------------------------------------- 289
```

b

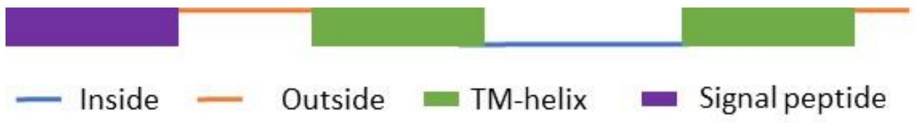

Supplementary Figure 31. Small subunit of the hydrogenase of *Quinella*. (a) Amino acid sequence alignment of the small subunit of the hydrogenase of *Quinella* together with O_2-tolerant (*Escherichia coli* Hyd-1, *Ralstonia eutropha* and *Aquifex aeolicus*) and O_2-sensitive (*E. coli* Hyd-2, *Desulfovibrio vulgaris* and *D. gigas*) small subunit sequences. Conserved cysteine (red) and glycine (light blue) residues proximal to the 4Fe4S cluster are indicators of O_2-sensitive type hydrogenases[105]. Conserved arginine and lysine residues (orange) are proposed to be exposed towards the carboxylate group of the heme of the *b*-type cytochrome associated with NiFe type membrane-bound hydrogenases[107]. (b) Schematic representation of the transmembrane helices and signal peptide in the small subunit of the *Quinella* hydrogenase, based on SPOCTOPUS predictions[18]. TM, transmembrane.

a

```
SR1Q5_382          ------------------MAIKSVKDVKRKEYYIFSPFLRFFHWIMASQIVVLFATGLLITKP--MNVGAVEPVFT---NTSMDLVRDIHFTAAFILCAALILRVY-GFIVNKGDR-LF-PRVWEGHFYEETVDVALHYM 114
SR1Q7_533          ------------------MASKSVKDVQRKEYYIFSPFLRIFHWIMASQIVILFATGLLITKP--MNVAITEPVFS---ITSMDLVRDIHFTSAFILCASLILRMY-GFIINKGDR-LF-PRVWEGHFYEETVDVALHYM 114
SR2Q5_1554         ------------------MAIKSVKDVHRKEYYIFSPFLRIFHWIMASQIVILFATGLLITKP--MNVAITEPVFS---ITSMDLVRDIHFTSAFILCASLILRIY-GFIINKGDR-LF-PRVWEGHFYEETVDVALHYM 114
SR3Q1_2243         ------------------MAIKSVKNVKRKEYYLFSPFLRIFHWIMVVMILVLFGTGLLITKP--MNVGGTEPVFA---GMSVDIIRDVHFLAAFILCAALIMRVY-GFIINKGDR-LF-PRVWEGHFYSETVEVALHYM 114
SR3Q1_2345         ------------------MAIKSVKDVKRKEYYLFSPFLRIFHWIMVVMILVLFGTGLLITKP--MNVGGTEPVFA---GMSVDIIRDIHFLAAFILCAGLIMRVY-GFIINKGDR-LF-PRVWEGHFYSETVEVALHYM 114
EcHyd-1            ------------------MQQKSDNVV---SHYVFEAPVRIWHWLTVLCMAVLMVTGYFIGKP--LPSVSGEATY----LFYMGYIRLIHFSAGMVFTVVLLMRIYWAFVGNRYSRELFIVPVWRKSWWQGVWYEIRWYL 113
A. aeolicus        ---------------------------MFKRIFIFSASLRIWHWINFLTIMVLFFTGLYIGTPYFTTSSGYEATYAYDKNITMGFIRFLHFSAGYVLLFSFLFRIFIAFF-RKGDR-LFIPKFWTIEYWEGIKETLKEYL 111
R. eutropha        MSTKMQADRIADATGTDEGAVASGKSI--KATYVYEAPVRLWHWVNALAIVVLAVTGFFIGSP--PATRPGEASA----NFLMGYIRFAHFVAAYIFAIGMLGRIYWATAGNHHSRELFSVPVFTRAYWQEVISMLRWYA 132
S. ruminantium     -----------------------MKQVVRREYYLFSPWLRIFHWIMVVSICVLFATGLMITSPWI--IYSAEPTFS---GMTVDNIRNIHFLAAYLFSAAFILKIY-GFIINPGDR-LF-PHVWKGYFYSNTVDVALHYM 109

SR1Q5_382          LLRGHHASFL-RNPLARGSYAVLYVL-LAIEIFTGFAMYCAANPTGTGY------LLFGWVNTLVGSEMMSHYIHHYVAWFIILFAIGHLYMVIRAEFMEGESEISSMFSGKKILAHTPKDANELD---- 232
SR1Q7_533          LIRPHHASFL-RNPLARGSYAALYLL-LAIEILTGFAMYFMTNPSSTGG------MLFGWVNVLVGSEMMAHYIHHYVAWFIILFAIGHLYMVIRAEFMEGESEISSMFSGKKILAHTPKDANELD---- 232
SR2Q5_1554         LIRPHHASFL-RNPLARGSYAALYLL-LLIEILTGFAMYFMTNPSSTGG------MLFGWVNVLVGSEMMAHYIHHYVAWFIILFAIGHLYMVIRAEFMEGESEISSMFSGKKILEHTPKDANELD---- 232
SR3Q1_2243         LLKSHHAPFL-RNPLARGSYAGLYVL-LAIEILTGFAMYLMANPFSTGG------MLFGWVNTLVGSEMMSHYIHHYVAWIIILFAIGHFYMVVRAEFMEGESEVSSMFSGKKVLPHTPKDANELD---- 232
SR3Q1_2345         LLKSHHSPFL-RNPLARGSYAGLYVL-LAIEILTGFAMYLMANPSSVFG------SLFNWVNVLVGSEMMSHYIHHYVAWIIILFAIGHFYMVVRAEFMEGESEVSSMFSGKKVLPHTPKDANELD---- 232
EcHyd-1            FLAKRPSADIGHNPIAQAAMFGYFLM-SVFMIITGFALYSEHSQYAIFAPFRYVVEFFYWTG---GNSMDIHSWHRLGMWLIGAFVIGHVYMALREDIMSDDTVISTMVNGYRSHKFGKISNKERS---- 235
A. aeolicus        LIGSRK-PMI-RNPLARTAYFFVYLM-IFFMIATGFGMYGLSDPDGFWA------SVFGWVVWSLGGEFEAHYWHHIVAWLIILFFIVHVYFVSREDFVHKNGEVSSMFNGSKFFKKDPVDLKDIK---- 228
R. euturopha       FLSARPSRYVGHNPLARFAMFFIFFLSSVFMILTGFAMYGEGAQMGSWQ-----ERMFGWVIPLLGQSQDVHTWHHLGMWFIVVFVIVHVYAAIREDIMGRQSVVSTMVSGYRTFKD------------ 244
S. ruminantium     FLRSEHASYL-RNPLARCSYAFLYVL-VFIEILTGFGIYYMTDPFGIGG------TLFGWVITVLGGEMMAHLVHHYVAWAIILFAIGHLYMVIRAEFMEGESEVSSMFSGSKILAHEPRDAGHLDGTLK 231
```

b

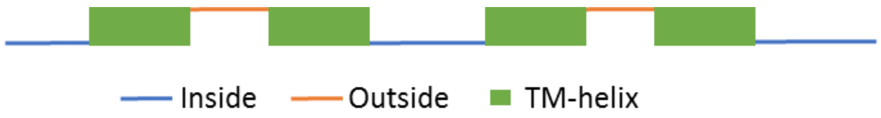

Supplementary Figure 32. Cytochrome-b subunit of the *Quinella* hydrogenase. (a) Amino acid sequences alignment of the cytochrome-*b* subunit of the *Quinella* hydrogenase, together with reference cyt*b* from other hydrogenases from *Escherichia coli*, (EcHyd-1), *Ralstonia eutropha*, *Aquifex aeolicus* and *Selenomonas ruminantium*. At least three (His43, His90, and His215) of the four conserved histidine residues (red) serve as ligands for two heme groups[109]. (b) Schematic representation showing the four transmembrane helices based on SPOCTOPUS predictions[18]. TM, transmembrane.

Supplementary Table 1. Relative abundance of *Quinella* spp. in rumen and forestomach samples from the Global Rumen Census. Relative abundances were calculated from counts of partial 16S rRNA genes amplified from 682 rumen samples[128] that were classified using QIIME[5] and the SILVA 123 16S rRNA gene database with refined rumen bacterial taxonomy[6]. Only the data for *Quinella* spp. are shown.

Source of samples	Mean relative abundance (%) of *Quinella* spp.[a]	Standard deviation	Maximum relative abundance (%)	Minimum relative abundance (%)	Number of samples
Bos indicus cattle	0.869	2.851	16.137	ND[a]	33
Bos taurus cattle	0.016	0.036	0.308	ND	320
Bos taurus × *indicus* hybrid cattle	0.077	0.282	1.760	ND	43
American bison (*Bison bison*)	0.323	0.640	1.871	0.008	14
Water buffalo (*Bubalus bubalis*)	0.028	0.065	0.301	ND	24
Sheep (*Ovis aries*)	2.367	6.369	48.566	ND	100
Goats (3 species)	2.859	4.194	18.967	ND	43
Deer (7 species)	4.283	6.048	24.200	ND	60
Antelopes (5 species)	6.459	5.746	16.316	0.025	13
South American camelids (3 species)	5.579	4.336	15.247	0.031	20
Giraffes (*Giraffa camelopardalis*)	2.342	5.526	19.700	ND	12
All samples	1.294	3.908	48.566	ND	682

[a]ND, not detected.

Supplementary Table 2. Long-length 16S rRNA gene sequences of *Quinella* spp. obtained from rumen samples. The rumen samples were from the study of Kittelmann et al.[4]. Samples from different cohorts of sheep within that study were selected to increase the likelihood of capturing a wider diversity of *Quinella* spp. Bacterial 16S rRNA genes were amplified, cloned, and partly sequenced to identify those actually from *Quinella* spp. These were then further sequenced to obtain 16S rRNA gene sequences of at least 1443 bp. The GenBank accessions are given in Supplementary Figure 1 and Supplementary Table 3.

Rumen sample[a]	Relative abundance (%) of *Quinella* spp.[a]	Total number of clones sequenced	Number of clones identified as related to *Quinella* spp. and sequenced to ≥ 1443 bp
S9643.C2b	74.4	10	3
S9110.C2b	68.8	10	4
S964.C3b	59.4	18	6
S154.C3b	51.7	15	5
S386.C4b	27.7	16	1[b]
S1120.C1b	28.4	33	6
S350.C4b	4.4	22	1

[a]See Kittelmann et al.[4].

[b]Close relative of *Quinella*, assigned to *Selenomonadaceae* candidate genus 1 candidate species 2 (Figure 1).

Supplementary Table 3. Alignment of probe Quin1231 (*Escherichia coli* positions 1231-1248) to matching region of *Quinella* 16S rRNA gene sequences. The 26 new sequences generated in this study are marked with an asterisk. Mismatches to the probe region are highlighted in grey.

Sequence (NCBI accession no.)	Sequence clusters[a]	Probe target region
MF184892*	*Quinella* candidate species 4	CUACACAC**GUACUACAAUGGG**-**CUGAA**CAGAAGG
MF184893*		CUACACAC**GUACUACAAUGGG**-**CUGAA**CAGAAGG
MF184888*		CUACACAC**GUACUACAAUGGG**-**CUGAA**CAGAAGG
MF184887*		CUACACAC**GUACUACAAUGGG**-**CUGAA**CAGAAGG
AB494921		CUACACAC**GUACUACAAUGGG-CUGAA**CAGAAGG
EF436437	*Quinella* candidate species 3	CUACACAC**GUACUACAAUGGG**-**CUGAA**CAGAAGG
EF436435		CUACACAC**GUACUACAAUGGG**-**CUGAA**CAGAAGG
EF436434		CUACACAC**GUACUACAAUGGG-CUGAA**CAGAAGG
EF436436		CUACACAC**GUACUACAAUGGG**-**CUGAA**CAGAAGG
EF436332		CUACACAC**GUACUACAAUGGG**-**CUGAA**CAGAAGG
MF184915*	*Quinella* candidate species 6	CUACACAC**GUACUACAAUGGG**-**CUGAA**CAGAAGG
MF184920*		CUACACAC**GUACUACAAUGGG**-**CUGAA**CAGAAGG
MF184919*		CUACACAC**GUACUACAAUGGG**-**CUGAA**CAGAAGG
MF184917*		CUACACAC**GUACUACAAUGGG**-**CUGAA**CAGAAGG
DQ673489		CUACACAC**GUACUACAAUGGG**-**CUGAA**CAGAAGG
EF436383		CUACACAC**GUACUACAAUGGG**-**CUGAA**CAGAGGG
MF184921*		CUACACAC**GUACUACAAUGGG**-**CUGAA**CAGAAGG
AB494837		CUACACACGUACUACAAUGGG-GCGAACAGAGGG
MF184871*	*Quinella ovalis*	CUACACAC**GUACUACAAUGGG**-**CUGAA**CAAAAGG
MF184873*		CUACACAC**GUACUACAAUGGG**-**CUGAA**CAAAAGG
MF184872*		CUACACAC**GUACUACAAUGGG**-**CUGAA**CAAAAGG
MF184874*		CUACACAC**GUACUACAAUGGG**-**CUGAA**CAAAAGG
MF184899*		CUACACAC**GUACUACAAUGGG**-**CUGAA**CAGAAGG
MF184876*		CUACACAC**GUACUACAAUGGG**-**CUGAA**CAAAAGG
M62701		CUACACAC**GUACUACAAUGGG**-**CUGAA**CAAAAGG
MF184889*		CUACACAC**GUACUACAAUGGG**-**CUGAA**CAGAAGG
AB494823		CUACACAC**GUACUACAAUGGG**-**CUGAA**CAGAAGG
MF184897*	*Quinella* candidate species 1	CUACACAC**GUACUACAAUGGG**-**CUGAA**CAGAAGG
MF184898*		CUACACAC**GUACUACAAUGGG**-**CUGAA**CAGAAGG
MF184895*		CUACACAC**GUACUACAAUGGG**-**CUGAA**CAGAAGG
MF184896*		CUACACAC**GUACUACAAUGGG**-**CUGAA**CAGAAGG
MF184894*		CUACACAC**GUACUACAAUGGG**-**CUGAA**CAGAAGG
MF184914*	*Quinella* candidate species 2	CUACACAC**GUACUACAAUGGG**-**CUGAA**CAGAAGG
MF184915*		CUACACAC**GUACUACAAUGGG**-**CUGAA**CAGAAGG
AB494931		CUACACAC**GUACUACAAUGGG**-**CUGAA**CAGAAGG
DQ673510	*Quinella* candidate species 7	CUACACAC**GUACUACAAUGGG**-**CUGAA**CAGAGGG
DQ673569		CUACACAC**GUACUACAAUGGG**-**CUGAA**CAGAGGG
DQ673570		CUACACAC**GUACUACAAUGGG**-**CUGAA**CAGAGGG
EF436320		CUACACAC**GUACUACAAUGGG**-**CUGAA**CAGAGGG
EF436424		CUACACAC**GUACUACAAUGGG**-**CUGAA**CAGAAGG

Sequence (NCBI accession no.)	Sequence clusters[a]	Probe target region
EF436425	*Quinella* candidate species 5	CUACACAC**GUACUACAAUGGG**-**CUGAA**CAGAAGG
MF184890*		CUACACAC**GUACUACAAUGGG**-**CUGAA**CAGAAGG
EF436426		CUACACAC**GUACUACAAUGGG**-**CUGAA**CAGAAGG
MF184879*		CUACACAC**GUACUACAAUGGG**-**CUGAA**CAAAAGG
DQ673559<br>DQ673560<br>DQ673499	*Selenomonadaceae* candidate genus 1 species 1	CUACACACGUACUACAAUGGGACGGAACAAAGGG<br>CUACACACGUACUACAAUGGGACGGAACAAAGGG<br>CUACACACGUACUACAAUGGGACGGAACAAAGGG
MF184882*<br>DQ673561	*Selenomonadaceae* candidate genus 1 species 2	CUACACACGUACUACAAUGGGACGGAACAAAGGG<br>CUACACACGUACUACAAUGGGACGGAACAAAGGG

[a]Clusters without names are singleton sequences, shown in Figure 1.

Supplementary Table 4. Read number and sequence quality from DNA sequencing data.

Sample	Description	Paired reads	Total nucleotides	Quality score (%Q30)[a]
1	Pooled from 12 sheep	6,997,737	4.199×10^9	84.2
2	Pooled from one sheep from two measuring rounds	5,688,064	3.413×10^9	85.0
3	Single rumen sample from one sheep	4,541,407	2.725×10^9	84.1

[a]%Q30, represents the percentage of bases with a quality score of at least 30 (i.e., an inferred base call accuracy of 99.9%).

Supplementary Table 5. Assemblies and bins generated from metagenomic DNA sequence data.

Assembly details	Sample 1	Sample 2	Sample 3
Numbers of paired reads	6,997,737	5,688,064	4,541,407
Numbers of contigs	290,401	277,479	416,020
Numbers of contigs >1000 bp	69,711	67,624	87,783
Largest contig (bp)	269,413	637,526	228,730
N50[a]	1,029	1,053	904
N75[a]	664	673	614
GC content (mol%)	47.26	44.42	44.97
Number of bins generated[b]	31	33	21
Number of bins >1 Mbp	12	13	9

[a]N50 and N75 are the contig lengths (bp) such that longer or equal length contigs account for 50% or 75% of the bases in the assembly[129].

[b]Using MetaBAT[130].

Supplementary Table 6. Steps to generate *Quinella* genome bins from metagenomic DNA sequence data. The bins generated in step 1 (Supplementary Table 5) were retained or removed using a series of sequential analyses (steps 2 to 7).

Steps	Description/filter	Total bins remaining
1	Binning with MetaBAT	85
2	Bins with >1,000,000 bp size	34
3	Bins containing 16S rRNA gene sequences retained	23
4	Bins originating from members of the family *Selenomonadaceae* retained (>70 % of the bins residues identified as belonging to *Selenomonadaceae*, using Amphora markers)	20
5	Bins containing *Quinella* 16S rRNA genes retained (BLAST and phylogenetic tree construction)	13
6	Bin with >90% completeness and <11% contamination retained	4
7	Bins with amplified full length 16S rRNA gene sequences identified as *Quinella* retained (BLAST and phylogenetic tree construction)	4

Supplementary Table 7. Lineage-specific quality control assessment of *Quinella* genome bins using CheckM.

Characteristic	*Quinella* genome bins												
	1Q2	SR1Q7[a]	SR1Q5[a]	1Q1	1Q9	2Q18	2Q15	SR2Q5[a]	2Q13	SR3Q1[a]	3Q5	SR3Q16	SR3Q12
No. of genomes used[b]	5449	100	100	100	100	5656	5449	100	100	5449	5656	5449	100
No. of markers used	104	295	295	295	295	56	104	295	295	104	56	104	295
Lineage-specific marker sets	58	158	158	158	158	24	58	158	158	58	24	58	158
No. of markers absent	0	18	30	87	191	0	3	17	146	6	0	18	80
Single copy markers	3	244	247	198	90	0	43	277	117	75	0	43	112
Duplicate markers	7	33	18	9	14	8	32	1	31	10	0	24	51
Triplicate markers	17	0	0	1	0	15	20	0	1	6	0	15	39
Four copies of markers	25	0	0	0	0	14	6	0	0	6	0	3	12
Markers present in 5+ copies	52	0	0	0	0	19	0	0	0	1	56	1	1
Genome completeness	100	94.2	91.0	76.0	28.8	100	96.6	92.7	43.9	91.4	100	78.6	76.1
Genome contamination	48.5	8.7	5.7	4.8	3.6	329.2	68.0	0.2	10.7	10.3	672.8	65.1	65.6
Strain heterogeneity	57.0	48.5	77.8	41.7	57.1	69.4	71.1	0.0	79.4	78.4	76.6	48.5	29.6
16S rRNA gene length (bp)	793[c]	426	122	966	444	915[c]	134[c]	399	136	373	976[c]	124	124

[a]Genome bins coloured in green were the shortlisted *Quinella* genome bins used for further analysis.

[b]Number of reference genomes used to identify lineages (root, 5656; Bacteria, 5449; *Firmicutes*, 100).

[c]Multiple 16S rRNA sequences were found, but only the length of sequences with an E-value of 0 when compared to known *Quinella* 16S rRNA genes are listed

Supplementary Table 8. Primers used to amplify 16S rRNA genes from DNA of *Quinella*-enriched samples.

Quinella genome bin and contig identifier (bin_contig)	Primer	Primer sequence (5'- 3')	Sequence start position	Sequence end position	Primer length (bp)	G+C (mol%)	Melting temperature (°C)	Self-complimentarity (bp)	Predicted product size in combination with 1492R[a] (bp)
SR1Q5_5057819	9354F	CTCGACGTTCT TAATCTTCG	9,354	9,373	20	45	54.1	4	1851
SR1Q7_5055757	19382F	ACGACGATAA TCCTGTGG	19,382	19,399	18	50	53.8	5	2194
SR2Q5_4645198	111625F	GATACGTCAG GTCATAGC	111,625	111,642	18	50	51.7	4	2026
SR3Q1_4968023	24448F	TGAATCAGCG AATAGAGC	24,448	24,465	18	44.4	51.8	5	2390

[a]Bacterial 16S rRNA gene universal primer 1492R (Supplementary Table 19) was used as the reverse primer.

Supplementary Table 9. Similarity matrix of cloned sequences. The upper triangle of the matrix gives the similarity (percentage of identical nucleotides) between full lengths of the cloned sequences (colour code white to red, low to high). The lower triangle of the matrix gives similarities between 16S rRNA gene regions of the cloned sequences (colour code white to purple, low to high). The three columns on the right give the similarities of the parts of the cloned sequences with their respective contigs in the genome bins.

		Similarity between cloned sequences (%)																				Similarity with overlapping region of the genome bins (%)		
Genome bins		SR1Q5					SR1Q7					SR2Q5					SR3Q1					With full overlapping region	With 16S rRNA gene only	Other than 16S rRNA gene region
	Clone no.	3	4	9	10	11	38	46	47	49	54	66	67	74	81	87	107	111	116	119	120			
SR1Q5	3		99.6	99.4	99.5	99.1	81.2	81.0	80.7	81.4	82.4	82.6	82.6	82.0	82.4	81.5	82.0	81.7	82.3	82.1	81.9	100.0	100.0	100.0
	4	99.7		99.6	99.5	99.0	81.3	81.1	80.8	81.3	82.3	82.4	82.5	82.0	82.3	81.4	82.1	81.8	82.3	82.1	82.0	100.0	100.0	100.0
	9	99.6	99.6		99.5	98.9	81.2	81.0	80.7	81.3	82.2	82.5	82.6	82.1	82.4	81.5	82.1	81.8	82.3	82.1	82.1	100.0	100.0	100.0
	10	99.8	99.7	99.5		99.0	81.2	81.0	80.6	81.2	82.2	82.5	82.6	82.1	82.4	81.5	82.1	81.8	82.2	82.0	82.0	100.0	100.0	100.0
	11	99.7	99.5	99.4	99.6		81.0	80.8	80.5	81.1	82.0	82.2	82.3	82.0	82.1	81.7	81.8	81.5	82.1	81.9	81.8	100.0	100.0	100.0
SR1Q7	38	94.4	94.2	94.1	94.3	94.1		94.6	93.7	99.6	96.8	79.8	80.1	80.0	81.1	79.5	75.3	75.1	74.2	75.3	75.3	99.0	99.5	98.6
	46	94.3	94.1	93.9	94.1	94.0	99.0		97.0	94.6	91.9	79.6	79.8	79.7	80.8	79.2	76.0	75.8	74.9	76.0	76.1	92.7	99.3	88.9
	47	94.4	94.3	94.1	94.3	94.1	99.4	99.0		93.6	91.0	79.2	79.4	79.4	80.4	78.9	75.9	75.6	74.8	75.9	76.0	90.5	99.8	85.2
	49	94.5	94.3	94.1	94.3	94.2	99.9	99.0	99.4		96.9	80.0	80.1	80.2	81.2	79.5	75.2	74.9	74.1	75.2	75.2	99.1	99.8	98.9
	54	94.4	94.2	94.1	94.3	94.1	99.9	99.0	99.4	99.9		81.0	81.0	80.9	81.2	80.1	74.1	73.7	74.0	74.1	74.0	99.0	99.8	98.6
SR2Q5	66	94.6	94.4	94.3	94.5	94.4	94.6	94.7	94.6	94.6	94.6		99.6	98.7	99.5	98.2	79.1	78.5	79.1	79.1	79.0	99.1	99.4	98.9
	67	94.5	94.3	94.2	94.4	94.3	94.6	94.6	94.6	94.6	94.6	99.8		98.9	99.6	98.2	79.1	78.7	79.2	79.1	79.1	99.2	99.2	99.1
	74	94.5	94.4	94.3	94.3	94.3	94.5	94.5	94.5	94.5	94.5	99.8	99.6		99.0	98.5	79.0	78.5	79.4	79.1	79.0	99.1	99.5	98.7
	81	94.5	94.3	94.2	94.4	94.3	94.5	94.5	94.5	94.5	94.5	99.8	99.7	99.7		98.6	80.1	79.7	79.7	80.0	80.1	98.9	99.2	98.6
	87	94.5	94.3	94.2	94.4	94.4	94.5	94.5	94.5	94.5	94.5	99.8	99.7	99.9	99.7		78.3	77.8	78.8	78.3	78.3	99.8	99.5	100.0
SR3Q1	107	94.2	94.0	93.9	94.1	93.9	95.5	95.4	95.4	95.6	95.5	94.3	94.3	94.1	94.2	94.2		98.9	99.0	99.5	99.5	99.7	99.5	99.8
	111	93.8	93.6	93.4	93.6	93.5	95.0	94.9	94.9	95.0	95.0	93.8	93.8	93.6	93.7	93.7	99.2		98.4	98.9	98.9	99.8	99.7	99.8
	116	93.6	93.5	93.4	93.5	93.4	94.9	94.9	94.9	95.0	94.9	93.7	93.7	93.7	93.6	93.7	98.9	98.4		99.1	99.1	99.8	99.7	99.8
	119	94.3	94.1	94.0	94.2	94.1	95.6	95.6	95.6	95.7	95.6	94.4	94.4	94.3	94.3	94.3	99.6	99.0	99.0		99.6	99.8	100.0	99.7
	120	94.3	94.1	94.0	94.2	94.1	95.6	95.6	95.6	95.7	95.6	94.4	94.4	94.3	94.3	94.3	99.6	99.0	99.0	99.7		99.9	99.7	100.0

Supplementary Table 10. ***Quinella*** **genome bin statistics**.

Attribute	*Quinella* genome bins			
	SR1Q5	SR1Q7	SR2Q5	SR3Q1
Genome size (bp)	2,125,473	2,584,672	1,821,931	2,614,227
DNA coding (%)	92.8	90.2	92.8	91.7
G + C (mol%)	49.0	52.9	56.0	49.1
Number of contigs	132	169	42	68
Total number of ORFs	2,067	2,445	1728	2521
Number of rRNAs	6	6	10	6
Number of tRNAs	49	33	43	34
Genes assigned to COGs	1769	2101	1543	2152
Genes with Pfam domains	1770	2030	1520	2130
Genes with signal peptides	184	203	171	222
Genes with transmembrane helices	426	475	363	508
CAZymes	68	83	62	93
CRISPR	0	1	0	2

Supplementary Table 11. Functional classification of the predicted genes in the four *Quinella* genome bins based on the clusters of orthologous proteins (COGs) database.[131]

COG categories	Description	SR1Q5		SR1Q7		SR2Q5		SR3Q1	
		Count	Percent	Count	Percent	Count	Percent	Count	Percent
C	Energy production and conversion	91	4.4	110	4.5	90	5.2	115	4.6
D	Cell cycle control, cell division, chromosome partitioning	51	2.5	51	2.1	41	2.4	52	2.1
E	Amino acid transport and metabolism	157	7.6	182	7.4	147	8.5	184	7.3
F	Nucleotide transport and metabolism	50	2.4	67	2.7	52	3.0	56	2.2
G	Carbohydrate transport and metabolism	110	5.3	158	6.5	120	6.9	159	6.3
H	Coenzyme transport and metabolism	111	5.4	116	4.7	100	5.8	111	4.4
I	Lipid transport and metabolism	54	2.6	71	2.9	55	3.2	80	3.2
J	Translation, ribosomal structure and biogenesis	171	8.3	198	8.1	172	9.9	226	9.0
K	Transcription	83	4.0	91	3.7	66	3.8	109	4.3
L	Replication, recombination and repair	83	4.0	92	3.8	60	3.5	89	3.5
M	Cell wall/membrane/envelope biogenesis	172	8.3	234	9.6	163	9.4	221	8.8
N	Cell motility	54	2.6	74	3.0	68	3.9	72	2.9
O	Posttranslational modification, protein turnover, chaperones	100	4.8	103	4.2	77	4.5	96	3.8
P	Inorganic ion transport and metabolism	58	2.8	63	2.6	45	2.6	78	3.1

COG categories	Description	SR1Q5		SR1Q7		SR2Q5		SR3Q1	
		Count	Percent	Count	Percent	Count	Percent	Count	Percent
Q	Secondary metabolites biosynthesis, transport and catabolism	51	2.5	73	3.0	32	1.9	77	3.1
R	General function prediction only	196	9.5	221	9.0	142	8.2	230	9.1
S	Function unknown	99	4.8	122	5.0	84	4.9	120	4.8
T	Signal transduction mechanisms	119	5.8	135	5.5	103	6.0	140	5.6
U	Intracellular trafficking, secretion, and vesicular transport	44	2.1	39	1.6	41	2.4	51	2.0
V	Defence mechanisms	68	3.3	88	3.6	42	2.4	85	3.4
W	Extracellular structures	20	1.0	17	0.7	16	0.9	28	1.1
X	Phage-derived proteins	33	1.6	29	1.2	4	0.2	28	1.1
Z	Cytoskeleton	1	0.05	0	0	0	0	1	0.04
	No COG category assigned	298	14.4	344	14.1	185	10.7	369	14.6
	Total number of genes[a]	2067		2445		1728		2521	

[a]Total gene numbers do not add up, as some genes were assigned to more than one COG category and so counted more than once.

Supplementary Table 12. CAZyme counts in the *Quinella* genome bins. E-values < 1E–18 and coverage > 0.35 were considered as good matches[132], and genes meeting both of these criteria were considered to be potential CAZymes.

Carbohydrate-active enzymes (CAZymes) classes[a]	*Quinella* genome bins				
	SR1Q5	SR1Q7	SR2Q5	SR3Q1	Total unique CAZymes
Auxiliary activities (AAs)	0	0	1	0	1
Carbohydrate esterases (CEs)	13 (1)	8	19 (1)	16 (3)[b]	5
Glycoside hydrolases (GHs)	13	15 (1)	12 (1)	18 (2)	4
Carbohydrate-binding modules (CBMs)	0	0	0	0	0
Glycosyl transferases (GTs)	33 (2)	58 (4)	39 (1)	53 (4)	11
Polysaccharide lyases (PLs)	0	0	0	0	0

[a]See Supplementary Table 13 for information of individual genes identified in each of the CAZymes classes.

[b]Values in parentheses represent unique CAZYmes present only in one bin out of four.

Supplementary Table 13. CAZymes found in *Quinella* genome bins. CAZymes with E-values < 1E−18 and coverage > 0.35 were considered as good matches[132] and are listed in this table.

Quinella genome bin genes	CAZyme families match	GAMOLA2 annotation match
SR2Q5_1268	AA4	Glycolate oxidase, subunit GlcD
SR1Q5_1058	CE10	Carboxylesterase
SR1Q7_161	CE10	Esterase, PHB depolymerase family
SR3Q1_1207	CE10	Esterase, PHB depolymerase family
SR1Q7_2225	CE10	Hypothetical protein
SR2Q5_1666	CE10	Hypothetical protein
SR2Q5_568	CE10	Hypothetical protein
SR2Q5_577	CE10	Hypothetical protein
SR3Q1_1020	CE10	Hypothetical protein
SR3Q1_1255	CE10	Hypothetical protein
SR3Q1_1456	CE10	Hypothetical protein
SR3Q1_1021	CE10	Polyketide synthase-associated domain
SR3Q1_2021	CE10	Putative carboxylesterase
SR1Q5_1059	CE10	Tat (twin-arginine translocation) pathway signal sequence
SR1Q5_1060	CE10	Tat (twin-arginine translocation) pathway signal sequence
SR1Q5_1061	CE10	Tat (twin-arginine translocation) pathway signal sequence
SR1Q5_1062	CE10	Tat (twin-arginine translocation) pathway signal sequence
SR1Q5_1859	CE10	Tat (twin-arginine translocation) pathway signal sequence
SR1Q5_1860	CE10	Tat (twin-arginine translocation) pathway signal sequence
SR1Q7_1258	CE10	Tat (twin-arginine translocation) pathway signal sequence
SR3Q1_1018	CE10	Tat (twin-arginine translocation) pathway signal sequence
SR3Q1_1019	CE10	Tat (twin-arginine translocation) pathway signal sequence
SR3Q1_2386	CE10	Tat (twin-arginine translocation) pathway signal sequence
SR1Q5_1783	CE11	UDP-3-*O*-[3-hydroxymyristoyl] *N*-acetylglucosamine deacetylase, LpxC
SR1Q7_242	CE11	UDP-3-*O*-[3-hydroxymyristoyl] *N*-acetylglucosamine deacetylase, LpxC
SR2Q5_109	CE11	UDP-3-*O*-[3-hydroxymyristoyl] *N*-acetylglucosamine deacetylase, LpxC
SR3Q1_1227	CE11	UDP-3-*O*-[3-hydroxymyristoyl] *N*-acetylglucosamine deacetylase, LpxC
SR1Q5_1463	CE4	Poly-β-1,6-*N*-acetyl-D-glucosamine *N*-deacetylase pgab, pgab
SR1Q5_1518	CE4	Poly-β-1,6-*N*-acetyl-D-glucosamine *N*-deacetylase pgab, pgab
SR1Q5_850	CE4	Poly-β-1,6-*N*-acetyl-D-glucosamine *N*-deacetylase pgab, pgab
SR1Q7_1593	CE4	Poly-β-1,6-*N*-acetyl-D-glucosamine *N*-deacetylase pgab, pgab
SR1Q7_2096	CE4	Poly-β-1,6-*N*-acetyl-D-glucosamine *N*-deacetylase pgab, pgab
SR1Q7_503	CE4	Poly-β-1,6-*N*-acetyl-D-glucosamine *N*-deacetylase pgab, pgab

Quinella genome bin genes	CAZyme families match	GAMOLA2 annotation match
SR2Q5_126	CE4	Poly-β-1,6-*N*-acetyl-D-glucosamine *N*-deacetylase pgab, pgab
SR2Q5_1278	CE4	Poly-β-1,6-*N*-acetyl-D-glucosamine *N*-deacetylase pgab, pgab
SR2Q5_935	CE4	Poly-β-1,6-*N*-acetyl-D-glucosamine *N*-deacetylase pgab, pgab
SR3Q1_1283	CE4	Poly-β-1,6-*N*-acetyl-D-glucosamine *N*-deacetylase pgab, pgab
SR3Q1_1994	CE4	Poly-β-1,6-*N*-acetyl-D-glucosamine *N*-deacetylase pgab, pgab
SR3Q1_943	CE4	Poly-β-1,6-*N*-acetyl-D-glucosamine *N*-deacetylase pgab, pgab
SR1Q5_1093	CE4	Polysaccharide deacetylase family sporulation protein pdab, pdab
SR2Q5_1068	CE4	Polysaccharide deacetylase family sporulation protein pdab, pdab
SR3Q1_923	CE4	Polysaccharide deacetylase family sporulation protein pdab, pdab
SR3Q1_371	CE4	Putative urate catabolism protein
SR1Q5_2001	CE9	*N*-acetylglucosamine-6-phosphate deacetylase, naga
SR1Q7_202	CE9	*N*-acetylglucosamine-6-phosphate deacetylase, naga
SR2Q5_369	CE9	*N*-acetylglucosamine-6-phosphate deacetylase, naga
SR3Q1_743	CE9	*N*-acetylglucosamine-6-phosphate deacetylase, naga
SR1Q5_297	GH1	β-galactosidase
SR1Q7_1166	GH1	β-galactosidase
SR1Q7_71	GH1	β-galactosidase
SR2Q5_701	GH1	β-galactosidase
SR3Q1_11	GH1	β-galactosidase
SR2Q5_1651	GH13	1,4-α-glucan branching enzyme, glgb
SR3Q1_1695	GH13	1,4-α-glucan branching enzyme, glgb
SR3Q1_2497	GH13	1,4-α-glucan branching enzyme, glgb
SR3Q1_445	GH13	1,4-α-glucan branching enzyme, glgb
SR1Q5_1565	GH13	1,4-α-glucan branching enzyme, glgb
SR1Q7_1628	GH13	1,4-α-glucan branching enzyme, glgb
SR1Q7_209	GH13	1,4-α-glucan branching enzyme, glgb
SR2Q5_1470	GH13	1,4-α-glucan branching enzyme, glgb
SR2Q5_1500	GH13	α-phosphotrehalase, trec
SR3Q1_1971	GH13	α-phosphotrehalase, trec
SR1Q7_1085	GH13	α-phosphotrehalase, trec
SR1Q5_127	GH13	α-phosphotrehalase, trec
SR2Q5_1718	GH23	Lytic murein transglycosylase B, mltb
SR1Q5_1599	GH23	Lytic transglycosylase
SR1Q7_2199	GH23	Lytic transglycosylase
SR2Q5_1168	GH23	Lytic transglycosylase
SR3Q1_342	GH23	Lytic transglycosylase

Quinella genome bin genes	CAZyme families match	GAMOLA2 annotation match
SR1Q5_965	GH23	Transglycosylase
SR1Q7_1734	GH23	Transglycosylase
SR2Q5_680	GH23	Transglycosylase
SR3Q1_1032	GH23	Transglycosylase
SR1Q7_77	GH23	Transglycosylase SLT domain protein
SR1Q5_1231	GH25	Hypothetical protein
SR3Q1_2221	GH25	Hypothetical protein
SR3Q1_925	GH25	Hypothetical protein
SR3Q1_1046	GH25	Phosphoglycolate phosphatase, bacterial, gph
SR1Q5_759	GH3	β-glucosidase-related glycosidases
SR1Q7_451	GH3	β -glucosidase-related glycosidases
SR2Q5_479	GH3	β -glucosidase-related glycosidases
SR3Q1_185	GH3	β -glucosidase-related glycosidases
SR1Q5_1015	GH32	Sucrose-6-phosphate hydrolase
SR1Q5_52	GH32	Sucrose-6-phosphate hydrolase
SR1Q5_723	GH32	Sucrose-6-phosphate hydrolase
SR1Q5_871	GH32	Sucrose-6-phosphate hydrolase
SR1Q7_1180	GH32	Sucrose-6-phosphate hydrolase
SR1Q7_1646	GH32	Sucrose-6-phosphate hydrolase
SR1Q7_186	GH32	Sucrose-6-phosphate hydrolase
SR1Q7_796	GH32	Sucrose-6-phosphate hydrolase
SR2Q5_1090	GH32	Sucrose-6-phosphate hydrolase
SR2Q5_590	GH32	Sucrose-6-phosphate hydrolase
SR3Q1_280	GH32	Sucrose-6-phosphate hydrolase
SR3Q1_485	GH32	Sucrose-6-phosphate hydrolase
SR3Q1_733	GH32	Sucrose-6-phosphate hydrolase
SR3Q1_1579	GH4	6-phospho-α-glucosidase
SR1Q5_1094	GH77	4-α-glucanotransferase, malq
SR1Q7_2201	GH77	4-α-glucanotransferase, malq
SR2Q5_1065	GH77	4-α-glucanotransferase, malq
SR3Q1_1217	GH77	4-α-glucanotransferase, malq
SR3Q1_2458	GH77	4-α-glucanotransferase, malq
SR1Q7_1014	GH84	Hypothetical protein
SR1Q5_1310	GH84	Putative *O*- GlcNAcase
SR2Q5_292	GH84	Putative *O*-GlcNAcase
SR3Q1_2032	GH84	Putative *O*-GlcNAcase

Quinella genome bin genes	CAZyme families match	GAMOLA2 annotation match
SR1Q7_60	GT11	Poly-β-1,6 *N*-acetyl-D-glucosamine synthase, pgac
SR1Q7_61	GT11	Poly-β-1,6 *N*-acetyl-D-glucosamine synthase, pgac
SR1Q5_5	GT17	β-1,4-*N*-acetylgalactosaminyltransferase
SR1Q5_827	GT17	Hypothetical protein
SR1Q7_1137	GT17	Hypothetical protein
SR1Q7_1141	GT17	Hypothetical protein
SR1Q7_1427	GT17	Hypothetical protein
SR2Q5_1396	GT17	Hypothetical protein
SR1Q5_1742	GT19	Lipid-A-disaccharide synthase, lpxb
SR1Q7_692	GT19	Lipid-A-disaccharide synthase, lpxb
SR2Q5_978	GT19	Lipid-A-disaccharide synthase, lpxb
SR3Q1_759	GT19	Lipid-A-disaccharide synthase, lpxb
SR2Q5_1672	GT2	Colanic acid biosynthesis glycosyltransferase wcaa, wcaa
SR3Q1_324	GT2	Colanic acid biosynthesis glycosyltransferase wcaa, wcaa
SR1Q7_1724	GT2	Glycosyltransferase domain
SR3Q1_291	GT2	Glycosyltransferase domain
SR1Q7_1906	GT2	Mycofactocin system glycosyltransferase, mftf
SR1Q7_2396	GT2	Mycofactocin system glycosyltransferase, mftf
SR2Q5_337	GT2	Mycofactocin system glycosyltransferase, mftf
SR2Q5_811	GT2	Mycofactocin system glycosyltransferase, mftf
SR2Q5_813	GT2	Mycofactocin system glycosyltransferase, mftf
SR3Q1_1204	GT2	Mycofactocin system glycosyltransferase, mftf
SR1Q5_1122	GT2	*N*-acetyl-α-D-glucosaminyl L-malate synthase bsha, bsha
SR3Q1_549	GT2	*N*-acetyl-α-D-glucosaminyl L-malate synthase bsha, bsha
SR1Q5_101	GT2	Poly-β-1,6 *N*-acetyl-D-glucosamine synthase, pgac
SR1Q5_1101	GT2	Poly-β-1,6 *N*-acetyl-D-glucosamine synthase, pgac
SR1Q5_1602	GT2	Poly-β-1,6 *N*-acetyl-D-glucosamine synthase, pgac
SR1Q5_1745	GT2	Poly-β-1,6 *N*-acetyl-D-glucosamine synthase, pgac
SR1Q5_209	GT2	Poly-β-1,6 *N*-acetyl-D-glucosamine synthase, pgac
SR1Q5_265	GT2	Poly-β-1,6 *N*-acetyl-D-glucosamine synthase, pgac
SR1Q5_266	GT2	Poly-β-1,6 *N*-acetyl-D-glucosamine synthase, pgac
SR1Q5_578	GT2	Poly-β-1,6 *N*-acetyl-D-glucosamine synthase, pgac
SR1Q5_579	GT2	Poly-β-1,6 *N*-acetyl-D-glucosamine synthase, pgac
SR1Q5_656	GT2	Poly-β-1,6 *N*-acetyl-D-glucosamine synthase, pgac
SR1Q5_986	GT2	Poly-β-1,6 *N*-acetyl-D-glucosamine synthase, pgac
SR1Q7_1197	GT2	Poly-β-1,6 *N*-acetyl-D-glucosamine synthase, pgac

Quinella genome bin genes	CAZyme families match	GAMOLA2 annotation match
SR1Q7_1410	GT2	Poly-β-1,6 *N*-acetyl-D-glucosamine synthase, pgac
SR1Q7_1693	GT2	Poly-β-1,6 *N*-acetyl-D-glucosamine synthase, pgac
SR1Q7_1782	GT2	Poly-β-1,6 *N*-acetyl-D-glucosamine synthase, pgac
SR1Q7_1783	GT2	Poly-β-1,6 *N*-acetyl-D-glucosamine synthase, pgac
SR1Q7_1784	GT2	Poly-β-1,6 *N*-acetyl-D-glucosamine synthase, pgac
SR1Q7_1786	GT2	Poly-β-1,6 *N*-acetyl-D-glucosamine synthase, pgac
SR1Q7_1974	GT2	Poly-β-1,6 *N*-acetyl-D-glucosamine synthase, pgac
SR1Q7_2395	GT2	Poly-β-1,6 *N*-acetyl-D-glucosamine synthase, pgac
SR1Q7_690	GT2	Poly-β-1,6 *N*-acetyl-D-glucosamine synthase, pgac
SR1Q7_819	GT2	Poly-β-1,6 *N*-acetyl-D-glucosamine synthase, pgac
SR1Q7_820	GT2	Poly-β-1,6 *N*-acetyl-D-glucosamine synthase, pgac
SR1Q7_978	GT2	Poly-β-1,6 *N*-acetyl-D-glucosamine synthase, pgac
SR2Q5_1075	GT2	Poly-β-1,6 *N*-acetyl-D-glucosamine synthase, pgac
SR2Q5_1116	GT2	Poly-β-1,6 *N*-acetyl-D-glucosamine synthase, pgac
SR2Q5_1118	GT2	Poly-β-1,6 *N*-acetyl-D-glucosamine synthase, pgac
SR2Q5_1120	GT2	Poly-β-1,6 *N*-acetyl-D-glucosamine synthase, pgac
SR2Q5_222	GT2	Poly-β-1,6 *N*-acetyl-D-glucosamine synthase, pgac
SR2Q5_418	GT2	Poly-β-1,6 *N*-acetyl-D-glucosamine synthase, pgac
SR2Q5_645	GT2	Poly-β-1,6 *N*-acetyl-D-glucosamine synthase, pgac
SR2Q5_646	GT2	Poly-β-1,6 *N*-acetyl-D-glucosamine synthase, pgac
SR2Q5_809	GT2	Poly-β-1,6 *N*-acetyl-D-glucosamine synthase, pgac
SR2Q5_812	GT2	Poly-β-1,6 *N*-acetyl-D-glucosamine synthase, pgac
SR2Q5_841	GT2	Poly-β-1,6 *N*-acetyl-D-glucosamine synthase, pgac
SR3Q1_1652	GT2	Poly-β-1,6 *N*-acetyl-D-glucosamine synthase, pgac
SR3Q1_1996	GT2	Poly-β-1,6 *N*-acetyl-D-glucosamine synthase, pgac
SR3Q1_216	GT2	Poly-β-1,6 *N*-acetyl-D-glucosamine synthase, pgac
SR3Q1_225	GT2	Poly-β-1,6 *N*-acetyl-D-glucosamine synthase, pgac
SR3Q1_755	GT2	Poly-β-1,6 *N*-acetyl-D-glucosamine synthase, pgac
SR3Q1_756	GT2	Poly-β-1,6 *N*-acetyl-D-glucosamine synthase, pgac
SR3Q1_84	GT2	Poly-β-1,6 *N*-acetyl-D-glucosamine synthase, pgac
SR3Q1_86	GT2	Poly-β-1,6 *N*-acetyl-D-glucosamine synthase, pgac
SR3Q1_89	GT2	Poly-β-1,6 *N*-acetyl-D-glucosamine synthase, pgac
SR3Q1_917	GT2	Poly-β-1,6 *N*-acetyl-D-glucosamine synthase, pgac
SR1Q7_1785	GT2	Putative glycosyltransferase, exosortase G- associated
SR3Q1_1225	GT2	Putative glycosyltransferase, exosortase G- associated
SR3Q1_215	GT2	Putative glycosyltransferase, exosortase G- associated

Quinella genome bin genes	CAZyme families match	GAMOLA2 annotation match
SR1Q7_768	GT2	Sugar transferase, PEP-CTERM/epsh1 system associated
SR2Q5_6	GT2	Sugar transferase, PEP-CTERM/epsh1 system associated
SR2Q5_818	GT2	Transferase 2, rsam/selenodomain-associated
SR1Q5_1255	GT26	Glycosyltransferase, wecb/taga/cpsf family
SR1Q7_1963	GT26	Glycosyltransferase, wecb/taga/cpsf family
SR2Q5_1414	GT26	Glycosyltransferase, wecb/taga/cpsf family
SR3Q1_1268	GT26	Glycosyltransferase, wecb/taga/cpsf family
SR1Q5_1278	GT28	Murg transferase
SR1Q5_718	GT28	Murg transferase
SR1Q7_1960	GT28	Murg transferase
SR1Q7_846	GT28	Murg transferase
SR2Q5_1515	GT28	Murg transferase
SR3Q1_1265	GT28	Murg transferase
SR1Q7_1977	GT30	Tetraacyldisaccharide 4'-kinase
SR1Q7_331	GT30	Tetraacyldisaccharide 4'-kinase
SR1Q5_69	GT30	Tetraacyldisaccharide 4'-kinase, lpxk
SR1Q7_22	GT30	Tetraacyldisaccharide 4'-kinase, lpxk
SR2Q5_1349	GT30	Tetraacyldisaccharide 4'-kinase, lpxk
SR3Q1_505	GT30	Tetraacyldisaccharide 4'-kinase, lpxk
SR3Q1_1203	GT32	Hypothetical protein
SR1Q7_210	GT35	Glycogen/starch/α-glucan phosphorylases, glgp
SR2Q5_1471	GT35	Glycogen/starch/α-glucan phosphorylases, glgp
SR3Q1_1696	GT35	Glycogen/starch/α-glucan phosphorylases, glgp
SR1Q7_1061	GT4	Accessory Sec system glycosylation protein gtfa, gtfa
SR1Q5_1054	GT4	*N*-acetyl-α-D-glucosaminyl L-malate synthase bsha, bsha
SR1Q5_1216	GT4	*N*-acetyl-α-D-glucosaminyl L-malate synthase bsha, bsha
SR1Q7_1350	GT4	*N*-acetyl-α-D-glucosaminyl L-malate synthase bsha, bsha
SR1Q5_68	GT4	Sugar transferase, PEP-CTERM/epsh1 system associated
SR1Q7_604	GT4	Sugar transferase, PEP-CTERM/epsh1 system associated
SR2Q5_820	GT4	Sugar transferase, PEP-CTERM/epsh1 system associated
SR3Q1_1169	GT4	Sugar transferase, PEP-CTERM/epsh1 system associated
SR1Q5_82	GT41	Hypothetical protein
SR1Q5_84	GT41	Hypothetical protein
SR1Q7_1076	GT41	Hypothetical protein
SR1Q7_258	GT41	Hypothetical protein
SR2Q5_1369	GT41	Hypothetical protein

Quinella genome bin genes	CAZyme families match	GAMOLA2 annotation match
SR3Q1_1043	GT41	Hypothetical protein
SR3Q1_1182	GT41	Hypothetical protein
SR3Q1_1183	GT41	Hypothetical protein
SR1Q5_1234	GT41	Hypothetical protein HMPREF9166
SR1Q5_1018	GT41	Peptide *S*-glycosyltransferase, suns family, suns
SR1Q7_962	GT41	Peptide *S*-glycosyltransferase, suns family, suns
SR2Q5_907	GT41	Peptide *S*-glycosyltransferase, suns family, suns
SR3Q1_487	GT41	Peptide *S*-glycosyltransferase, suns family, suns
SR1Q7_792	GT41	Poly-β-1,6 *N*-acetyl-D-glucosamine export porin pgaa, pgaa
SR1Q7_2089	GT41	Putative PEP-CTERM system TPR-repeat lipoprotein, prst
SR2Q5_1003	GT41	Putative PEP-CTERM system TPR-repeat lipoprotein, prst
SR2Q5_306	GT41	Putative PEP-CTERM system TPR-repeat lipoprotein, prst
SR3Q1_1574	GT41	Surface carbohydrate biosynthesis protein
SR3Q1_1186	GT41	Type VI secretion lipoprotein, VC
SR1Q7_208	GT5	Glycogen/starch synthase, ADP-glucose type, glga
SR2Q5_1469	GT5	Glycogen/starch synthase, ADP-glucose type, glga
SR3Q1_1694	GT5	Glycogen/starch synthase, ADP-glucose type, glga
SR1Q5_1079	GT51	Penicillin-binding protein, 1A family
SR1Q5_1167	GT51	Penicillin-binding protein, 1A family
SR1Q7_1592	GT51	Penicillin-binding protein, 1A family
SR1Q7_2234	GT51	Penicillin-binding protein, 1A family
SR1Q7_890	GT51	Penicillin-binding protein, 1A family
SR2Q5_1186	GT51	Penicillin-binding protein, 1A family
SR2Q5_1279	GT51	Penicillin-binding protein, 1A family
SR3Q1_1596	GT51	Penicillin-binding protein, 1A family
SR3Q1_334	GT51	Penicillin-binding protein, 1A family
SR3Q1_364	GT8	CXXX repeat peptide maturase
SR1Q7_213	GT8	Deoxycytidine triphosphate deaminase, dcd
SR1Q7_2218	GT8	Excinuclease ABC subunit B, uvrb
SR1Q7_1952	GT8	Glycosyl transferase family 8
SR1Q7_2219	GT8	Glycosyl transferase family 8
SR1Q7_2220	GT8	Glycosyl transferase family 8
SR3Q1_354	GT8	Glycosyl transferase family 8
SR3Q1_357	GT8	Glycosyl transferase family 8
SR3Q1_365	GT8	Glycosyl transferase family 8
SR1Q7_2216	GT8	Hypothetical protein

Quinella genome bin genes	CAZyme families match	GAMOLA2 annotation match
SR1Q7_2217	GT8	Hypothetical protein
SR2Q5_1184	GT8	Hypothetical protein
SR3Q1_353	GT8	Hypothetical protein
SR3Q1_355	GT8	Hypothetical protein
SR3Q1_356	GT8	Hypothetical protein
SR3Q1_87	GT8	Hypothetical protein
SR1Q5_73	GT8	Poly-β-1,6 *N*-acetyl-D-glucosamine synthase, pgac
SR1Q5_900	GT81	Glycosyltransferase, TIGR04182 family
SR3Q1_129	GT81	Glycosyltransferase, TIGR04182 family
SR1Q7_1272	GT83	Dolichyl-phosphate-mannose-protein mannosyltransferase
SR2Q5_30	GT83	Glycosyl transferase
SR3Q1_2287	GT83	Glycosyl transferase
SR3Q1_2105	GT83	Oligosaccharyl transferase, archaeosortase A system- associated
SR1Q7_1268	GT9	Lipopolysaccharide heptosyltransferase I, waac
SR1Q7_1563	GT9	Lipopolysaccharide heptosyltransferase I, waac
SR2Q5_34	GT9	Lipopolysaccharide heptosyltransferase I, waac
SR3Q1_2109	GT9	Lipopolysaccharide heptosyltransferase I, waac
SR3Q1_2283	GT9	Lipopolysaccharide heptosyltransferase I, waac
SR3Q1_695	GT9	Lipopolysaccharide heptosyltransferase I, waac
SR1Q5_1659	GT9	Lipopolysaccharide heptosyltransferase II, waaf
SR1Q7_598	GT9	Lipopolysaccharide heptosyltransferase II, waaf
SR2Q5_819	GT9	Lipopolysaccharide heptosyltransferase II, waaf
SR3Q1_1170	GT9	Lipopolysaccharide heptosyltransferase II, waaf
SR1Q5_181	GT9	Putative lipopolysaccharide heptosyltransferase III, rfaq
SR1Q7_2075	GT9	Putative lipopolysaccharide heptosyltransferase III, rfaq
SR2Q5_1350	GT9	Putative lipopolysaccharide heptosyltransferase III, rfaq
SR3Q1_1870	GT9	Putative lipopolysaccharide heptosyltransferase III, rfaq
SR2Q5_321	GT92	Glycosyltransferase, TIGR04182 family
SR1Q5_1130	GT92	Hypothetical protein
SR1Q7_2203	GT92	Hypothetical protein
SR1Q7_2204	GT92	Hypothetical protein
SR1Q7_268	GT92	Hypothetical protein
SR2Q5_1067	GT92	Hypothetical protein
SR2Q5_1467	GT92	Hypothetical protein
SR3Q1_1218	GT92	Hypothetical protein
SR3Q1_1219	GT92	Hypothetical protein

Quinella genome bin genes	CAZyme families match	GAMOLA2 annotation match
SR3Q1_1220	GT92	Hypothetical protein
SR3Q1_1768	GT92	Hypothetical protein
SR3Q1_2335	GT92	Hypothetical protein

Supplementary Table 14. Key enzymes in sugar fermentation and associated energetics found in *Quinella* genome bins.

Enzyme	E.C. number	*Quinella* genome bins and gene numbers			
		SR1Q5	SR1Q7	SR2Q5	SR3Q1
β-Glucosidase		759[a]	451	479	185
α-Phosphotrehalase	3.2.1.1	127	1085	1500	1971
1,4-α-Glucan branching enzyme		1565	209, 1682	1470, 1651	445, 1695, 2497
Phosphoenolpyruvate-dependent phosphotransferase (ptsI)	2.7.3.9	1813	987	1461	2430
Phosphocarrier protein, Hpr	2.7.11.	332, 1814, 1815	988, 1602	883, 997, 1462	1951, 2429
Phosphoglucomutase	5.4.2.2	59	320	1353, 1357	792
Glucose-1-phosphatase	3.1.3.10	1118	1004	326	553
Glucokinase	2.7.1.1	1337	1991	1371	1543

Enzyme	E.C. number	*Quinella* genome bins and gene numbers			
		SR1Q5	SR1Q7	SR2Q5	SR3Q1
Glucose-6-phosphate isomerase	5.3.1.9	58	321	1352, 1356	793, 2391, 2467
6-Phosphofructokinase	2.7.1.11	1017, 1087	529, 1648	1089, 1662	66, 486
Fructose-1,6-bisphosphatase I	3.1.3.11	238	1559	-[b]	-
Fructose-bisphosphate aldolase	4.1.2.13	1533	2286	16	1198, 2174
Triosephosphate isomerase[c]	5.3.1.1	314	1842, 1909	284	272
Glyceraldehyde-3-phosphate dehydrogenase	1.2.1.12	313	1908	283	273
Phosphoglycerate kinase[c]	2.7.2.3	314	1842, 1909	284	272
Phosphoglycerate mutase	5.4.2.12	316	1845, 1911	287, 748	270
Phosphoglycerate enolase	4.2.1.11	897	1816	309	132
Phosphoenolpyruvate carboxykinase (ATP)	4.1.1.49	1272	705	1220	1538
Pyruvate kinase	2.7.1.40	1341	868	-	423
L-Lactate dehydrogenase	1.1.1.27	964	1633	1519	1033, 2465

Enzyme	E.C. number	*Quinella* genome bins and gene numbers			
		SR1Q5	SR1Q7	SR2Q5	SR3Q1
Ribose-5-phosphate isomerase	5.3.1.6	135, 379, 1511	530, 1024	461	200, 1960, 1962
Ribulose-5-phosphate 3-epimerase	5.1.3.1	437, 1718	96, 615	861	-
Transketolase	2.2.1.1	2058, 2062	460, 757, 758, 881, 882, 1667, 1668	469, 470, 471, 472, 481, 1571, 1572	189, 190, 1448, 1449
Transaldolase	2.2.1.2	-	-	-	2173
Pyruvate-ferredoxin/flavodoxin oxidoreductase	1.2.7.1	1976	152	868	2206
Flavodoxin		18, 1794	124, 1500	616, 1441	157, 1712
2-Oxoglutarate ferredoxin oxidoreductase, α subunit	1.2.7.8 or 1.2.7.3	1125	772	2	542
2-Oxoglutarate ferredoxin oxidoreductase, β subunit	1.2.7.8	1126	773	1	541
2-Oxoglutarate ferredoxin oxidoreductase, γ subunit	1.2.7.8	1127	774	-	540

Enzyme	E.C. number	*Quinella* genome bins and gene numbers			
		SR1Q5	SR1Q7	SR2Q5	SR3Q1
2-Oxoglutarate ferredoxin oxidoreductase, δ subunit/ 4Fe-4S ferredoxin		1124	771	3	543
NAD(P)H:flavin oxidoreductase/NADPH-flavin oxidoreductase		939, 1068	10, 1168, 1359	388, 774	307, 315, 769, 1313, 2281, 2513
Succinate CoA-transferase	2.8.3.-	482	972	1058	778
Formate dehydrogenase major subunit, fdoG	1.2.1.2	-	-	-	1644
Formate dehydrogenase iron-sulfur subunit, fdoH		-	-	-	1646
Formate dehydrogenase gamma subunit, fdoI		-	-	-	1647
Lactaldehyde reductase		-	413	-	-
Malate dehydrogenase		368	488	1303	2327
NiFe hydrogenase large subunit	1.12.2.1	381	532	1553	2344, 2244

Enzyme	E.C. number	*Quinella* genome bins and gene numbers			
		SR1Q5	SR1Q7	SR2Q5	SR3Q1
NiFe hydrogenase small subunit		380	531	1552	2245, 2502, 2343
NiFe hydrogenase cytochrome-*b* subunit		382	533	1554	2243, 2345
Methylmalonyl-CoA mutase		1136, 1137, 1910	573, 574, 2128	1082, 1083	526, 527, 2241, 2242
Methylmalonyl-CoA epimerase		485	969	1056	416, 775, 2283
Methylmalonyl-CoA decarboxylase, mmdA	4.1.1.41	486	969	1055	774
Methylmalonyl-CoA decarboxylase, mmdB		1999/767[d]	200	371, 1126	146
Methylmalonyl-CoA decarboxylase, mmdC		488	968	1055	773
Methylmalonyl-CoA decarboxylase, mmdD		2000	201	370, 1127	145
Oxaloacetate decarboxylase, oadA		441	1912	1198	582, 2395
Fumarate reductase subunit A		1181	2326	1218	1943
Fumarate reductase subunit B		1179	2328	1219	1944
Fumarate reductase subunit C		1182	2325	1217	1942

Enzyme	E.C. number	*Quinella* genome bins and gene numbers			
		SR1Q5	SR1Q7	SR2Q5	SR3Q1
ATP synthase F_o, a subunit		1785	891	1188	1281
ATP synthase F_o, b subunit		1787	893	1190	1279
ATP synthase F_o, c subunit		1786	892	1189	1280
ATP synthase F_1, α subunit	3.6.3.14	1789	896	1193	1277
ATP synthase F_1, β subunit		1791	898	1196	415
ATP synthase F_1, γsubunit		1790	897	1194	417
ATP synthase F_1, δ subunit		1788	894	1191	1278
ATP synthase F_1, ε subunit		1939	899	1197	414
Na^+/H^+ antiporter, nhac		520	867	1606	1074, 1545

[a]Gene number in Supplementary Data 1.

[b]–, Not detected.

[c]Fused genes present in all *Quinella* genome bins.

[d]Parts of the gene were present on the termini of different contigs and were assembled together to a generate full gene sequence.

Supplementary Table 15. GH enzyme family and related enzymes found in all *Quinella* genome bins.

GH family	Enzyme	Presence of signal peptides[a]	Possible function
GH1	β-Galactosidase	No	Galactose breakdown
GH3	β-Glucosidase	Yes	Cellobiose and cellodextrins breakdown
GH13	1,4-α-Glucan branching enzyme	No	Glycogen degradation
	α-Phosphotrehalase	Yes	Glycogen degradation
GH23	Lytic transglycosylase	No	Converts peptidoglycan to 1,6-anhydro sugars
	Transglycosylase	Yes	Convert peptidoglycan to 1,6-anhydro sugars
GH77	4-α-Glucanotransferase	No	Glycogen degradation
GH84	*O*-GlcNAcase	Yes	Unknown

[a]All are predicted to be non-cytoplasmic enzymes.

Supplementary Table 16. Phosphotransferase system (PTS) transporter components found in *Quinella* genome bins using TransportDB 2.0 database[28].

Quinella genome bin genes	Sub family	Substrate
SR1Q7_1675	EnzymeIIC	Ascorbate
SR1Q7_1676	EnzymeIIC	Ascorbate
SR1Q7_455	EnzymeIIC	Ascorbate
SR2Q5_487	EnzymeIIC	Ascorbate
SR1Q7_455	EnzymeIIC	Ascorbate
SR1Q5_1523	EnzymeIIA	Fructose
SR1Q7_1384	EnzymeIIAB	Fructose
SR1Q7_1385	EnzymeIIABC	Fructose
SR1Q7_1677	EnzymeIIA	Fructose
SR1Q7_454	EnzymeIIA	Fructose
SR1Q7_1754	EnzymeIIAB	Fructose
SR1Q7_1757	EnzymeIIAB	Fructose
SR2Q5_1321	EnzymeIIABC	Fructose
SR2Q5_1509	EnzymeIIABC	Fructose
SR2Q5_1757	EnzymeIIABC	Fructose
SR2Q5_181	EnzymeIIAB	Fructose
SR2Q5_488	EnzymeIIA	Fructose
SR3Q1_2251	EnzymeIIB	Fructose
SR3Q1_2252	EnzymeIIC	Fructose
SR3Q1_2253	EnzymeIIA	Fructose
SR3Q1_783	EnzymeIIABC	Fructose
SR3Q1_786	EnzymeIIAB	Fructose
SR3Q1_1576	EnzymeIIA	Fructose
SR1Q7_456	EnzymeIIB	Galactitol

Quinella genome bin genes	Sub family	Substrate
SR2Q5_485	EnzymeIIB	Galactitol
SR2Q5_486	EnzymeIIB	Galactitol
SR1Q5_1287	EnzymeIIA	Glucitol/sorbitol
SR1Q5_434	EnzymeIIC	Glucitol/sorbitol
SR1Q5_435	EnzymeIIB	Glucitol/sorbitol
SR1Q7_802	EnzymeIIA	Glucitol/sorbitol
SR1Q7_432	EnzymeIIA	Glucitol/sorbitol
SR1Q7_617	EnzymeIIA	Glucitol/sorbitol
SR2Q5_1289	EnzymeIIA	Glucitol/sorbitol
SR2Q5_605	EnzymeIIA	Glucitol/sorbitol
SR3Q1_1106	EnzymeIIA	Glucitol/sorbitol
SR3Q1_589	EnzymeIIA	Glucitol/sorbitol
SR3Q1_341	EnzymeIIA	Glucitol/sorbitol
SR1Q5_2046	EnzymeIIABC	Glucose
SR1Q7_793	EnzymeIIABC	Glucose
SR2Q5_586	EnzymeIIABC	Glucose
SR3Q1_1024	EnzymeIIABC	Glucose
SR3Q1_279	EnzymeIIABC	Glucose
SR3Q1_619	EnzymeIIABC	Glucose
SR1Q5_550	EnzymeIIABC	Glucose/maltose/*N*-acetylglucosamine
SR1Q5_873	EnzymeIIABC	Glucose/maltose/*N*-acetylglucosamine
SR1Q5_1782	EnzymeIIABC	Glucose/maltose/*N*-acetylglucosamine
SR1Q7_565	EnzymeIIABC	Glucose/maltose/*N*-acetylglucosamine
SR1Q7_683	EnzymeIIABC	Glucose/maltose/*N*-acetylglucosamine
SR1Q7_1999	EnzymeIIABC	Glucose/maltose/*N*-acetylglucosamine
SR2Q5_1011	EnzymeIIABC	Glucose/maltose/*N*-acetylglucosamine

Quinella genome bin genes	Sub family	Substrate
SR2Q5_1128	EnzymeIIABC	Glucose/maltose/*N*-acetylglucosamine
SR3Q1_143	EnzymeIIABC	Glucose/maltose/*N*-acetylglucosamine
SR3Q1_1581	EnzymeIIABC	Glucose/maltose/*N*-acetylglucosamine
SR3Q1_1499	EnzymeIIABC	Glucose/maltose/*N*-acetylglucosamine
SR3Q1_942	EnzymeIIABC	Glucose/maltose/*N*-acetylglucosamine
SR1Q5_325	EnzymeIIABC	Mannitol
SR1Q7_1379	EnzymeIIABC	Mannitol
SR2Q5_900	EnzymeIIABC	Mannitol
SR1Q5_508	EnzymeIID	Mannose/fructose
SR1Q5_509	EnzymeIIC	Mannose/fructose
SR1Q5_510	EnzymeIIB	Mannose/fructose
SR1Q5_511	EnzymeIIA	Mannose/fructose
SR1Q7_1652	EnzymeIIA	Mannose/fructose
SR1Q7_1653	EnzymeIIB	Mannose/fructose
SR1Q7_1654	EnzymeIIC	Mannose/fructose
SR1Q7_1655	EnzymeIID	Mannose/fructose
SR1Q7_1899	EnzymeIIA	Mannose/fructose
SR1Q7_1900	EnzymeIIB	Mannose/fructose
SR1Q7_1901	EnzymeIIC	Mannose/fructose
SR2Q5_41	EnzymeIID	Mannose/fructose
SR2Q5_42	EnzymeIIC	Mannose/fructose
SR2Q5_43	EnzymeIIB	Mannose/fructose
SR2Q5_44	EnzymeIIA	Mannose/fructose

Supplementary Table 17. Amino acid sequence similarities of fumarate reductase subunit C of *Quinella* genome bins, and other fumarate reductase subunit C proteins.

SQOR[a]	Genes in *Quinella* genome bins			
	SR1Q5_1182	SR1Q7_2325	SR2Q5_1217	SR3Q1_1942
B. subtilis	33.8[b]	34.8	34.8	33.8
P. macerans	38.9	37.9	36.0	39.3
C. jejuni	12.8	14.1	13.2	14.1
H. pylori	16.4	15.2	16.1	17.0
W. succinogenes	17.2	15.9	15.9	18.5
E. coli (type D SQOR)	14.9	14.9	14.0	14.0

[a]All are B type SQOR unless noted otherwise.

[b]All values are represented as percentages.

Supplementary Table 18. Steps and enzymes involved in ATP formation and consumption. The schemes are the possible different pathways (Supplementary Figure 15) for end product formation by *Quinella*.

Steps	Enzymes	ATP balances per substrate for different pathways				
		Glucose to 2 lactate	Glucose to 1.33 propionate + 0.66 acetate	Glucose + 2 H_2 to 2 propionate	Lactate to 0.66 propionate + 0.33 acetate	Lactate + H_2 to propionate
Glucose + Phosphoenolpyruvate → Glucose 6-phosphate	Phosphotransferase	0[a]	0[a]	0[a]	–[b]	–
Fructose-6-phosphate → Fructose-1, 6-bisphosphate	Phosphofructokinase	–1.0	–1.0	–1.0	–	–
1, 3-bisphosphoglycerate → 3-Phosphoglycerate	3-Phosphoglycerate kinase	2.0	2.0	2.0	–	–
Phosphoenolpyruvate → Pyruvate	Pyruvate kinase	1.0[a]	1.0[a]	1.0[a]	–	–
Pyruvate → Oxaloacetate	Oxaloacetate decarboxylase	–	–0.4[c]	–0.6[c]	–0.2[c]	–0.3[c]
Phosphoenolpyruvate → Oxaloacetate	Phosphoenolpyruvate carboxykinase (ATP-forming)		1.0	1.0		
Fumarate → Succinate	Fumarate reductase	–	0.8?[d]	2.4?[e]	0.4?[d]	1.2?[e]
Methylmalonyl-CoA → Propionyl-CoA	Methylmalonyl-CoA decarboxylase	–	0.4[c]	0.6[c]	0.2[c]	0.3[c]
Acetyl-CoA → Acetate	Succinate CoA-transferase	–	0.66	–	0.33	–
**Total ATP gain**		**2.0**	**2.66 to 3.06**	**2.0 to 5.0**	**0.33 to 0.73**	**0 to 1.2**

[a]One phosphoenolpyruvate is used in the phosphotransferase step, leaving only one to generate ATP at the pyruvate kinase step.

[b]–, not needed in this pathway.

[c]Na^+ pumping step, written as ATP equivalents, assuming 10 Na^+ are used to generate 3 ATP[82]. This would not be active if oxaloacetate is formed by phosphoenolpuyruvate carboxykinase. Other stoichiometries of Na^+ to ATP are possuble, which would change these ATP yields[82].

[d]Uncertain, because enzyme for transfer of NADH from fermentation to the fumarate reductase was not able to be identified. Assuming a quinone cycle is present, this could translocate 2 H^+ at the fumarate reductase step, which could be converted to ATP via the ATP synthase, assuming 10 H^+ are used to generate 3 ATP[82]. Other stoichiometries of H^+ to ATP are possuble, which would change these ATP yields[82].

[e]Potentially 4 H^+ translocated/generated at the fumarate reductase step, which could be converted to ATP via the ATP synthase, assuming 10 H^+ are used to generate 3 ATP[82]. Other stoichiometries of H^+ to ATP are possuble, which would change these ATP yields[82].

Supplementary Table 19. Primers used for amplification and sequencing of DNA and plasmid fragments.

Purpose	Primers	Primer sequence (5' - 3')	Target gene	Fragment size	PCR conditions
Amplification of bacterial 16S rRNA gene[a]	27F[c]<br>1492R[c]	GAGTTTGATCMTGGCTCAG<br>GGYTACCTTGTTACGACTT	16S rRNA	~1465 bp	94 °C for 4 min<br>94 °C for 1 min, 55 °C for 1 min, 72 °C for 1 min – 25 cycles<br>72 °C for 10 min
Amplification of insert from pCR2.1-TOPO plasmid vector[b]	GEM2987F<br>TOP168R	CCCAGTCACGACGTTGTAAAACG<br>ATGTTGTGTGGAATTGTGAGCGG	pCR2.1-TOPO vector and insert sequence	varied	94 °C for 4 min<br>94 °C for 15 s, 55 °C for 30 s, 72 °C for 1 min – 30 cycles<br>72 °C for 7 min
Amplification of 16S rRNA genes from DNA from concentrated *Quinella*-like cells[d]	9354F<br>19382F<br>111625F<br>24448F	CTCGACGTTCTTAATCTTCG<br>ACGACGATAATCCTGTGG<br>GATACGTCAGGTCATAGC<br>TGAATCAGCGAATAGAGC	16S rRNA gene plus flanking region from genome bins	varied	94 °C for 4 min<br>94 °C for 1 min, 55 °C for 1 min, 72 °C for 1 min – 25 cycles<br>72 °C for 10 min
Sequencing bacterial 16S rRNA genes[e]	514R[c]<br>518F[c]<br>800R<br>968F[f]<br>1100R[g]	CCGCGGCKGCTGGCAC<br>CCAGCAGCCGCGGTAATACG<br>TACCAGGGTATCTAATCC<br>AACGCGAAGAACCTTAC<br>GGGTTGCGCTCGTTG	16S rRNA	not applicable	–
Sequencing inserts in from pCR2.1-TOPO plasmid vector[e]	M13F<br>M13R	GTAAAACGACGGCCAGT<br>GCGGATAACAATTTCACACAGG	pCR2.1-TOPO vector and insert sequence	not applicable	–

[a, b]Used as pairs for amplification of defined products.
[c]Modified from Lane[133].
[d]Each used with primer 1492R.
[e]Used for sequencing only.
[f]Zoetendal et al.[134].
[g]Lane[133].

Supplementary Table 20. Hybridisation and washing buffer preparation for FISH.

	Formamide concentration (v/v, %)				
	0	20	40	60	80
Hybridisation buffer	Volume (µL)				
5 M NaCl	360	360	360	360	360
1 M Tris-HCl, pH 8.0	40	40	40	40	40
100% formamide	0	400	800	1200	1598
Sterile Milli-Q water	1598	1198	798	398	0
10% (w/v) SDS	2	2	2	2	2
Total volume	2000	2000	2000	2000	2000
Wash buffer	Volume (µL)				
5 M NaCl	9000	2150	460	40	0
1 M Tris-HCl, pH 8.0	1000	1000	1000	1000	1000
0.5 M EDTA	0	500	500	500	175
Sterile Milli-Q water	39950	46300	47990	48410	48775
10% (w/v) SDS	50	50	50	50	50
Total volume	50000	50000	50000	50000	50000

Supplementary References

1. Krumholz LR, Bryant MP, Brulla WJ, Vicini JL, Clark JH, Stahl DA. Proposal of *Quinella ovalis* gen. nov., sp. nov., based on phylogenetic analysis. *International Journal of Systematic Bacteriology* **43**, 393-296 (1993).

2. Woldringh CL, Nanninga N. Structure of the nucleoid and cytoplasm in the intact cell. In: *Molecular Cytology of Escherichia coli* (ed Nanninga N). Academic Press (1985).

3. Satterly J. Formulae for volumes, surface areas and radii of gyration of spheres, ellipsoids and spheroids. *Mathematical Gazette* **44**, 15-19 (1960).

4. Kittelmann S, et al. Two different bacterial community types are linked with the low-methane emission trait in sheep. *PLoS One* **9**, e103171 (2014).

5. Caporaso JG, et al. QIIME allows analysis of high-throughput community sequencing data. *Nature methods* **7**, 335-336 (2010).

6. Henderson G, et al. Improved taxonomic assignment of rumen bacterial 16S rRNA sequences using a revised SILVA taxonomic framework. *PeerJ* **7**, e6496 (2019).

7. Ludwig W, et al. ARB: A software environment for sequence data. *Nucleic Acids Research* **32**, 1363-1371 (2004).

8. Kunin V, Engelbrektson A, Ochman H, Hugenholtz P. Wrinkles in the rare biosphere: Pyrosequencing errors can lead to artificial inflation of diversity estimates. *Environmental Microbiology* **12**, 118-123 (2010).

9. Huse SM, Welch DM, Morrison HG, Sogin ML. Ironing out the wrinkles in the rare biosphere through improved OTU clustering. *Environmental Microbiology* **12**, 1889-1898 (2010).

10. Greuter D, Loy A, Horn M, Rattei T. probeBase—an online resource for rRNA-targeted oligonucleotide probes and primers: new features 2016. *Nucleic Acids Research* **44**, D586-D589 (2016).

11. Parks DH, Imelfort M, Skennerton CT, Hugenholtz P, Tyson GW. CheckM: assessing the quality of microbial genomes recovered from isolates, single cells, and metagenomes. *Genome research* **25**, 1043-1055 (2015).

12. Yutin N, Galperin MY. A genomic update on clostridial phylogeny: Gram-negative spore formers and other misplaced clostridia. *Environmental Microbiology* **15**, 2631-2641 (2013).

13. Yarza P, et al. The All-Species Living Tree project: A 16S rRNA-based phylogenetic tree of all sequenced type strains. *Systematic and Applied Microbiology* **31**, 241-250 (2008).

14. Kaneko J, et al. Complete genome sequence of *Selenomonas ruminantium subsp. lactilytica* will accelerate further understanding of the nature of the class Negativicutes. *FEMS Microbiology Letters* **362**, fnv050 (2015).

15. Altermann E, Lu J, McCulloch A. GAMOLA2, a comprehensive software package for the annotation and curation of draft and complete microbial genomes. *Frontiers in Microbiology* **8**, 346 (2017).

16. Lombard V, Golaconda Ramulu H, Drula E, Coutinho PM, Henrissat B. The carbohydrate-active enzymes database (CAZy) in 2013. *Nucleic Acids Research* **42**, D490-D495 (2014).

17. Käll L, Krogh A, Sonnhammer ELL. A combined transmembrane topology and signal peptide prediction method. *Journal of Molecular Biology* **338**, 1027-1036 (2004).

18. Viklund H, Bernsel A, Skwark M, Elofsson A. SPOCTOPUS: a combined predictor of signal peptides and membrane protein topology. *Bioinformatics* **24**, 2928-2929 (2008).

19. Tsirigos KD, Peters C, Shu N, Käll L, Elofsson A. The TOPCONS web server for consensus prediction of membrane protein topology and signal peptides. *Nucleic Acids Research* **43**, W401-407 (2015).

20. Forsberg CW, Cheng KJ, White BA. Polysaccharide degradation in the rumen and large intestine. In: *Gastrointestinal Microbiology: Volume 1 Gastrointestinal Ecosystems and Fermentations* (eds Mackie RI, White BA). Springer (1997).

21. Krause DO, et al. Opportunities to improve fiber degradation in the rumen: microbiology, ecology, and genomics. *FEMS microbiology reviews* **27**, 663-693 (2003).

22. Henrissat B, Bairoch A. New families in the classification of glycosyl hydrolases based on amino acid sequence similarities. *Biochemical Journal* **293**, 781-788 (1993).

23. Cerrilla MEO, Martínez GM. Starch digestion and glucose metabolism in the ruminant: A review. *Interciencia* **28**, 380-386 (2003).

24. Brough EB, Reid TC, Howard BH. The biochemistry of the rumen bacterium Quin's oval - part 1. *New Zealand Journal of Science* **13**, 570-575 (1970).

25. Cloud-Hansen KA, Peterson SB, Stabb EV, Goldman WE, McFall-Ngai MJ, Handelsman J. Breaching the great wall: Peptidoglycan and microbial interactions. *Nature Reviews Microbiology* **4**, 710-716 (2006).

26. Scheurwater E, Reid CW, Clarke AJ. Lytic transglycosylases: Bacterial space-making autolysins. *International Journal of Biochemistry and Cell Biology* **40**, 586-591 (2008).

27. Alonso J, Schimpl M, Van Aalten DMF. O-GlcNAcase: Promiscuous hexosaminidase or key regulator of O-GlcNAc signaling? *Journal of Biological Chemistry* **289**, 34433-34439 (2014).

28. Elbourne LDH, Tetu SG, Hassan KA, Paulsen IT. TransportDB 2.0: a database for exploring membrane transporters in sequenced genomes from all domains of life. *Nucleic Acids Research* **45**, D320-D324 (2017).

29. Tchieu JH, Norris V, Edwards JS, Saier M.H, Jr. The complete phosphotransferase system in *Escherichia coli*. *Journal of Molecular Microbiology and Biotechnology* **3**, 329-346 (2001).

30. Schurig H, Beaucamp N, Ostendorp R, Jaenicke R, Adler E, Knowles JR. Phosphoglycerate kinase and triosephosphate isomerase from the hyperthermophilic bacterium *Thermotoga maritima* form a covalent bifunctional enzyme complex. *EMBO Journal* **14**, 442-451 (1995).

31. Waygood EB, Sanwal BD. The control of pyruvate kinases of *Escherichia coli*. I. Physicochemical and regulatory properties of the enzyme activated by fructose 1,6 diphosphate. *Journal of Biological Chemistry* **249**, 265-274 (1974).

32. Cronín CN, Nolan DP, Paul Voorheis H. The enzymes of the classical pentose phosphate pathway display differential activities in procyclic and bloodstream forms of *Trypanosoma brucei*. *FEBS Letters* **244**, 26-30 (1989).

33. Chen X, et al. The Entner–Doudoroff pathway is an overlooked glycolytic route in cyanobacteria and plants. *Proceedings of the National Academy of Sciences USA* **113**, 5441-5446 (2016).

34. Murray EL, Conway T. Multiple regulators control expression of the Entner-Doudoroff aldolase (Eda) of *Escherichia coli*. *Journal of Bacteriology* **187**, 991-1000 (2005).

35. Kamke J, et al. Rumen metagenome and metatranscriptome analyses of low methane yield sheep reveals a *Sharpea*-enriched microbiome characterised by lactic acid formation and utilisation. *Microbiome* **4**, 56 (2016).

36. Kumar S, et al. *Sharpea* and *Kandleria* are lactic acid producing rumen bacteria that do not change their fermentation products when co-cultured with a methanogen. *Anaerobe* **54**, 31-38 (2018).

37. Kanegasaki S, Takahashi H. Function of growth factors for rumen microorganisms. I. Nutritional characteristics of *Selenomonas ruminantium*. *Journal of Bacteriology* **93**, 456-463 (1967).

38. Orpin CG. The culture in vitro of the rumen bacterium Quin's oval. *Journal of General Microbiology* **73**, 523-530 (1972).

39. Pieulle L, Guigliarelli B, Asso M, Dole F, Bernadac A, Hatchikian EC. Isolation and characterization of the pyruvate-ferredoxin oxidoreductase from the sulfate-reducing bacterium *Desulfovibrio africanus*. *Biochimica et Biophysica Acta* **1250**, 49-59 (1995).

40. Gottschalk G. *Bacterial Metabolism*, 2 edn. Springer-Verlag (1986).

41. Ferry J. Acetate metabolism in anaerobes from the domain archaea. *Life* **5**, 1454-1571 (2015).

42. Mack M, Buckel W. Conversion of glutaconate CoA-transferase from *Acidaminococcus fermentans* into an acyl-CoA hydrolase by site-directed mutagenesis. *FEBS Letters* **405**, 209-212 (1997).

43. Solomon F, Jencks WP. Identification of an enzyme-gamma-glutamyl coenzyme A intermediate from coenzyme A transferase. *Journal of Biological Chemistry* **244**, 1079-1081 (1969).

44. Tielens AGM, van Grinsven KWA, Henze K, van Hellemond JJ, Martin W. Acetate formation in the energy metabolism of parasitic helminths and protists. *International Journal for Parasitology* **40**, 387-397 (2010).

45. Heider J. A new family of CoA-transferases. *FEBS Letters* **509**, 345-349 (2001).

46. Fraser ME, James MNG, Bridger WA, Wolodko WT. A detailed structural description of *Escherichia coli* succinyl-CoA synthetase. *Journal of Molecular Biology* **285**, 1633-1653 (1999).

47. Rivière L, et al. Acetyl:succinate CoA-transferase in procyclic Trypanosoma brucei. Gene identification and role in carbohydrate metabolism. *Journal of Biological Chemistry* **279**, 45337-45346 (2004).

48. Van Grinsven KWA, et al. Acetate:succinate CoA-transferase in the hydrogenosomes of *Trichomonas vaginalis*: Identification and characterization. *Journal of Biological Chemistry* **283**, 1411-1418 (2008).

49. Marvin-Sikkema FD, Pedro Gomes TM, Grivet JP, Gottschal JC, Prins RA. Characterization of hydrogenosomes and their role in glucose metabolism of *Neocallimastix* sp. L2. *Archives of Microbiology* **160**, 388-396 (1993).

50. Michel TA, Macy JM. Purification of an enzyme responsible for acetate formation from acetyl coenzyme A in *Selenomonas ruminatium*. *FEMS Microbiology Letters* **68**, 189-194 (1990).

51. Knappe J, Blaschkowski HP, Grobner P, Schmttt T. Pyruvate formate-lyase of *Escherichia coli*: the acetyl-enzyme intermediate. *European Journal of Biochemistry* **50**, 253-263 (1974).

52. Crable BR, Plugge CM, McInerney MJ, Stams AJM. Formate formation and formate conversion in biological fuels production. *Enzyme Research* **2011**, 532-536 (2011).

53. Brown AT, Patterson CE. Ethanol production and alcohol dehydrogenase activity in *Streptococcus mutans*. *Archives of Oral Biology* **18**, 127-131 (1973).

54. Asanuma N, Ishiwata M, Yoshii T, Kikuchi M, Nishina Y, Hino T. Characterization and transcription of the genes involved in butyrate production in *Butyrivibrio fibrisolvens* type I and II strains. *Current Microbiology* **51**, 91-94 (2005).

55. Paillard D, et al. Relation between phylogenetic position, lipid metabolism and butyrate production by different *Butyrivibrio*-like bacteria from the rumen. *Antonie van Leeuwenhoek* **91**, 417-422 (2007).

56. Samols D, Thornton CG, Murtif VL, Kumar GK, Haase FC, Wood HG. Evolutionary conservation among biotin enzymes. *Journal of Biological Chemistry* **263**, 6461-6464 (1988).

57. Dimroth P, Kaim G, Matthey U. The motor of the ATP synthase. *Biochimica et Biophysica Acta* **1365**, 87-92 (1998).

58. Hilpert W, Dimroth P. Purification and characterization of a new sodium-transport decarboxylase. *European Journal of Biochemistry* **132**, 579-587 (1983).

59. Bott M, Pfister K, Burda P, Kalbermatter O, Woehlke G, Dimroth P. Methylmalonyl-CoA decarboxylase from *Propionigenium modestum*. *European Journal of Biochemistry* **250**, 590-599 (1997).

60. Huder JB, Dimroth P. Sequence of the sodium ion pump methylmalonyl-CoA decarboxylase from *Veillonella parvula*. *Journal of Biological Chemistry* **268**, 24564-24571 (1993).

61. Hilpert W, Dimroth P. On the mechanism of sodium ion translocation by methylmalonyl-CoA decarboxylase from *Veillonella alcalescens*. *European Journal of Biochemistry* **195**, 79-86 (1991).

62. Huder JB, Dimroth P. Expression of the sodium ion pump methylmalonyl-coenzyme A-decarboxylase from *Veillonella parvula* and of mutated enzyme specimens in *Escherichia coli*. *Journal of Bacteriology* **177**, 3623-3630 (1995).

63. Denger K, Schink B. Energy conservation by succinate decarboxylation in *Veillonella parvula*. *Microbiology* **138**, 967-971 (1992).

64. Laussermair E, Schwarz E, Oesterhelt D, Reinke H, Beyreuther K, Dimroth P. The sodium ion translocating oxaloacetate decarboxylase of *Klebsiella pneumoniae*. Sequence of the integral membrane-bound subunits β and γ. *Journal of Biological Chemistry* **264**, 14710-14715 (1989).

65. Schwarz E, Oesterhelt D, Reinke H, Beyreuther K, Dimroth P. The sodium ion translocating oxalacetate decarboxylase of *Klebsiella pneumoniae*. Sequence of the biotin-containing α-subunit and relationship to other biotin-containing enzymes. *Journal of Biological Chemistry* **263**, 9640-9645 (1988).

66. Woehlke G, Wifling K, Dimroth P. Sequence of the sodium ion pump oxaloacetate decarboxylase from *Salmonella typhimurium*. *Journal of Biological Chemistry* **267**, 22798-22803 (1992).

67. Jockel P, Di Berardino M, Dimroth P. Membrane topology of the β-Subunit of the oxaloacetate decarboxylase Na^+ pump from *Klebsiella pneumoniae*. *Biochemistry* **38**, 13461-13472 (1999).

68. Dahinden P, Pos KM, Dimroth P. Identification of a domain in the α-subunit of the oxaloacetate decarboxylase Na^+ pump that accomplishes complex formation with the γ-subunit. *FEBS Journal* **272**, 846-855 (2005).

69. Studer R, Dahinden P, Wang W-W, Auchli Y, Li X-D, Dimroth P. Crystal structure of the carboxyltransferase domain of the oxaloacetate decarboxylase Na^+ pump from *Vibrio cholerae*. *Journal of Molecular Biology* **367**, 547-557 (2007).

70. Benning MM, Haller T, Gerlt JA, Holden HM. New reactions in the crotonase superfamily: structure of methylmalonyl CoA decarboxylase from *Escherichia coli*. *Biochemistry* **39**, 4630-4639 (2000).

71. Gronow S, et al. Complete genome sequence of *Veillonella parvula* type strain ($Te3^T$). *Standards in Genomic Science* **2**, 57-65 (2010).

72. Hoffmann A, Hilpert W, Dimroth P. The carboxyltransferase activity of the sodium-ion-translocating methylmalonyl-CoA decarboxylase of *Veillonella alcalescens*. *European Journal of Biochemistry* **179**, 645-650 (1989).

73. Dimroth P, Thomer A. Subunit composition of oxaloacetate decarboxylase and characterization of the α chain as carboxyltransferase. *European Journal of Biochemistry* **137**, 107-112 (1983).

74. Di Berardino M, Dimroth P. Aspartate 203 of the oxaloacetate decarboxylase β-subunit catalyses both the chemical and vectorial reaction of the Na^+ pump. *EMBO Journal* **15**, 1842-1849 (1996).

75. Jockel P, Schmid M, Choinowski T, Dimroth P. Essential role of tyrosine 229 of the oxaloacetate decarboxylase β-subunit in the energy coupling mechanism of the Na^+ pump. *Biochemistry* **39**, 4320-4326 (2000).

76. Jockel P, Schmid M, Steuber J, Dimroth P. A molecular coupling mechanism for the oxaloacetate decarboxylase Na^+ pump as inferred from mutational analysis. *Biochemistry* **39**, 2307-2315 (2000).

77. Kumar GK, Bahler CR, Wood HG, Merrifield RB. The amino acid sequences of the biotinyl subunit essential for the association of transcarboxylase. *Journal of Biological Chemistry* **257**, 13828-13834 (1982).

78. Buckel W. Sodium ion-translocating decarboxylases. *Biochimica et Biophysica Acta* **1505**, 15-27 (2001).

79. Towbin H, Staehelin T, Gordon J. Electrophoretic transfer of proteins from polyacrylamide gels to nitrocellulose sheets: Procedure and some applications. *Proceedings of the National Academy of Sciences USA* **76**, 4350-4354 (1979).

80. Boutet E, et al. UniProtKB/Swiss-Prot, the manually annotated section of the UniProt knowledgebase: How to use the entry view. *Methods in Molecular Biology* **1374**, 23-54 (2016).

81. Dimroth P, Jockel P, Schmid M. Coupling mechanism of the oxaloacetate decarboxylase Na^+ pump. *Biochimica et Biophysica Acta* **1505**, 1-14 (2001).

82. Meier T, Faraldo-Gómez J, Börsch M. ATP synthase – A paradigmatic molecular machine. In: *Molecular Machines in Biology: Workshop of the Cell* (ed Frank J). Cambridge University Press (2011).

83. Hägerhäll C, Hederstedt L. A structural moDAl for the membrane-integral domain of succinate:quinone oxidoreductases. *FEBS Letters* **389**, 25-31 (1996).

84. Saraste M. Oxidative phosphorylation at the *fin de siècle*. *Science* **283**, 1488-1493 (1999).

85. Lancaster CRD, Kröger A, Auer M, Michel H. Structure of fumarate reductase from *Wolinella succinogenes* at 2.2Å resolution. *Nature* **402**, 377-385 (1999).

86. Iverson TM, Luna-Chavez C, Cecchini G, Rees DC. Structure of the *Escherichia coli* fumarate reductase respiratory complex. *Science* **284**, 1961-1966 (1999).

87. Kröger A, Geisler V, Lemma E, Theis F, Lenger R. Bacterial fumarate respiration. *Archives of Microbiology* **158**, 311-314 (1992).

88. Kröger A. Fumarate as terminal acceptor of phosphorylative electron transport. *Biochimica et Biophysica Acta* **505**, 129-145 (1978).

89. Maklashina E, Berthold DA, Cecchini G. Anaerobic expression of *Escherichia coli* succinate dehydrogenase: functional replacement of fumarate reductase in the respiratory chain during anaerobic growth. *Journal of Bacteriology* **180**, 5989-5996 (1998).

90. Lancaster CRD. *Wolinella succinogenes* quinol:fumarate reductase and its comparison to *E. coli* succinate:quinone reductase. *FEBS Letters* **555**, 21-28 (2003).

91. Hederstedt L. Respiration without O_2. *Science* **284**, 1941-1942 (1999).

92. Lancaster CRD, Kröger A. Succinate: Quinone oxidoreductases: new insights from X-ray crystal structures. *Biochimica et Biophysica* **1459**, 422-431 (2000).

93. Engelbrecht S, Junge W. ATP synthase: A tentative structural model. *FEBS Letters* **414**, 485-491 (1997).

94. Grüber G, Manimekalai MSS, Mayer F, Müller V. ATP synthases from archaea: The beauty of a molecular motor. *Biochimica et Biophysica Acta* **1837**, 940-952 (2014).

95. Santana M, et al. Bacillus subtilis F_0F_1 ATPase: DNA sequence of the *atp* operon and characterization of *atp* mutants. *Journal of Bacteriology* **176**, 6802-6811 (1994).

96. Ito M, Guffanti AA, Zemsky J, Ivey DM, Krulwich TA. Role of the nhaC-encoded Na^+/H^+ antiporter of alkaliphilic *Bacillus firmus* OF4. *Journal of Bacteriology* **179**, 3851-3857 (1997).

97. Greening C, et al. Genomic and metagenomic surveys of hydrogenase distribution indicate H_2 is a widely utilised energy source for microbial growth and survival. *ISME Journal* **10**, 761-777 (2016).

98. Vignais PM, Billoud B. Occurrence, classification, and biological function of hydrogenases: An overview. *Chemical Reviews* **107**, 4206-4272 (2007).

99. Shima S, et al. The crystal structure of [Fe]-hydrogenase reveals the geometry of the active site. *Science* **321**, 572-575 (2008).

100. Flanagan LA, Parkin A. Electrochemical insights into the mechanism of NiFe membrane-bound hydrogenases. *Biochemical Society Transactions* **44**, 315-328 (2016).

101. Volbeda A, Charon M-H, Piras C, Hatchikian EC, Frey M, Fontecilla-Camps JC. Crystal structure of the nickel-iron hydrogenase from *Desulfovibrio gigas*. *Nature* **373**, 580-587 (1995).

102. Goris T, et al. A unique iron-sulfur cluster is crucial for oxygen tolerance of a [NiFe]-hydrogenase. *Nature Chemical Biology* **7**, 310-318 (2011).

103. Fritsch J, et al. The crystal structure of an oxygen-tolerant hydrogenase uncovers a novel iron-sulphur centre. *Nature* **479**, 249-252 (2011).

104. Shomura Y, Yoon K-S, Nishihara H, Higuchi Y. Structural basis for a [lsqb]4Fe-3S[rsqb] cluster in the oxygen-tolerant membrane-bound [lsqb]NiFe[rsqb]-hydrogenase. *Nature* **479**, 253-256 (2011).

105. Lukey MJ, et al. Oxygen-tolerant [NiFe]-hydrogenases: The individual and collective importance of supernumerary cysteines at the proximal Fe-S cluster. *Journal of the American Chemical Society* **133**, 16881-16892 (2011).

106. Volbeda A, et al. X-ray crystallographic and computational studies of the O2-tolerant [NiFe]-hydrogenase 1 from *Escherichia coli*. *Proceedings of the National Academy of Sciences USA* **109**, 5305-5310 (2012).

107. Volbeda A, Darnault C, Parkin A, Sargent F, Armstrong Fraser A, Fontecilla-Camps Juan C. Crystal structure of the O_2-tolerant membrane-bound hydrogenase 1 from *Escherichia coli* in complex with its cognate cytochrome *b*. *Structure* **21**, 184-190 (2013).

108. Menon NK, Robbins J, Wendt JC, Shanmugam KT, Przybyla AE. Mutational analysis and characterization of the E*scherichia coli* hya operon, which encodes [NiFe] hydrogenase 1. *Journal of Bacteriology* **173**, 4851-4861 (1991).

109. Berks BC, et al. Sequence analysis of subunits of the membrane-bound nitrate reductase from a denitrifying bacterium: the integral membrane subunit provides a prototype for the dihaem electron-carrying arm of a redox loop. *Molecular Microbiology* **15**, 319-331 (1995).

110. Meek L, Arp DJ. The hydrogenase cytochrome b heme ligands of *Azotobacter vinelandii* are required for full H_2 oxidation capability. *Journal of Bacteriology* **182**, 3429-3436 (2000).

111. Gross R, Simon J, Lancaster CRD, Kröger A. Identification of histidine residues in *Wolinella succinogenes* hydrogenase that are essential for menaquinone reduction by H_2. *Molecular Microbiology* **30**, 639-646 (1998).

112. Buckel W, Thauer RK. Energy conservation via electron bifurcating ferredoxin reduction and proton/Na^+ translocating ferredoxin oxidation. *Biochimica et Biophysica Acta* **1827**, 94-113 (2013).

113. Biegel E, Schmidt S, Müller V. Genetic, immunological and biochemical evidence for a Rnf complex in the acetogen *Acetobacterium woodii*. *Environmental Microbiology* **11**, 1438-1443 (2009).

114. Kerepesi C, Bánky D, Grolmusz V. AmphoraNet: The webserver implementation of the AMPHORA2 metagenomic workflow suite. *Gene* **533**, 538-540 (2014).

115. Bankevich A, et al. SPAdes: A new genome assembly algorithm and its applications to single-cell sequencing. *Journal of Computational Biology* **19**, 455-477 (2012).

116. Stackebrandt E, Goebel BM. Taxonomic note: A place for DNA-DNA reassociation and 16S rRNA sequence analysis in the present species definition in bacteriology. *International Journal of Systematic Bacteriology* **44**, 846-849 (1994).

117. Kim M, Oh HS, Park SC, Chun J. Towards a taxonomic coherence between average nucleotide identity and 16S rRNA gene sequence similarity for species demarcation of prokaryotes. *International Journal of Systematic and Evolutionary Microbiology* **64**, 346-351 (2014).

118. Altermann E. Tracing lifestyle adaptation in prokaryotic genomes. *Frontiers in Microbiology* **3**, 48 (2012).

119. Kumar S, Stecher G, Tamura K. MEGA7: Molecular evolutionary genetics analysis version 7.0 for bigger datasets. *Molecular Biology and Evolution* **33**, 1870-1874 (2016).

120. Sneath PHA, Sokal RR. Numerical taxonomy. *Nature* **193**, 855-860 (1962).

121. Kanehisa M, Furumichi M, Tanabe M, Sato Y, Morishima K. KEGG: new perspectives on genomes, pathways, diseases and drugs. *Nucleic Acids Research* **45**, D353-D361 (2017).

122. Webb EC. *Enzyme Nomenclature 1992: Recommendations of the Nomenclature Committee of the International Union of Biochemistry and Molecular Biology on the nomenclature and Classification of Enzymes.* Academic Press (1992).

123. Jukes HT, Cantor RC. Evolution of protein molecules. In: *Mammalian Protein Metabolism* (ed Munro HN). Academic Press (1969).

124. Saitou N, Nei M. The neighbor-joining method: a new method for reconstructing phylogenetic trees. *Molecular Biology and Evolution* **4**, 406-425 (1987).

125. Klimchuk OI, Dibrova DV, Mulkidjanian AY. Phylogenomic analysis identifies a sodium-translocating decarboxylating oxidoreductase in thermotogae. *Biochemistry (Moscow)* **81**, 481-490 (2016).

126. Edgar RC. MUSCLE: multiple sequence alignment with high accuracy and high throughput. *Nucleic Acids Research* **32**, 1792-1797 (2004).

127. Larkin MA, et al. Clustal W and Clustal X version 2.0. *Bioinformatics* **23**, 2947-2948 (2007).

128. Henderson G, et al. Rumen microbial community composition varies with diet and host, but a core microbiome is found across a wide geographical range. *Scientific Reports* **5**, 14567 (2015).

129. Gurevich A, Saveliev V, Vyahhi N, Tesler G. QUAST: Quality assessment tool for genome assemblies. *Bioinformatics* **29**, 1072-1075 (2013).

130. Kang DD, Froula J, Egan R, Wang Z. MetaBAT, an efficient tool for accurately reconstructing single genomes from complex microbial communities. *PeerJ* **3**, e1165 (2015).

131. Tatusov RL, et al. The COG database: New developments in phylogenetic classification of proteins from complete genomes. *Nucleic Acids Research* **29**, 22-28 (2001).

132. Yin Y, Mao X, Yang J, Chen X, Mao F, Xu Y. DbCAN: A web resource for automated carbohydrate-active enzyme annotation. *Nucleic Acids Research* **40**, W445-W451 (2012).

133. Lane DJ. 16S/23S rRNA sequencing. In: *Nucleic Acid Techniques in Bacterial Systematic* (eds Stackebrandt E, Goodfellow M). John Wiley and Sons (1991).

134. Zoetendal EG, von Wright A, Vilpponen-Salmela T, Ben-Amor K, Akkermans AD, de Vos WM. Mucosa-associated bacteria in the human gastrointestinal tract are uniformly distributed along the colon and differ from the community recovered from feces. *Applied and Environmental Microbiology* **68**, 3401-3407 (2002).
