# Supplementary material for: Genomic insights into the physiology of Quinella, an iconic uncultured rumen bacterium
Source: Nat Commun. 2022 Oct 20;13:6240. doi: 10.1038/s41467-022-34013-1 (PMC9585023; doi:10.1038/s41467-022-34013-1)
Supplement: Supplementary file 2 — Description of Additional Supplementary Files [file 41467_2022_34013_MOESM2_ESM.pdf]

Description of Additional Supplementary Files:

Supplementary Data 1. Summary of the genome bin annotations based on the outputs from multiple automated annotation tools and manual curation after further investigation of key proteins encoded in the genome bins. The file contains four sheets, one for each genome bin.

Supplementary Data 2. Hyperlinks to GenBank accessions for additional DNA sequence data generated in this study.
