# Supplementary material for: Genomic insights into the physiology of Quinella, an iconic uncultured rumen bacterium
Source: Nat Commun. 2022 Oct 20;13:6240. doi: 10.1038/s41467-022-34013-1 (PMC9585023; doi:10.1038/s41467-022-34013-1)
Supplement: Supplementary file 5 — Reporting Summary [file 41467_2022_34013_MOESM5_ESM.pdf]

nature portfolio

Corresponding author(s): Sandeep Kumar
Peter H. Janssen

Last updated by author(s): Oct 7, 2022

Reporting Summary

Nature Portfolio wishes to improve the reproducibility of the work that we publish. This form provides structure for consistency and transparency in reporting. For further information on Nature Portfolio policies, see our Editorial Policies and the Editorial Policy Checklist.

Statistics

For all statistical analyses, confirm that the following items are present in the figure legend, table legend, main text, or Methods section.

n/a	Confirmed	
☐	☒	The exact sample size (*n*) for each experimental group/condition, given as a discrete number and unit of measurement
☒	☐	A statement on whether measurements were taken from distinct samples or whether the same sample was measured repeatedly
☒	☐	The statistical test(s) used AND whether they are one- or two-sided<br>*Only common tests should be described solely by name; describe more complex techniques in the Methods section.*
☒	☐	A description of all covariates tested
☒	☐	A description of any assumptions or corrections, such as tests of normality and adjustment for multiple comparisons
☐	☒	A full description of the statistical parameters including central tendency (e.g. means) or other basic estimates (e.g. regression coefficient) AND variation (e.g. standard deviation) or associated estimates of uncertainty (e.g. confidence intervals)
☒	☐	For null hypothesis testing, the test statistic (e.g. *F*, *t*, *r*) with confidence intervals, effect sizes, degrees of freedom and *P* value noted<br>*Give P values as exact values whenever suitable.*
☒	☐	For Bayesian analysis, information on the choice of priors and Markov chain Monte Carlo settings
☒	☐	For hierarchical and complex designs, identification of the appropriate level for tests and full reporting of outcomes
☒	☐	Estimates of effect sizes (e.g. Cohen's *d*, Pearson's *r*), indicating how they were calculated

Our web collection on statistics for biologists contains articles on many of the points above.

Software and code

Policy information about availability of computer code

Data collection	*Image capture using phase contrast, fluorescence and electron microscopy*<br>*- Leica Application Suite, V2.2, Leica Microsystems Cambridge Ltd*<br>*- XT Microscope Control, FEI Company*<br>*- TUI version 4.5, FEI Company*<br>*Image capture for DNA gel electrophoresis*<br>*- Camera Control Pro 2, V2.7, Nikon*<br>*- Lightroom 2.15.0, Adobe*<br>*Measurements on images*<br>*- Paint version 21H2, Microsoft Corporation*
Data analysis	*16S rRNA gene amplicon formation*<br>*- Geneious 8.1, https://www.geneious.com/*<br><br>*16S rRNA gene chimera detection*<br>*- Bellerophon 2003, https://comp-bio.anu.edu.au/Bellerophon/doc/doc.html*<br>*- UCHIME Version 7, https://www.drive5.com/usearch/manual/uchime_algo.html*<br>*- ARB Versions 5.5 and 6.0, http://www.arb-home.de/*

16S rRNA gene analysis and tree generation
- QIIME version 1.0, http://qiime.org/
- SINA aligner version 1.2.11, https://www.arb-silva.de/aligner
- ARB versions 5.5 and 6.0, http://www.arb-home.de/
- RAxML version 8.2.0, https://github.com/stamatak/standard-RAxML
- Geneious 8.1, https://www.geneious.com/

16S rRNA probe design
- ARB probe match tool in ARB version 5.5, http://www.arb-home.de/

DNA sequencing quality control
- FastQC version 0.10.1, https://www.bioinformatics.babraham.ac.uk/projects/fastqc/
- Trimmomatic version 0.36, http://www.usadellab.org/cms/?page=trimmomatic

RNA sequence analysis
- Flash2 version 1.2.11, https://github.com/dstreett/FLASH2
- seqtk version 1.3, https://github.com/lh3/seqtk
- Diamond version 2.0.14, https://github.com/bbuchfink/diamond

Genome bin construction and analysis
- SPAdes Genome Assembler version 3.7.1, https://github.com/ablab/spades
- MetaBAT v0.26.3, https://bitbucket.org/berkeleylab/metabat/src/master/
- CheckM version 1.0.5, https://github.com/Ecogenomics/CheckM
- QUAST version 4.1, http://quast.sourceforge.net/quast.html
- AMPHORA2, https://github.com/wu-lab-uva/AMPHORA2

Genome bin phylogenomics
- GTDB-Tk version 2.1.0, https://github.com/Ecogenomics/GTDBTk
- FGD version 0.18, code is available from Eric Altermann, e.altermann@massey.ac.nz
- MEGA version 7, https://www.megasoftware.net/

Gene calling, protein translations and annotation
- Prodigal V2.6.3, https://github.com/hyattpd/Prodigal/releases
- GAMOLA2 version 2.00.09, download links available at https://www.ncbi.nlm.nih.gov/pmc/articles/PMC5362640/
- Artemis version 16.0.4, http://sanger-pathogens.github.io/Artemis/Artemis/
- dbCAN 3.0, https://bcb.unl.edu/dbCAN/
- TransportDB 2.0, http://www.membranetransport.org/transportDB2/index.html
- EffectiveT3 model 2.0.2 and EffectiveS346 in EffectiveDB, https://effectors.csb.univie.ac.at/

Further protein sequence analysis
- Geneious 8.1, https://www.geneious.com/
- ClustalW version 1.74 implemented in Geneious 8.1, http://www.clustal.org/clustal2/, https://www.geneious.com/
- MUSCLE implemented in Geneious 8.1, http://www.drive5.com/muscle/, https://www.geneious.com/
- Tree Builder in Geneious 8.1, https://www.geneious.com/
- Phobius 2004, https://phobius.sbc.su.se/
- SPOCTOPUS 2008, http://octopus.cbr.su.se/
- TOPCONS 2.0, https://topcons.net/

Simple data management and depiction
- Microsoft Word, PowerPoint, Excel (2013, 2016), Microsoft Corporation

For manuscripts utilizing custom algorithms or software that are central to the research but not yet described in published literature, software must be made available to editors and reviewers. We strongly encourage code deposition in a community repository (e.g. GitHub). See the Nature Portfolio guidelines for submitting code & software for further information.

Data

Policy information about availability of data

All manuscripts must include a data availability statement. This statement should provide the following information, where applicable:
- Accession codes, unique identifiers, or web links for publicly available datasets
- A description of any restrictions on data availability
- For clinical datasets or third party data, please ensure that the statement adheres to our policy

- The reconstructed Quinella genomes and the raw sequence data are deposited in GenBank under BioProject PRJNA373898 (https://www.ncbi.nlm.nih.gov/bioproject/PRJNA373898).
- New long length 16S rRNA gene sequences are deposited in GenBank under accessions MF184869 to MF184922 in PopSet 1199303303 (https://www.ncbi.nlm.nih.gov/popset/?term=1199303303).
- Shorter 16S rRNA gene sequences amplified from concentrated Quinella cell suspensions are deposited in GenBank under accessions OM320214 to OM320357.
- 16S rRNA genes with flanking regions amplified from the DNA preparations used to generate the reconstructed genomes are deposited in GenBank under accessions

OM303038 to OM303057.
- Previously published metatranscriptome data were from GenBank BioProject PRJNA202380 (https://www.ncbi.nlm.nih.gov/bioproject/PRJNA202380).
- Previously published 16S rRNA gene sequence data from sheep were from GenBank BioProject PRJEB4486 (https://www.ncbi.nlm.nih.gov/bioproject/PRJEB4486).

16S rRNA gene analysis and tree generation
- SILVA version 123, https://www.arb-silva.de/documentation/release-123/
- GenBank, https://www.ncbi.nlm.nih.gov/genbank/

RNA sequence analysis
- HydDB 2016, https://services.birc.au.dk/hyddb/

Genome bin phylogenomics
- GTDB Release 207, https://gtdb.ecogenomic.org/

Gene calling, protein translations and annotation
- Kyoto Encyclopedia of Genes and Genomes (KEGG), https://www.kegg.jp/blastkoala/
- MetaCyc, https://metacyc.org/
- NCBI nr protein database, https://www.ncbi.nlm.nih.gov/refseq/about/nonredundantproteins/
- COG database Release 2014, https://www.ncbi.nlm.nih.gov/research/cog/
- OrthoMCL version 1.4, https://github.com/stajichlab/OrthoMCL
- Pfam database 28.0, https://pfam.xfam.org/
- TIGRFAMs database 15.0, http://tigrfams.jcvi.org/cgi-bin/index.cgi
- UniProtKB/Swiss Prot database Release 2016_01, https://www.uniprot.org/
- CAZyDB v12, http://www.cazy.org/
- TransportDB 2.0, http://www.membranetransport.org/transportDB2/index.html
- HydDB 2016, https://services.birc.au.dk/hyddb/

Human research participants

Policy information about studies involving human research participants and Sex and Gender in Research.

Reporting on sex and gender	N/A
Population characteristics	N/A
Recruitment	N/A
Ethics oversight	N/A

Note that full information on the approval of the study protocol must also be provided in the manuscript.

Field-specific reporting

Please select the one below that is the best fit for your research. If you are not sure, read the appropriate sections before making your selection.

☒ Life sciences ☐ Behavioural & social sciences ☐ Ecological, evolutionary & environmental sciences

For a reference copy of the document with all sections, see nature.com/documents/nr-reporting-summary-flat.pdf

Life sciences study design

All studies must disclose on these points even when the disclosure is negative.

Sample size	Sample size was not predetermined using statistical methods and the experiments were not randomised. All details of sample sizes are given in the paper. DNA for genome reconstruction was extracted from different sample combinations, originating from 12 sheep from an original set of 24. This was chosen as a practical number to process in parallel. A total of 85 genome bins were generated. Three genome bins that originated from Quinella and met the quality criteria were included in the study together with one additional Quinella genome bin that had 10.3% contamination (the target was 10% or less). This determined the number of genome bins used for genome annotation.
Data exclusions	Genome bins other than the four listed in Sample Size were excluded because they did not reach the targeted criteria for completeness and contamination, or were not assigned to the target genus Quinella. Since the aim was to make a first study the potential physiology of this genus, the data were limited to the four genome bins that could be assigned to this genus with confidence.
Replication	Light microscopy was performed on samples from 7 different sheep, while electron microscopy was performed on a sample from one sheep. All

amplicon data were pooled, and statistical analyses were not made as part of this study. Four genome bins ascribed to Quinella were used to provide a consensus view from the reconstructed genomes.

Randomization	Randomization was not necessary for the experiments. Randomization is not relevant in genomic, transcriptomic and phylogenetic studies that are not testing hypotheses. This study was targeted to providing data on Quinella, and so the experimental flow was directed towards that goal. Sample size was not predetermined using statistical methods and the experiments were not randomised. A total of 85 genome bins were generated. Three genome bins that originated from Quinella and met the quality criteria were included in the study together with one additional Quinella genome bin that had 10.3% contamination (the target was 10% or less). This determined the number of genome bins used for genome annotation.
Blinding	Blinding was not performed as it was not necessary. All experimental conditions were known to the researchers. This study was targeted to providing data on Quinella, and so the experimental flow was directed to that goal.

Behavioural & social sciences study design

All studies must disclose on these points even when the disclosure is negative.

Study description	This study was not a behavioural and social sciences study.
Research sample	This study was not a behavioural and social sciences study.
Sampling strategy	This study was not a behavioural and social sciences study.
Data collection	This study was not a behavioural and social sciences study.
Timing	This study was not a behavioural and social sciences study.
Data exclusions	This study was not a behavioural and social sciences study.
Non-participation	This study was not a behavioural and social sciences study.
Randomization	This study was not a behavioural and social sciences study.

Ecological, evolutionary & environmental sciences study design

All studies must disclose on these points even when the disclosure is negative.

Study description	This study was not an ecological, evolutionary & environmental sciences study.
Research sample	This study was not an ecological, evolutionary & environmental sciences study.
Sampling strategy	This study was not an ecological, evolutionary & environmental sciences study.
Data collection	This study was not an ecological, evolutionary & environmental sciences study.
Timing and spatial scale	This study was not an ecological, evolutionary & environmental sciences study.
Data exclusions	This study was not an ecological, evolutionary & environmental sciences study.
Reproducibility	This study was not an ecological, evolutionary & environmental sciences study.
Randomization	This study was not an ecological, evolutionary & environmental sciences study.
Blinding	This study was not an ecological, evolutionary & environmental sciences study.

Did the study involve field work? ☐ Yes ☒ No

Field work, collection and transport

Field conditions	Describe the study conditions for field work, providing relevant parameters (e.g. temperature, rainfall).
Location	State the location of the sampling or experiment, providing relevant parameters (e.g. latitude and longitude, elevation, water depth).

Access & import/export | *Describe the efforts you have made to access habitats and to collect and import/export your samples in a responsible manner and in compliance with local, national and international laws, noting any permits that were obtained (give the name of the issuing authority, the date of issue, and any identifying information).*

Disturbance | *Describe any disturbance caused by the study and how it was minimized.*

Reporting for specific materials, systems and methods

We require information from authors about some types of materials, experimental systems and methods used in many studies. Here, indicate whether each material, system or method listed is relevant to your study. If you are not sure if a list item applies to your research, read the appropriate section before selecting a response.

Materials & experimental systems

n/a	Involved in the study
☒	☐ Antibodies
☒	☐ Eukaryotic cell lines
☒	☐ Palaeontology and archaeology
☐	☒ Animals and other organisms
☒	☐ Clinical data
☒	☐ Dual use research of concern

Methods

n/a	Involved in the study
☒	☐ ChIP-seq
☒	☐ Flow cytometry
☒	☐ MRI-based neuroimaging

Antibodies

Antibodies used | *Describe all antibodies used in the study; as applicable, provide supplier name, catalog number, clone name, and lot number.*

Validation | *Describe the validation of each primary antibody for the species and application, noting any validation statements on the manufacturer's website, relevant citations, antibody profiles in online databases, or data provided in the manuscript.*

Eukaryotic cell lines

Policy information about cell lines and Sex and Gender in Research

Cell line source(s) | *State the source of each cell line used and the sex of all primary cell lines and cells derived from human participants or vertebrate models.*

Authentication | *Describe the authentication procedures for each cell line used OR declare that none of the cell lines used were authenticated.*

Mycoplasma contamination | *Confirm that all cell lines tested negative for mycoplasma contamination OR describe the results of the testing for mycoplasma contamination OR declare that the cell lines were not tested for mycoplasma contamination.*

Commonly misidentified lines (See ICLAC register) | *Name any commonly misidentified cell lines used in the study and provide a rationale for their use.*

Palaeontology and Archaeology

Specimen provenance | *Provide provenance information for specimens and describe permits that were obtained for the work (including the name of the issuing authority, the date of issue, and any identifying information). Permits should encompass collection and, where applicable, export.*

Specimen deposition | *Indicate where the specimens have been deposited to permit free access by other researchers.*

Dating methods | *If new dates are provided, describe how they were obtained (e.g. collection, storage, sample pretreatment and measurement), where they were obtained (i.e. lab name), the calibration program and the protocol for quality assurance OR state that no new dates are provided.*

☐ Tick this box to confirm that the raw and calibrated dates are available in the paper or in Supplementary Information.

Ethics oversight | *Identify the organization(s) that approved or provided guidance on the study protocol, OR state that no ethical approval or guidance was required and explain why not.*

Note that full information on the approval of the study protocol must also be provided in the manuscript.

Animals and other research organisms

Policy information about studies involving animals; ARRIVE guidelines recommended for reporting animal research, and Sex and Gender in Research

Laboratory animals	*Female cattle aged 10 years and female sheep aged 13 months were used in this study.*
Wild animals	*No wild animals were used in this study.*
Reporting on sex	*All cattle and sheep were females.*
Field-collected samples	*Thus study did not involve samples collected from the field.*
Ethics oversight	*All procedures involving animals were approved by the AgResearch Grasslands Animal Ethics Committee, Palmerston North, New Zealand, and adhered to the guidelines of the 1999 New Zealand Animal Welfare Act and AgResearch Code of Ethical Conduct. The collection of rumen contents from fistulated cows for culture media preparation was approved under animal ethics approval AE13398. The collection of rumen samples from sheep for metagenomic analyses, cultivation attempts, and microscopy was under animal ethics approvals AE11975 and AE13282.*

Note that full information on the approval of the study protocol must also be provided in the manuscript.

Clinical data

Policy information about clinical studies

All manuscripts should comply with the ICMJEguidelines for publication of clinical research and a completedCONSORT checklist must be included with all submissions.

Clinical trial registration	*Provide the trial registration number from ClinicalTrials.gov or an equivalent agency.*
Study protocol	*Note where the full trial protocol can be accessed OR if not available, explain why.*
Data collection	*Describe the settings and locales of data collection, noting the time periods of recruitment and data collection.*
Outcomes	*Describe how you pre-defined primary and secondary outcome measures and how you assessed these measures.*

Dual use research of concern

Policy information about dual use research of concern

Hazards

Could the accidental, deliberate or reckless misuse of agents or technologies generated in the work, or the application of information presented in the manuscript, pose a threat to:

No	Yes	
☒	☐	Public health
☒	☐	National security
☒	☐	Crops and/or livestock
☒	☐	Ecosystems
☒	☐	Any other significant area

Experiments of concern

Does the work involve any of these experiments of concern:

No	Yes	
☒	☐	Demonstrate how to render a vaccine ineffective
☒	☐	Confer resistance to therapeutically useful antibiotics or antiviral agents
☒	☐	Enhance the virulence of a pathogen or render a nonpathogen virulent
☒	☐	Increase transmissibility of a pathogen
☒	☐	Alter the host range of a pathogen
☒	☐	Enable evasion of diagnostic/detection modalities
☒	☐	Enable the weaponization of a biological agent or toxin
☒	☐	Any other potentially harmful combination of experiments and agents

ChIP-seq

Data deposition

☐ Confirm that both raw and final processed data have been deposited in a public database such as GEO.

☐ Confirm that you have deposited or provided access to graph files (e.g. BED files) for the called peaks.

Data access links<br>*May remain private before publication.*	*For "Initial submission" or "Revised version" documents, provide reviewer access links. For your "Final submission" document, provide a link to the deposited data.*
Files in database submission	*Provide a list of all files available in the database submission.*
Genome browser session<br>(e.g. UCSC)	*Provide a link to an anonymized genome browser session for "Initial submission" and "Revised version" documents only, to enable peer review. Write "no longer applicable" for "Final submission" documents.*

Methodology

Replicates	*Describe the experimental replicates, specifying number, type and replicate agreement.*
Sequencing depth	*Describe the sequencing depth for each experiment, providing the total number of reads, uniquely mapped reads, length of reads and whether they were paired- or single-end.*
Antibodies	*Describe the antibodies used for the ChIP-seq experiments; as applicable, provide supplier name, catalog number, clone name, and lot number.*
Peak calling parameters	*Specify the command line program and parameters used for read mapping and peak calling, including the ChIP, control and index files used.*
Data quality	*Describe the methods used to ensure data quality in full detail, including how many peaks are at FDR 5% and above 5-fold enrichment.*
Software	*Describe the software used to collect and analyze the ChIP-seq data. For custom code that has been deposited into a community repository, provide accession details.*

Flow Cytometry

Plots

Confirm that:

☐ The axis labels state the marker and fluorochrome used (e.g. CD4-FITC).

☐ The axis scales are clearly visible. Include numbers along axes only for bottom left plot of group (a 'group' is an analysis of identical markers).

☐ All plots are contour plots with outliers or pseudocolor plots.

☐ A numerical value for number of cells or percentage (with statistics) is provided.

Methodology

Sample preparation	*Describe the sample preparation, detailing the biological source of the cells and any tissue processing steps used.*
Instrument	*Identify the instrument used for data collection, specifying make and model number.*
Software	*Describe the software used to collect and analyze the flow cytometry data. For custom code that has been deposited into a community repository, provide accession details.*
Cell population abundance	*Describe the abundance of the relevant cell populations within post-sort fractions, providing details on the purity of the samples and how it was determined.*
Gating strategy	*Describe the gating strategy used for all relevant experiments, specifying the preliminary FSC/SSC gates of the starting cell population, indicating where boundaries between "positive" and "negative" staining cell populations are defined.*

☐ Tick this box to confirm that a figure exemplifying the gating strategy is provided in the Supplementary Information.

Magnetic resonance imaging

Experimental design

Design type	*Indicate task or resting state; event-related or block design.*

Design specifications	*Specify the number of blocks, trials or experimental units per session and/or subject, and specify the length of each trial or block (if trials are blocked) and interval between trials.*
Behavioral performance measures	*State number and/or type of variables recorded (e.g. correct button press, response time) and what statistics were used to establish that the subjects were performing the task as expected (e.g. mean, range, and/or standard deviation across subjects).*

Acquisition

Imaging type(s)	*Specify: functional, structural, diffusion, perfusion.*
Field strength	*Specify in Tesla*
Sequence & imaging parameters	*Specify the pulse sequence type (gradient echo, spin echo, etc.), imaging type (EPI, spiral, etc.), field of view, matrix size, slice thickness, orientation and TE/TR/flip angle.*
Area of acquisition	*State whether a whole brain scan was used OR define the area of acquisition, describing how the region was determined.*

Diffusion MRI ☐ Used ☐ Not used

Preprocessing

Preprocessing software	*Provide detail on software version and revision number and on specific parameters (model/functions, brain extraction, segmentation, smoothing kernel size, etc.).*
Normalization	*If data were normalized/standardized, describe the approach(es): specify linear or non-linear and define image types used for transformation OR indicate that data were not normalized and explain rationale for lack of normalization.*
Normalization template	*Describe the template used for normalization/transformation, specifying subject space or group standardized space (e.g. original Talairach, MNI305, ICBM152) OR indicate that the data were not normalized.*
Noise and artifact removal	*Describe your procedure(s) for artifact and structured noise removal, specifying motion parameters, tissue signals and physiological signals (heart rate, respiration).*
Volume censoring	*Define your software and/or method and criteria for volume censoring, and state the extent of such censoring.*

Statistical modeling & inference

Model type and settings	*Specify type (mass univariate, multivariate, RSA, predictive, etc.) and describe essential details of the model at the first and second levels (e.g. fixed, random or mixed effects; drift or auto-correlation).*
Effect(s) tested	*Define precise effect in terms of the task or stimulus conditions instead of psychological concepts and indicate whether ANOVA or factorial designs were used.*

Specify type of analysis: ☐ Whole brain ☐ ROI-based ☐ Both

Statistic type for inference (See Eklund et al. 2016)	*Specify voxel-wise or cluster-wise and report all relevant parameters for cluster-wise methods.*
Correction	*Describe the type of correction and how it is obtained for multiple comparisons (e.g. FWE, FDR, permutation or Monte Carlo).*

Models & analysis

n/a	Involved in the study
☐	☐ Functional and/or effective connectivity
☐	☐ Graph analysis
☐	☐ Multivariate modeling or predictive analysis

Functional and/or effective connectivity	*Report the measures of dependence used and the model details (e.g. Pearson correlation, partial correlation, mutual information).*
Graph analysis	*Report the dependent variable and connectivity measure, specifying weighted graph or binarized graph, subject- or group-level, and the global and/or node summaries used (e.g. clustering coefficient, efficiency, etc.).*
Multivariate modeling and predictive analysis	*Specify independent variables, features extraction and dimension reduction, model, training and evaluation metrics.*
